# The WorkReach model for urban work location choices through economic complexity and informality

**DOI:** 10.1038/s41467-026-78293-3

**Published:** 2026-10-07

**Authors:** Ollin D. Langle-Chimal, Steffen Knoblauch, Marta C. González

**Affiliations:** 1 Department of Civil and Environmental Engineering, University of California, Berkeley, Berkeley, CA USA; 2 Interdisciplinary Centre of Scientific Computing (IWR), Heidelberg University, Heidelberg, Germany; 3 Heidelberg Institute for Geoinformation Technology, Heidelberg University, Heidelberg, Germany; 4 Department of City and Regional Planning, University of California, Berkeley, Berkeley, CA USA

**Keywords:** Decision making, Scientific data, Geography, Social sciences

## Abstract

Human mobility in urban areas largely depends on the spatial distribution of jobs and who pursues them, yet how workers balance proximity against economic opportunity remains poorly understood. Existing mechanistic models capture physically interpretable effects such as distance decay, but overlook how socioeconomic factors shape commuting decisions. We present WorkReach, a model grounded in discrete choice theory that embeds informality and economic complexity into a utility function and represents commuting as the outcome of workers behaving as if maximizing it. Applied to four cities in the United States, Mexico, and Brazil, it reproduces commuting flows as accurately as widely used benchmarks while making the underlying choices interpretable. Here, we show that workers consistently commute farther to economically sophisticated areas, while the role of informality differs across regions, and that measuring accessibility by the perceived benefit of opportunities, not just physical proximity, highlights disparities conventional metrics overlook.

## Abstract (web summary)

The WorkReach model captures how economic opportunity and informality shape commuting. Analyzing four major cities, this study shows that measuring job access by perceived benefits, not just proximity, exposes hidden socioeconomic disparities.

## Introduction

The decision of where to live and work is a fundamental aspect of urban life and has great consequences at the individual and collective urban dynamics. This choice is often a complex trade-off, balancing the desire for proximity with the pursuit of better economic opportunities, all within a landscape defined by varying labor market conditions^1^. Understanding these trade-offs is crucial for addressing spatial inequalities in employment accessibility, particularly in metropolitan regions characterized by high socioeconomic heterogeneity. Factors such as the prevalence of informal labor and the spatial distribution of the economic desirability of work opportunities can create large disparities in the access to quality jobs, which greatly affects economic stability and social mobility^2–4^. Regardless of the importance of these socioeconomic dimensions in shaping urban mobility patterns, current mobility flow modeling approaches do not take them into account.

Traditional spatial interaction models, particularly gravity and radiation^5^ models are known for their intuitive formulation and parsimony^6–9^, which has led to their popularity in urban and transportation planning. Gravity models, as the name suggests, are inspired by Newtonian gravity, modeling mobility flows between origins and destinations as a function of their population (or mass) and a distance-decay component. Radiation models, in contrast, are parameter-free and rely on the number of intervening opportunities located closer to the origin^10^. While the accuracy and general interpretability of these models have long been studied and described^7,11^, they provide limited information on the behavioral mechanisms that drive these flows, particularly when socioeconomic factors are taken into account. Recent advances in the field, including deep learning approaches such as Deep Gravity^12^ characterized by being data intensive and other based on symbolic regression such as the gravity-like models found by the Bayesian Machine Scientist (BMS)^13^, have pushed the limits of predictive accuracy. However, this increased accuracy doesn’t necessarily offer greater interpretability of the underlying decision-making processes.

In order to address these limitations, we present WorkReach, a model designed to provide behavioral insights into urban work location choices. WorkReach accounts for the trade-offs commuters make between distance and work quality by conditioning work location choices on residential locations and their associated socioeconomic characteristics. We apply this framework to analyze commuting patterns across four urban areas; the San Francisco Bay Area, Los Angeles, Mexico City, and Rio de Janeiro. These cities represent different regions, physical characteristics, and structures of the urban labor market.

This study prioritizes model interpretability and the extraction of actionable insights. To this end, we ground our approach in discrete choice methods, which rely on the theory of Random Utility Models (RUM), by integrating key socioeconomic variables directly into a utility function that commuters are assumed to maximize, so that the resulting parameters approximate the aggregate trade-offs reflected in observed commuting patterns^14,15^. Specifically, we incorporate two critical, yet not commonly studied, dimensions in flow modeling: economic complexity and labor informality. Economic complexity, a measure of the diversity and sophistication of a region’s economy^16,17^, serves as a useful proxy for the economic quality of work opportunities. Although earlier work has used economic complexity to study cities as a whole^18^, its application at the sub-city level is just starting to be explored^19–23^, revealing fine-grained differences in urban economies. The trade-off between commuting distance and wages is well established in the labor economics literature^24,25^, but wages capture only current compensation. Economic complexity, by contrast, is derived from the productive structure of each zone and therefore reflects location-level advantages that current pay does not internalize. Agglomeration theory states that zones where diverse and complementary industries co-locate generate productivity externalities from sectoral complementarities, lower job-search costs by concentrating a wider range of employment opportunities, and facilitate human capital accumulation through exposure to a broader set of economic activities^26,27^.

Labor informality is defined as the employment outside formal regulatory frameworks and lacking social protections^28^. It is a widespread characteristic of urban economies, particularly in Latin America^2^. However, despite its documented presence, informality remains less systematically studied in high-income countries such as the United States^3,4^. By incorporating economic complexity at the work location and informality at the home location into a utility function, our model quantifies how these factors, along with commuting distance, shape work location choices. This approach let us estimate a set of parameters that are interpretable and useful to quantify the trade-offs workers are willing to make. For example, we can measure how much additional distance a worker would travel for a work in a more economically complex area, and how this willingness is modulated by the informality context of their home location. These behavioral insights are crucial to understanding how the aggregation of individual decisions shapes macro-level mobility patterns and the socioeconomic stratification of a city^29–31^.

Another key advantage of the WorkReach model is that it allows us to derive economically meaningful work accessibility metrics based on a perceived satisfaction of the decision maker. Accessibility metrics usually focus on the number of opportunities reachable from a specific location within a certain travel time or physical distance^6,32,33^. Discrete choice models, on the other hand, directly derive accessibility through consumer-surplus, which reflects the expected maximum utility an agent gains from the whole set of available work choices^34–37^. These measures provide a more holistic understanding of disparities in access to opportunities by not only accounting for proximity but also the perceived quality of the choices^38,39^.

Here, we show both fundamental differences and striking similarities between the cities and regions studied. For example, we find that while workers in the U.S. cities weigh economic opportunity across the whole distance spectrum, commuters in the Latin American cities operate within a proximity-first regime. This divergence can be explained with the role of informality in the utility function. In Mexico City and Rio de Janeiro, informal workers perceive a utility gain by undertaking longer commutes to access high-complexity jobs, whereas in U.S. cities, this behavior is observed among workers from low-informality areas. Our framework allows for a quantification of these behavioral nuances, showing, for instance, that a worker in Mexico City is willing to trade nearly four times the distance for a one-unit gain in economic complexity compared to a worker in Los Angeles, or that a reduction of economic complexity of 1% in a work location in the Bay Area is associated with a 0.804% reduction in the probability of a commuter to select that specific location as their work choice. Despite these regional differences, our utility-based accessibility metric indicates that home locations from high-informality workers experience a lower consumer-surplus accessibility in three of the four cities, translating into an overall lower satisfaction with the work market. Work location choices are shaped by co-existing, complementary influences, including the economic complexity of the destination and the sectoral relatedness between home and work locations, the similarity of their sector portfolios. Relatedness reproduces the flows slightly more closely than economic complexity, yet we focus on complexity because, as an intrinsic destination attribute, it is an actionable lever for spatially targeted policy. WorkReach is therefore not another flow-prediction model; it is a framework for interpreting the trade-offs reflected in these flows and translating them into accessibility and policy.

## Results

### Study areas

Our study focuses on four major urban areas that are comparable in population size: Mexico City and the municipality of Rio de Janeiro in Latin America, and Los Angeles and the San Francisco Bay Area in the United States. These areas of interest represent diverse urban landscapes and economic structures with different characteristics in their labor markets, giving us a rich comparative context. Mexico City and Rio de Janeiro are two megacities in middle income economies that have very high levels of informal employment and population density^40^. Los Angeles and the Bay Area, in contrast, are major economic hubs in a high income country, with substantial socioeconomic disparities and distinct patterns of suburbanization and polycentricity ^41,42^. Informality is less systematically tracked or studied in the U.S. compared to Latin America, yet California is one of the States where researchers have estimated its prevalence^3^. For Mexico City, Los Angeles, and Rio de Janeiro, the study area corresponds to the city proper, while the San Francisco Bay Area encompasses multiple cities within a multi-county region. This choice prioritizes comparability in population scale and number of spatial units across the four cities. The composition of these urban areas (Table 1) allows us to examine the differences and similarities that make up the economic decision of commuters to choose their work location based on distance from their home location, economic opportunity, and the prevalence of informality of the job.

**Table 1:** Key descriptive statistics for the four mobility datasets

	Bay Area	Los Angeles	Mexico City	Rio de Janeiro
Links (origin–destination, OD pairs)	457,652	365,420	628,056	39,402
Nodes (Locations)	677	605	793	199
Area (median/mean [km^2^])	5.41 / 27.18	5.77 / 16.56	0.85 / 0.98	2.93 / 6.03
Total area [km^2^]	18,402.05	10,020.90	776.94	1199.24
Flow range (min–max)	1–40,119	1–21,036	1–3243	6–9780
Total observed flows	1,936,002	2,388,812	473,078	357,005
Median OD distance [km]	11.92	10.39	4.12	4.08
Diameter [km]	283.22	145.42	52.62	73.65
Population range (min–max)	39–100,177	5–297,978	9–38,381	3361–65,846
Total population	7,432,481	10,088,110	8,995,924	6,289,038

Total population refers to the sum within the study area boundaries.

### Economic complexity framework

We summarize each work location zone’s productive structure with the economic complexity framework^16^, which yields an Economic Complexity Index (ECI^emp^) for sub-city zones and a Product Complexity Index (PCI^emp^) for economic sectors. To reflect their use of employment data and distinguish them from the original formulation, we denote them ECI^emp^ and PCI^emp^.

**Fig. 1:**
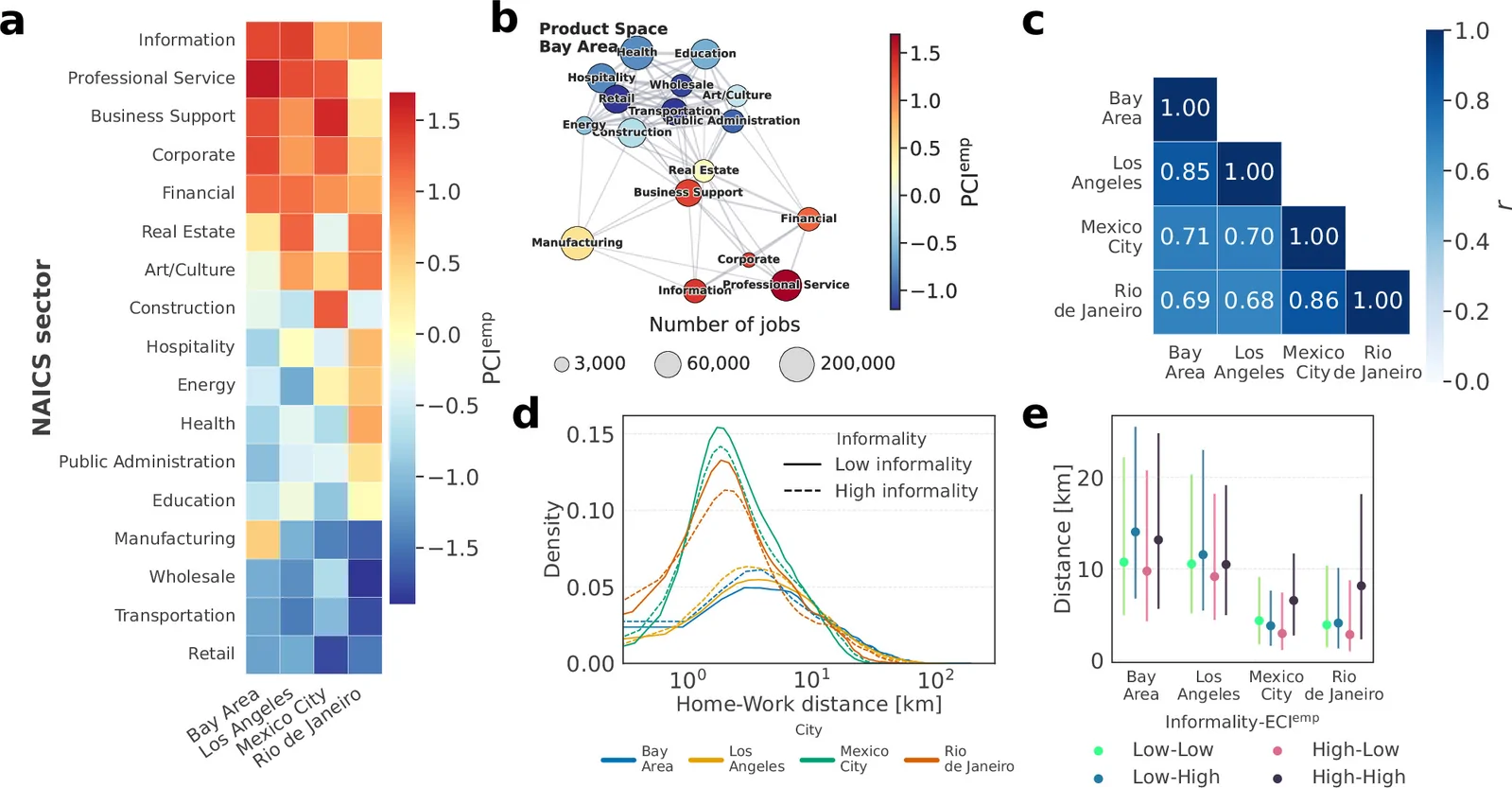
Multi-scale structure of productive specialization and commuting in four cities of study. **a** Product-complexity heat-map across 17 North American Industry Classification System (NAICS) sectors and cities, with colors representing Product Complexity Index (PCI^emp^). **b** Product-space network for the Bay Area, where node size reflects workforce and color indicates PCI^emp^; edges represent sector co-location probabilities. **c** Correlation matrix of sector co-location patterns showing high within-region similarity (U.S. cities *r* = 0.85; Latin American cities *r* = 0.86) and lower cross-regional correlations (*r* = 0.68–0.71). Correlations are Pearson coefficients between the proximity matrices of each pair of cities, computed over the 136 unique sector pairs of the 17 NAICS sectors, giving 134 degrees of freedom; all are significant at *p* < 0.001 under a two-sided test, with no correction for multiple comparisons. **d** Commuting distance distributions by origin informality level (solid = low, dashed = high), showing heavier right tails in U.S. cities. **e** Median commuting distances differ across informality and economic complexity (ECI^emp^) groups: longest commutes occur in high-informality/high-ECI^emp^ areas in Latin America versus low-informality/high-ECI^emp^ areas in the U.S., reflecting different socio-spatial segregation patterns. Groups combine origin informality and destination ECI^emp^, each split at its city-specific median. Points are medians and vertical bars span the interquartile range. The unit of analysis is the individual commuter trip. In the order (Informality-ECI^emp^) Low-Low, Low-High, High-Low and High-High, *n* = 484,303; 485,515; 486,136; and 479,247 trips for the Bay Area; 608,916; 586,164; 585,396; and 606,555 for Los Angeles; 99,106; 136,756; 137,151; and 97,846 for Mexico City; and 73,290; 105,975; 105,418; and 71,682 for Rio de Janeiro. Trips whose origin has no informality value or whose destination has no ECI^emp^ value are excluded (801; 1,781; 2,219; and 640 trips respectively).

We apply this framework to 17 standardized two-digit North American Industry Classification System (NAICS) sectors and find distinct specialization patterns. For example, the Bay Area shows a higher than average PCI^emp^ in the information sector, reflecting its tech-driven economy. In the product-space network (Fig. 1b), where sectors are linked by how often they co-locate across zones, highly complex sectors cluster closely, as do less complex ones. The correlation matrix of these co-location patterns across cities, shown in Fig. 1c), reflects regional similarities: the Bay Area and Los Angeles exhibit a high similarity (Pearson correlation coefficient *r* = 0.85), and Mexico City and Rio de Janeiro show an equally strong correlation (*r* = 0.86). Cross-regional correlations are lower (*r* = 0.68–0.71), indicating that U.S. and Latin American cities share the broad sectoral co-location structure but differ in the finer details of which sectors cluster together.

The informality rate, economic complexity, and commuting flows are constructed from census, employment-registry, mobility, and synthetic data sources; full details are given in the “Methods.”

### Descriptive statistics and spatial distributions

Table 1 provides key descriptive statistics for the four cities. The number of origin-destination links and unique geostatistical nodes varies, reflecting differences in urban scale and data granularity. Rio de Janeiro has the fewest links and nodes, while Mexico City has the most. In all four cities the number of links corresponds to a complete graph without self-loops: every pair of locations is connected by at least one observed commute. Locations without incoming or outgoing trips are discarded. The median and mean area of these nodes show the spatial resolution of every city; Mexico City has the finest resolution shown by the smaller average area, as we map all zones to Uber’s H3 hexagonal grid (Methods) and use a finer H3 resolution for Mexico City (level 8) than for the U.S. areas (level 7), due to the small overall diameter of the area covered; for Rio de Janeiro we retain the original Área de Estatística e Planeamento (AEP) polygons, which are already coarse, since aggregating them further would substantially reduce the number of zones and introduce census estimation errors from redistributing their values across cells. Where an original zone spans several H3 cells, we preserve its scale by merging the contained hexagons into a single unit rather than splitting it. Flow counts, commute distances, and populations show wide ranges within and between cities, highlighting the heterogeneous nature of commute patterns. Los Angeles and the Bay Area exhibit the longest maximum commute distances, consistent with their larger areas and polycentric nature^42^.

**Fig. 2:**
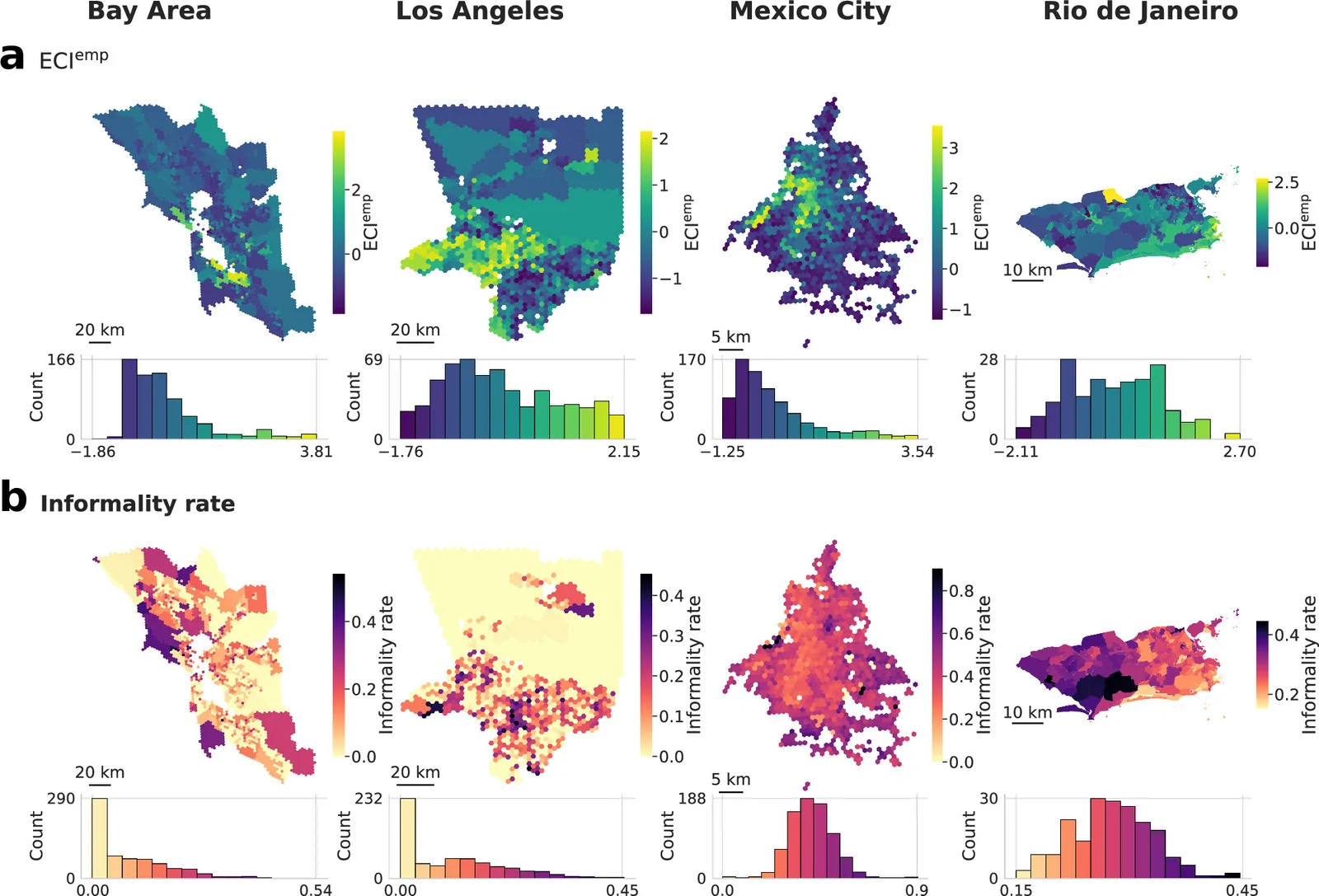
Choropleth maps depicting the Economic Complexity Index (ECI^emp^) and informality rate for each geographical unit within the study cities. The inset histograms illustrate the overall distributions. **a** Economic Complexity Index **b** Informality rate. Spatial units are H3 level 7 hexagons for the Bay Area and Los Angeles, H3 level 8 hexagons for Mexico City, and Área de Estatística e Planeamento (AEP) polygons for Rio de Janeiro. Where a single census geometry intersects several hexagons, those hexagons are merged into one unit that covers it. Color scales are set independently for each city, so values are comparable within a panel but not across cities. Each map shows the full study area, which is the city proper for Los Angeles, Mexico City and Rio de Janeiro and the multi-county San Francisco Bay Area region described in “Methods”.

The spatial distribution of ECI^emp^ and informality rates, shown in Fig. 2 varies across cities. Both variables have statistically significant spatial autocorrelation in all cities, as indicated by Moran’s I, which indicates that similar characteristics tend to cluster together, especially in the Latin American region (Supplementary Table 1 and Supplementary Figs. 1–8). However, the spatial correlation between both variables differs greatly by region. Mexico City and Rio de Janeiro show a moderate negative correlation between informality rates and ECI^emp^ (*r* ~ −0.4), suggesting spatial segregation where informal workers live far away from high-complexity economic areas. In contrast, the correlation between these two variables in the U.S. cities is close to zero (*r* ~ 0), which suggests that the spatial distribution of informal workers is less systematically excluded from high economically complex areas. This points to different forms of socio-spatial organization: whereas Latin American cities display sharper patterns of economic segregation, U.S. cities exhibit a more mixed spatial arrangement of formal and informal employment.

### Mobility patterns and socioeconomic characteristics

Mobility patterns also vary systematically with socioeconomic characteristics. U.S. cities generally feature longer commutes compared to their Latin American counterparts (Fig. 1d). However, disaggregating commutes by origin informality and work location complexity highlights an interesting pattern (Fig. 1e). In all four cities, workers from high-informality origins traveling to low-complexity destinations have the shortest median commutes, but those who face the longest median commutes varies between regions. In Mexico City and Rio de Janeiro, the longest commutes are typically made by workers from high-informality areas traveling to high-ECI^emp^ destinations, indicating that vulnerable populations often endure longer commutes to reach better opportunities. In contrast, in the Bay Area and Los Angeles, the longest commutes are taken by workers from low-informality areas traveling to high-ECI^emp^ destinations, reflecting the behavior of affluent, formally employed suburban commuters. These patterns highlight the importance of understanding how workers, faced with different conditions when choosing where to live and work, respond to varying needs and constraints in their work location decisions across different economies.

### Estimated coefficients

**Fig. 3:**
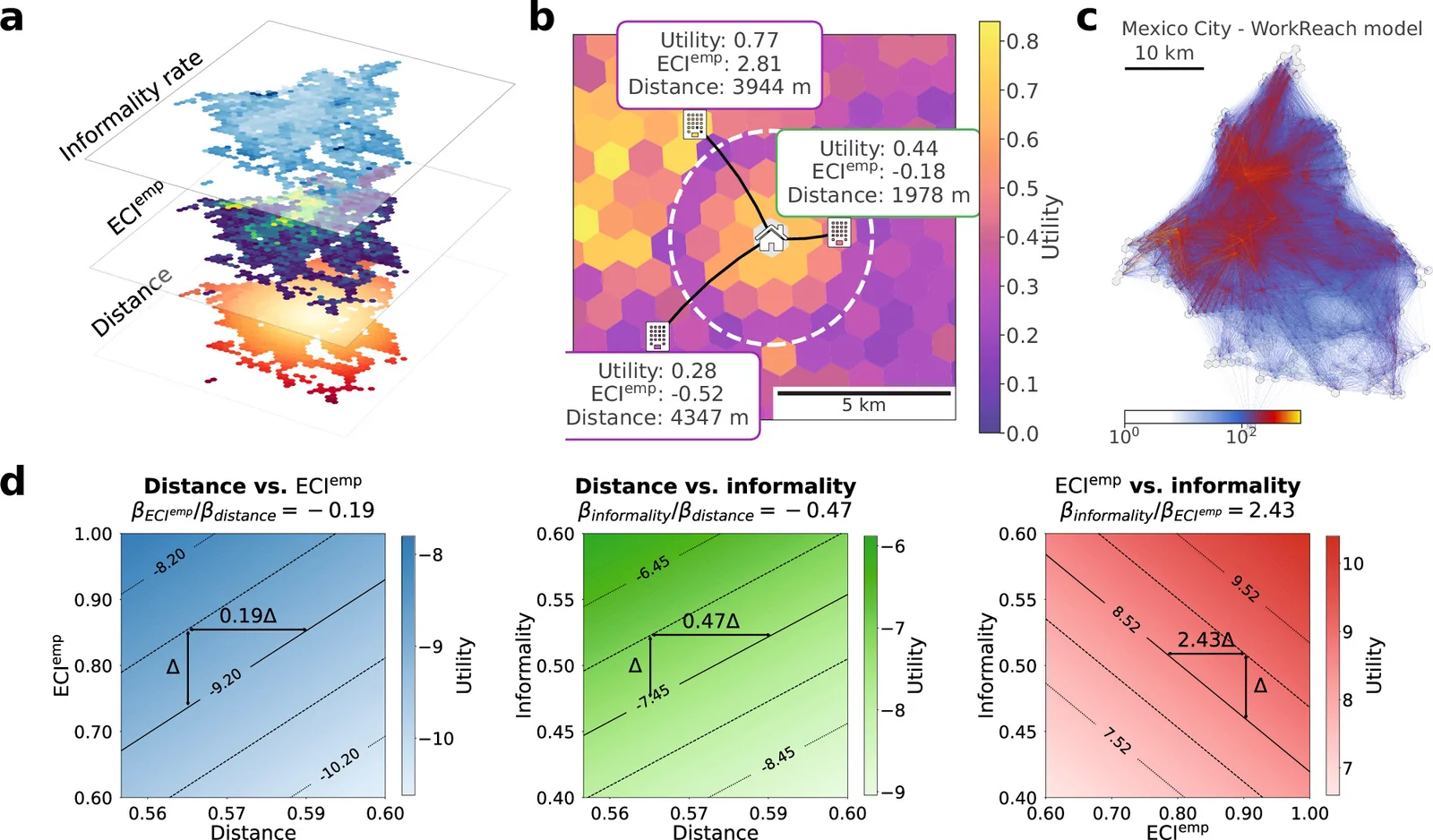
Conceptual overview of the WorkReach model. **a** Stacked data inputs: commuting distance, economic complexity (ECI^emp^), and informality rate. Distance is illustrated for a given home location. **b** Utility calculation showing distance threshold *τ* (white dashed circle) dividing choice set into the proximity-first and opportunity-first regimes. Beyond the threshold, transition weight *w*_*ij*_(*τ*, *k*) modulates distance influence while utility incorporates ECI^emp^ and informality effects. The panel is drawn on Mexico City’s H3 level 8 hexagons using illustrative values, so it shows how the utility is built rather than a fitted result. **c** Predicted origin-destination commuting network for Mexico City, on H3 level 8 hexagons, with edge intensity representing expected commuter flows. **d** Iso-utility contours in three attribute planes (distance vs. ECI^emp^; distance vs. informality; ECI^emp^ vs. informality) showing trade-offs that maintain constant utility in the opportunity-first regime (*w*_*ij*_ = 1).

WorkReach turns these patterns into a discrete choice model^15^. A multinomial logit gives every work zone *j* a probability of being chosen from home zone *i* that increases with a utility we measure as a combination of three terms, the commuting distance, the economic complexity of the destination and the informality rate at the origin, with coefficients *β*_dist_, *β*_ECI_ and ${\beta }_{\inf }$. Economic complexity and informality enter the utility through a transition weight that rises with distance along a logistic curve, set by a distance threshold *τ* and a steepness *k*. The threshold splits each choice set into nearby and distant work locations (Fig. 3b), so nearby ones are chosen mainly on proximity while distant ones trade extra travel against economic opportunity. This is motivated by the empirical result in Fig. 1e, which shows that the shortest commutes in all four cities run from high-informality origins to low-complexity destinations, while the longest ones reach high-complexity destinations everywhere, even though the workers who make them differ by region. We estimate all five parameters, *β*_dist_, *β*_ECI_, ${\beta }_{\inf }$, *τ* and *k*, by maximizing the log-likelihood of the observed flows. We give the full specification and the estimation procedure in the Methods, and Supplementary Fig. 15 shows the calibrated transition weight for each city.

We min-max scale distance, ECI^emp^ and informality to [0, 1] before estimation, so each coefficient refers to moving that variable across its full range within a city. Distance acts on utility directly through *β*_dist_ and also indirectly, by shifting the transition weight, while *β*_ECI_ and ${\beta }_{\inf }$ take effect mainly among the distant work locations, where that weight is close to one. The estimated coefficients of our calibrated model (Table 2) capture different behavioral patterns across cities. We compute 95% confidence intervals using a row-wise multinomial bootstrap in which origin-level destination shares are resampled conditional on observed home populations and the model is re-estimated for each replicate. The distance coefficient (*β*_dist_) is negative and relatively large in all four cities. This result indicates, as expected, that the utility decreases with distance. This coefficient has its largest magnitude value in Mexico City (*β*_dist_ = −21.881) and smallest in Los Angeles (*β*_dist_ = −9.601). The economic complexity coefficient (*β*_ECI_) is positive in all four cities, indicating that higher-ECI^emp^ work locations are consistently associated with a higher probability of being chosen as a workplace. Interestingly, the informality coefficient (${\beta }_{\inf }$) shows different regional patterns. In Mexico City and Rio de Janeiro, it has a positive value (10.250 and 4.267, respectively), consistent with workers in areas of high-informality traveling longer distances to find complex work opportunities. In contrast, the coefficient is negative in the Bay Area and Los Angeles, which indicates that high-informality areas in these cities are less prone to travel long distances for high-ECI^emp^ work alternatives. This could be due more spatial integration of informal workers or the willingness of low informality workers to live far away from high-ECI^emp^ areas and closer to strictly residential zones. This regional contrast is consistent with high-ECI^emp^ destinations offering a broader and denser set of employment opportunities, reducing matching costs for workers with fewer local options. In Latin American cities, where high-informality residential zones are spatially segregated from high-ECI^emp^ employment hubs, workers from those zones are willing to overcome larger geographic barriers to access them (Supplementary Note 11).

**Table 2:** Optimized utility-model coefficients, substitution rates, and elasticities for the four cities

	Bay Area	Los Angeles	Mexico City	Rio de Janeiro
Coefficients:				
*β*_dist_	−21.292	−9.601	−21.881	−16.447
95% CI	[−21.298, −21.279]	[−9.607, −9.592]	[−21.899, −21.864]	[−16.454, −16.434]
*β*_ECI_	2.462	0.482	4.220	6.603
95% CI	[2.460, 2.463]	[0.480, 0.484]	[4.211, 4.232]	[6.584, 6.620]
${\beta }_{\inf }$	−14.735	−8.702	10.250	4.267
95% CI	[−14.767, −14.702]	[−8.721, −8.692]	[10.235, 10.270]	[4.254, 4.284]
*τ* [km]	0.006	0.006	16.201	24.955
95% CI	[0.006, 0.006]	[0.006, 0.006]	[16.179, 16.217]	[24.911, 24.997]
*k*	128.764	32.413	10.824	9.654
95% CI	[128.761, 128.766]	[32.412, 32.416]	[10.799, 10.847]	[9.620, 9.686]
Marginal Rates of Substitution (MRS):				
MRS_ECI/dist_	−0.116	−0.050	−0.193	−0.401
95% CI	[−0.116, −0.116]	[−0.050, −0.050]	[−0.194, −0.192]	[−0.402, −0.400]
${{\rm{MRS}}}_{\inf /{\rm{dist}}}$	0.692	0.906	−0.468	−0.259
95% CI	[0.690, 0.694]	[0.905, 0.909]	[−0.469, −0.468]	[−0.260, −0.259]
${{\rm{MRS}}}_{\inf /{\rm{ECI}}}$	−5.985	−18.056	2.429	0.646
95% CI	[−6.001, −5.972]	[−18.106, −17.998]	[2.421, 2.434]	[0.644, 0.649]
Elasticities (*E*):				
*E*_dist_	−5.177	−3.238	−3.682	−2.616
95% CI	[−5.186, −5.167]	[−3.243, −3.233]	[−3.691, −3.674]	[−2.641, −2.595]
*E*_ECI_	0.804	0.215	0.477	0.979
95% CI	[0.802, 0.805]	[0.214, 0.215]	[0.476, 0.478]	[0.969, 0.988]
${E}_{\inf }$	−2.305	−1.917	2.222	0.666
95% CI	[−2.312, −2.296]	[−1.924, −1.911]	[2.219, 2.226]	[0.659, 0.673]

The estimated distance threshold *τ* marks the approximate distance at which behavior shifts from the proximity-first to the opportunity-first regime. In the U.S. cities, *τ* is strikingly small, suggesting that ECI^emp^ and informality considerations become relevant almost immediately as distance increases. This likely reflects a combination of socioeconomic conditions and urban shape. In the U.S., better highway infrastructure and widespread access to private vehicles make longer commutes more feasible, while at the same time, there’s a preference for single-family housing, often located far from dense work centers. In contrast, *τ* is substantially larger for Mexico City (16.201 km) and Rio de Janeiro (24.955 km), indicating a longer proximity-first regime before ECI^emp^ and informality weigh in. The steepness parameter *k* is higher for U.S. cities, especially the Bay Area (128.764), implying a very sharp transition, while it is more gradual for the Latin American cities.

### Marginal rates of substitution and elasticities

The estimated coefficients allow us to derive interpretable metrics such as substitution rates and elasticities, which quantify how commuting flows vary with different work attributes.

Marginal Rates of Substitution (MRS) are calculated as the ratio of the marginal utilities between variables (e.g., (∂*V*_*ij*_/∂*X*_1_)/(∂*V*_*ij*_/∂*X*_2_)), and they give the marginal rate at which individuals are willing to trade one attribute for another while maintaining the same level of utility. The actual derivation of the MRS for our model can be found in the Methods. For instance, the MRS_ECI/distance_ indicates how much the negative effect of an additional unit of distance can be offset by an increase in ECI^emp^. A negative value for this ratio, such as −0.193 for Mexico City shown in Table 2, depicts a positive trade-off: given that *β*_dist_ is negative and *β*_ECI_ is positive, this MRS means that for every unit increase Δ in ECI^emp^, individuals are willing to tolerate an increase in distance equivalent to 0.193 × Δ to keep the same level of utility. This suggests that workers are willing to travel farther for jobs in locations with higher ECI^emp^. Our results show Rio de Janeiro exhibiting the highest willingness to trade distance for ECI^emp^, while Los Angeles displays the lowest among the studied cities.

The interpretation of the MRS_informality/distance_ is shaped by the different signs of ${\beta }_{\inf }$ across regions. For U.S. cities, the positive MRS (0.692 for the Bay Area; 0.906 for Los Angeles), combined with a negative *β*_dist_ and a negative ${\beta }_{\inf }$, imply that residing in an area with higher informality amplifies the negative effect of distance when considering distant jobs. In contrast, for Latin American cities, the negative ratios (−0.468 for Mexico City; −0.259 for Rio de Janeiro), coupled with a positive ${\beta }_{\inf }$, suggest that higher informality in the origin somewhat mitigates the utility cost associated with traveling longer distances to pursue jobs in high-ECI^emp^ locations.

While substitution rates describe trade-offs in utility terms, it is also useful to understand how a change in an attribute directly affects the probability of a specific work location being chosen. For this, we use elasticities (*E*). An elasticity measures the percentage change in the probability of choosing a particular alternative in response to a 1% change in one of its attributes, holding all else constant (Table 2). In simpler terms, it tells us how sensitive our choice probabilities are to small changes in factors like distance, ECI^emp^, or origin informality. A key property of elasticities in multinomial logit models is that a change in an attribute of one alternative affects not only its own choice probability, but also the probabilities of all other alternatives in the choice set, as the sum of probabilities must always equal one. The direct elasticity of the probability of choosing an alternative *j* from origin *i* (*P*_*ij*_) with respect to an attribute *X*_*ij*_ (an attribute *X* specific to the alternative *j* for an individual from *i*) is given by ${E}_{{X}_{ij}}^{{P}_{ij}}=(\partial {V}_{ij}/\partial {X}_{ij}){X}_{ij}(1-{P}_{ij})$.

Across all cities, commuting distance exhibits the largest (negative) elasticity. For example, in Mexico City, *E*_dist_ = −3.682. This means that for a worker from a given origin *i*, if one potential work location *j* is 1% farther away than an otherwise identical work location $j{\prime}$, the probability of choosing *j* is approximately 3.682% lower than choosing $j{\prime}$. The ECI^emp^ term generally shows a smaller, positive elasticity, indicating that choice probabilities increase with higher ECI^emp^, but at a proportionally lower rate than the change in the ECI^emp^ term itself. The elasticity related to origin informality varies considerably with a negative sign for U.S. cities and positive for Latin American cities. This highlights that the socio-demographic composition of a worker’s home location, through its interaction with perceived remoteness, has a substantial and directionally distinct impact on average choice probabilities. Because ECI^emp^ and informality enter the utility through the transition weight, their elasticities are those of the interacted terms ${w}_{ij}{{\rm{ECI}}}_{j}^{{\rm{emp}}}$ and ${w}_{ij}{{\rm{Inf}}}_{i}$ (see “Methods,” Equation (6)).

### Flow estimation and model performance

A key application of our utility-based discrete choice model, which estimates the probability that an individual of a given origin will select any particular location for work, is its ability to predict aggregate commute flows between locations. This predictive power is valuable not only for validating the model’s behavioral assumptions but also for estimating trips in contexts where empirical data may be incomplete or unavailable. To evaluate this predictive capacity, we compare the commute flows generated by our WorkReach model with the observed flows. Furthermore, we benchmark its performance against three established spatial interaction models: the gravity model with a power-law distance decay function, the extended version of the radiation model^5^, and a recently proposed Bayesian Machine Scientist (BMS) gravity-like model in its plausible version^13^.

To quantify this performance, we used two standard metrics. The first is the Pearson correlation coefficient (*r*), which assesses the linear association between the predicted and observed flows. The second metric, which is widely used in mobility studies, is the Common Part of Commuters (CPC)^7,43^. The CPC measures the degree of overlap between the observed and predicted flow distributions across all origin-destination pairs. Then it can be interpreted as the fraction of commuters whose trips are correctly allocated by the model to the observed destinations, a mathematical description is described in the Methods. Similarly to *r*, the CPC is a number between 0 and 1, 1 being a perfect match, while a value 0 means that there is no overlap between the predicted and observed flows.

**Fig. 4:**
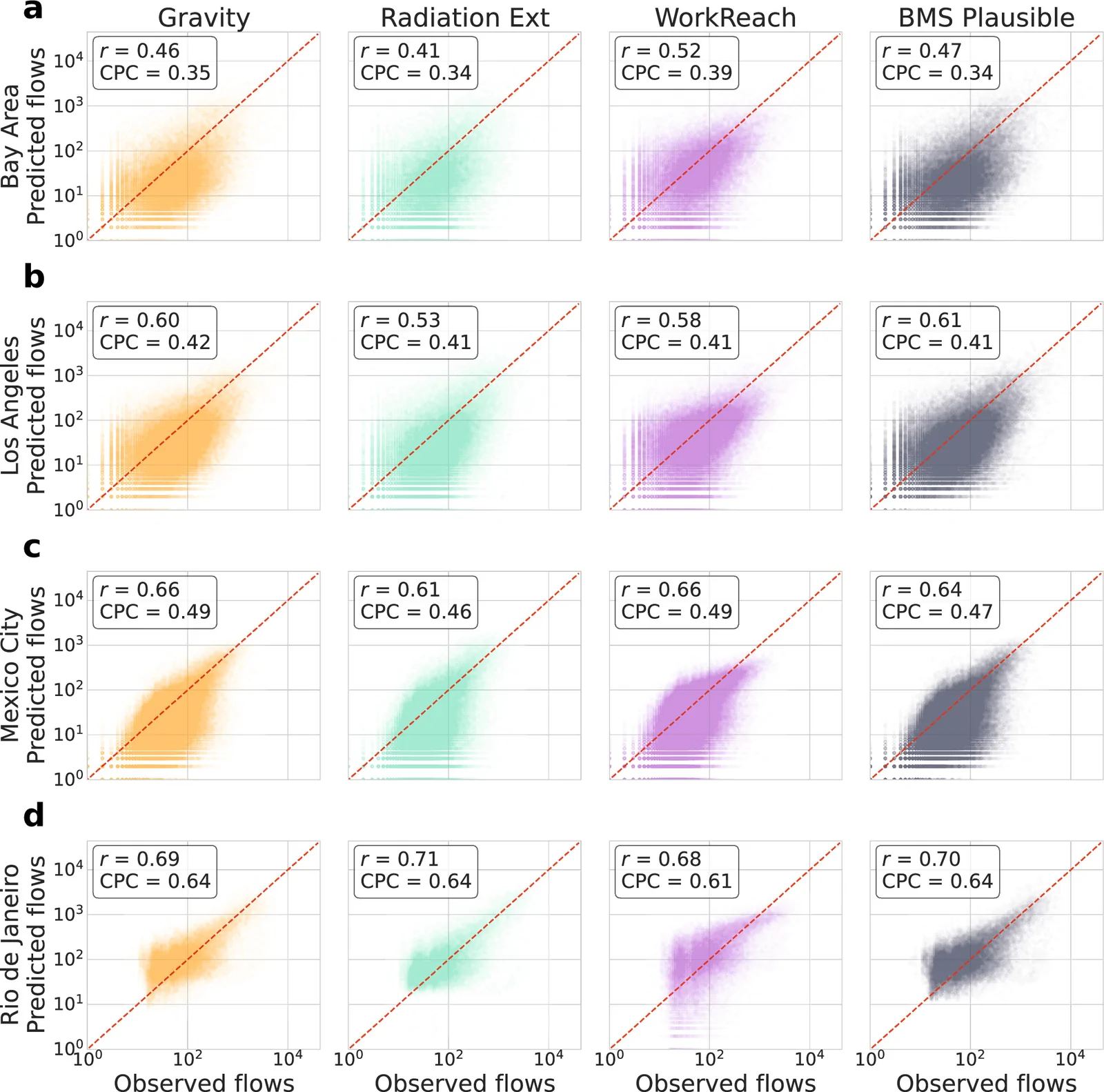
Scatterplot comparison of model performance. Observed flows (x-axis) versus predicted flows (y-axis) for four models across the four study cities, on a log-log scale. The models are: Gravity (power-law distance decay), Radiation Extended, the WorkReach model, and the Bayesian Machine Scientist (BMS) Plausible model. Values for Pearson Correlation Coefficient (*r*) and Common Part of Commuters (CPC) are displayed for each city and model. Rows are the cities, **a** Bay Area, **b** Los Angeles, **c** Mexico City and **d** Rio de Janeiro, and columns are the models, from left to right Gravity, Radiation Extended, WorkReach and BMS Plausible. Each panel contains every origin-destination pair retained for the city, *n* = 457,652 (Bay Area), 365,420 (Los Angeles), 628,056 (Mexico City) and 39,402 (Rio de Janeiro) pairs (Table 1). The WorkReach model attains predictive accuracy comparable to established benchmark models across all cities.

Our WorkReach model achieves CPC values that are comparable with those of the benchmark models (Fig. 4), and in some cases as for the Bay Area, our model yields the highest CPC (0.389). In Los Angeles and Mexico City, the CPC values are closely aligned with the top-performing gravity models. For Rio de Janeiro, the CPC (0.614) is slightly lower than the other alternatives but still yields a high value. The Pearson correlations have a similar pattern, with our WorkReach model consistently achieving high correlation coefficients. These findings suggest that our behaviorally-grounded model, which uniquely incorporates ECI^emp^ and informality through an interactive utility structure, can predict commuting flows with an accuracy comparable to established spatial interaction models (Supplementary Figs. 16–19). The primary advantage of our approach, therefore, lies not solely in its predictive accuracy but in the enhanced interpretability and deeper understanding it offers regarding the socioeconomic drivers shaping commuting choices. A stratified 5-fold cross-validation on origin zones indicates that these results generalize out of sample: the train–test performance gap is negligible for all models (ΔCPC ≤ 0.001; Supplementary Note 9).

Beyond overall fit, we audited WorkReach’s predictive fairness by computing the CPC across ECI^emp^ and informality rate bins (Supplementary Figs. 20 and 21). While CPC remains relatively high across most strata, we observe a drop in the highest informality bins, especially in the Bay Area and Mexico City. This drop may reflect the fact that WorkReach estimates flows at the population level without group-specific optimization, unlike fairness-aware models such as FairMobi-Net^44^, which incorporate a fairness-loss term to explicitly address such disparities. However, this audit identifies where future Fairness Enhancement of WorkReach could be most impactful.

To validate our use of economic complexity, we tested it against a more traditional economic property, the local economic status of a destination. A consistent wage measure is not available at the spatial resolution we require, so for the two U.S. cities we use individual wage records, while for Mexico City and Rio de Janeiro, where fine-grained wage data are lacking, we build income and wealth proxies from census data and aggregate them to work locations through the commuting flows (Supplementary Note 15). Replacing economic complexity with this proxy brings no clear or consistent gain, and the apparent edge of the proxy in the Latin American cities should be read with caution, since it is built from the flows themselves. The economic complexity framework also lets us measure sectoral relatedness, the overlap in sector composition between a worker’s home zone and a potential destination (Supplementary Note 16). Substituting relatedness for ECI^emp^ in the utility slightly improves the CPC, yielding 0.391 (Bay Area), 0.425 (Los Angeles), 0.495 (Mexico City), and 0.652 (Rio de Janeiro) against WorkReach’s 0.389, 0.413, 0.486, and 0.614, while a model with both reaches 0.399, 0.428, 0.497, and 0.655 (Supplementary Table 13). Adding either to a model containing the other still improves fit significantly (Supplementary Table 15), so the two co-exist rather than substitute for one another. Relatedness enters with a negative coefficient in every city, so commuters tend to travel to zones offering structurally different employment from what is locally available, consistent with a spatial mismatch interpretation. Its inclusion also changes the role of informality, turning ${\beta }_{\inf }$ negative in all four cities and reversing its sign in Mexico City (10.250 to −4.620) and Rio de Janeiro (4.267 to −1.133) (Supplementary Table 14). Our framework quantifies the trade-offs these properties imply and how they interact, yet we retain economic complexity as the model’s opportunity measure because, unlike the alternatives, its value for a destination is invariant to the origin and it is actionable as a lever for urban policy, since pairing it with the opportunity score^17,45^ lets us simulate how targeted economic development would reshape commuting accessibility (Supplementary Note 19).

### Accessibility

**Fig. 5:**
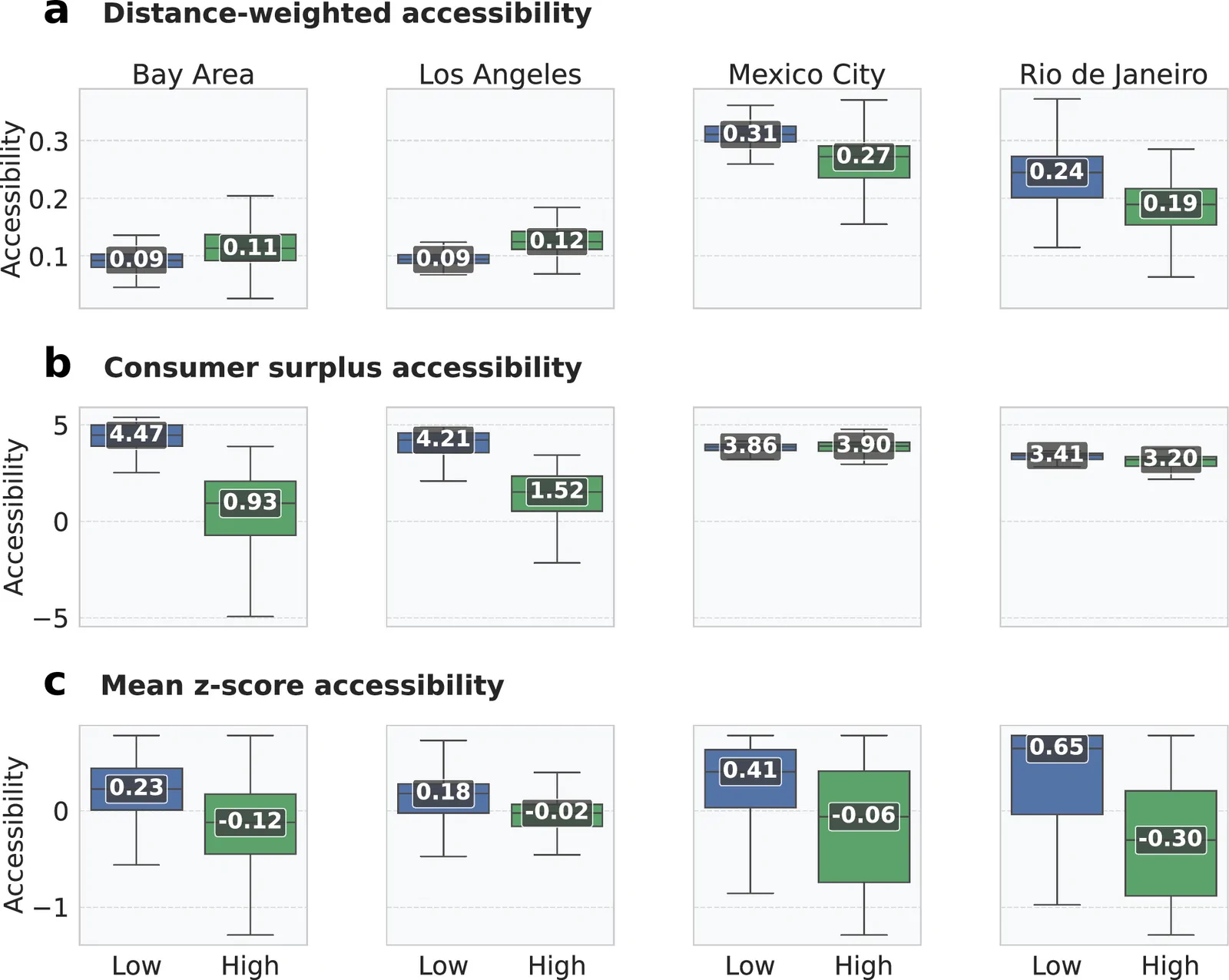
Accessibility measures by origin informality group. Origins are classified as low or high informality based on whether their informality rate falls below or above the city-specific median. The unit of analysis is the origin zone, with *n* = 339 low and 338 high informality zones for the Bay Area, 303 and 302 for Los Angeles, 397 and 396 for Mexico City, and 100 and 99 for Rio de Janeiro. Boxes span the 25th–75th percentiles with the median as the center line, and whiskers extend to the most extreme values within 1.5 times the interquartile range; outliers are not shown. Numbers indicate median values. **a** Distance-weighted accessibility boxplots showing higher median values for high-informality areas in U.S. cities but lower values (distance penalty) in Latin American cities. **b** Consumer-surplus accessibility (expected maximum utility) lower for high-informality areas in three of four cities; Mexico City is the exception, where the positive ${\beta }_{\inf }$ partially offsets distance penalties. **c** Combined accessibility index computed as the mean of the *z*-scored distance-weighted and consumer-surplus measures. High-informality origins have lower combined accessibility in all four cities.

Our calibrated discrete choice model allows us to compute accessibility measures that go beyond a physical proximity measure by incorporating the trade-offs and preferences revealed by commuter behavior. We use two such measures. Distance-weighted accessibility (${A}_{i}^{{\rm{DW}}}$) averages the inverse distance to every destination, weighting each by the predicted flow towards it, so it rewards origins whose realized commutes are short. Consumer-surplus accessibility (${A}_{i}^{{\rm{CS}}}$), also known as logsum accessibility^34,35^, is defined in terms of random utility theory as the expected maximum utility of the whole choice set at an origin, so it measures how attractive the set of work choices is from that origin rather than how close those choices are. We define both measures in full in the “Methods.” Figure 5 compares two accessibility metrics across locations by informality rate (above or below the city median). In Fig. 5a, U.S. cities show higher median distance-weighted accessibility for high-informality neighborhoods. In contrast, in Mexico City and Rio de Janeiro, low-informality areas enjoy greater distance-weighted accessibility. This pattern reflects the peripheralization of informal settlements in Latin American metros, which forces residents to live farther from employment centers^2,33^. Fig. 5b shows consumer-surplus accessibility, which measures the net benefit of available work choices by incorporating distance, destination ECI^emp^, and origin informality. Here, median consumer-surplus accessibility is lower for high-informality origins in every city except Mexico City, where the two medians nearly coincide. In the U.S. cities, this reverses the distance advantage observed in Fig. 5a: once work quality and informality effects enter utility, high-informality neighborhoods lose their apparent edge. In Mexico City and Rio de Janeiro, the positive informality coefficient (${\beta }_{\inf } > 0$) partially offsets raw distance penalties. In other words, this benefit generally does not compensate for longer distances and typically lower ECI^emp^ levels of accessible jobs.

These results highlight the value of utility-based accessibility in urban analysis, but no single metric fits every purpose: relying solely on distance-weighted measures can mask quality differences, while using only consumer-surplus might understate disparities faced by informal workers who must accept distant, low-quality jobs for lack of alternatives. A combined view delivers a more behaviorally grounded assessment, revealing how uneven spatial distributions of homes and jobs create socioeconomic inequities in urban labor markets.

**Fig. 6:**
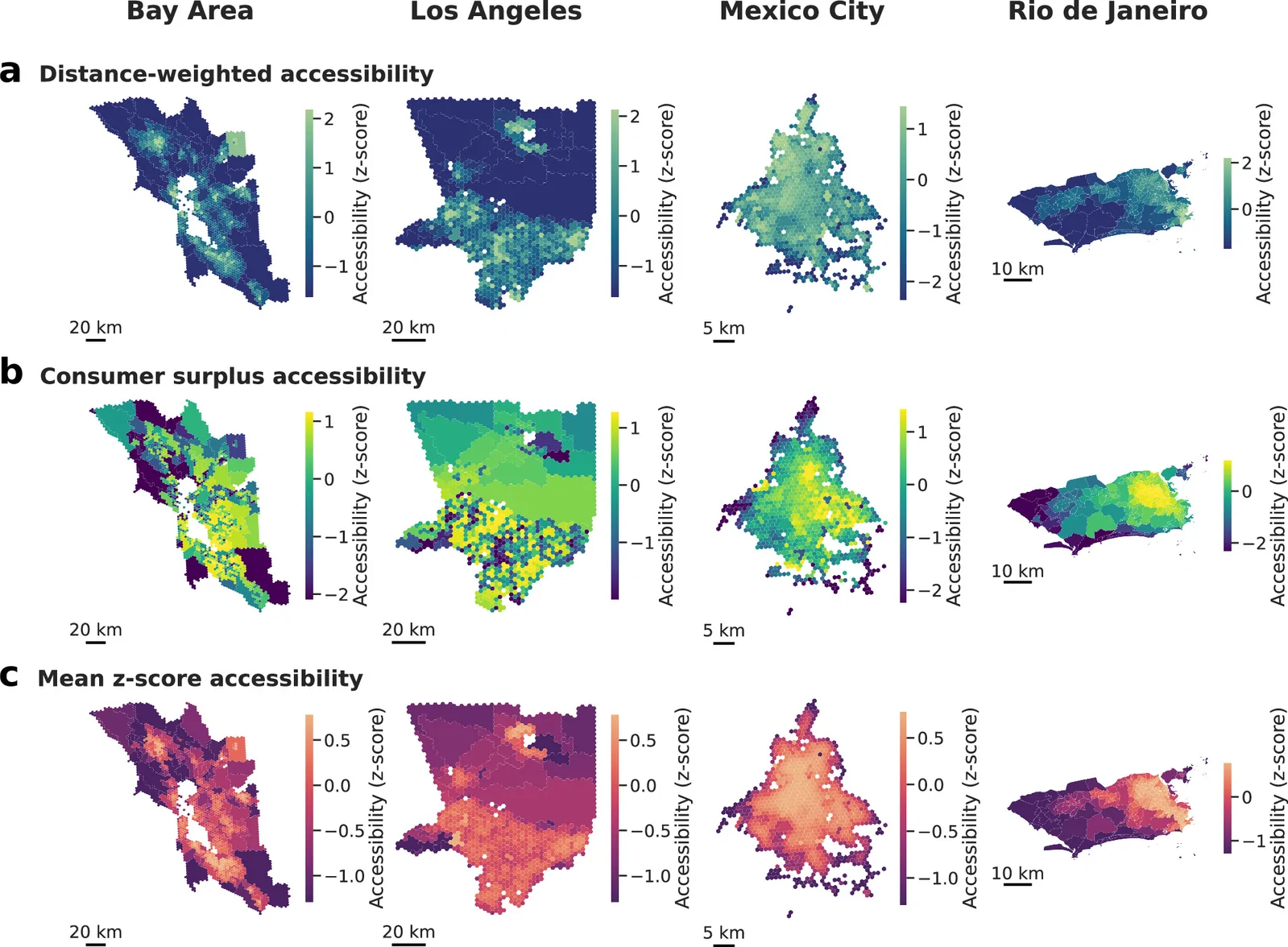
Spatial distribution of accessibility measures (z-scores). **a** Distance-weighted accessibility z-scores standardized by city for cross-regional comparison. **b** Consumer-surplus accessibility z-scores, with discrepancies from (**a**) highlighting impacts of work quality (economic complexity, ECI^emp^) and origin informality beyond distance. **c** Combined accessibility index (mean of *z*-scored distance-weighted and consumer-surplus measures), representing the overall accessibility level. Spatial units are H3 level 7 hexagons for the Bay Area and Los Angeles, H3 level 8 hexagons for Mexico City, and Área de Estatística e Planeamento (AEP) polygons for Rio de Janeiro. Where a single census geometry intersects several hexagons, those hexagons are merged into one unit that covers it. Accessibility is *z*-scored within each city. Each map shows the full study area, which is the city proper for Los Angeles, Mexico City and Rio de Janeiro and the multi-county San Francisco Bay Area region described in “Methods”.

Because only relative utilities matter (absolute levels can even be negative), we standardize both metrics using within-city *z*-scores and compute a combined index as their mean (Fig. 5c). This combined measure captures the overall accessibility level by averaging the contributions of physical proximity and utility-based attractiveness, so that areas scoring low on both dimensions are identified as the most underserved. Figure 6 maps these *z*-scores and their combined index. Across the four cities, peripheral locations, especially the western zones of Rio de Janeiro, emerge as the most underserved.

Correlation analysis between informality rates and accessibility measures shows regional differences (Supplementary Fig. 34). For distance-weighted accessibility, correlations are positive in U.S. cities, meaning that higher informality areas have better distance-based accessibility, while this is negative in Latin American cities, reflecting the peripheralization of higher informal rates away from complex work hubs. Consumer-surplus accessibility, on the other hand, shows negative correlation with informality in all cities but Mexico City, which suggests that the perceived advantage of pursuing high complexity jobs increasingly outweighs the distance penalty for workers in more informal areas. The combined accessibility measure (mean *z*-score) shows decreasing accessibility with increasing informality in all cities, confirming that high-informality origins face systematic accessibility disadvantages when both dimensions are considered jointly.

## Discussion

Understanding commuter behavior is essential for developing urban policies that reduce socioeconomic inequality. Incorporating economic complexity (ECI^emp^) and informality into an interpretable model based on discrete choice theory illustrates how these factors, alongside distance, shape home-work choices in ways that differ across cities. This broadens the concept of accessibility from a purely spatial measure to one that also reflects perceived economic benefit.

Our results indicate that higher-complexity workplaces are consistently associated with longer commuting distances across the four cities studied, while commuter demographics, represented by the informality rate in our model, vary in behavior across urban contexts. Workers from high-informality neighborhoods in Mexico City and Rio de Janeiro travel farther to reach economically complex job centers, overcoming geographic and socioeconomic barriers. In contrast, in the Bay Area and Los Angeles, workers from low-informality areas are more likely to travel greater distances, implying that informality in these contexts is more related to social or institutional constraints than to spatial accessibility challenges. WorkReach captures these context-specific dynamics while matching the predictive performance of established parsimonious models, yet gains interpretability by deriving utility coefficients, MRS, and elasticities. Moreover, a critical insight from our accessibility analysis is that physical proximity to employment and meaningful access to quality opportunities are fundamentally different. In cases where distance-weighted accessibility presents advantages for high-informality populations, consumer-surplus accessibility follows the opposite pattern. This suggests that simply being near employment centers does not guarantee access to jobs that maximize worker utility. This distinction highlights that socioeconomic background and job quality matter just as much as physical proximity in determining access to meaningful economic opportunities, revealing disparities that conventional distance-based metrics overlook.

ECI^emp^ and residential informality are empirically distinct: their zone-level correlations are near-zero in U.S. cities and moderate in Latin American cities (*r* ≈ −0.40), confirming that each captures a different spatial dimension (Supplementary Note 11). The positive ${\beta }_{\inf }$ in Latin American cities is consistent with the spatial structure of these metropolitan areas, where high-informality residential zones tend to be peripheral and offer fewer local employment options. In addition, the employment data used to construct ECI^emp^ in these cities records formally registered establishments, so working in a high-ECI^emp^ zone tends to carry social security affiliation and statutory benefits even for non-specialized roles, providing an additional incentive for workers from high-informality areas to overcome geographic barriers. Because the WorkReach utility readily accommodates additional covariates, relatedness can replace or complement ECI^emp^ in our formulation. In Mexico City and Rio de Janeiro, relatedness declines with distance for high-informality origins but not for low-informality ones (Supplementary Fig. 33), so the longer commutes from high-informality zones are consistent with a mismatch cost alongside the complexity attraction. Complexity and relatedness describe co-existing influences on work location choices, the first a property of the destination and the second of the home–work pair.

Together, the interpretable parameters and the accessibility metric allow WorkReach to generate policy-relevant insights that other models cannot provide, and illustrate the importance of customizing interventions to local contexts. In Latin American cities, improving connectivity between peripheral, high-informality neighborhoods and central high-ECI^emp^ areas may help expand economic opportunities. However, these measures need to be paired with strategies to incentivize local employment, especially formal jobs, and improve local economic complexity to avoid merely shifting burdens onto workers by making them commute long distances. In contrast, in U.S. cities, more effective interventions might be those focused on targeted skill development and reducing discriminatory barriers, as there the physical distance barrier is lower for workers in the informal sector. Our results can directly inform infrastructure investment, as areas with high ECI^emp^ but limited accessibility for disadvantaged groups represent good candidates for the improvement of public transportation. In contrast, regions characterized by high physical accessibility and low ECI^emp^ offer good opportunities for focused economic development programs.

Our analysis has several limitations. The estimated coefficients reflect systematic associations in cross-sectional commuting flows, not experimentally identified preferences; quasi-experimental or longitudinal designs would be needed to establish causal links between changes in ECI^emp^ and shifts in commuting behavior. Relatedly, our framework treats residence as fixed and models only workplace choice, a standard assumption supported by the higher frequency of job transitions relative to residential moves in both the United States^46^ and Latin America^47^, but one that does not capture the joint residential-workplace decision. Finally, ECI^emp^ remains an aggregate index: decomposing which agglomeration channels most strongly attract commuters is an important direction for future work. Data resolution limitations and inconsistencies in measuring informality across regions are difficult to overcome. Harmonizing the underlying data across regions, both the informality definitions and the employment registries from which economic complexity is derived, remains a key area for improvement. Future research should also expand this analysis to additional urban settings, particularly in regions with different economic structures. Longitudinal studies would be beneficial for evaluating the causal effects of particular interventions over time. Furthermore, incorporating additional socioeconomic variables could deepen our understanding of the mechanisms driving urban labor market dynamics.

## Methods

### Conceptual and theoretical background

The study of how individuals choose where to live and work is grounded in economic theories of spatial interaction and individual decision-making. We build upon the random utility maximization (RUM) framework^48^, which assumes that individuals choose the alternative (in this case, a work location) that offers the highest utility from a set of available options. Utility is typically a function of attributes of the alternatives and characteristics of the decision-maker, and is written *U*_*pc*_ = *V*_*pc*_ + *ε*_*pc*_, where *V*_*pc*_ is the systematic utility, the measurable portion of it, and *ε*_*pc*_ is the unobserved part. Our work extends this by explicitly incorporating measures of local economic structure (ECI^emp^) and labor market conditions (informality) into the utility function, alongside traditional factors like travel distance.

### Data and variable construction

#### Informality rate

Given the varying prevalence and nature of informal employment, we need to utilize context-specific methodologies for each specific region, as there is no universal definition that can be obtained or derived directly from available data. For Mexico City, we estimate informality rates using microdata from the 2020 Population and Housing Census from the National Institute of Statistics and Geography (INEGI), aggregated at the urban Basic Geostatistical Area (AGEB) level. We used affiliation to social security services as a proxy for formality, following the legal requirement in Mexico for formal employers to register workers in the Instituto Mexicano del Seguro Social (IMSS). For this, we calculate the proportion of the economically active population without access to medical insurance services within each AGEB as the informality rate, adjusting for insured dependents (minors covered through a family member) who are not themselves part of the workforce. This proxy is validated using Mexico’s National Survey of Occupations and Employment (ENOE, 2022), which tracks formal and informal employment and indicates that 99.95% of formally employed individuals have access to medical insurance, compared to only 11.18% of informal workers.

For Rio de Janeiro, we follow the methodology applied by Moreno-Monroy and Ramos for the city of São Paulo^2^, using census data from 2010, which is the latest available at the Área de Estatística e Planeamento (AEP) scale, which is the highest resolution publicly available. Workers are classified as informal if they are employees without formal registration (*empregado sem carteira assinada*), or self-employed individuals who do not contribute to social security.

For Los Angeles and the Bay Area, measuring informality is more challenging due to its low prevalence and low government tracking. We adapt a method used by the U.S. Census Bureau staff in a study by Graham and Ong^3^, which compares workforce numbers from the American Community Survey (ACS 5-year estimates) with employment records from the Longitudinal Employer-Household Dynamics (LEHD) dataset. Workers present in ACS data but absent from LEHD records are considered informal as the latter only track individuals in payroll with unemployment insurance contributions, effectively tracking formal employment. This approach was originally developed using the Census Long Form, which is no longer available but has been replaced by the ACS. This is done at the Census Block Group level.

#### Economic complexity index (ECI^emp^)

ECI^emp^ is computed at the H3 hexagonal zone level by assigning each individual job record to its containing hexagon based on the establishment’s geographic coordinates. This differs from the informality rate, which is measured at the census-tract level (Census Block Groups in the U.S., AGEBs in Mexico City, AEPs in Rio de Janeiro) and then area-weighted to H3 zones; for Rio de Janeiro, where AEP polygons are already coarser than H3 cells, we retain the original boundaries for both variables. Computing ECI^emp^ directly at the H3 level, rather than aggregating from administrative units, provides a consistent spatial resolution across cities and enables the product-space analyses reported in the SI, including sectoral relatedness (Supplementary Note 16), diversity and ubiquity decompositions (Supplementary Note 14), and counterfactual ECI^emp^ uplift scenarios (Supplementary Note 19). For this, we use the number of employed individuals at each location in any of the 17 industry sectors from the 2-digit NAICS, excluding Agriculture and Other Services to retain sectors with clear interpretability in the product-space framework. These sectors are treated as the “products” in Economic Complexity terms^16,17^, and their associated Product Complexity Index (PCI^emp^) reflects the complexity of each work sector. We confirm that the zone–sector matrices underlying the ECI^emp^ computation are nested in all four cities (Supplementary Note 12), a property that supports the reliability of the complexity rankings^49^. For Mexico City, employment data come from INEGI’s Directory of Economic Units (DENUE), which provides geocoded establishment records that are spatially joined to H3 hexagons. DENUE reports the number of employees per establishment in categorical size ranges rather than exact counts, so we impute a point estimate by drawing a uniform random integer within each range, capping the open-ended upper category at 500 employees. For Rio de Janeiro, employment records come from Brazil’s Annual Social Information Report (RAIS), which includes codes from the National Classification of Economic Activities (CNAE) and are manually harmonized with NAICS. As RAIS reports employment in a similar categorical format as DENUE, we apply the same imputation procedure. For Los Angeles and the Bay Area, we use synthetic employment data from Replica, which integrates demographic, economic, and transportation data including NAICS sector. Replica provides individual-level records with geographic coordinates, which we spatially join to H3 hexagons via an area-weighted overlay from the Census Block Group level.

#### Data

In order to understand the connection between home and work location choices we use commuting flows derived from different data sources. For Mexico City and Rio de Janeiro, we used anonymized Location-Based Services (LBS) data from a single commercial provider, Quadrant, covering March-May 2023 and March-May 2024 (six months total), the period for which data are available. We process raw ping data to estimate frequent visitations to specific locations and used a heuristic method based on circadian rhythms to assign a home and workplace location of each individual user, as described below. Because we infer only home and work locations rather than modeling the full set of trips, and because inference requires a minimum of 14 and 8 distinct days of observations respectively (see below), the resulting commuting patterns reflect habitual behavior and are robust to short-term seasonal variation. We retain only users whose inferred home and work locations are consistent across both years. For Los Angeles and the San Francisco Bay Area, Replica’s synthetic mobility dataset provides average weekday commuting patterns. Multiple years and seasons are available, and we used the generated data for Spring 2023. Across all four cities, once home and workplace locations are identified, they are spatially merged with their respective geostatistical units to assign origin-level informality metrics and destination-level ECI^emp^.

To build comparable flow networks across cities, we remap the original geographic units to Uber’s H3 hexagonal grid system, which offers a consistent spatial resolution. When administrative or census polygons are smaller than the target H3 cells, we aggregate them using area-weighted averages for ECI^emp^ and informality rates, based on their overlap with each hexagon. In contrast, when original zones are larger and encompass multiple H3 cells, we preserve their scale by taking the union of the fully contained hexagons and treating them as a single unit. For Rio de Janeiro, where the original AEP polygons are already coarse, we retain the original boundaries without remapping.

### Location inference from LBS data

Raw LBS pings, each with latitude, longitude and timestamp, are processed in six steps.

We first detect stay events, flagging, for each device, consecutive chains of pings that fall within a radius of 20 meters and span at least 5 minutes in total. We then cluster these events spatially with the Density-Based Spatial Clustering of Applications with Noise (DBSCAN) algorithm^50^ with an *ϵ* = 20 m and a minimum of 2 points, which produces distinct stop location clusters. Because the start times are recorded in UTC, we spatially join each cluster centroid to a timezone lookup table and convert them to local datetimes using the matched timezone. We then label home and work locations for each user and year. We label a cluster *home* if it is the most visited one during home hours, meaning weekends or weekdays between 19:00 and 08:00, on at least 14 distinct days, and *work* if it is the most visited on weekdays between 09:00 and 18:00 on at least 8 distinct days and lies at least 50 meters away from the home cluster.

We spatially join the derived home and work locations to the city administrative polygons, AGEBs for Mexico City and AEPs for Rio de Janeiro, to generate aggregated origin-destination matrices. To improve the representativeness and scale of the actual flows, we then compute a scaling factor as the ratio of observed home locations to census population within each administrative unit. This accounts for differences in penetration rates of the mobility data and adjusts the flows to better represent the actual population.

### Construction of the complexity indices

Economic Complexity provides a quantitative framework to analyze the productive structure of geographic regions by assessing the diversity and sophistication of their economic activities^16^. Central to this approach are two interrelated metrics: the Economic Complexity Index (ECI^emp^) for zones (our sub-city zones), and the Product Complexity Index (PCI^emp^) for economic activities (here, NAICS industry sectors, treated as “products” in line with the ECI literature^17^).

The calculation begins by building a binary matrix *M*_*zs*_ indicating whether a zone *z* is specialized in sector *s*. Specialization is determined using the revealed comparative advantage (RCA):

1 $${{\rm{RCA}}}_{zs}=\dfrac{{W}_{zs}/{\sum }_{s^{\prime} }{W}_{zs^{\prime} }}{{\sum }_{z^{\prime} }{W}_{z^{\prime} s}/{\sum }_{z^{\prime},s^{\prime} }{W}_{z^{\prime} s^{\prime} }}\,,$$

where *W*_*zs*_ is the employment in sector *s* within zone *z*. If RCA_*zs *_≥ 1, then *M*_*zs*_ = 1, indicating specialization; otherwise, *M*_*zs*_ = 0. This would mean that the proportion of employed persons in sector *s* in zone *z* to all of the employed individuals in that given zone is larger than the same proportion for the whole city.

2 $${\tilde{K}}_{z}^{0}=\sum _{s}{M}_{zs}$$

3 $${\tilde{K}}_{s}^{0}=\sum _{z}{M}_{zs}$$

4 $${{\rm{ECI}}}_{z}^{{\rm{employment}},\,(N)}={K}_{z}^{N}=\dfrac{1}{{\tilde{K}}_{z}^{0}}\sum _{s}{M}_{zs}{K}_{s}^{N-1}$$

5 $${{\rm{PCI}}}_{s}^{{\rm{employment}},\,(N)}={K}_{s}^{N}=\dfrac{1}{{\tilde{K}}_{s}^{0}}\sum _{z}{M}_{zs}{K}_{z}^{N-1}\,.$$

This iterative process (the method of reflections) starts with ${\tilde{K}}_{z}^{0}={\sum }_{s}{M}_{zs}$ (diversity of zone *z*) and ${\tilde{K}}_{s}^{0}={\sum }_{z}{M}_{zs}$ (ubiquity of sector *s*). The iterations (*N* > 0) refine these initial measures by averaging the complexity of the connected nodes in the bipartite network. The ECI^emp^ is typically taken as the second eigenvector of a matrix derived from *M*_*zs*_, which this iterative process converges to. Formally, this eigenvector corresponds to the largest non-trivial eigenvalue of the projection of the bipartite zone-sector network onto the zone space^16^. Its entries partition zones into two groups according to their connectivity patterns in this projected network, analogous to a spectral bisection of the graph: zones with similar entries share denser connections to zones with similar sector structure. Because the eigenvector is defined only up to a sign, its direction is fixed following the convention of^16^ by choosing the orientation in which the eigenvector entries correlate positively with diversity. The resulting ECI^emp^ values are standardized for use in the WorkReach model. We use employment data from DENUE (Mexico City), RAIS (Rio de Janeiro), and Replica (U.S. cities), harmonized to NAICS sectors, aggregated to H3 hexagonal zones for consistent ECI^emp^ calculation across cities. All calculations of the Economic Complexity framework were done via the econci Python package^51^.

### Discrete choice model specification

The work location choice process is modeled using a multinomial logit (MNL) framework. The utility *V*_*ij*_ that a worker residing in origin zone *i* derives from choosing a workplace in destination zone *j* is

6 $${V}_{ij}={\beta }_{{\rm{dist}}}{d}_{ij}+{w}_{ij}(\tau,k)\cdot \left({\beta }_{{\rm{ECI}}}{{\rm{ECI}}}_{j}^{{\rm{emp}}}+{\beta }_{\inf }{{\rm{Inf}}}_{i}\right)\,,$$

where *d*_*ij*_ is the min-max standardized Haversine distance between *i* and *j*. ${{\rm{ECI}}}_{j}^{{\rm{emp}}}$ is the min-max standardized Economic Complexity Index of destination *j*. ${{\rm{Inf}}}_{i}$ is the min-max standardized informality rate at origin *i*. All three variables are min-max scaled to [0, 1] directly, placing them on a comparable scale and improving the efficiency and convergence of the estimation procedure. The distributions of the three inputs before and after scaling are given in Supplementary Note 3 and Supplementary Figs. 9–12.

The transition weight *w*_*ij*_(*τ*, *k*) is a logistic function of the standardized distance,

7 $${w}_{ij}(\tau,k)=\dfrac{1}{1+\exp (-k({d}_{ij}-\tau ))}\,,$$

where *τ* is the distance around which work selection shifts from a local to an economic criterion and *k* controls how sharply that shift occurs; both are estimated alongside the three *β* coefficients. For short distances (*d*_*ij*_ ≪ *τ*), *w*_*ij*_ ≈ 0 and utility is driven by minimizing distance, whereas for long distances (*d*_*ij*_ ≫ *τ*), *w*_*ij*_ ≈ 1 and destination ECI^emp^ and origin informality exert their full influence. The calibrated shape of this function in each city is shown in Supplementary Fig. 15. Crucially, attributes of the decision-maker (or their origin, like ${{\rm{Inf}}}_{i}$) that do not vary over alternatives can only enter the WorkReach model if they are specified in ways that create differences in utility over alternatives^15^. The interaction term ${w}_{ij}(\tau,k)\cdot {\beta }_{\inf }{{\rm{Inf}}}_{i}$ achieves this: because *w*_*ij*_ is a function of *d*_*ij*_ (an alternative-specific attribute, i.e., distance to work *j*), the impact of origin informality ${{\rm{Inf}}}_{i}$ on utility now varies across the different work alternatives *j* available from origin *i*. This makes the socio-demographic variable ${{\rm{Inf}}}_{i}$ identifiable and interpretable within the choice model framework.

The probability *P*_*ij*_ of choosing *j* from *i* is given by the standard MNL formula,

8 $${P}_{ij}=\dfrac{\exp ({V}_{ij})}{{\sum }_{j{\prime} \in {J}_{i}}\exp ({V}_{ij^{\prime} })}\,,$$

where *J*_*i*_ is the set of work locations available from origin *i*. The model parameters $\theta=({\beta }_{{\rm{dist}}},{\beta }_{{\rm{ECI}}},{\beta }_{\inf },\tau,k)$ are estimated by maximum likelihood, as described in Parameter Estimation and Optimization. Numerical stability is ensured by subtracting the maximum utility within each choice set before exponentiation. Prior to estimation, we discard origin-destination pairs with fewer than five observed flows and origins with fewer than ten total outgoing flows.

In order to obtain the model’s commuter flows, we multiply the probability of observing a flow going from *i* to *j* to the total population from the origin and round the result to the nearest integer:

9 $${T}_{ij}=\,{\mathrm{round}}\,\left({m}_{i}\cdot {P}_{ij}\right).$$

### Iso-utility curves, substitution rates, and elasticities

WorkReach stands out in terms of interpretability and as a discrete choice based model allows for the quantification of trade-offs between work attributes. This framework enables the derivation of key behavioral measures that indicate how workers value and substitute between different work characteristics.

Iso-utility curves represent the combination of attribute levels that maintain a same level of systematic utility V^*^. In general, given two attributes *X* and *Y* with coefficients *β*_*X*_ and *β*_*Y*_, the iso-utility condition is *β*_*X*_*X* + *β*_*Y*_*Y* = *C*, where *C* is a constant. For example, in the opportunity-first regime where the transition weight *w*_*ij*_(*τ*, *k*) ≈ 1 (i.e., for distant choices where socioeconomic effects are fully active), and holding informality constant, the iso-utility condition for distance and ECI^emp^ approximates ${\beta }_{{\rm{dist}}}{d}_{ij}+{\beta }_{{\rm{ECI}}}{{\rm{ECI}}}_{j}^{{\rm{emp}}}=C$. This equation describes how an increase in distance can be compensated for by a relative increase in ECI^emp^ in order to maintain the same utility level.

We can measure these trade-off more generally using MRS that are the rate at which individuals are willing to exchange one attribute for another while maintaining constant utility. The MRS between any two attributes is defined as the ratio of their marginal utilities: MRS_*X*,*Y*_ = (∂*V*/∂*Y*)/(∂*V*/∂*X*).

For attributes modulated by the transition weight (ECI^emp^ and informality), the marginal utility takes the form:

10 $$\dfrac{\partial {V}_{ij}}{\partial {X}_{j}}={w}_{ij}{\beta }_{X}.$$

For distance, the marginal utility is more complex because distance appears both directly in the utility function and indirectly through the transition weight *w*_*ij*_. The transition weight ${w}_{ij}(\tau,k)=\frac{1}{1+\exp (-k({d}_{ij}-\tau ))}$, has the derivative with respect to distance:

11 $$\dfrac{\partial {w}_{ij}}{\partial {d}_{ij}}=k\cdot {w}_{ij}(1-{w}_{ij}).$$

Applying the chain rule to the full utility function, the marginal utility of distance becomes:

12 $$\dfrac{\partial {V}_{ij}}{\partial {d}_{ij}}={\beta }_{{\rm{dist}}}+\dfrac{\partial {w}_{ij}}{\partial {d}_{ij}}\left({\beta }_{{\rm{ECI}}}{{\rm{ECI}}}_{j}^{{\rm{emp}}}+{\beta }_{\inf }{{\rm{Inf}}}_{i}\right).$$

This derivative has a direct component (*β*_dist_) and an indirect component that captures how distance modulates the importance of socioeconomic factors. Then, the complete MRS between distance and any modulated attribute *X* takes the form:

13 $${{\rm{MRS}}}_{d,X}=\dfrac{{w}_{ij}{\beta }_{X}}{{\beta }_{{\rm{dist}}}+k\cdot {w}_{ij}\left(1-{w}_{ij}\right)\left({\beta }_{{\rm{ECI}}}{{\rm{ECI}}}_{j}^{{\rm{emp}}}+{\beta }_{\inf }{{\rm{Inf}}}_{i}\right)}.$$

This expression implies that trade-offs are not constant across space but vary with distance due to the transition weight effects. For choices far from the distance threshold *τ* (where *w*_*ij*_ ≈ 1 and ∂*w*_*ij*_/∂*d*_*ij*_ ≈ 0), the MRS simplifies to the traditional ratio *β*_*X*_/*β*_dist_. The same derivation extends to specifications with additional transition-weight-modulated attributes, such as sectoral relatedness, with the general form for an arbitrary number of attributes given in Supplementary Note 16.

Elasticities provide a complementary measure of sensitivity, representing the percentage change in choice probability for a 1% change in an attribute, ceteris paribus. For any attribute *X*, the elasticity of the probability of choosing alternative *j* is given by:

14 $${E}_{X}^{{P}_{ij}}=\dfrac{\partial {V}_{ij}}{\partial X}X\left(1-{P}_{ij}\right)\,,$$

where ∂*V*_*ij*_/∂*X* is the marginal utility of the attribute and *P*_*ij*_ represents the probability of the pair *ij* from the multinomial logit model^15^.

In our model, elasticities are computed differently for each type of attribute.

For the attributes modulated by the transition weight, ECI^emp^ and informality, the marginal utility is *w*_*ij*_*β*_*X*_, so that

15 $${E}_{X}^{{P}_{ij}}={w}_{ij}{\beta }_{X}X\left(1-{P}_{ij}\right).$$

For distance, the elasticity uses the full marginal utility, including both the direct effect and the indirect effect that acts through the transition weight,

16 $${E}_{{d}_{ij}}^{{P}_{ij}}=\left({\beta }_{{\rm{dist}}}+k{w}_{ij}(1-{w}_{ij})({\beta }_{{\rm{ECI}}}{{\rm{ECI}}}_{j}^{{\rm{emp}}}+{\beta }_{\inf }{{\rm{Inf}}}_{i})\right){d}_{ij}(1-{P}_{ij}).$$

The reported elasticities are unweighted means over all origin-destination pairs.

### Benchmark models

For flow generation accuracy we compare our WorkReach model against three established models in their singly constrained versions to meet the outflow, i.e., ${\sum }_{k}{T}_{ik}={T}_{i}^{{\rm{tot}}}$. All of the models were directly implemented in Python using the SciPy library for parameter optimization^52^. The gravity model has four parameters. The estimated flow *T*_*ij*_ from origin *i* (with population *m*_*i*_ and total observed outflow ${T}_{i}^{{\rm{obs}}}$) to destination *j* (with jobs *m*_*j*_) is modeled using a singly-constrained (production-constrained) formulation with a power-law distance decay. Formally:

17 $${T}_{ij}={T}_{i}^{{\rm{obs}}}\dfrac{C{m}_{i}^{\alpha }{m}_{j}^{\beta }/{d}_{ij}^{\gamma }}{{\sum }_{l\ne i}\left(C{m}_{i}^{\alpha }{m}_{l}^{\beta }/{d}_{il}^{\gamma }\right)}\,,$$

where *d*_*ij*_ is the distance. The parameters *C* (scaling constant), *α* (origin mass exponent), *β* (destination mass exponent), and *γ* (distance decay exponent) are estimated by minimizing the negative log-likelihood of observed flows, the same method is used for the optimization of the parameters in all 4 models^53^.

The extended radiation model has one free parameter. The original radiation model^10^ is parameter-free and predicts flows from the spatial distribution of population alone. The extended version introduces a single free parameter *α* that calibrates the decay of opportunity influence^5^. The predicted flows are given by:

18 $${T}_{ij}={\gamma} \,{m}_{i}\frac{P\left(1| {m}_{i},{m}_{j},{a}_{ij}\right)}{{\sum }_{k}P\left(1| {m}_{i},{m}_{k},{a}_{ik}\right)}\,,$$

where

19 $$P(1| {m}_{i},{m}_{j},{a}_{ij})=\frac{\left[{({a}_{ij}+{m}_{j})}^{\alpha }-{a}_{ij}^{\alpha }\right]\,({m}_{i}^{\alpha }+1)}{({a}_{ij}^{\alpha }+1)\,\left[{({a}_{ij}+{m}_{j})}^{\alpha }+1\right]}\,,$$

and

20 $${a}_{ij}\equiv {m}_{i}+{s}_{ij}\,.$$

*γ* being a normalization constant. This extended version lets the decay of opportunity influence be calibrated, improving fit to empirical flows by capturing regional heterogeneity in commuters’ sensitivity to intervening opportunities^5,10^.

The BMS plausible gravity model has six parameters. It is a gravity-like model variant derived from Bayesian symbolic regression^13^. The predicted flow *T*_*ij*_ from origin *i* (with mass *m*_*i*_ and total observed outflow ${T}_{i}^{{\rm{obs}}}$) to destination *j* (with mass *m*_*j*_) is:

21 $${T}_{ij}={T}_{i}^{{\rm{obs}}}\dfrac{A{\left(1+B\dfrac{{m}_{i}{m}_{j}+C{m}_{j}+D}{{d}_{ij}^{\alpha }}\right)}^{\gamma }}{{\sum }_{k\ne i}A{\left(1+B\dfrac{{m}_{i}{m}_{k}+C{m}_{k}+D}{{d}_{ik}^{\alpha }}\right)}^{\gamma }}\,,$$

where *A*, *B*, *C*, *D*, *α*, and *γ* are parameters estimated.

### Parameter estimation and optimization

The parameters for all presented models (Utility, Gravity, and BMS Plausible Gravity) are estimated by maximizing the likelihood of observing the empirical commuting flows. This is equivalent to minimizing the negative log-likelihood of the data given the model and its parameters.

Let ${T}_{ij}^{{\rm{obs}}}$ be the observed flow of commuters from origin *i* to destination *j*, and let *P*_*ij*_(*θ*) be the probability of a commuter from *i* choosing *j*, or the predicted proportion of flow from *i* to *j*, as defined by a specific model with parameter vector *θ*. For models predicting flow proportions, the likelihood of observing the set of flows from a given origin *i*, ${T}_{i*}=\{{T}_{i1}^{{\rm{obs}}},{T}_{i2}^{{\rm{obs}}},\ldots,{T}_{iJ}^{{\rm{obs}}}\}$, given a total outflow ${O}_{i}={\sum }_{j}{T}_{ij}^{{\rm{obs}}}$, follows a multinomial distribution. The log-likelihood for a single origin *i* is:

22 $${{\mathcal{L}}}_{i}(\theta \big| {T}_{i*})=\sum _{j}{T}_{ij}^{{\rm{obs}}}\log {P}_{ij}(\theta )\,.$$

The total log-likelihood for the entire dataset is the sum over all origins:

23 $${\mathcal{L}}\left(\theta \big| {{\bf{T}}}^{{\rm{obs}}}\right)=\sum _{i}{{\mathcal{L}}}_{i}\left(\theta \big| {T}_{i*}\right)=\sum _{i}\sum _{j}{T}_{ij}^{{\rm{obs}}}\log {P}_{ij}(\theta )\,,$$

where **T**^obs^ represents the matrix of all observed flows.

The objective is to find the parameter vector $\hat{\theta }$ that maximizes ${\mathcal{L}}(\theta | {{\bf{T}}}^{{\rm{obs}}})$, or equivalently, minimizes $-{\mathcal{L}}(\theta | {{\bf{T}}}^{{\rm{obs}}})$.

24 $$\hat{\theta }=\arg {\mathop{\max }\limits_{\theta }}\,{\mathcal{L}}\left(\theta \big| {{\bf{T}}}^{{\rm{obs}}}\right)=\arg {\mathop{\min }\limits_{\theta }}\left[-{\mathcal{L}}\left(\theta \big| {{\bf{T}}}^{{\rm{obs}}}\right)\right]\,.$$

### Evaluation metrics

Model performance is assessed with two metrics. The Common Part of Commuters (CPC) measures the proportion of overlapping flows between the observed and the predicted matrices^7^,

25 $${\rm{CPC}}=\dfrac{2{\sum }_{i}{\sum }_{j}\min ({{T}_{ij}^{{\rm{obs}}}},{T}_{ij}^{{\rm{pred}}})}{{\sum }_{i}{\sum }_{j}({T}_{ij}^{{\rm{obs}}}+{T}_{ij}^{{\rm{pred}}})}.$$

The Pearson correlation (*r*) measures the strength of the linear relationship between observed and predicted flows,

26 $$r=\dfrac{{\sum }_{ij}\left({T}_{ij}^{{\rm{obs}}}-{\bar{T}}^{{\rm{obs}}}\right)\left({T}_{ij}^{{\rm{pred}}}-{\bar{T}}^{{\rm{pred}}}\right)}{\sqrt{{\sum }_{ij}{\left({T}_{ij}^{{\rm{obs}}}-{\bar{T}}^{{\rm{obs}}}\right)}^{2}{\sum }_{ij}{\left({T}_{ij}^{{\rm{pred}}}-{\bar{T}}^{{\rm{pred}}}\right)}^{2}}}\,.$$

### Accessibility measures

We compute two accessibility measures. Distance-weighted accessibility quantifies the difficulty of reaching job locations from a given origin *i*, considering the number of predicted flows and the impedance given by distance. This is, the sum of predicted flows *T*_*ij*_ to each destination *j*, weighted by the inverse of the distance *d*_*ij*_ to that destination, and then normalized by the total predicted outflow from *i*. Formally:

27 $${A}_{i}^{{\rm{DW}}}=\dfrac{{\sum }_{j}{T}_{ij}\left(1/{d}_{ij}\right)}{{\sum }_{j}{T}_{ij}}\,,$$

where *T*_*ij*_ are the predicted commuting flows from origin *i* to destination *j*, and *d*_*ij*_ is the distance between them (a minimum distance of 0.1 km is enforced to prevent division by zero if *d*_*ij*_ is very small). This metric, is widely used in accessibility studies^6,33,34^. It reflects that closer opportunities, or opportunities reached by larger flows, contribute more to accessibility.

Consumer-surplus accessibility, also known as logsum accessibility, is derived from RUM and is the expected maximum utility from *i*:

28 $${A}_{i}^{{\rm{CS}}}=\dfrac{1}{\lambda }\ln \sum _{j\in {J}_{i}}\exp ({V}_{ij})\,,$$

where *V*_*ij*_ is the systematic utility (Eq. (6)) and *λ* is the scale parameter of the Gumbel distribution (typically normalized to 1 in MNL estimation, so ${A}_{i}=\ln {\sum }_{j}\exp ({V}_{ij})$). This metric incorporates the attractiveness of all alternatives by using their utility ^14,34^. It represents the overall benefit or surplus a commuter gains from the available set of work opportunities.

The combined accessibility measure brings both types together in a single index. Each measure is standardized to *z*-scores within each city and the composite is their mean. This combined measure identifies areas with high benefit from both physical proximity and utility-based attractiveness (positive values) versus locations facing disadvantages in both dimensions (negative values). These measures are calculated for each origin zone *i* and analyzed spatially (Fig. 6) and by socioeconomic groups (Fig. 5).

All analyses were performed in Python 3.12.3, using NumPy 1.26.4, pandas 2.2.3, SciPy 1.16.0 for parameter optimization, GeoPandas 1.0.1 and Shapely 2.0.6 for the spatial operations, NetworkX 3.4.2 for the product-space networks, h3-py 4.2.2 for the hexagonal grid, and econci for the economic complexity calculations. The figures were produced with Matplotlib 3.9.2 and seaborn 0.13.2. The pipeline that derives home and work locations from the raw location pings was implemented in custom BigQuery SQL scripts.

### Reporting summary

Further information on research design is available in the Nature Portfolio Reporting Summary linked to this article.

## Supplementary information

Supplementary Information

Reporting Summary

Transparent Peer Review file

## Data Availability

The aggregated commuting flows and the derived ECI^emp^ and informality indices at the H3 zone level generated in this study are available in the project repository at https://github.com/humnetlab/workreach and archived at Zenodo (https://doi.org/10.5281/zenodo.21924441). The raw location-based services traces for Mexico City and Rio de Janeiro were obtained under a commercial license from Quadrant that does not permit redistribution and can be licensed from that provider. The Replica synthetic mobility and employment data for the Bay Area and Los Angeles were accessed under the agreement Replica holds with the University of California, Berkeley, which grants access to researchers affiliated with the university; other researchers can request access from Replica. The census and employment data used in this study are publicly available from the national statistical agencies, namely DENUE and the 2020 population census from INEGI for Mexico City, RAIS and the 2010 demographic census from IBGE for Rio de Janeiro, and the American Community Survey and the Longitudinal Employer-Household Dynamics data from the U.S. Census Bureau for the Bay Area and Los Angeles.

## Code availability

The code used to perform the analyses and generate the figures in this study is available in a public repository at https://github.com/humnetlab/workreach and archived at Zenodo (https://doi.org/10.5281/zenodo.21924441). The home and work locations were inferred from the raw location traces with our stay detection code, available at https://github.com/ollin18/gc-lbs and archived at Zenodo (https://doi.org/10.5281/zenodo.21924437).

## References

[1] Gobillon, L., Selod, H. & Zenou, Y. The mechanisms of spatial mismatch. *Urban Stud.* **44**, 2401–2427 (2007).

[2] Moreno-Monroy, A. I. & Ramos, F. R. The impact of public transport expansions on informality: The case of the São Paulo Metropolitan Region. *Res. Transp. Econ.* **88**, 100928 (2021).

[3] Graham, M. R. & Ong, P. Social, economic, spatial, and commuting patterns of informal jobholders. Tech. Rep., Center for Economic Studies, US Census Bureau (2007).

[4] Theodore, N. & Rondeau, J. L’informalité et la sélectivité stratégique de l’État : la montée de l’emploi précaire dans l’industrie de la construction aux États-Unis. *Lien social et Politiques* 114–136 (2016).

[5] Yang, Y., Herrera, C., Eagle, N. & González, M. C. Limits of predictability in commuting flows in the absence of data for calibration. *Sci. Rep.* **4**, 5662 (2014). 10.1038/srep05662 PMC4092333 25012599

[6] Piovani, D., Arcaute, E., Uchoa, G., Wilson, A. & Batty, M. Measuring accessibility using gravity and radiation models. *R. Soc. Open Sci.* **5**, 171668 (2018). 10.1098/rsos.171668 PMC6170557 30839729

[7] Lenormand, M., Bassolas, A. & Ramasco, J. J. Systematic comparison of trip distribution laws and models. *J. Transp. Geogr.* **51**, 158–169 (2016).

[8] Erlander, S. & Stewart, N. F. *The Gravity Model in Transportation Analysis: Theory and Extensions* (VSP, 1990).

[9] Barthelemy, M. The role of parsimonious models in addressing mobility challenges. *npj Sustai. Mobil. Transp.* **1**, 11 (2024).

[10] Simini, F., González, M. C., Maritan, A. & Barabási, A.-L. A universal model for mobility and migration patterns. *Nature* **484**, 96–100 (2012). 10.1038/nature10856 22367540

[11] Pappalardo, L., Rinzivillo, S. & Simini, F. Human mobility modelling: Exploration and preferential return meet the gravity model. *Procedia Comput. Sci.* **83**, 934–939 (2016).

[12] Simini, F., Barlacchi, G., Luca, M. & Pappalardo, L. A Deep Gravity model for mobility flows generation. *Nat. Commun.* **12**, 6576 (2021). 10.1038/s41467-021-26752-4 PMC8589995 34772925

[13] Cabanas-Tirapu, O., Danús, L., Moro, E., Sales-Pardo, M. & Guimerà, R. Human mobility is well described by closed-form gravity-like models learned automatically from data. *Nat. Commun.* **16**, 1336 (2025). 10.1038/s41467-025-56495-5 PMC11794706 39904994

[14] Ben-Akiva, M. & Bierlaire, M. Discrete choice methods and their applications to short term travel decisions. In *Handbook of Transportation Science* (Hall, R. W. ed.) 5–33 (Springer 1999).

[15] Train, K. E. *Discrete Choice Methods with Simulation* (Cambridge University Press, 2009).

[16] Hidalgo, C. A. & Hausmann, R. The building blocks of economic complexity. *Proc. Natl. Acad. Sci. USA* **106**, 10570–10575 (2009). 10.1073/pnas.0900943106 PMC2705545 19549871

[17] Hidalgo, C. A. Economic complexity theory and applications. *Nat. Rev. Phys.* **3**, 92–113 (2021).

[18] Balland, P.-A. et al. Complex economic activities concentrate in large cities. *Nat. Hum. Behav.* **4**, 248–254 (2020). 10.1038/s41562-019-0803-3 31932688

[19] Magalhães, L., Kuffer, M., Schwarz, N. & Haddad, M. Bringing economic complexity to the intra-urban scale: The role of services in the urban economy of Belo Horizonte, Brazil. *Appl. Geogr.* **150**, 102837 (2023).

[20] Juhász, S. et al. Amenity complexity and urban locations of socio-economic mixing. *EPJ Data Sci.* **12**, 34 (2023).

[21] Kim, J., Yu, D., Choi, H., Seo, D. & Jun, B. Redefining urban centrality: Integrating economic complexity indices into Central Place Theory (2024).

[22] Rossi Mori, L., Loreto, V. & Di Clemente, R. Time-space dynamics of income segregation in the city of Milan. *PNAS Nexus* **4**, pgaf283 (2025). 10.1093/pnasnexus/pgaf283 PMC12449691 40978089

[23] Chen, Z., Zhang, X. & van der Wouden, F. Neighborhood clusters and citywide technological diversification. *Res. Policy* **55**, 105397 (2026).

[24] Manning, A. *Monopsony in Motion: Imperfect Competition in Labor Markets* (Princeton University Press, 2003).

[25] Le Barbanchon, T., Rathelot, R. & Roulet, A. Gender differences in job search: Trading off commute against wage. * Q. J. Econ.* **136**, 381–426 (2021).

[26] Moretti, E. Local labor markets. In *Handbook of Labor Economics* (Ashenfelter, O. & Card, D. eds.) vol. 4B, 1237–1313 (Elsevier, 2011).

[27] Glaeser, E. L. & Gottlieb, J. D. The wealth of cities: agglomeration economies and spatial equilibrium in the United States. *J. Econ. Lit.* **47**, 983–1028 (2009).

[28] Abowd, J. M. et al. The LEHD infrastructure files and the creation of the quarterly workforce indicators. In *Producer Dynamics: New Evidence from Micro Data* (Dunne, T., Jensen, J. B. & Roberts, M. J. eds) 149–230 (University of Chicago Press, 2009).

[29] González, M. C., Hidalgo, C. A. & Barabási, A.-L. Understanding individual human mobility patterns. *Nature* **453**, 779–782 (2008). 10.1038/nature06958 18528393

[30] Song, C., Koren, T., Wang, P. & Barabási, A.-L. Modeling the scaling properties of human mobility. *Nat. Phys.* **6**, 818–823 (2010).

[31] Schläpfer, M. et al. The universal visitation law of human mobility. *Nature* **593**, 522–527 (2021). 10.1038/s41586-021-03480-9 34040209

[32] Geurs, K. T. & van Wee, B. Accessibility evaluation of land-use and transport strategies: review and research directions. *J. Transp. Geogr.* **12**, 127–140 (2004).

[33] Pereira, R. H. M., Banister, D., Schwanen, T. & Wessel, N. Distributional effects of transport policies on inequalities in access to opportunities in Rio de Janeiro. *J. Transp. Land Use* **12**, 741–764 (2019).

[34] Guzman, L. A., Cantillo-Garcia, V. A., Oviedo, D. & Arellana, J. How much is accessibility worth? Utility-based accessibility to evaluate transport policies. *J. Transp. Geogr.* **112**, 103683 (2023).

[35] Ben-Akiva, M. & Lerman, S. R. *Discrete Choice Analysis: Theory and Application to Travel Demand* (MIT Press, 2018).

[36] Chorus, C. G. Logsums for utility-maximizers and regret-minimizers, and their relation with desirability and satisfaction. *Transp. Res. Part A Policy Pract.* **46**, 1003–1012 (2012).

[37] de Jong, G., Daly, A., Pieters, M. & van der Hoorn, T. The logsum as an evaluation measure: review of the literature and new results. *Transp. Res. Part A Policy Pract.* **41**, 874–889 (2007).

[38] Hernandez, D. Uneven mobilities, uneven opportunities: social distribution of public transport accessibility to jobs and education in Montevideo. *J. Transp. Geogr.* **67**, 119–125 (2018).

[39] Bocarejo S, J. P. & Oviedo H, D. R. Transport accessibility and social inequities: a tool for identification of mobility needs and evaluation of transport investments. *J. Transp. Geogr.* **24**, 142–154 (2012).

[40] World Bank Group. World Bank Open Data. https://data.worldbank.org (2025).

[41] Giuliano, G., Hou, Y., Kang, S. & Shin, E. J. Polycentricity and the evolution of metropolitan spatial structure. *Growth Change* **53**, 593–627 (2022).

[42] Xu, Y. et al. Urban dynamics through the lens of human mobility. *Nat. Comput. Sci.* **3**, 611–620 (2023). 10.1038/s43588-023-00484-5 38177741

[43] Sørensen, T. A method of establishing groups of equal amplitude in plant sociology based on similarity of species and its application to analyses of the vegetation on Danish commons. *Biol. Skrifter/K. Dan. Videnskabernes Selsk.* **5**, 1 (1948).

[44] Liu, Z., Huang, L., Fan, C. & Mostafavi, A. Generating equitable urban human flows with a fairness-aware deep learning model. *Cities* **167**, 106296 10.1016/j.cities.2025.106296 (2025).

[45] Hidalgo, C. A., Klinger, B., Barabási, A.-L. & Hausmann, R. The product space conditions the development of nations. *Science* **317**, 482–487 (2007). 10.1126/science.1144581 17656717

[46] U.S. Bureau of Labor Statistics. Job Openings and Labor Turnover Survey (JOLTS) highlights. Federal Reserve Bank of St. Louis (FRED) (2024).

[47] Alaimo, V., Bosch, M., Kaplan, D. S., Pagés, C. & Ripani, L. *Jobs for Growth* (Inter-American Development Bank, 2017).

[48] McFadden, D. Conditional logit analysis of qualitative choice behavior. In *Frontiers in Econometrics*, Zarembka, P. (ed.) 105–142 (Academic Press, 1974).

[49] Bustos, S., Gomez, C., Hausmann, R. & Hidalgo, C. A. The dynamics of nestedness predicts the evolution of industrial ecosystems. *PLOS ONE* **7**, e49393 (2012). 10.1371/journal.pone.0049393 PMC3501524 23185326

[50] Ester, M., Kriegel, H.-P., Sander, J. & Xu, X. A density-based algorithm for discovering clusters in large spatial databases with noise. In *Proc. Second International Conference on Knowledge Discovery and Data Mining (KDD-96)*, 226–231 (AAAI Press, 1996).

[51] Soares, P. Econci. https://github.com/phcsoares/econci (2020).

[52] Virtanen, P. et al. SciPy 1.0: fundamental algorithms for scientific computing in Python. *Nat. Methods* **17**, 261–272 (2020). 10.1038/s41592-019-0686-2 PMC7056644 32015543

[53] Masucci, A. P., Serras, J., Johansson, A. & Batty, M. Gravity versus radiation models: on the importance of scale and heterogeneity in commuting flows. *Phys. Rev. E Stat. Nonlinear, Soft Matter Phys.* **88**, 022812 (2013). 10.1103/PhysRevE.88.022812 24032888

