# Supplementary material for: The WorkReach model for urban work location choices through economic complexity and informality
Source: Nat Commun. 2026 Oct 7;17:11422. doi: 10.1038/s41467-026-78293-3 (PMC13737282; doi:10.1038/s41467-026-78293-3)
Supplement: Supplementary file 1 [file 41467_2026_78293_MOESM1_ESM.pdf]

The WorkReach model for urban work location choices through economic complexity and informality

Supplementary Information

Ollin D. Langle-Chimal[1,*] Steffen Knoblauch[2,3] Marta C. González[1,4,*]

[1]Department of Civil and Environmental Engineering, University of California, Berkeley, Berkeley, CA 94720, USA.
[2]Interdisciplinary Centre of Scientific Computing (IWR), Heidelberg University, Heidelberg, Germany.
[3]Heidelberg Institute for Geoinformation Technology, Heidelberg University, Heidelberg, Germany.
[4]Department of City and Regional Planning, University of California, Berkeley, Berkeley, CA 94720, USA.

[*]Corresponding authors: ollin@berkeley.edu, martag@berkeley.edu

Supplementary Figures

1 Spatial autocorrelation of ECI^{emp} in the Bay Area . . . 4
2 Spatial autocorrelation of ECI^{emp} in Los Angeles . . . 5
3 Spatial autocorrelation of ECI^{emp} in Mexico City . . . 5
4 Spatial autocorrelation of ECI^{emp} in Rio de Janeiro . . . 6
5 Spatial autocorrelation of the informality rate in the Bay Area . . . 6
6 Spatial autocorrelation of the informality rate in Los Angeles . . . 7
7 Spatial autocorrelation of the informality rate in Mexico City . . . 7
8 Spatial autocorrelation of the informality rate in Rio de Janeiro . . . 8
9 Distributions of the transformed variables for the Bay Area . . . 8
10 Distributions of the transformed variables for Los Angeles . . . 9
11 Distributions of the transformed variables for Mexico City . . . 9
12 Distributions of the transformed variables for Rio de Janeiro . . . 9
13 Validation of the LBS commuting data against the EOD 2017 survey . . . 11
14 Informality rate in Mexico City proper versus the wider metropolitan area . . . 12
15 Shape of the transition weight $w_{ij}(\tau, k)$ at the fitted parameters . . . 13
16 Commuter flows for the Bay Area . . . 14
17 Commuter flows for Los Angeles . . . 15
18 Commuter flows for Mexico City . . . 16
19 Commuter flows for Rio de Janeiro . . . 17
20 Model performance across bins of ECI^{emp} . . . 18
21 Model performance across bins of the informality rate . . . 18
22 Pairwise Pearson correlations between prediction residuals . . . 20
23 WorkReach versus benchmark prediction residuals, by city and benchmark . . . 21
24 Train versus test predictive performance across five stratified folds . . . 23
25 Out-of-sample observed versus predicted flows for all models and cities . . . 24
26 Zone-level ECI^{emp} versus residential informality rate for each city . . . 26
27 Zone–sector binary RCA matrices sorted by diversity and ubiquity . . . 27
28 Product-space networks for the four cities . . . 29
29 Zone-level ECI^{emp} versus diversity and mean ubiquity . . . 31
30 Zone-level ECI^{emp} versus the employment-location income or wealth proxy . . . 33
31 Bootstrap confidence intervals for the ECI^{emp} minus proxy performance gap . . . 35
32 Within-sector commuting fraction by sector and city . . . 41
33 Sectoral relatedness versus commuting distance by origin informality group . . . 42
34 Accessibility versus informality rate for each city . . . 43
35 Distance-weighted versus consumer-surplus accessibility across cities . . . 44
36 Counterfactual ECI^{emp} uplift analysis . . . 47
37 Cross-city summary of the counterfactual inflow changes . . . 48

Supplementary Tables

1 Moran's I and p-value for spatial autocorrelation by city and variable . . . 4
2 Commuting distance and travel time in the EOD 2017 survey versus LBS . . . 10
3 LBS commuting distance at different spatial resolutions for Mexico City . . . 11
4 Permutation analysis of model performance (CPC) . . . 19
5 Mean train and test performance across five stratified folds . . . 22
6 Cross-correlation between ECI^{emp} and residential informality rate . . . 25
7 NODF nestedness scores for the four zone–sector binary matrices . . . 28
8 Product-space edge counts and mean degree under different filtering rules . . . 28
9 Variance in ECI^{emp} explained by diversity and mean ubiquity . . . 30
10 Model performance using ECI^{emp}, diversity, ubiquity or the orthogonal residual . . . 31
11 Predictive performance across all four cities for three model variants . . . 34
12 Likelihood-ratio tests for ECI^{emp} and the income or wealth proxy . . . 34
13 Predictive performance for WorkReach, SR-only, and combined (ECI^{emp} + SR) models . . . 36
14 Parameter estimates with multinomial bootstrap 95% confidence intervals . . . 37
15 Likelihood-ratio tests for ECI^{emp} and sectoral relatedness . . . 38
16 Marginal substitution rates for the sectoral-relatedness-only model . . . 39
17 Marginal substitution rates for the combined ECI^{emp} + SR model . . . 39

18 Census variables most correlated with the informality rate 45

Supplementary Notes

Supplementary Note 1: Spatial Properties of Model Inputs 4
Supplementary Note 2: Spatial Autocorrelation 4
Supplementary Note 3: Variable Distributions and Preprocessing 8
Supplementary Note 4: Validation of Commuting Data in Mexico City 10
Supplementary Note 5: Shape of Transition Weight 13
Supplementary Note 6: Flow Map Comparison 13
Supplementary Note 7: Permutation Based Null Model 17
Supplementary Note 8: Prediction Error Correlation Analysis 20
Supplementary Note 9: Out-of-Sample Cross-Validation 22
Supplementary Note 10: Robustness of the Economic Complexity Index 25
Supplementary Note 11: ECI^{emp} and Informality as Complementary Dimensions 25
Supplementary Note 12: Nested Structure of Zone–Sector Binary Matrices 27
Supplementary Note 13: Product-Space Network Structure 28
Supplementary Note 14: ECI^{emp} versus Diversity and Ubiquity 30
Supplementary Note 15: ECI^{emp} versus Income and Wealth Proxies 32
Supplementary Note 16: Sectoral Relatedness Analysis 36
Supplementary Note 17: Relationship between Accessibility and Informality 43
Supplementary Note 18: Correlation to Census Variables 45
Supplementary Note 19: Counterfactual ECI^{emp} Uplift and Worker Inflow 46

Supplementary References **49**

Supplementary Note 1: Spatial Properties of Model Inputs

In order to understand the spatial structure of the variables, we analyze their spatial autocorrelation. These analyses confirm that the variables are not randomly distributed but follow underlying spatial patterns, which justifies their use in the model.

Supplementary Note 2: Spatial Autocorrelation

We calculated Moran's I to test for spatial autocorrelation in the Economic Complexity Index (ECI^{emp}) and the informality rate for each city. Table 1 shows that both variables in all cities are statistically significant ($p = 0.001$) and that they all show a positive spatial autocorrelation. This result indicates that the spatial distribution of the measured variables is clustered, with high value areas tending to be near other high value areas and vice versa. Figures 1 through 8 represent the spatial patterns of ECI^{emp} and informality rate along the their Moran's I analysis.

Supplementary Table 1: Moran's I and pseudo p-value for spatial autocorrelation by city and variable. Significance is assessed with a one-sided permutation test (999 permutations); no permuted value reached the observed statistic, so $p = 0.001$, the smallest value the test can return. No correction for multiple comparisons was applied.

City	Variable	Moran's I	P-value
Bay Area	Informality rate	0.171	0.001
Los Angeles	Informality rate	0.253	0.001
Mexico City	Informality rate	0.407	0.001
Rio de Janeiro	Informality rate	0.467	0.001
Bay Area	ECI^{emp}	0.364	0.001
Los Angeles	ECI^{emp}	0.519	0.001
Mexico City	ECI^{emp}	0.594	0.001
Rio de Janeiro	ECI^{emp}	0.425	0.001

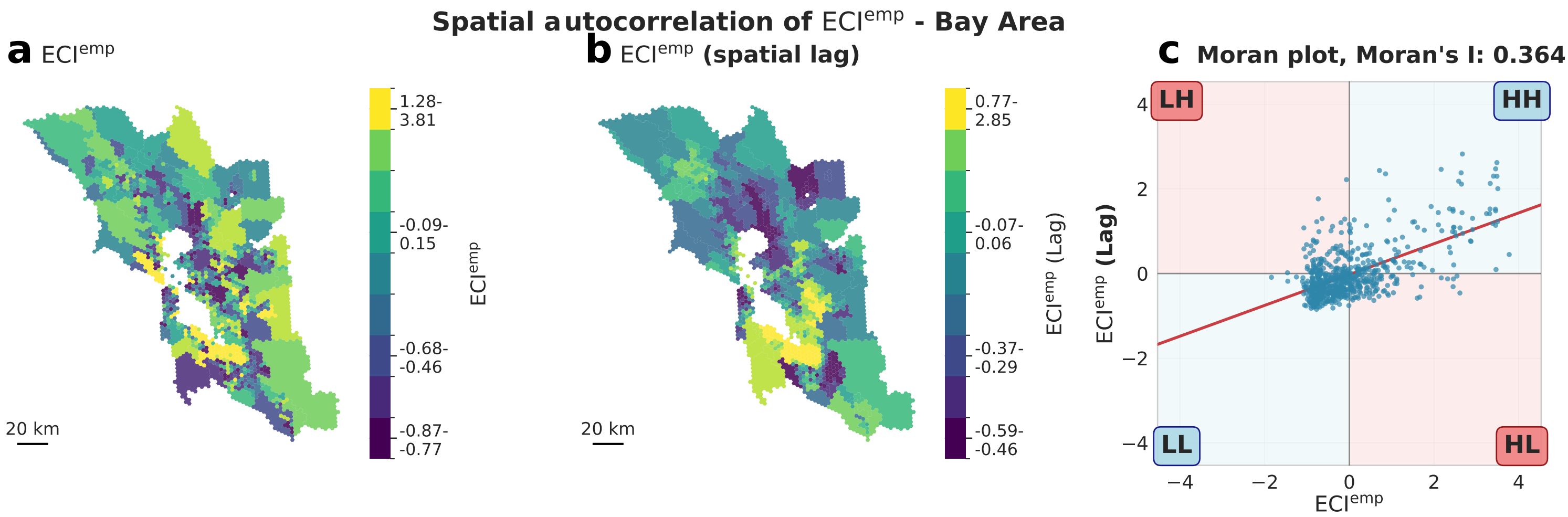

Supplementary Figure 1: Spatial autocorrelation analysis of Economic Complexity Index (ECI^{emp}) for the Bay Area. The figure shows **a** the choropleth map of ECI^{emp}, **b** the map of the spatial lag of ECI^{emp}, and **c** the Moran's I scatter plot (Moran's I = 0.364). Moran's I is computed over n = 677 zones using a k-nearest-neighbor spatial weights matrix with k = 8. Significance is assessed with a one-sided permutation test (999 permutations); no permuted value reached the observed statistic, so $p = 0.001$, the smallest value the test can return. No correction for multiple comparisons was applied.

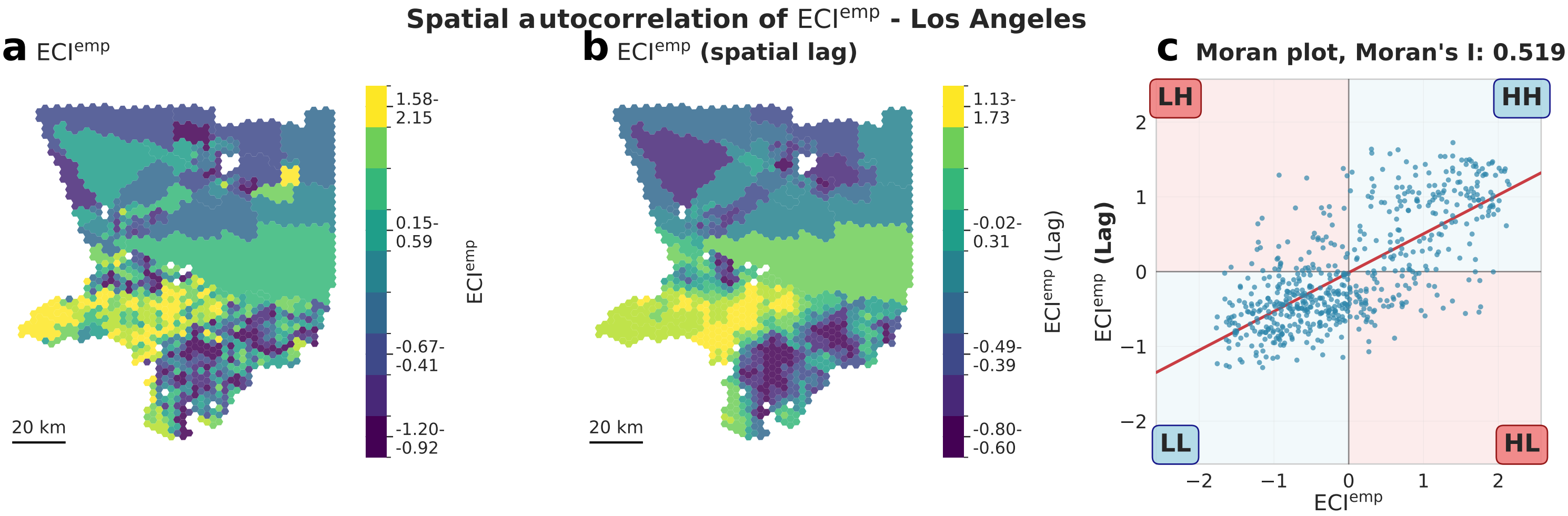

Supplementary Figure 2: Spatial autocorrelation analysis of Economic Complexity Index (ECI^{emp}) for Los Angeles. The figure shows **a** the choropleth map of ECI^{emp}, **b** the map of the spatial lag of ECI^{emp}, and **c** the Moran's I scatter plot (Moran's I = 0.519). Moran's I is computed over n = 605 zones using a k-nearest-neighbor spatial weights matrix with k = 8. Significance is assessed with a one-sided permutation test (999 permutations); no permuted value reached the observed statistic, so $p = 0.001$, the smallest value the test can return. No correction for multiple comparisons was applied.

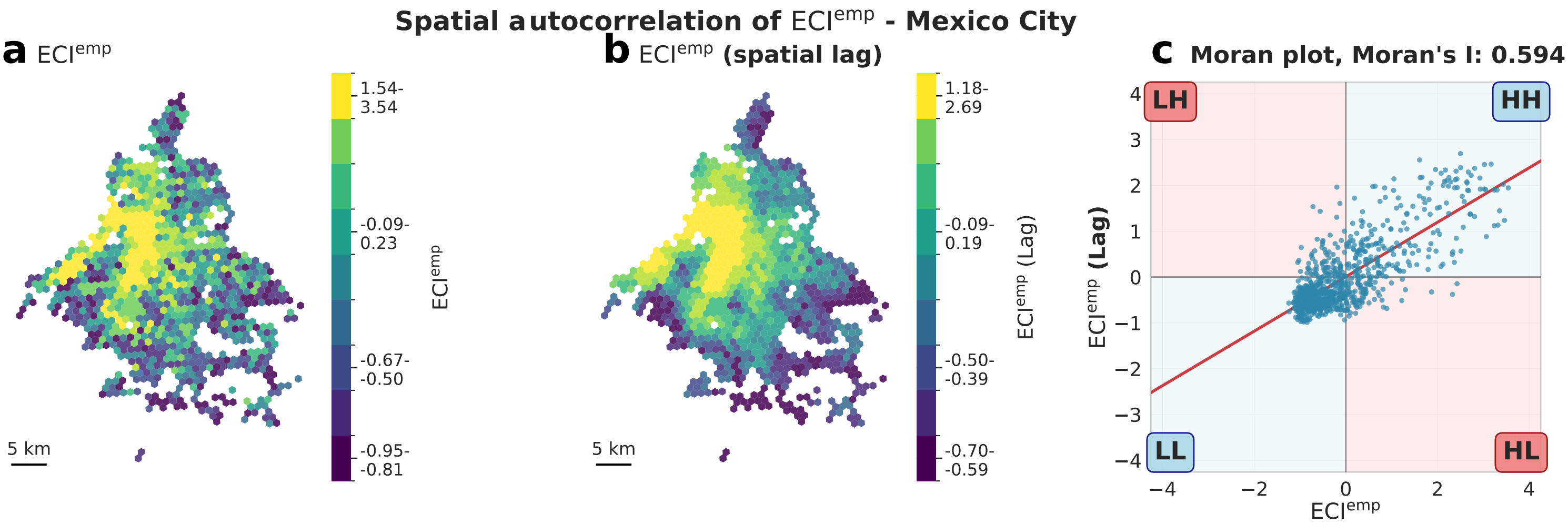

Supplementary Figure 3: Spatial autocorrelation analysis of Economic Complexity Index (ECI^{emp}) for Mexico City. The figure shows **a** the choropleth map of ECI^{emp}, **b** the map of the spatial lag of ECI^{emp}, and **c** the Moran's I scatter plot (Moran's I = 0.594). Moran's I is computed over n = 793 zones using a k-nearest-neighbor spatial weights matrix with k = 8. Significance is assessed with a one-sided permutation test (999 permutations); no permuted value reached the observed statistic, so $p = 0.001$, the smallest value the test can return. No correction for multiple comparisons was applied.

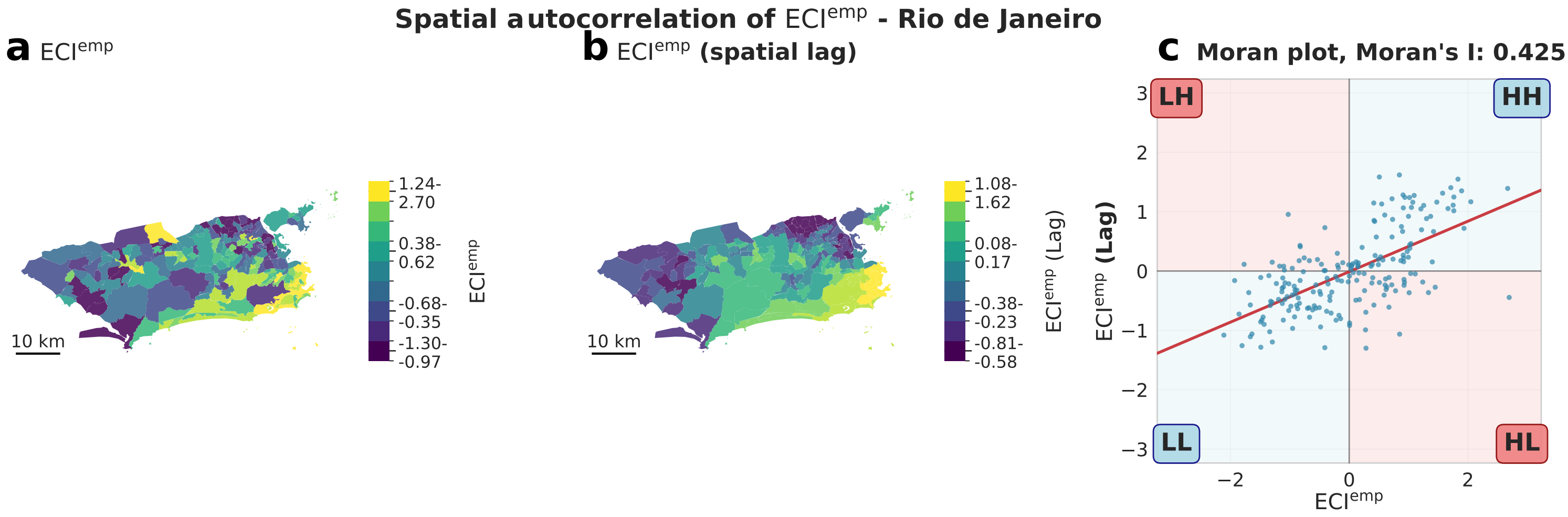

Supplementary Figure 4: Spatial autocorrelation analysis of Economic Complexity Index (ECI^{emp}) for Rio de Janeiro. The figure shows **a** the choropleth map of ECI^{emp}, **b** the map of the spatial lag of ECI^{emp}, and **c** the Moran's I scatter plot (Moran's I = 0.425). Moran's I is computed over n = 199 zones using a k-nearest-neighbor spatial weights matrix with k = 8. Significance is assessed with a one-sided permutation test (999 permutations); no permuted value reached the observed statistic, so $p = 0.001$, the smallest value the test can return. No correction for multiple comparisons was applied.

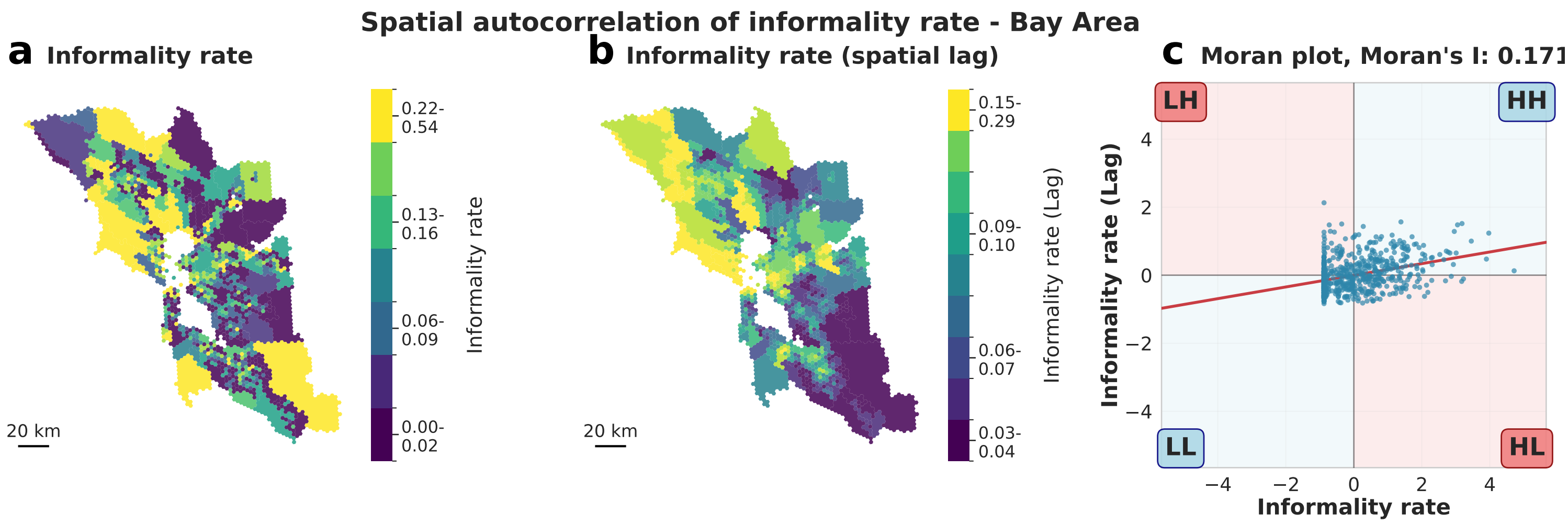

Supplementary Figure 5: Spatial autocorrelation analysis of informality rate for the Bay Area. The figure shows **a** the choropleth map of informality rate, **b** the map of the spatial lag of informality rate, and **c** the Moran's I scatter plot (Moran's I = 0.171). Moran's I is computed over n = 677 zones using a k-nearest-neighbor spatial weights matrix with k = 8. Significance is assessed with a one-sided permutation test (999 permutations); no permuted value reached the observed statistic, so $p = 0.001$, the smallest value the test can return. No correction for multiple comparisons was applied.

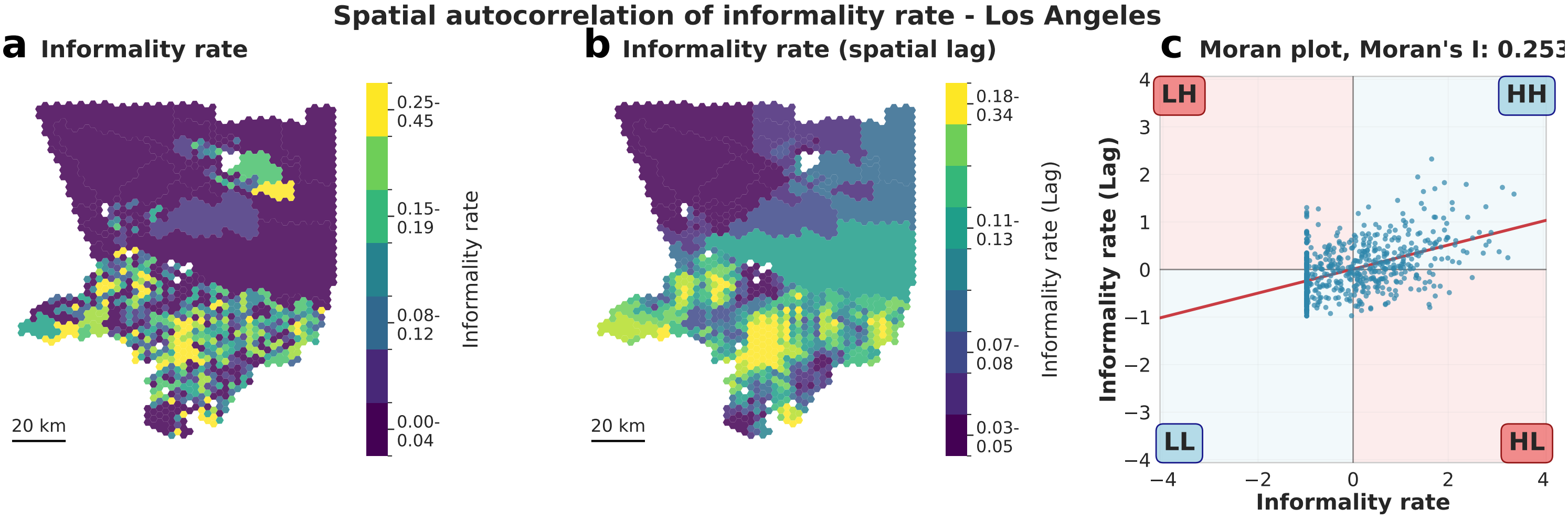

Supplementary Figure 6: Spatial autocorrelation analysis of informality rate for Los Angeles. The figure shows **a** the choropleth map of informality rate, **b** the map of the spatial lag of informality rate, and **c** the Moran's I scatter plot (Moran's I = 0.253). Moran's I is computed over n = 605 zones using a k-nearest-neighbor spatial weights matrix with k = 8. Significance is assessed with a one-sided permutation test (999 permutations); no permuted value reached the observed statistic, so $p = 0.001$, the smallest value the test can return. No correction for multiple comparisons was applied.

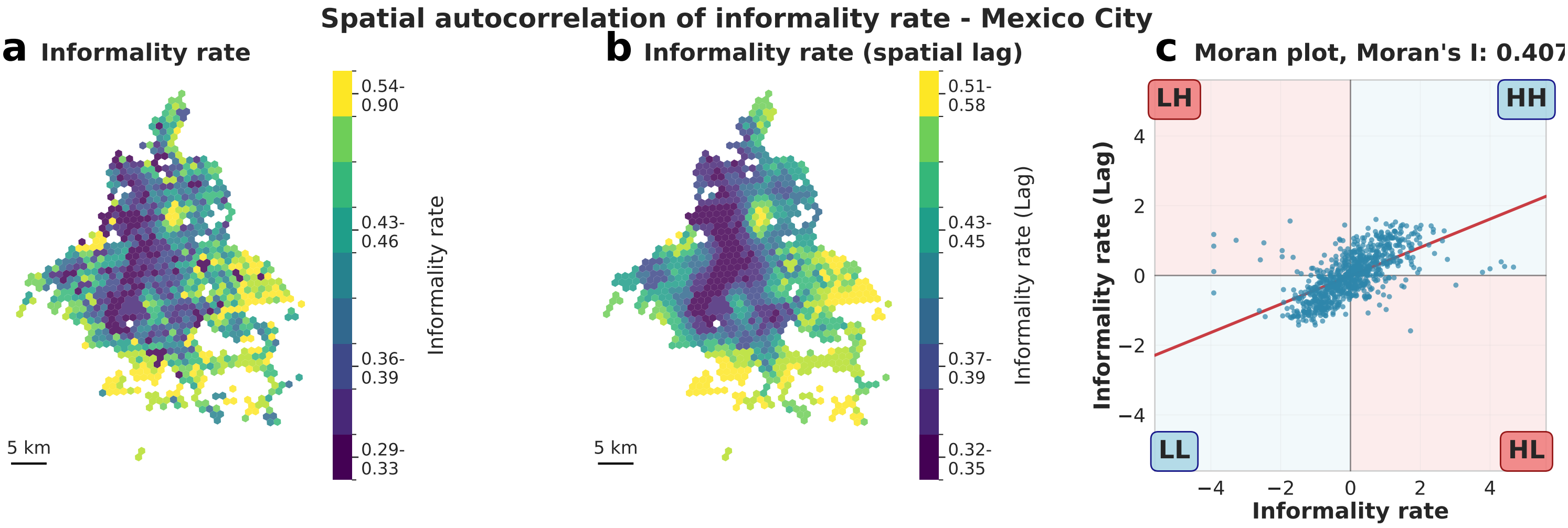

Supplementary Figure 7: Spatial autocorrelation analysis of informality rate for Mexico City. The figure shows **a** the choropleth map of informality rate, **b** the map of the spatial lag of informality rate, and **c** the Moran's I scatter plot (Moran's I = 0.407). Moran's I is computed over n = 793 zones using a k-nearest-neighbor spatial weights matrix with k = 8. Significance is assessed with a one-sided permutation test (999 permutations); no permuted value reached the observed statistic, so $p = 0.001$, the smallest value the test can return. No correction for multiple comparisons was applied.

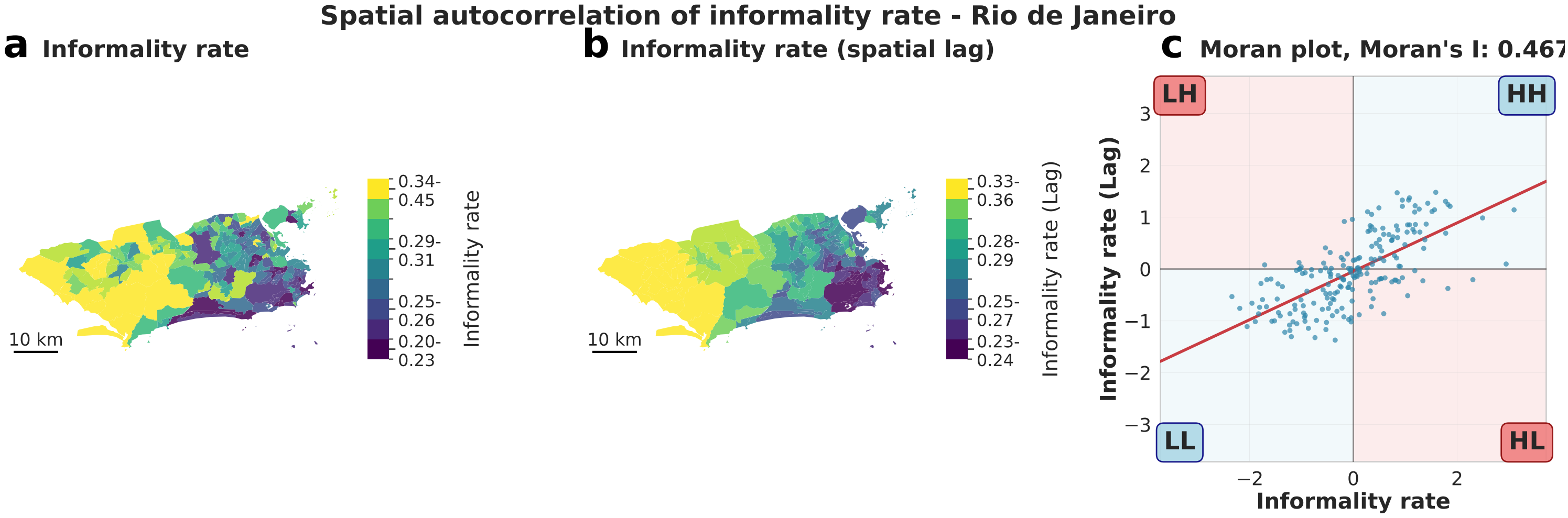

Supplementary Figure 8: Spatial autocorrelation analysis of informality rate for Rio de Janeiro. The figure shows **a** the choropleth map of informality rate, **b** the map of the spatial lag of informality rate, and **c** the Moran's I scatter plot (Moran's I = 0.467). Moran's I is computed over n = 199 zones using a k-nearest-neighbor spatial weights matrix with k = 8. Significance is assessed with a one-sided permutation test (999 permutations); no permuted value reached the observed statistic, so $p = 0.001$, the smallest value the test can return. No correction for multiple comparisons was applied.

Supplementary Note 3: Variable Distributions and Preprocessing

The WorkReach model has 3 input variables (Distance, ECI^{emp}, informality rate) which are pre-processed for model stability and interpretability of results. All three variables are min-max scaled to the $[0, 1]$ interval. Figures 9 to 12 show the final distributions of these variables that were used as input to the model.

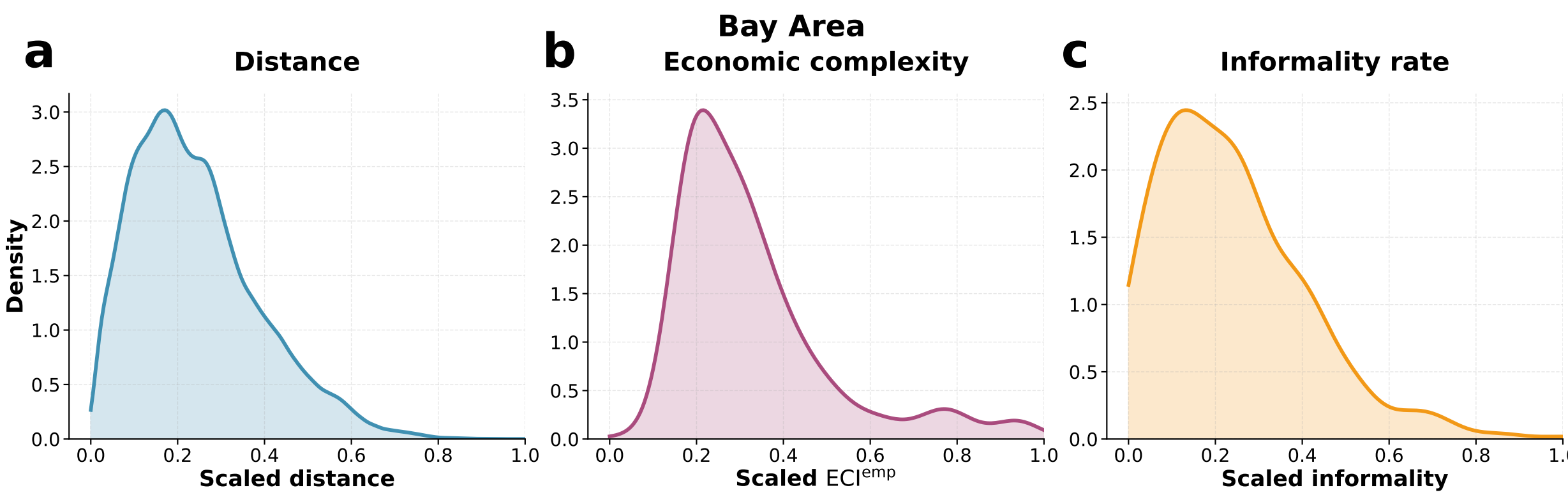

Supplementary Figure 9: Distributions of the transformed variables for the Bay Area. The panels show the distribution of **a** scaled commuting distance, **b** scaled ECI^{emp} and **c** scaled informality rate. Distances are taken over n = 457,652 origin-destination pairs and the two zone attributes over n = 677 zones.

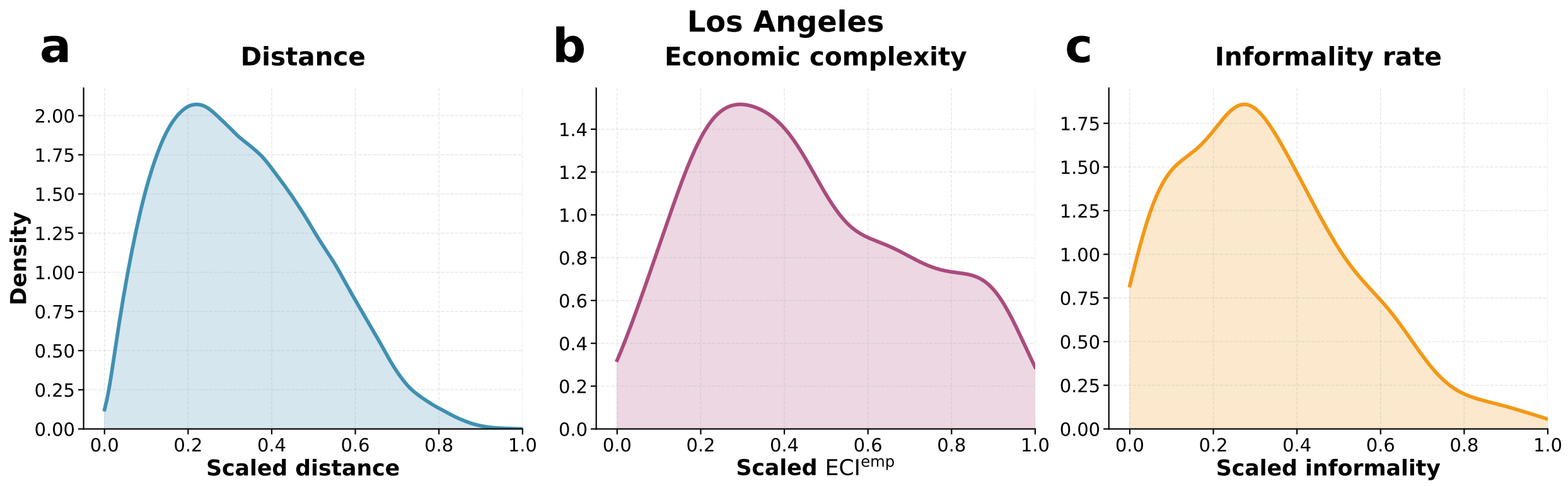

Supplementary Figure 10: Distributions of transformed variables for Los Angeles. The panels show the distribution of **a** scaled commuting distance, **b** scaled $\mathrm{ECI}^{\mathrm{emp}}$ and **c** scaled informality rate. Distances are taken over n = 365,420 origin-destination pairs and the two zone attributes over n = 605 zones.

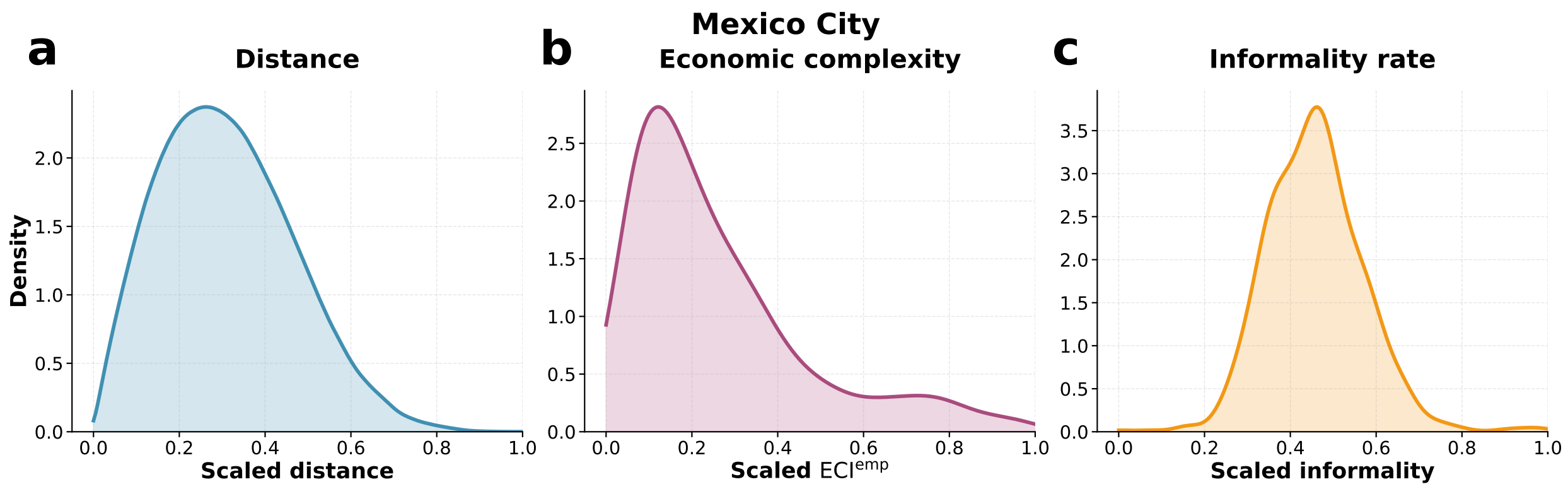

Supplementary Figure 11: Distributions of transformed variables for Mexico City. The panels show the distribution of **a** scaled commuting distance, **b** scaled $\mathrm{ECI}^{\mathrm{emp}}$ and **c** scaled informality rate. Distances are taken over n = 628,056 origin-destination pairs and the two zone attributes over n = 793 zones.

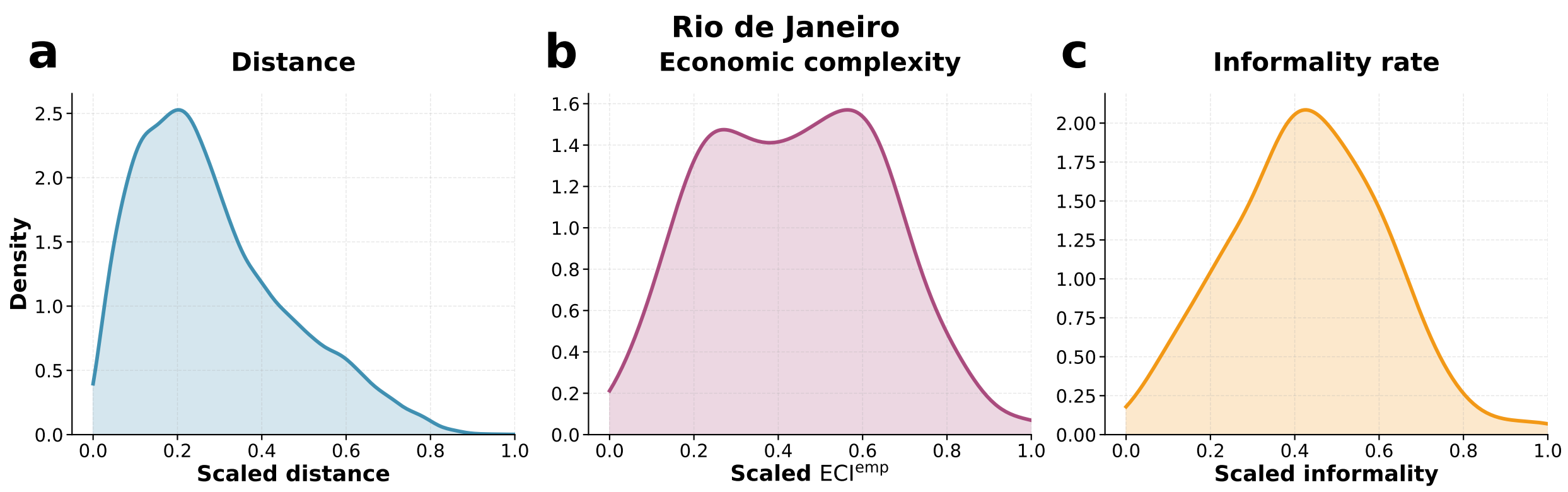

Supplementary Figure 12: Distributions of the transformed variables for Rio de Janeiro. The panels show the distribution of **a** scaled commuting distance, **b** scaled $\mathrm{ECI}^{\mathrm{emp}}$ and **c** scaled informality rate. Distances are taken over n = 39,402 origin-destination pairs and the two zone attributes over n = 199 zones.

Supplementary Note 4: Validation of Commuting Data in Mexico City

To validate our LBS-derived commuting data for Mexico City, we compare aggregate commuting distances against the 2017 Origin-Destination Survey (EOD 2017), conducted by INEGI across the Zona Metropolitana del Valle de México (ZMVM) [1]. The EOD collected trip records for the entire metropolitan area, with geographic detail at the district level. Districts are spatial units defined by the survey to contain roughly equal populations; CDMX proper comprises 85 districts, distinct from the 16 administrative alcaldías. No equivalent OD survey exists for Rio de Janeiro; because both cities rely on the same LBS vendor and processing pipeline, this validation also provides indirect support for the quality of the Rio de Janeiro commuting data.

Distance computation from the EOD survey

The EOD 2017 does not report trip-level distances directly. We compute centroid-to-centroid Haversine distances between origin and destination districts using the geographic center of each administrative unit. The 85 CDMX districts vary considerably in size; the mean minimum bounding circle (MBC) radius is 3.14 km and the median is 2.61 km. For intra-district trips (where origin and destination share the same district), the centroid-to-centroid distance would be zero. We instead assign these trips a distance equal to the mean MBC radius of their district, providing a rough but non-trivial estimate of the average within-district trip length.

Distance comparison

Supplementary Table 2 summarizes the commuting distances and travel times from both data sources. We subset the EOD's district-level OD matrix to the 85 districts within CDMX proper to match the spatial extent of our LBS data. From this subset, we obtain a mean centroid-to-centroid distance of 7.74 km (median 6.23 km) and a mean travel time of 50.28 minutes (median 45.00 min). Our LBS data at H3 resolution 8 (the level used in our analysis, with cell edge length ≈0.575 km) yields a mean Haversine distance of 6.02 km and a median of 4.30 km for CDMX.

Supplementary Table 2: Commuting distance and travel time comparison between the EOD 2017 survey and LBS data for Mexico City. Survey distances are computed as centroid-to-centroid Haversine between districts; LBS distances are centroid-to-centroid Haversine between H3 cells. Standard errors are shown in parentheses.

Dataset	Mean	Median	Std. error
EOD 2017 survey			
Distance, ZMVM (km)	10.18	7.39	0.030
Distance, CDMX (km)	7.74	6.23	0.028
Travel time, ZMVM (min)	55.84	50.00	0.131
Travel time, CDMX (min)	50.28	45.00	0.169
LBS data (CDMX)			
District level	7.00	5.09	0.168
AGEB level	6.01	4.20	0.020
H3 resolution 6	7.56	6.88	0.400
H3 resolution 7	6.29	4.53	0.102
H3 resolution 8	6.02	4.30	0.030
H3 resolution 9	5.99	4.21	0.021

Sensitivity to spatial resolution

To understand how spatial aggregation affects distance estimates, we re-compute the LBS distances at multiple resolutions: the 85 EOD districts, the native AGEB (census tract) level, and four H3 hexagonal grid levels (resolutions 6 through 9). Supplementary Table 3 reports the cell radius, number of cells covering CDMX, and the resulting mean and median distances for each level.

At H3 resolution 6, the 35 hexagonal cells covering CDMX are most comparable in number and size to the 16 survey districts. At this level, the LBS data yields a mean distance of 7.56 km and a median of 6.88 km, compared to the survey's 7.74 km (mean) and 6.23 km (median) for CDMX proper. The mean difference is only 0.18 km. As resolution increases (smaller cells), distances converge downward toward the

Supplementary Table 3: LBS commuting distance at different spatial resolutions for CDMX. Cell radius refers to the circumscribed radius of each hexagonal cell (or the mean MBC radius for AGEBs). The number of cells, mean distance, and median distance are shown for each level.

Resolution	Cell radius (km)	Cells	Mean (km)	Median (km)
District	3.136	85	7.00	5.09
AGEB	0.478	2,431	5.98	4.20
H3 res. 6	4.023	35	7.56	6.88
H3 res. 7	1.521	250	6.29	4.53
H3 res. 8	0.575	1,766	6.02	4.30
H3 res. 9	0.217	12,325	5.99	4.21

AGEB-level values, where finer spatial detail allows the identification of short-range commutes that coarser grids merge into single zones. The stability of estimates between H3 resolution 8 (our analysis level) and the finest resolution 9 indicates that our working resolution adequately captures the distance distribution without excessive aggregation.

Origin-destination matrix comparison

Beyond aggregate distance metrics, we compare individual zone-to-zone flows in the origin-destination matrices. Both the EOD survey and our LBS data are aggregated to Mexico City's 85 districts. Supplementary Fig. 13 shows the OD heatmaps from the EOD survey and the LBS data alongside a scatter plot of district-level OD flows. The spatial patterns are visually consistent: both matrices exhibit a strong diagonal (intra-district flows) and highlight the attraction of central districts. The Pearson correlation between the two matrices is $r = 0.76$ and the cosine similarity is 0.887, indicating strong concordance in the overall structure of commuting flows despite differences in data source, time period, and collection methodology. The LBS data also captures OD pairs for which the survey reports zero flows, providing broader spatial coverage than the survey alone. Together with the distance estimates at comparable spatial resolutions (mean difference < 0.2 km at H3 resolution 6), these results confirm that the LBS data reliably represents Mexico City's commuting structure.

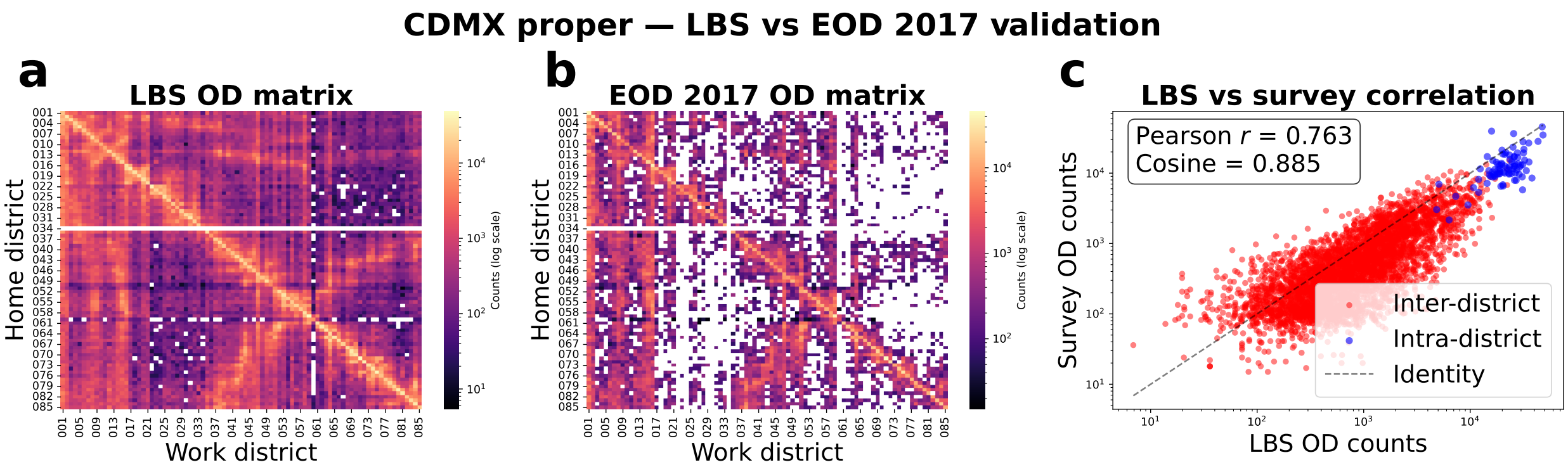

Supplementary Figure 13: Validation of LBS commuting data against the EOD 2017 survey for Mexico City proper, aggregated to 85 districts. **a** OD heatmap from LBS data. **b** OD heatmap from the EOD survey. **c** Scatter plot of district-level OD flows (Pearson $r = 0.76$; cosine similarity $= 0.887$). Both matrices cover n $= 85$ districts within Mexico City proper. The reported correlation is a two-sided Pearson coefficient with $n - 2$ degrees of freedom.

Spatial coverage of the informality gradient in Mexico City

Informality enters WorkReach as the origin-side covariate, describing the socioeconomic context of a worker's home zone. Capturing how commuting varies with it depends on the model observing the full span of residential informality within a city. Because residential informality varies across a metropolitan area, the boundary drawn around a city affects how much of that variation enters the analysis. In Mexico City, our analysis covers CDMX proper, the administrative boundary of the city, while commuting extends beyond it into the surrounding municipalities of the Estado de México and Hidalgo, which with CDMX proper form the

Zona Metropolitana del Valle de México (ZMVM). We therefore examined how the informality distribution within CDMX proper ($n = 908$ zones) compares with that of the full metropolitan area ($n = 2{,}610$ zones), computing the rate for every populated H3 zone with the same census-based informality proxy.

Supplementary Fig. 14 compares the two distributions, which cover essentially the same range of informality, each running from near-zero informality to highly informal zones (from near 0 to 0.90 in CDMX proper, and from near 0 to 0.96 across the ZMVM). They overlap substantially and differ mainly in where their mass falls, with the ZMVM sitting somewhat higher (median 0.486 versus 0.414), reflecting the larger share of high-informality zones in the conurbated periphery. The two-sample Kolmogorov–Smirnov statistic, $D = 0.282$ (the maximum gap between the cumulative distributions), captures this moderate shift.

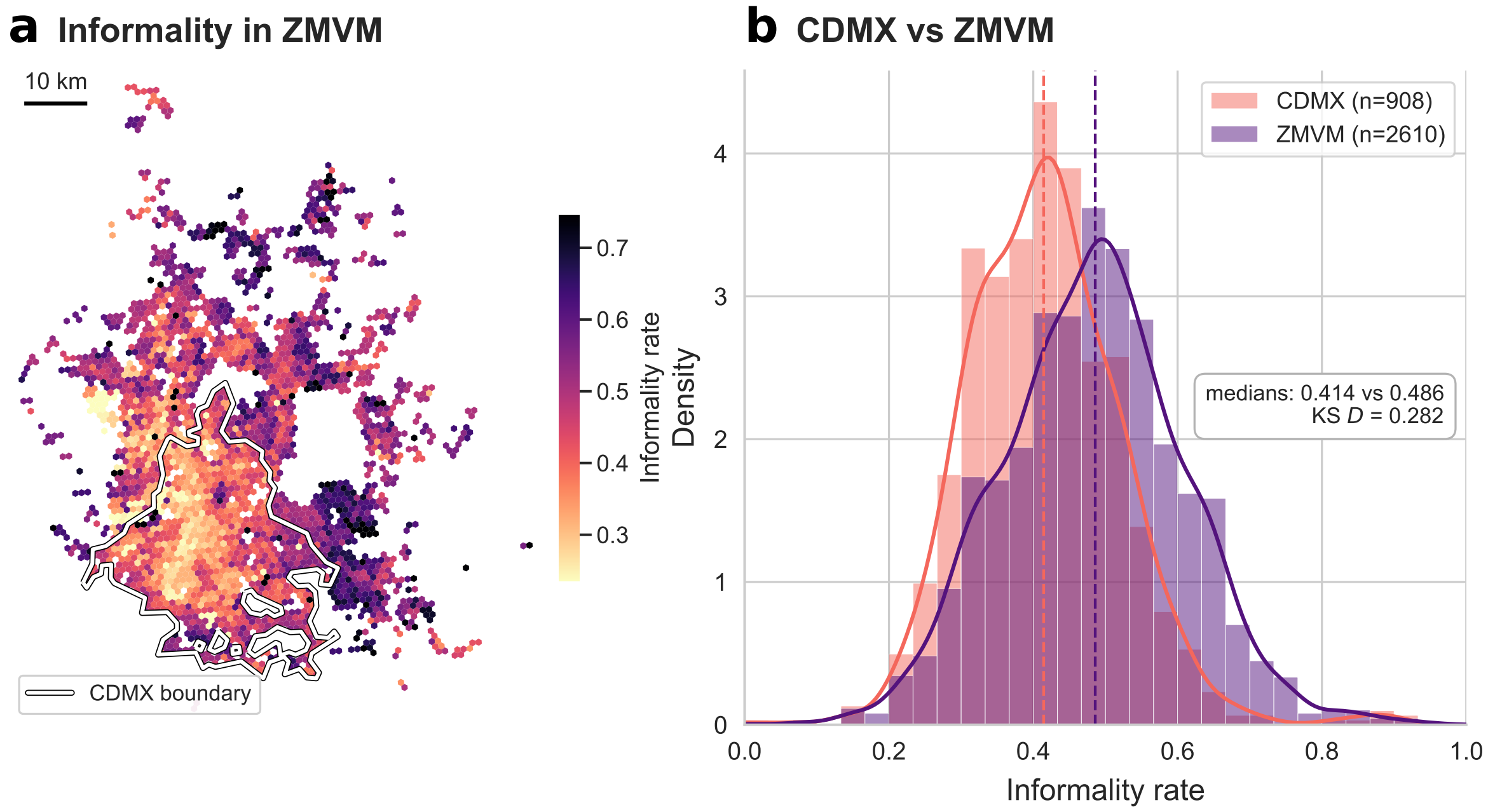

Supplementary Figure 14: Informality rate in CDMX proper versus the wider metropolitan area (ZMVM). **a** Informality rate across all populated H3 zones of the ZMVM. **b** Distribution of the informality rate within CDMX proper and across the full ZMVM. The two distributions span essentially the same range and overlap substantially (medians 0.414 versus 0.486; two-sample Kolmogorov–Smirnov $D = 0.282$), with the ZMVM shifted toward higher informality. Distributions cover n = 908 AGEBs within Mexico City proper and n = 2,610 across the wider metropolitan area; the reported statistic is the two-sample Kolmogorov-Smirnov distance.

Supplementary Note 5: Shape of Transition Weight

A core component of our WorkReach model is the transition weight function $w_{ij}(\tau, k)$, which introduces a behavioral shift in job choice based on distance. Figure 15 shows the shape of this transition weight for each city.

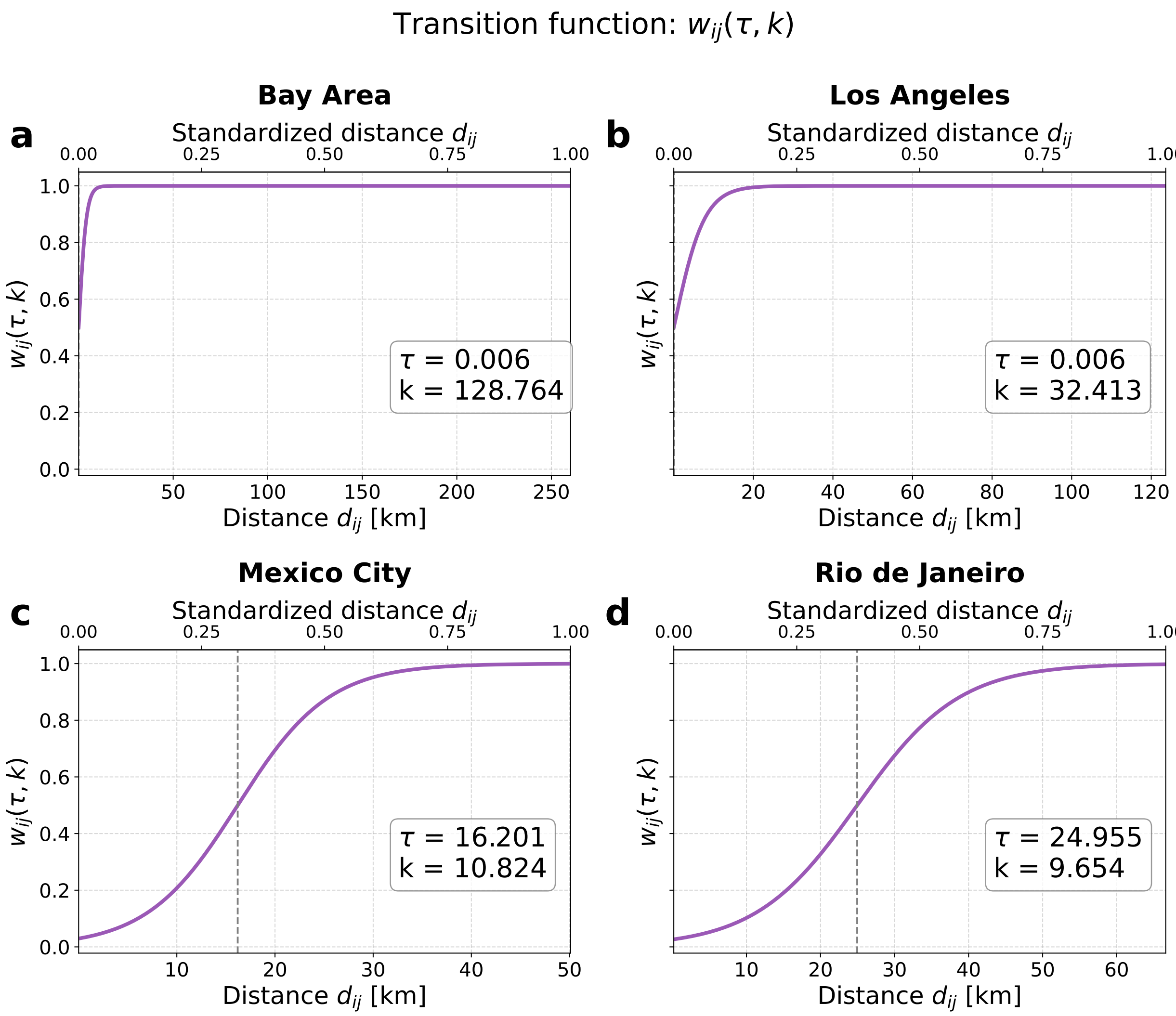

Supplementary Figure 15: Shape of the transition weight with the optimized parameters, $w_{ij}(\tau, k)$, for all four cities. The w represents a logistic function which models the behavioral shift in local vs opportunity based job choice. For $d_{ij} > \tau$, the influence of ECI^{emp} and informality in the utility function is maximal. The parameters (τ, k) show differences by region. While U.S. cities have lower thresholds (τ) and steeper transitions (k), the Latin American cities have a higher distance threshold and a less steep transition. Panels **a**–**d** correspond to the Bay Area, Los Angeles, Mexico City and Rio de Janeiro, respectively. Each curve is drawn from the parameters fitted to n = 457,652, 365,420, 628,056 and 39,402 origin-destination pairs respectively.

Supplementary Note 6: Flow Map Comparison

We compare the predictive performance of our WorkReach model, by comparing its generated flows against the observed ones and the obtained by other three benchmark models. Figures 16 to 19 show the flow visualizations for each model and city along with the observed flows.

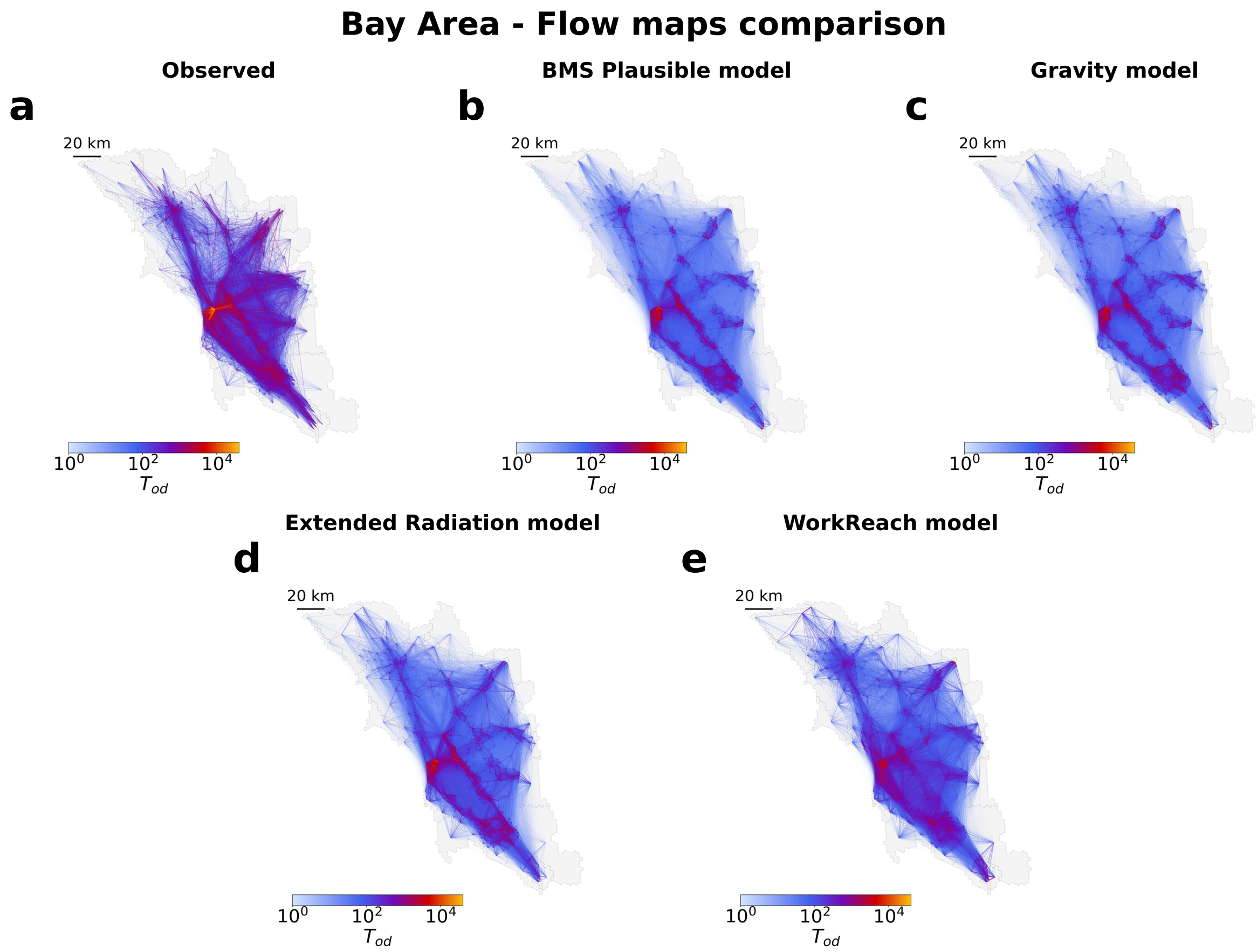

Supplementary Figure 16: Commuter flows for the Bay Area. **a** Observed flows **b** BMS Plausible, **c** Gravity, **d** Extended Radiation, and **e** WorkReach. Each panel draws n = 457,652 origin-destination pairs between 677 zones. Line color encodes the number of commuters on a logarithmic scale, as given by the color bar in each panel.

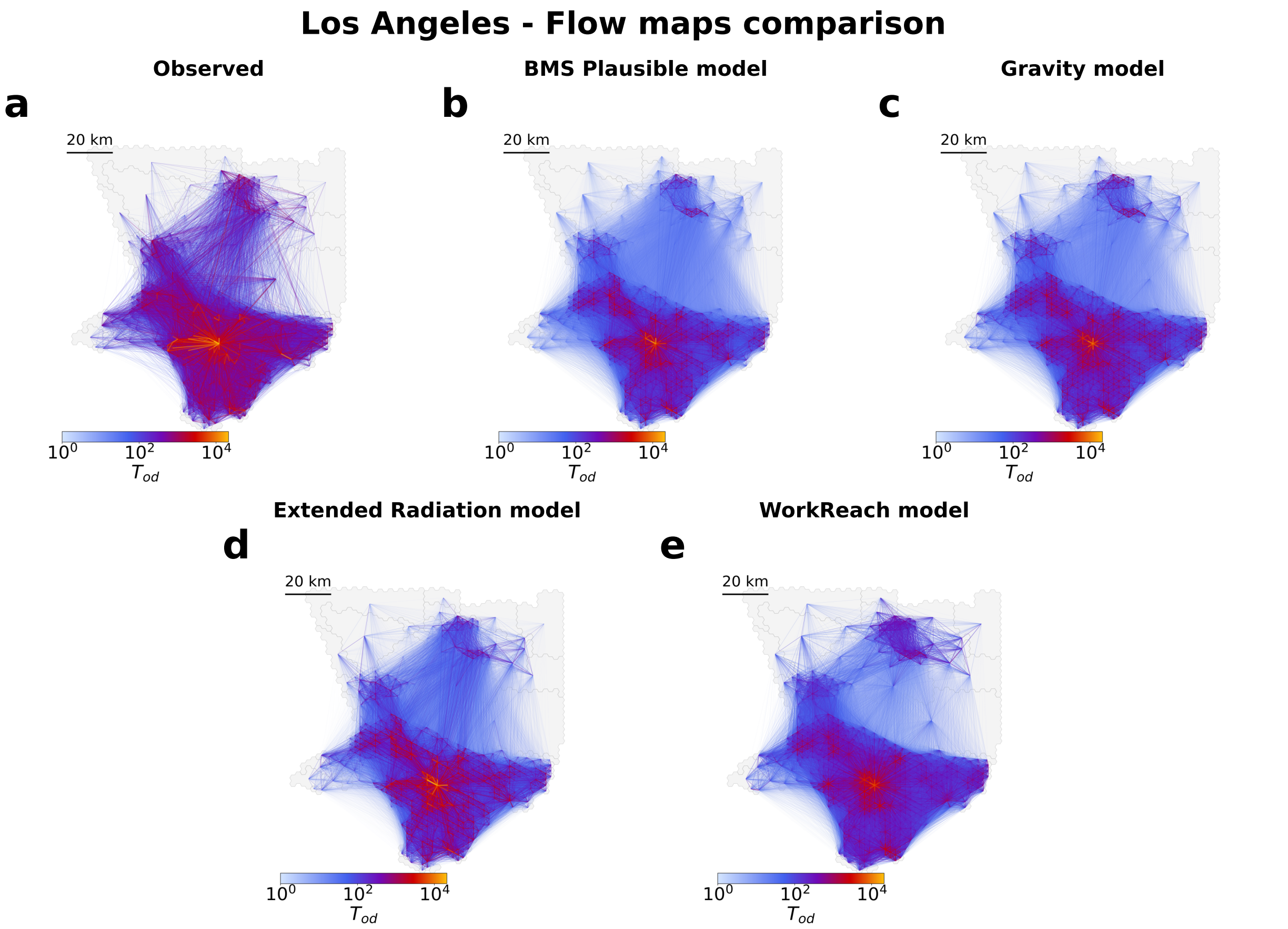

Supplementary Figure 17: Commuter flows for Los Angeles. **a** Observed flows **b** BMS Plausible, **c** Gravity, **d** Extended Radiation, and **e** WorkReach. Each panel draws n = 365,420 origin-destination pairs between 605 zones. Line color encodes the number of commuters on a logarithmic scale, as given by the color bar in each panel.

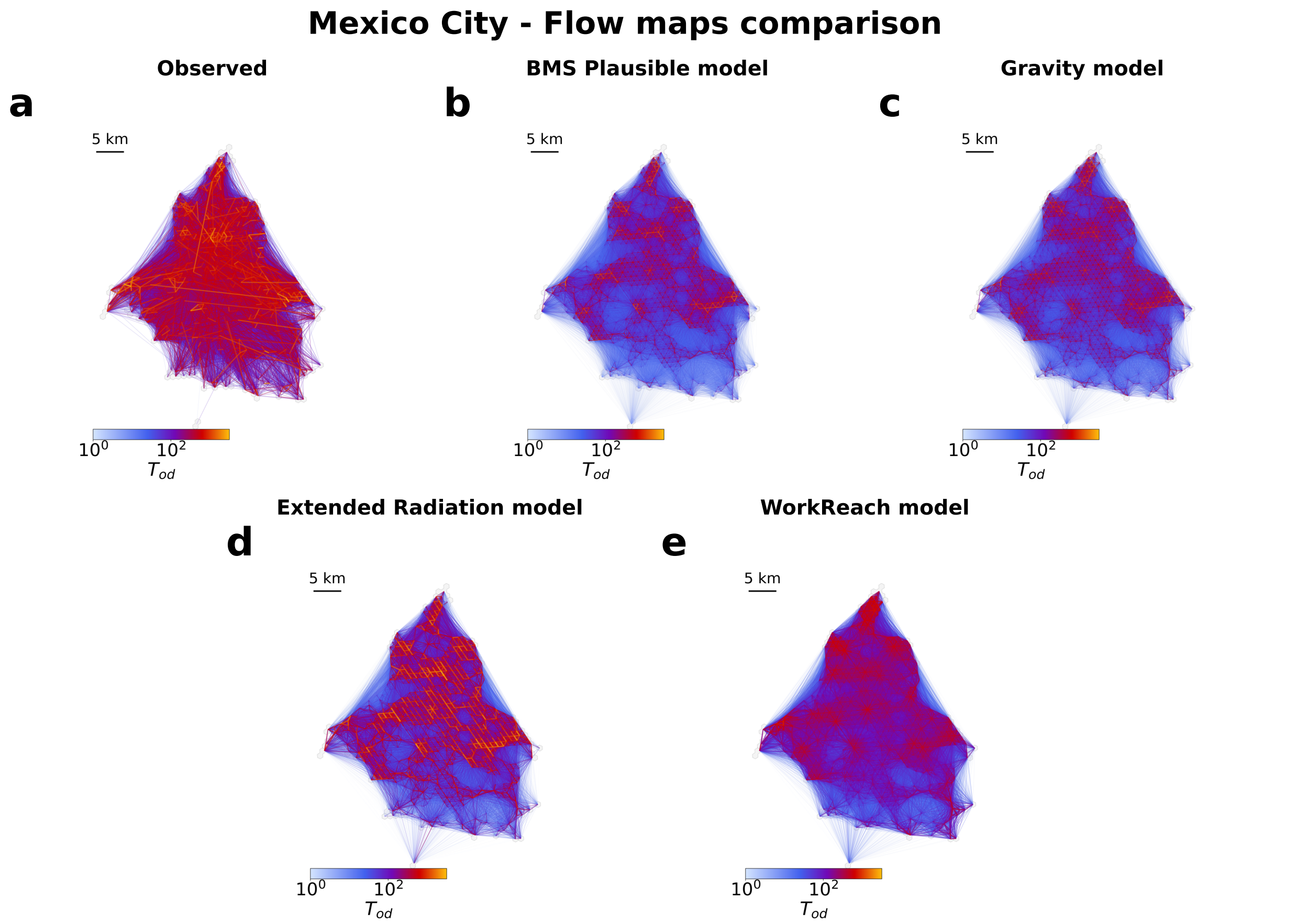

Supplementary Figure 18: Commuter flows for Mexico City. **a** Observed flows **b** BMS Plausible, **c** Gravity, **d** Extended Radiation, and **e** WorkReach. Each panel draws n = 628,056 origin-destination pairs between 793 zones. Line color encodes the number of commuters on a logarithmic scale, as given by the color bar in each panel.

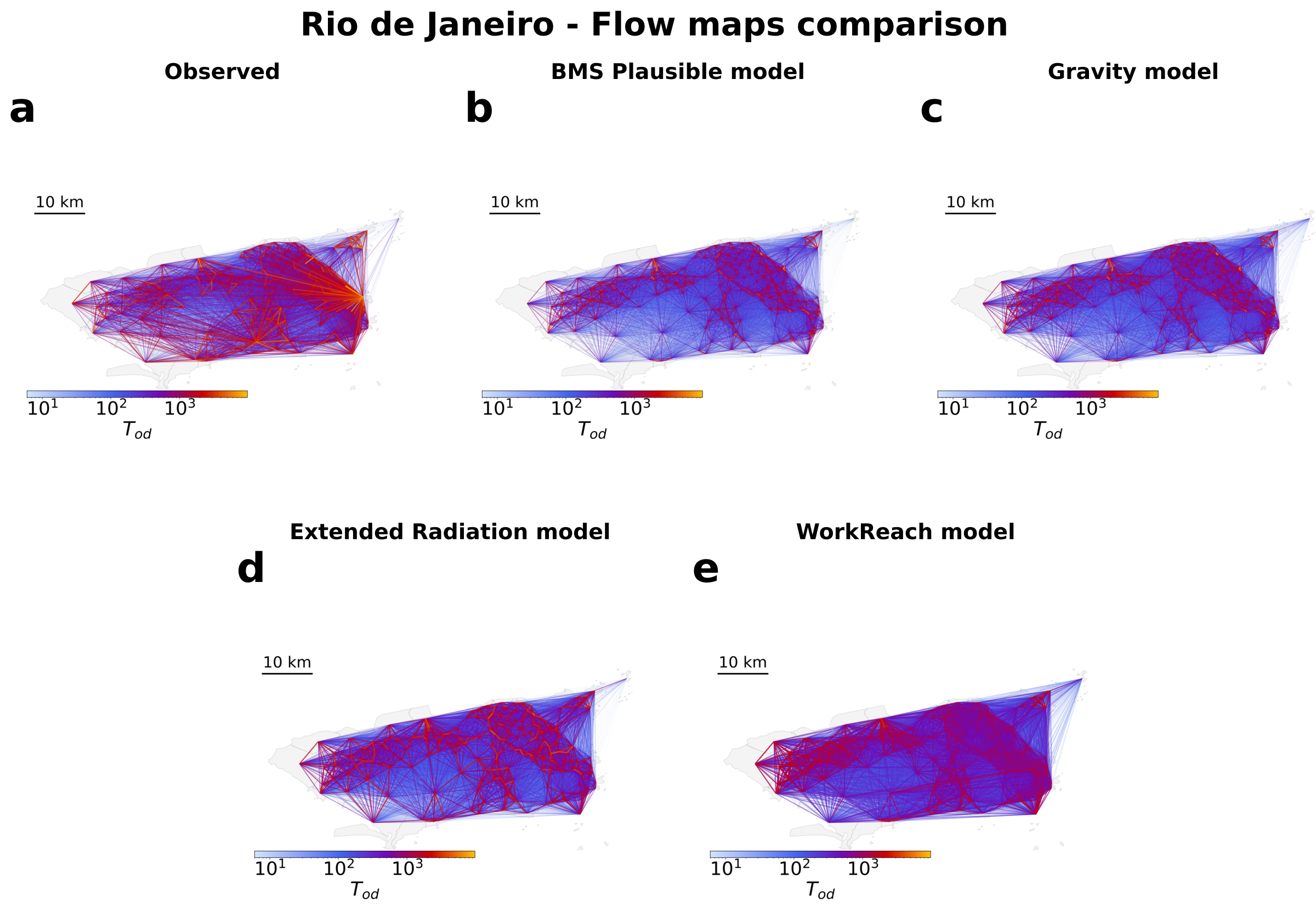

Supplementary Figure 19: Commuter flows for Rio de Janeiro. **a** Observed flows **b** BMS Plausible, **c** Gravity, **d** Extended Radiation, and **e** WorkReach. Each panel draws n = 39,402 origin-destination pairs between 199 zones. Line color encodes the number of commuters on a logarithmic scale, as given by the color bar in each panel.

Supplementary Note 7: Permutation Based Null Model

To test the added value of ECI^{emp} and informality rate as contributors to WorkReach's predictive power, we performed a permutation analysis. We compare the Common Part of Commuters (CPC) of the full model against versions where the spatial distribution of ECI^{emp}, informality rate, or both are randomly shuffled across zones, preserving the original marginal distributions. For each variant, the model is re-estimated on the shuffled inputs (1000 replications). Figures 20 and 21 show this comparison across variable bins, and Table 4 summarizes the overall results. In all four cities the full model significantly outperforms every randomized variant ($p < 0.001$), confirming that both variables contribute to predictive power independently and jointly.

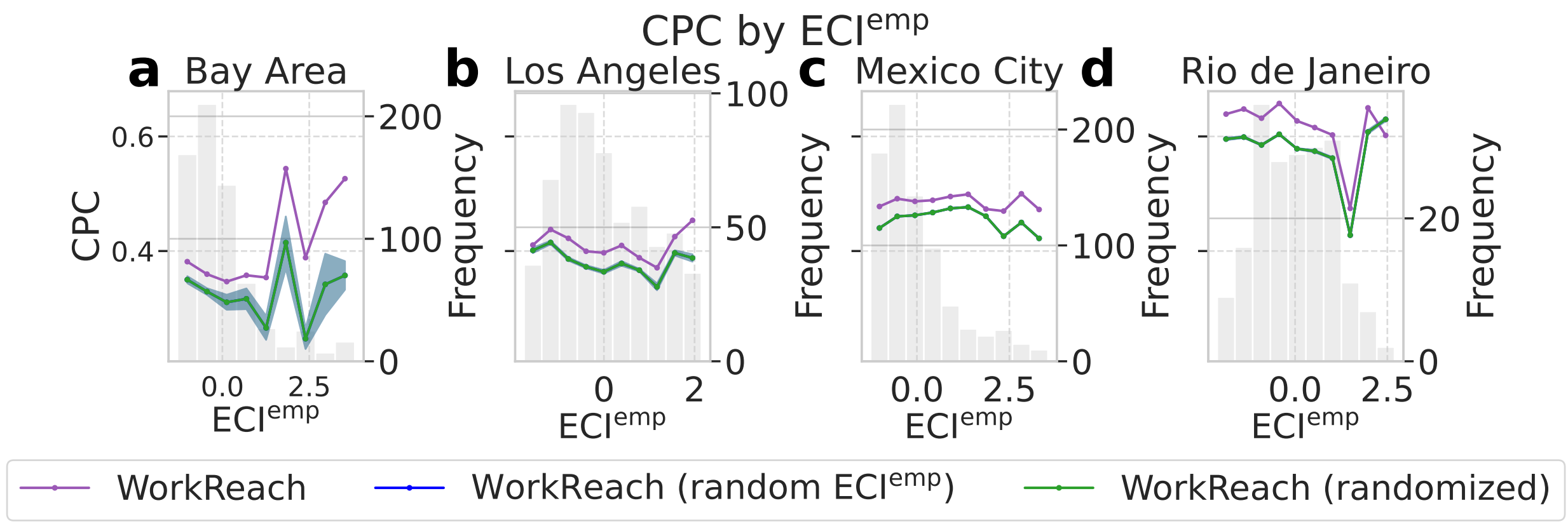

Supplementary Figure 20: Model performance (CPC) across bins of Economic Complexity Index (ECI^{emp}). The purple line shows the full WorkReach model; the blue line shows the model with randomized ECI^{emp} only; the green line shows the model with both ECI^{emp} and informality rate randomized. Shaded bands indicate ±1 standard deviation across n = 1,000 replications. Panels **a**–**d** correspond to the Bay Area, Los Angeles, Mexico City and Rio de Janeiro, respectively.

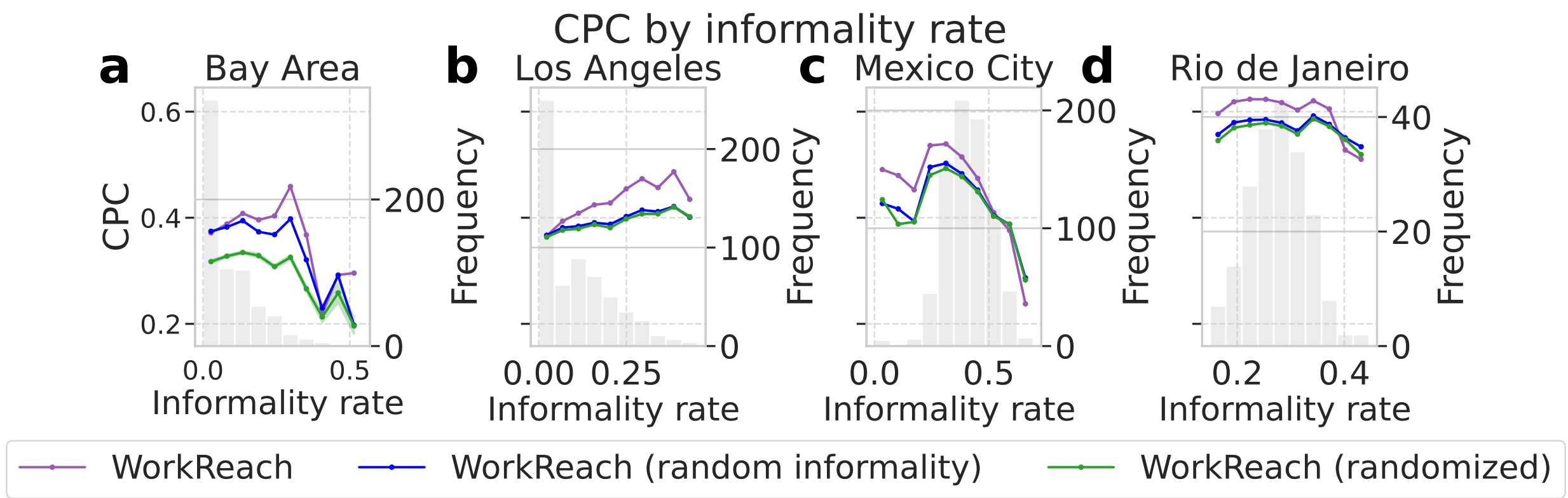

Supplementary Figure 21: Model performance (CPC) across bins of informality rate. The purple line shows the full WorkReach model; the blue line shows the model with randomized informality rate only; the green line shows the model with both ECI^{emp} and informality rate randomized. Shaded bands indicate ±1 standard deviation across n = 1,000 replications. Panels **a**–**d** correspond to the Bay Area, Los Angeles, Mexico City and Rio de Janeiro, respectively.

Supplementary Table 4: Permutation analysis of model performance (CPC). Each variant shuffles the spatial distribution of the target variable across zones and re-estimates the model (1000 replications). Standard errors and one-sided permutation p-values test whether the full model significantly outperforms each null, with no correction for multiple comparisons. All $p < 0.001$.

**City**	**WorkReach**	**Random** $\mathrm{ECI}^{\mathrm{emp}}$	**Random Inf.**	**Random Both**	p-value
Bay Area	0.389	$0.330 \pm <0.001$	$0.363 \pm <0.001$	$0.330 \pm <0.001$	< 0.001
Los Angeles	0.413	$0.379 \pm <0.001$	$0.394 \pm <0.001$	$0.379 \pm <0.001$	< 0.001
Mexico City	0.486	$0.457 \pm <0.001$	$0.461 \pm <0.001$	$0.457 \pm <0.001$	< 0.001
Rio de Janeiro	0.614	$0.572 \pm <0.001$	$0.578 \pm <0.001$	$0.572 \pm <0.001$	< 0.001

Supplementary Note 8: Prediction Error Correlation Analysis

To understand whether the four models capture distinct or overlapping aspects of commuting behavior, we computed pairwise Pearson correlations between prediction residuals (observed minus predicted flows) for all models and cities. Supplementary Fig. 22 displays the residual correlation matrices.

The most striking pattern is the near-perfect residual correlation between Gravity and BMS Plausible (r = 0.96–0.99 across all cities), consistent with the structural similarity of their formulations [2]. Both models rely on origin and destination populations weighted by distance decay, so they succeed and fail on essentially the same OD pairs. WorkReach's residual correlations with the benchmarks vary across cities: highest in Los Angeles (r = 0.92–0.95) and lowest in the Bay Area (r = 0.86–0.92) and Mexico City (r = 0.66–0.86), indicating that the inclusion of $\mathrm{ECI}^{\mathrm{emp}}$ and informality captures distinct predictive information in these cities. The Extended Radiation model diverges most from all other models, particularly in Mexico City ($r = 0.66$ with WorkReach, $r = 0.77$ with Gravity) and Los Angeles ($r = 0.73$ with WorkReach, $r = 0.80$ with Gravity), likely reflecting the radiation model's reliance on intervening opportunities rather than parametric distance decay.

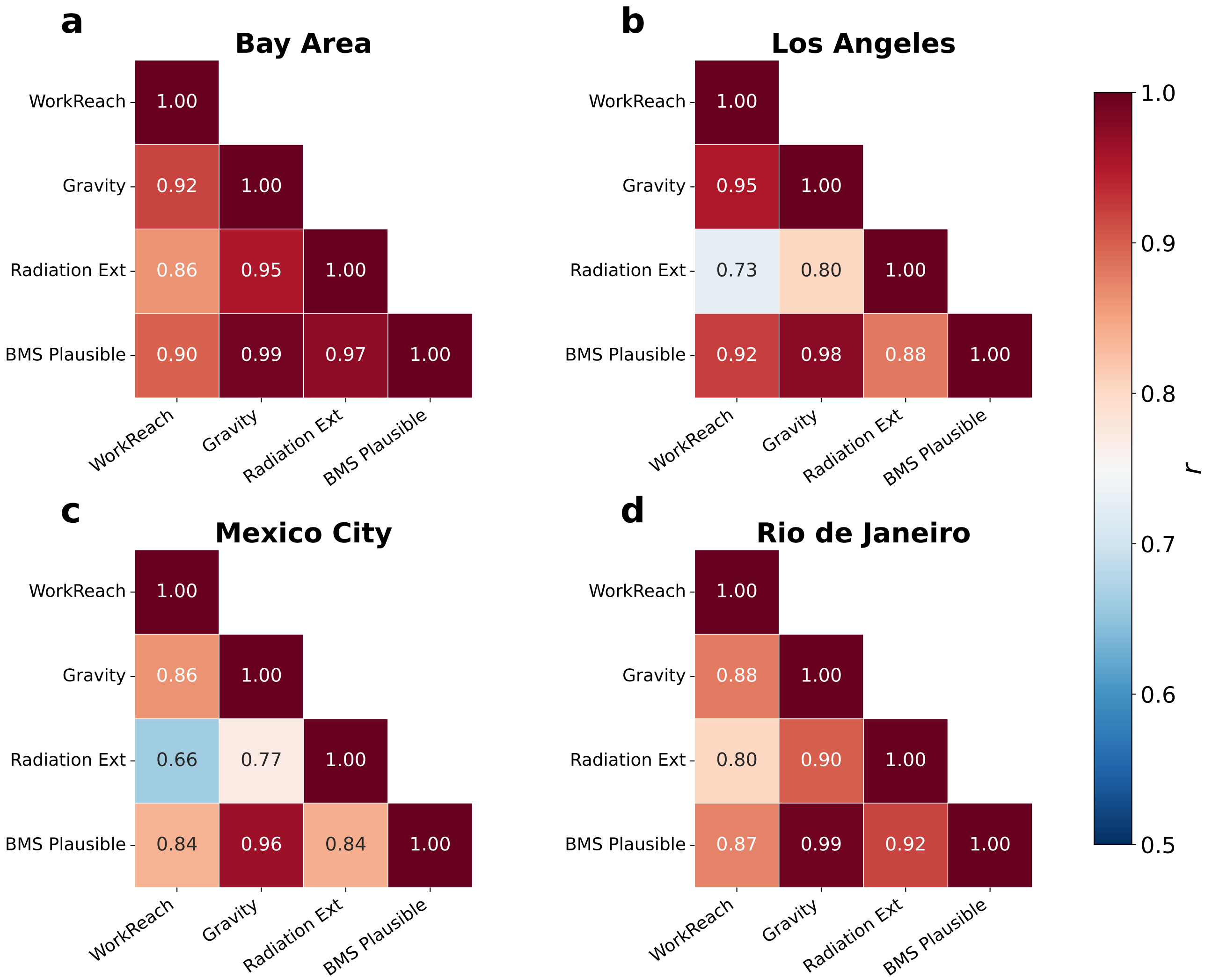

Supplementary Figure 22: Pairwise Pearson correlations between prediction residuals (observed − predicted) for all four models, by city. Gravity and BMS Plausible share near-identical residual structure ($r > 0.96$). The Extended Radiation model is the most distinct, particularly in Mexico City and Los Angeles. Panels **a**–**d** correspond to the Bay Area, Los Angeles, Mexico City and Rio de Janeiro, respectively. Correlations are computed over n = 457,652, 365,420, 628,056 and 39,402 origin-destination pairs for the Bay Area, Los Angeles, Mexico City and Rio de Janeiro respectively, excluding self-flows. Entries are Pearson correlations between the residual vectors of each pair of models, from two-sided tests with $n - 2$ degrees of freedom and no correction for multiple comparisons.

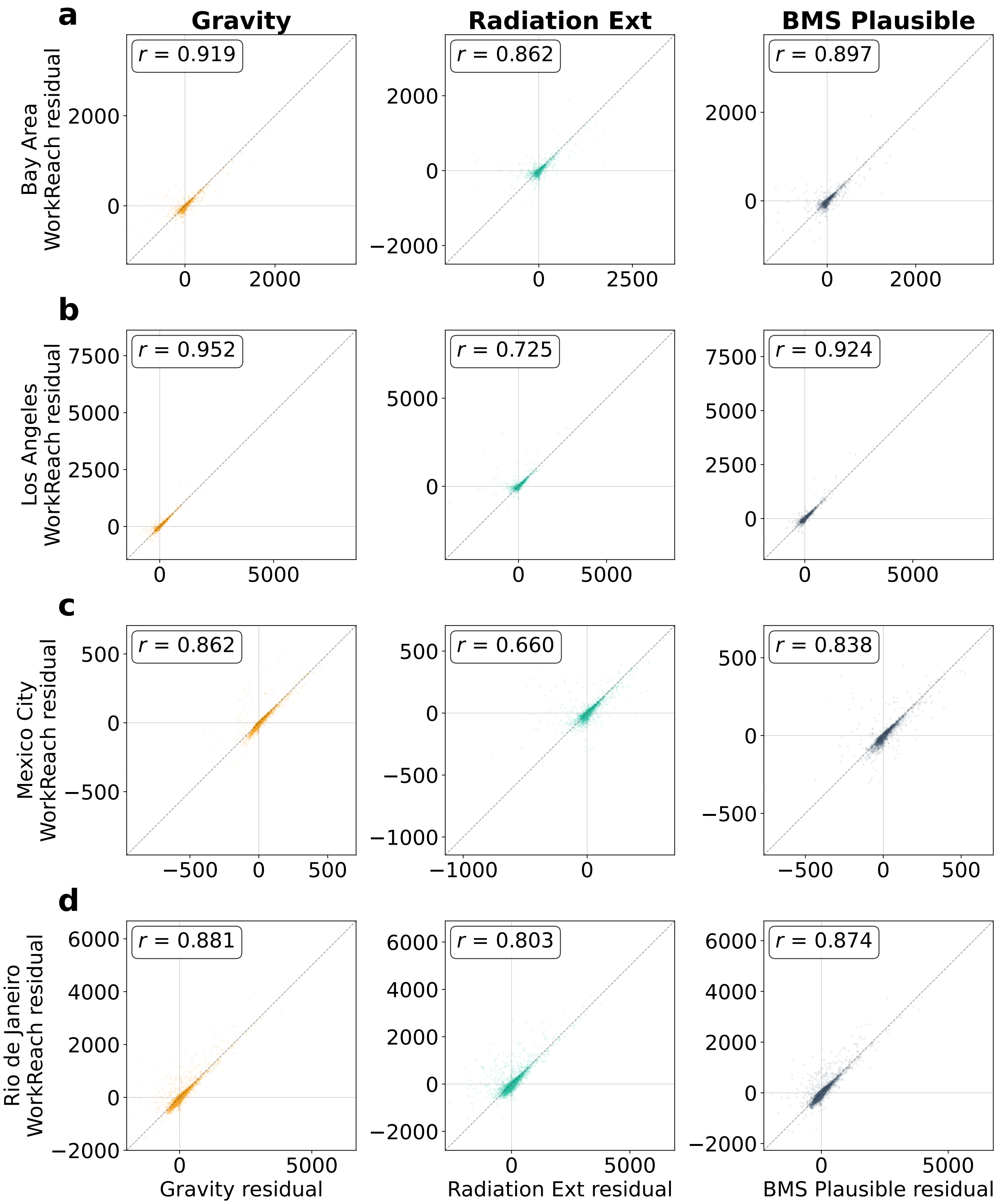

Supplementary Figure 23: WorkReach prediction residuals versus benchmark model residuals for each city (rows) and benchmark (columns). Points near the diagonal indicate shared prediction errors; deviations reveal OD pairs where WorkReach predicts differently from the benchmark. Pearson correlations are annotated in each panel. Rows **a**–**d** correspond to the Bay Area, Los Angeles, Mexico City and Rio de Janeiro, respectively, and the columns to the Gravity, Extended Radiation and BMS Plausible benchmarks, in that order. Residuals are computed over n = 457,652, 365,420, 628,056 and 39,402 origin-destination pairs for the Bay Area, Los Angeles, Mexico City and Rio de Janeiro respectively, excluding self-flows.

Supplementary Note 9: Out-of-Sample Cross-Validation

To assess whether the reported performance metrics reflect genuine predictive ability rather than overfitting, we conducted a stratified 5-fold cross-validation on origin zones. In each fold, 80% of origin zones are used for training and the remaining 20% are held out for evaluation. The split is stratified by quartile bins of zone population, $\mathrm{ECI}^{\mathrm{emp}}$, and informality rate to ensure that both train and test sets span the full range of zone characteristics. Crucially, all destination zones remain in the choice set for both train and test origins: we withhold only the observed outflow rows of test origins during parameter estimation, preserving the multinomial logit structure in which every origin faces the same set of destination alternatives. Each model is re-estimated from scratch on the training origins; the fitted parameters are then used to predict destination shares for the held-out origins.

Because each origin zone appears in the test set in exactly one fold, stitching the test predictions across all five folds yields a complete out-of-sample prediction for every origin–destination pair. Supplementary Table 5 reports the mean train and test CPC and Pearson r across folds; Supplementary Fig. 24 visualizes the comparison.

The train–test performance gap is negligible for all models and cities ($\Delta\mathrm{CPC} \leq 0.001$, $\Delta r \leq 0.02$). This confirms that the parsimonious parameterizations of all four models (1–6 free parameters on 40,000–630,000 OD pairs) are not susceptible to overfitting, and that the in-sample performance comparison reported in the main text accurately reflects out-of-sample predictive ability.

Supplementary Table 5: Mean train and test performance across 5 stratified folds (± standard deviation). The negligible train–test gap confirms the absence of overfitting across all models.

		CPC		Pearson r	
City	Model	Train	Test	Train	Test
Bay Area	WorkReach	0.389 ± 0.001	0.389 ± 0.007	0.524 ± 0.013	0.524 ± 0.029
	Gravity	0.347 ± 0.001	0.347 ± 0.002	0.461 ± 0.014	0.454 ± 0.045
	Ext. Radiation	0.335 ± 0.001	0.335 ± 0.003	0.413 ± 0.003	0.415 ± 0.013
	BMS Plausible	0.342 ± 0.001	0.342 ± 0.003	0.468 ± 0.017	0.460 ± 0.050
Los Angeles	WorkReach	0.413 ± 0.002	0.413 ± 0.006	0.580 ± 0.015	0.580 ± 0.044
	Gravity	0.418 ± 0.002	0.417 ± 0.006	0.595 ± 0.015	0.581 ± 0.046
	Ext. Radiation	0.406 ± 0.001	0.406 ± 0.006	0.530 ± 0.011	0.522 ± 0.043
	BMS Plausible	0.414 ± 0.002	0.413 ± 0.007	0.606 ± 0.018	0.587 ± 0.056
Mexico City	WorkReach	0.486 ± 0.002	0.486 ± 0.005	0.658 ± 0.003	0.658 ± 0.009
	Gravity	0.488 ± 0.002	0.488 ± 0.006	0.661 ± 0.010	0.664 ± 0.035
	Ext. Radiation	0.462 ± 0.002	0.461 ± 0.006	0.613 ± 0.003	0.613 ± 0.013
	BMS Plausible	0.474 ± 0.002	0.473 ± 0.007	0.646 ± 0.010	0.649 ± 0.038
Rio de Janeiro	WorkReach	0.614 ± 0.002	0.614 ± 0.003	0.677 ± 0.005	0.677 ± 0.015
	Gravity	0.639 ± 0.002	0.639 ± 0.005	0.694 ± 0.008	0.694 ± 0.033
	Ext. Radiation	0.644 ± 0.001	0.644 ± 0.005	0.707 ± 0.005	0.707 ± 0.018
	BMS Plausible	0.641 ± 0.001	0.641 ± 0.004	0.697 ± 0.008	0.697 ± 0.033

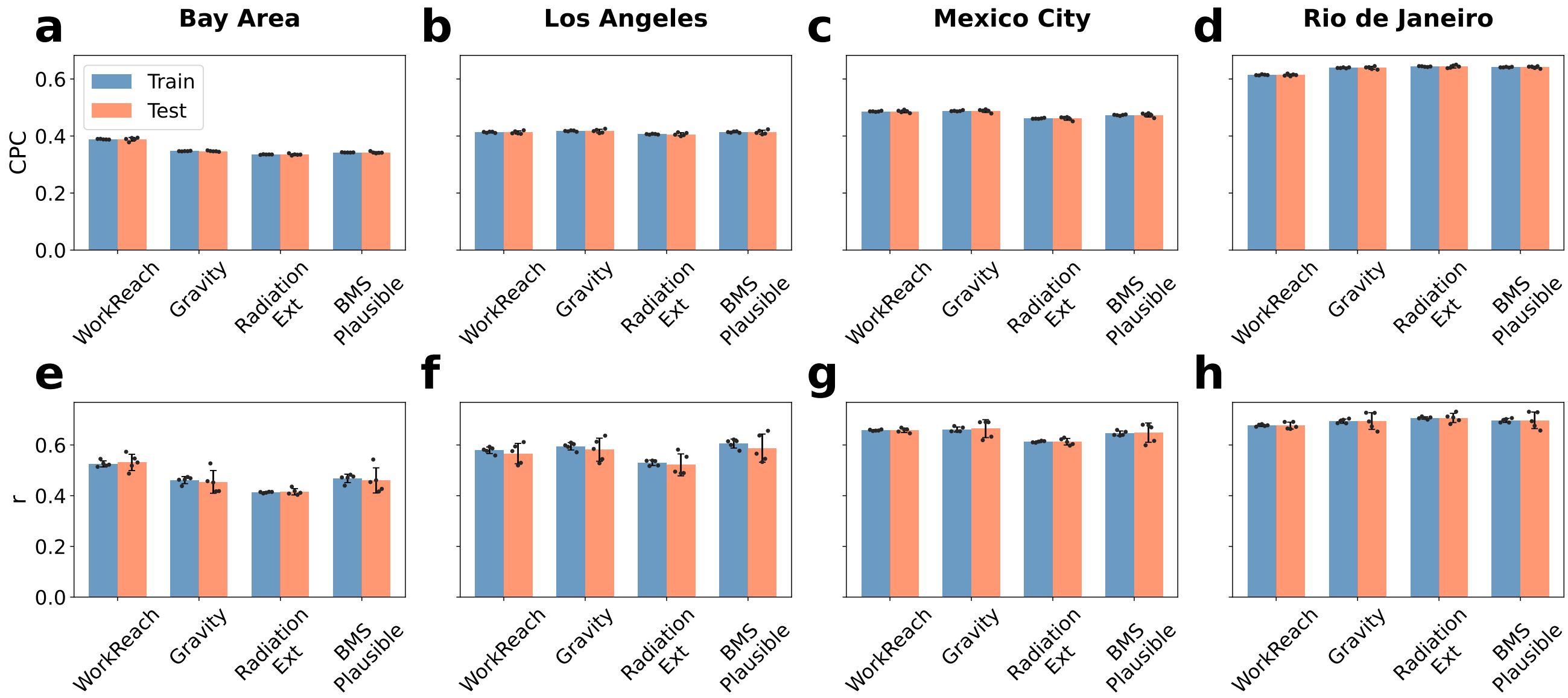

Supplementary Figure 24: Train (blue) versus test (coral) predictive performance across n = 5 stratified folds for all four models and cities. Error bars show ±1 standard deviation across folds and the individual folds are overlaid as points. The negligible gap between train and test confirms that none of the models overfit. Panels **a**–**d** show CPC for the Bay Area, Los Angeles, Mexico City and Rio de Janeiro; panels **e**–**h** show Pearson r for the same four cities in the same order.

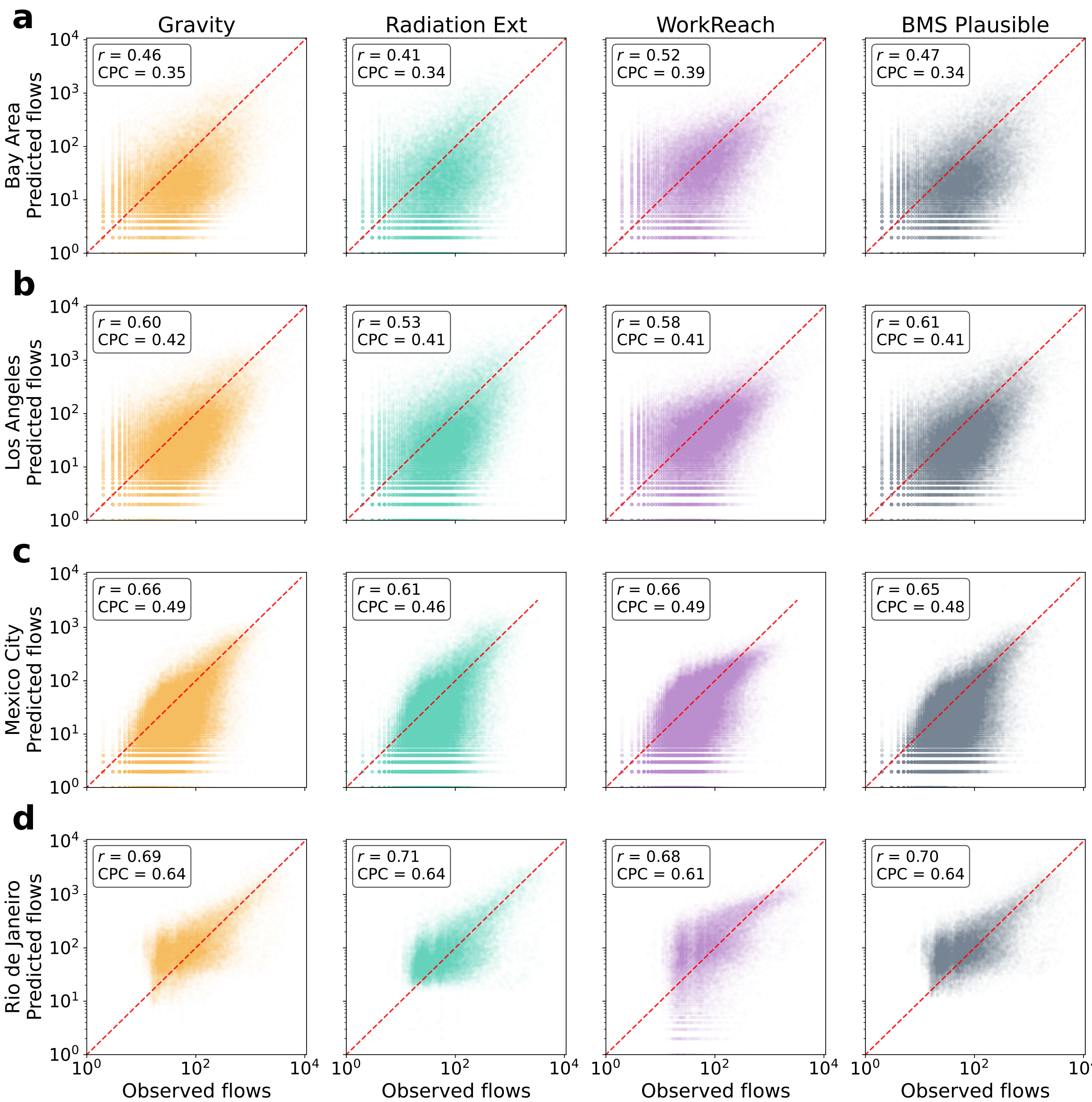

Supplementary Figure 25: Out-of-sample observed versus predicted flows (log–log scale) for all four models and cities. Each prediction is fully out of sample: origin zones were stitched across the five cross-validation folds so that every origin appears in the test set exactly once. CPC and Pearson r values are comparable to the full-data estimates reported in the main text (Figure 4), confirming that the in-sample results are not driven by overfitting. Rows **a**–**d** correspond to the Bay Area, Los Angeles, Mexico City and Rio de Janeiro, respectively, and the columns to the Gravity, Extended Radiation, WorkReach and BMS Plausible models, in that order. Each panel draws n = 457,652, 365,420, 628,056 and 39,402 origin-destination pairs for the Bay Area, Los Angeles, Mexico City and Rio de Janeiro respectively.

Supplementary Note 10: Robustness of the Economic Complexity Index

A central claim of WorkReach is that $\mathrm{ECI}^{\mathrm{emp}}$ captures a meaningful dimension of destination quality not reducible to simpler alternatives. This section examines that claim from five complementary angles. We first verify that $\mathrm{ECI}^{\mathrm{emp}}$ and the model's other covariate, residential informality, are not proxies for the same underlying variable (Supplementary Note 11). We then confirm that the zone–sector binary matrices underlying the $\mathrm{ECI}^{\mathrm{emp}}$ computation exhibit the nested structure required for reliable complexity rankings (Supplementary Note 12). We also characterize the product-space network that underlies the $\mathrm{PCI}^{\mathrm{emp}}$ and proximity calculations, documenting meaningful cross-city differences in co-location density (Supplementary Note 13). Next, we show that $\mathrm{ECI}^{\mathrm{emp}}$ retains predictive power even after removing all variation explained by its two raw ingredients, diversity and mean ubiquity (Supplementary Note 14). Finally, we compare $\mathrm{ECI}^{\mathrm{emp}}$ against income and wealth proxies across all four cities and find that both carry complementary information about destination attractiveness (Supplementary Note 15).

Supplementary Note 11: $\mathrm{ECI}^{\mathrm{emp}}$ and Informality as Complementary Dimensions

The WorkReach model enters $\mathrm{ECI}^{\mathrm{emp}}$ as a destination attribute and informality as an origin attribute, assigning them structurally different roles in the utility function. To verify that these two variables are not redundant, we examine their cross-zone correlation, which quantifies the degree to which they capture overlapping spatial variation. For each city, every H3 zone (or AEP zone for Rio de Janeiro) carries both an $\mathrm{ECI}^{\mathrm{emp}}$ value derived from its employment structure and a residential informality rate derived from census data. Supplementary Fig. 26 and Supplementary Table 6 report this correlation across all four cities.

At the zone level, the Pearson correlations for the Bay Area and Los Angeles are $r = 0.028$ and $r = 0.008$ respectively (both $p > 0.4$), indicating that $\mathrm{ECI}^{\mathrm{emp}}$ and residential informality are essentially uncorrelated in these labor markets. High-$\mathrm{ECI}^{\mathrm{emp}}$ employment zones are found across the full informality spectrum of residential zones; no systematic spatial alignment exists between productive complexity and informality in these cities. In Mexico City and Rio de Janeiro, the zone-level correlations are $r = -0.402$ ($p < 0.001$) and $r = -0.404$ ($p < 0.001$) respectively. The negative sign is consistent with a spatial structure in which central zones have both higher employment complexity and lower residential informality, while peripheral zones have lower complexity and higher informality. However, the moderate magnitude ($|r| < 0.45$, $R^2 \leq 0.17$) indicates that each variable accounts for at most 17% of the variance in the other.

Because the model pairs origin informality with destination $\mathrm{ECI}^{\mathrm{emp}}$, we also compute the flow-weighted Pearson correlation between home-zone informality and work-zone $\mathrm{ECI}^{\mathrm{emp}}$ across all observed origin-destination pairs (Supplementary Table 6). In the U.S. cities the flow-weighted correlations are near zero ($r = -0.012$, $p = 0.394$ for the Bay Area; $r = 0.023$, $p = 0.013$ for Los Angeles, where 81,515 pairs make even a negligible correlation detectable). In Mexico City and Rio de Janeiro they are $r = -0.172$ and $r = -0.202$ respectively (both $p < 0.001$, permutation test), substantially weaker than the zone-level values. The two variables as they actually enter the model are thus only weakly associated, with $R^2 \leq 0.05$ even in the Latin American cities.

Supplementary Table 6: Cross-correlation between $\mathrm{ECI}^{\mathrm{emp}}$ and residential informality rate. The first two columns report the zone-level Pearson correlation (each zone carries both an $\mathrm{ECI}^{\mathrm{emp}}$ value and an informality rate). The last column reports the flow-weighted Pearson correlation computed across all observed origin-destination pairs, where origin informality and destination $\mathrm{ECI}^{\mathrm{emp}}$ are the two variables and commuting flows are the weights. Zone-level p-values are from two-sided Pearson correlation tests and flow-weighted p-values from a two-sided permutation test with 2,000 permutations, with no correction for multiple comparisons. Zone-level $p = 0.463$ (Bay Area), 0.842 (Los Angeles), < 0.001 (Mexico City) and < 0.001 (Rio de Janeiro). Flow-weighted $p = 0.394$, 0.013, < 0.001 and < 0.001, respectively.

City	Zones (n)	Pearson r (zone)	p-value	Pearson r (flow-wtd)	p-value
Bay Area	677	0.028	0.463	-0.012	0.394
Los Angeles	605	0.008	0.842	0.023	0.013
Mexico City	793	-0.402	< 0.001	-0.172	< 0.001
Rio de Janeiro	199	-0.404	< 0.001	-0.202	< 0.001

Several mechanisms, which are not mutually exclusive, can account for why high-$\mathrm{ECI}^{\mathrm{emp}}$ destinations attract workers across the formality spectrum. High-$\mathrm{ECI}^{\mathrm{emp}}$ zones concentrate a broader and more diverse set of employment opportunities, so workers at all skill levels are more likely to find a suitable match, an effect akin to the labor-market pooling described in the agglomeration literature [3]. In addition, the employment data used to compute $\mathrm{ECI}^{\mathrm{emp}}$ in Latin American cities records formally registered establishments (DENUE

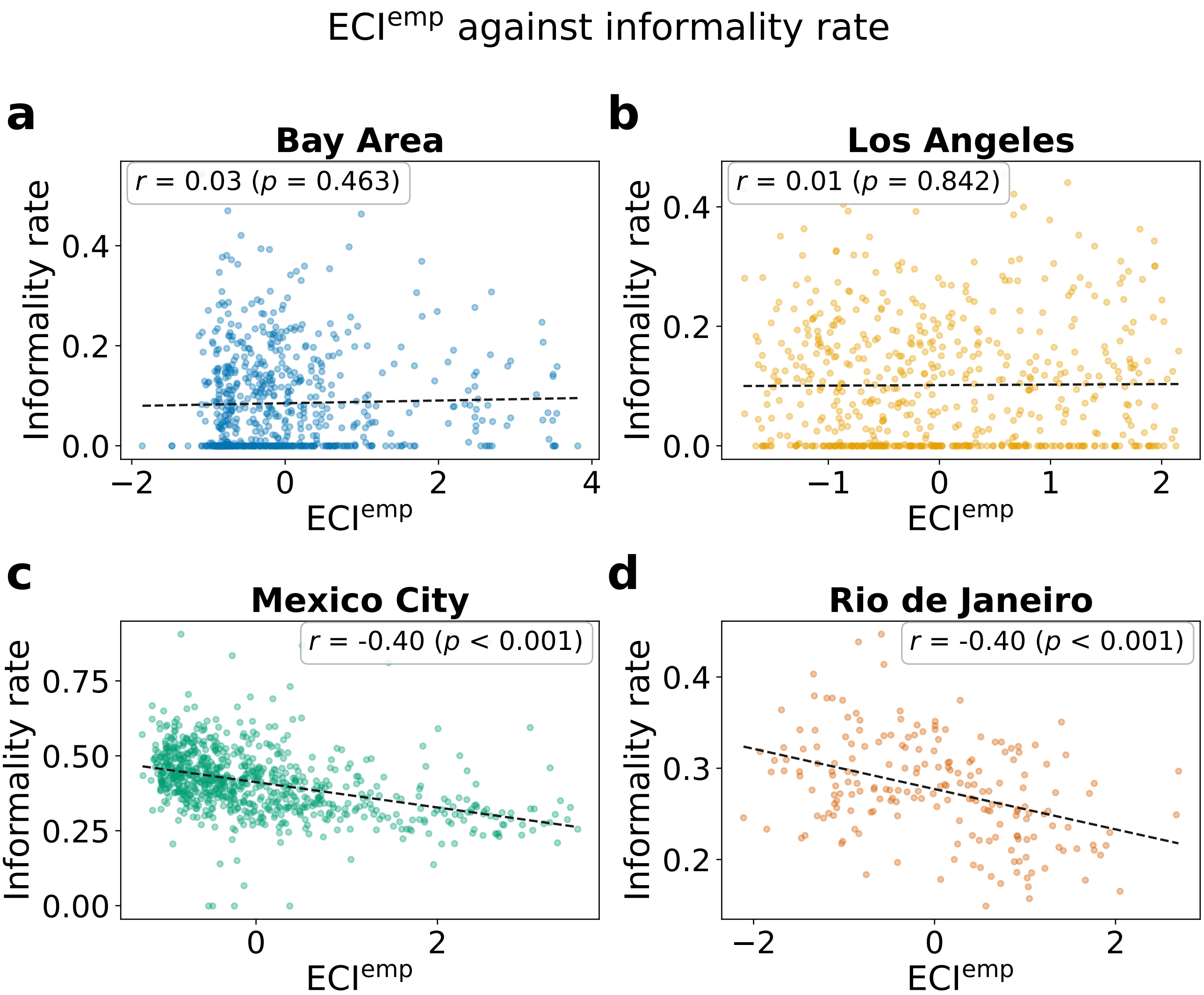

Supplementary Figure 26: Zone-level ECI^{emp} versus residential informality rate for each city. Dashed lines show OLS fits; Pearson r and p-values are reported. The correlation is near-zero in the U.S. cities and moderately negative in the Latin American cities. Panels **a**–**d** correspond to the Bay Area, Los Angeles, Mexico City and Rio de Janeiro, respectively. Correlations are computed over n = 677, 605, 793 and 199 zones for the Bay Area, Los Angeles, Mexico City and Rio de Janeiro respectively. All correlations are Pearson coefficients from two-sided tests with no correction for multiple comparisons; degrees of freedom are $n-2$ for the sample size given above.

in Mexico City; RAIS in Rio de Janeiro); employment in these zones therefore tends to carry statutory protections, including social security affiliation and health coverage, regardless of the worker's role. A worker employed in a non-specialized capacity at a formally registered firm, such as building maintenance, administrative support, or supply services, gains institutional protections that would be unavailable through informal employment near their residence. Finally, workers whose skills are not matched by local demand must cross greater distances to reach zones where that demand exists. Our aggregate flow data do not permit us to distinguish these channels, and all three are consistent with a positive β_{ECI} and a positive β_{inf} in Latin American cities.

Supplementary Note 12: Nested Structure of Zone–Sector Binary Matrices

A binary matrix is nested if the sector portfolio of less-diversified zones is a proper subset of the portfolio of more-diversified zones, a property that supports reliable ECI^{emp} estimation [4]. For each city we construct the zone–sector binary matrix $M_{zs} = \mathbf{1}[\text{RCA}_{zs} \geq 1]$ and sort rows by diversity (descending) and columns by ubiquity (descending). Supplementary Fig. 27 shows the resulting staircase pattern across all four cities.

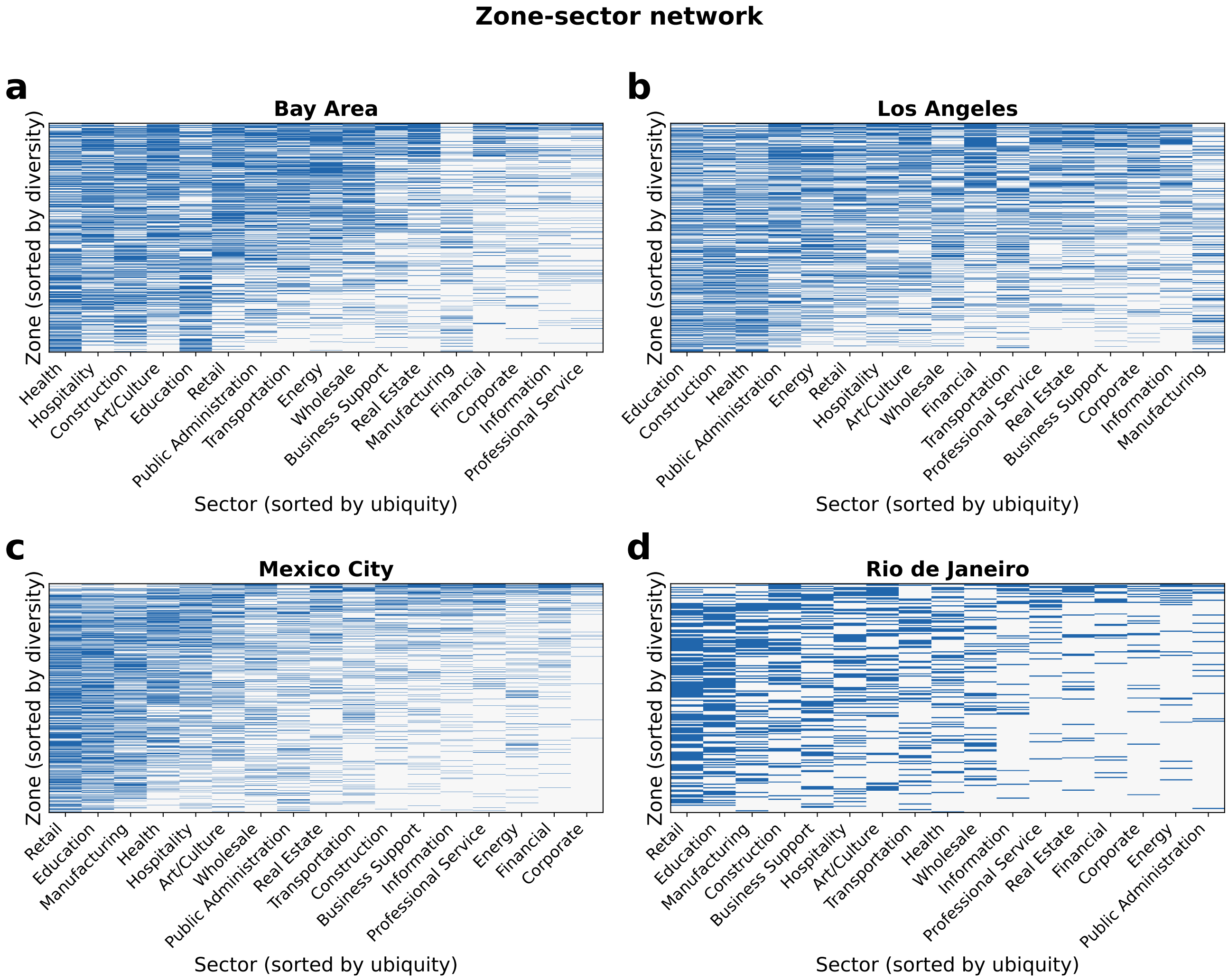

Supplementary Figure 27: Zone–sector binary RCA matrices for the four cities, sorted by zone diversity (rows, high to low) and sector ubiquity (columns, high to low). Blue cells indicate RCA ≥ 1; white cells RCA < 1. The staircase pattern is the signature of a nested structure. Panels **a**–**d** correspond to the Bay Area, Los Angeles, Mexico City and Rio de Janeiro, respectively. Matrices cover n = 677, 605, 793 and 199 zones for the Bay Area, Los Angeles, Mexico City and Rio de Janeiro respectively, each against the 17 NAICS sectors.

We quantify nestedness using the NODF metric (Nestedness based on Overlap and Decreasing Fill), which measures the extent to which the sectoral composition of less diversified zones forms subsets of more diversified ones. The observed score is compared against a null distribution obtained by randomizing the zone–sector matrix at the observed fill (Supplementary Table 7). In all four cities the observed NODF

substantially exceeds the null expectation ($p < 0.001$), confirming significant nestedness.

Supplementary Table 7: NODF nestedness scores for the four zone–sector binary matrices, compared to 999 matrices randomized at the observed fill. p-values are from a one-sided permutation test with no correction for multiple comparisons. All $p < 0.001$.

City	NODF (observed)	Null mean ± SD	p-value
Bay Area	47.7	38.1 ± 0.4	< 0.001
Los Angeles	44.0	40.0 ± 0.3	< 0.001
Mexico City	42.6	31.8 ± 0.4	< 0.001
Rio de Janeiro	42.1	32.2 ± 0.9	< 0.001

Supplementary Note 13: Product-Space Network Structure

The product-space proximity matrix $\mathbf{\Phi}$ quantifies the tendency of sectors to co-locate within the same zones: $\phi_{ss'} = \Pr(\mathrm{RCA}_{zs} \geq 1 \mid \mathrm{RCA}_{zs'} \geq 1)$, taken as the minimum of the two conditional probabilities. Because $\mathrm{PCI}^{\mathrm{emp}}$ and the sectoral relatedness score are both derived from this matrix, it is useful to characterize its structure across the four cities.

With 17 NAICS sectors the complete graph contains $\binom{17}{2} = 136$ possible edges. Following the approach of Hidalgo et al. [5], who used a maximum spanning tree (MST) supplemented by all edges above a proximity threshold to visualize the product space, we construct each city's network as the union of the MST and all edges with $\phi > \phi^*$, where $\phi^* = 0.30$ for the U.S. cities and $\phi^* = 0.20$ for the Latin American cities. The MST guarantees a single connected component, while the threshold controls network density. We chose a lower cutoff for Mexico City and Rio de Janeiro because their proximity matrices are sparser, reflecting sharper sectoral specialization (Supplementary Table 8).

Supplementary Table 8: Product-space network edge counts (and mean degree) under different filtering strategies. "MST only" retains the 16 edges of the maximum spanning tree. Each subsequent column shows the result of supplementing the MST with all edges above the indicated proximity threshold. The final column ($\phi > 0.20$ only) applies a fixed threshold without the MST. The maximum possible number of edges is 136 ($= \binom{17}{2}$). Bold values indicate the configuration used in the manuscript figures.

City	MST	$\phi > 0.30$	$\phi > 0.40$	$\phi > 0.50$	$\phi > 0.60$	$\phi > 0.20$ only
Bay Area	16	**62 (7.3)**	48 (5.6)	26 (3.1)	18 (2.1)	86 (10.1)
Los Angeles	16	**73 (8.6)**	42 (4.9)	27 (3.2)	20 (2.4)	106 (12.5)
Mexico City	16	30 (3.5)	20 (2.4)	16 (1.9)	16 (1.9)	**57 (6.7)**
Rio de Janeiro	16	33 (3.9)	17 (2.0)	16 (1.9)	16 (1.9)	**60 (7.1)**

The U.S. cities exhibit denser co-location structure: many sectors co-occur at comparable rates, yielding numerous edges above even a moderate threshold. The Latin American cities display sparser networks with fewer strong ties, indicating sharper sectoral specialization in which zones tend to concentrate employment in a smaller set of sectors. This asymmetry is consistent with the higher nestedness scores observed for the U.S. cities (Supplementary Table 7). Supplementary Fig. 28 visualizes the product-space network for each city, with nodes colored by $\mathrm{PCI}^{\mathrm{emp}}$ and sized by total sector employment.

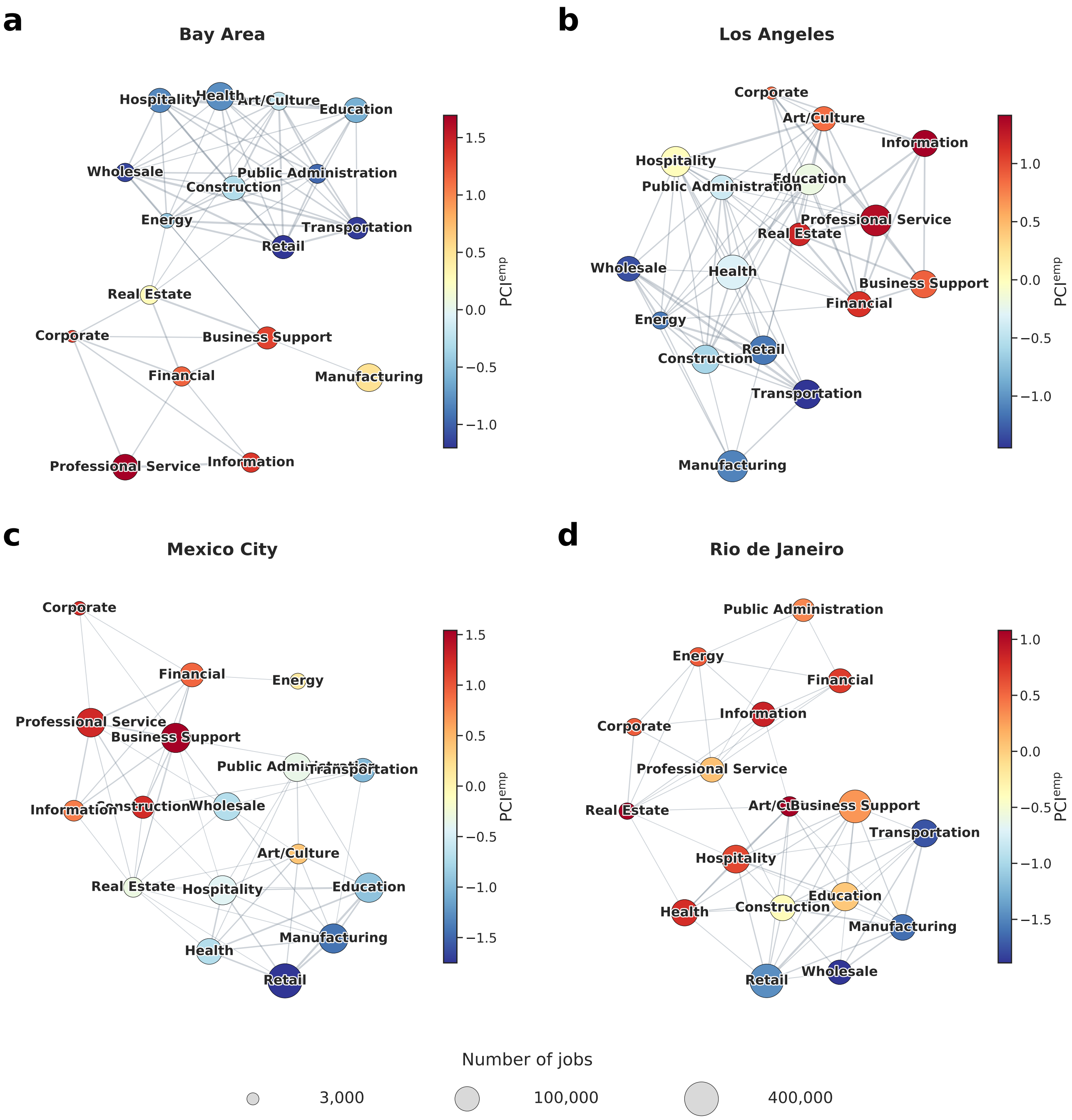

Supplementary Figure 28: Product-space networks for the four cities. Nodes represent NAICS sectors, colored by $\mathrm{PCI}^{\mathrm{emp}}$ and sized by total sector employment. Edge width is proportional to proximity ϕ. U.S. cities use MST + $\phi > 0.30$; Latin American cities use MST + $\phi > 0.20$. The denser networks in the U.S. cities reflect more homogeneous sector co-location patterns, while the sparser Latin American networks indicate sharper sectoral specialization. Panels **a**–**d** correspond to the Bay Area, Los Angeles, Mexico City and Rio de Janeiro, respectively. Each network contains n = 17 NAICS sectors as nodes.

Supplementary Note 14: $\mathrm{ECI}^{\mathrm{emp}}$ versus Diversity and Ubiquity

$\mathrm{ECI}^{\mathrm{emp}}$ is constructed from two zone-level quantities: industrial diversity (the number of sectors in which a zone holds a revealed comparative advantage) and sector ubiquity (how widespread those sectors are across zones). To test whether $\mathrm{ECI}^{\mathrm{emp}}$ carries information about destination attractiveness beyond what diversity and ubiquity alone provide, we decompose it into a component explained by these two inputs and an orthogonal residual, then evaluate whether the residual retains predictive power in the WorkReach model.

Definitions and Orthogonal Decomposition

Given the binary RCA matrix M_{zs}, we define for each zone z:

$$\mathrm{Diversity}_z = \sum_s M_{zs} \,, \tag{1}$$

$$\overline{\mathrm{Ubiquity}}_z = \frac{1}{\mathrm{Diversity}_z} \sum_s M_{zs} \cdot \left(\sum_{z'} M_{z's} \right) , \tag{2}$$

where $\overline{\mathrm{Ubiquity}}_z$ is the mean ubiquity of the sectors in which zone z has a comparative advantage. We then regress $\mathrm{ECI}^{\mathrm{emp}}$ on diversity and mean ubiquity via ordinary least squares:

$$\mathrm{ECI}_z^{\mathrm{emp}} = \alpha + \beta_D \,\mathrm{Diversity}_z + \beta_U \,\overline{\mathrm{Ubiquity}}_z + \varepsilon_z \,. \tag{3}$$

The residual ε_z, which we denote $\mathrm{ECI}_z^{\mathrm{employment},\perp}$, is orthogonal to both regressors by construction and isolates the information in $\mathrm{ECI}^{\mathrm{emp}}$ that cannot be recovered from diversity or mean ubiquity alone.

Supplementary Table 9 reports the R^2 of this regression for each city. The fraction of $\mathrm{ECI}^{\mathrm{emp}}$ variance explained by its two ingredients varies widely: from 90% in the Bay Area to 9% in Rio de Janeiro. In all cities, a non-trivial share of $\mathrm{ECI}^{\mathrm{emp}}$ variation is orthogonal to both components, confirming that the iterative reflections extract a joint signal that neither diversity nor mean ubiquity fully captures.

Supplementary Table 9: Variance in $\mathrm{ECI}^{\mathrm{emp}}$ explained by diversity and mean ubiquity (R^2 of OLS regression). The residual $\mathrm{ECI}^{\mathrm{employment},\perp}$ captures the remaining variation, which is orthogonal to both regressors by construction.

City	R^2
Bay Area	0.900
Los Angeles	0.219
Mexico City	0.757
Rio de Janeiro	0.090

Correlation between $\mathrm{ECI}^{\mathrm{emp}}$ and Its Components

Supplementary Fig. 29 shows $\mathrm{ECI}^{\mathrm{emp}}$ plotted against diversity (panels **a**–**d**) and against mean ubiquity (panels **e**–**h**) for all four cities. $\mathrm{ECI}^{\mathrm{emp}}$ is positively correlated with diversity (r between 0.18 and 0.49) and negatively correlated with mean ubiquity (r between -0.29 and -0.95), but the strength varies across cities. In the Bay Area and Mexico City, mean ubiquity is a strong predictor of $\mathrm{ECI}^{\mathrm{emp}}$ ($r = -0.95$ and -0.87), whereas in Los Angeles and Rio de Janeiro the relationship is weaker ($r = -0.46$ and -0.29). Diversity shows moderate to weak correlations in all cities.

Predictive Performance of $\mathrm{ECI}^{\mathrm{emp}}$ and Its Components

To test whether diversity or ubiquity alone can substitute for $\mathrm{ECI}^{\mathrm{emp}}$ in the WorkReach model, we estimate four specifications per city: the original WorkReach with $\mathrm{ECI}^{\mathrm{emp}}$, a diversity-only variant, a ubiquity-only variant, and a variant using the orthogonal residual $\mathrm{ECI}^{\mathrm{employment},\perp}$. Supplementary Table 10 reports CPC and r with bootstrap 95% confidence intervals.

Ubiquity alone yields higher CPC and r than $\mathrm{ECI}^{\mathrm{emp}}$ in all four cities, and diversity outperforms $\mathrm{ECI}^{\mathrm{emp}}$ in Mexico City and Rio de Janeiro. Because $\mathrm{ECI}^{\mathrm{emp}}$ combines diversity and ubiquity into a single index, a model that uses one component directly has more flexibility to fit city-specific patterns. Notably, even the orthogonal residual $\mathrm{ECI}^{\mathrm{employment},\perp}$, which by construction contains no information from diversity or

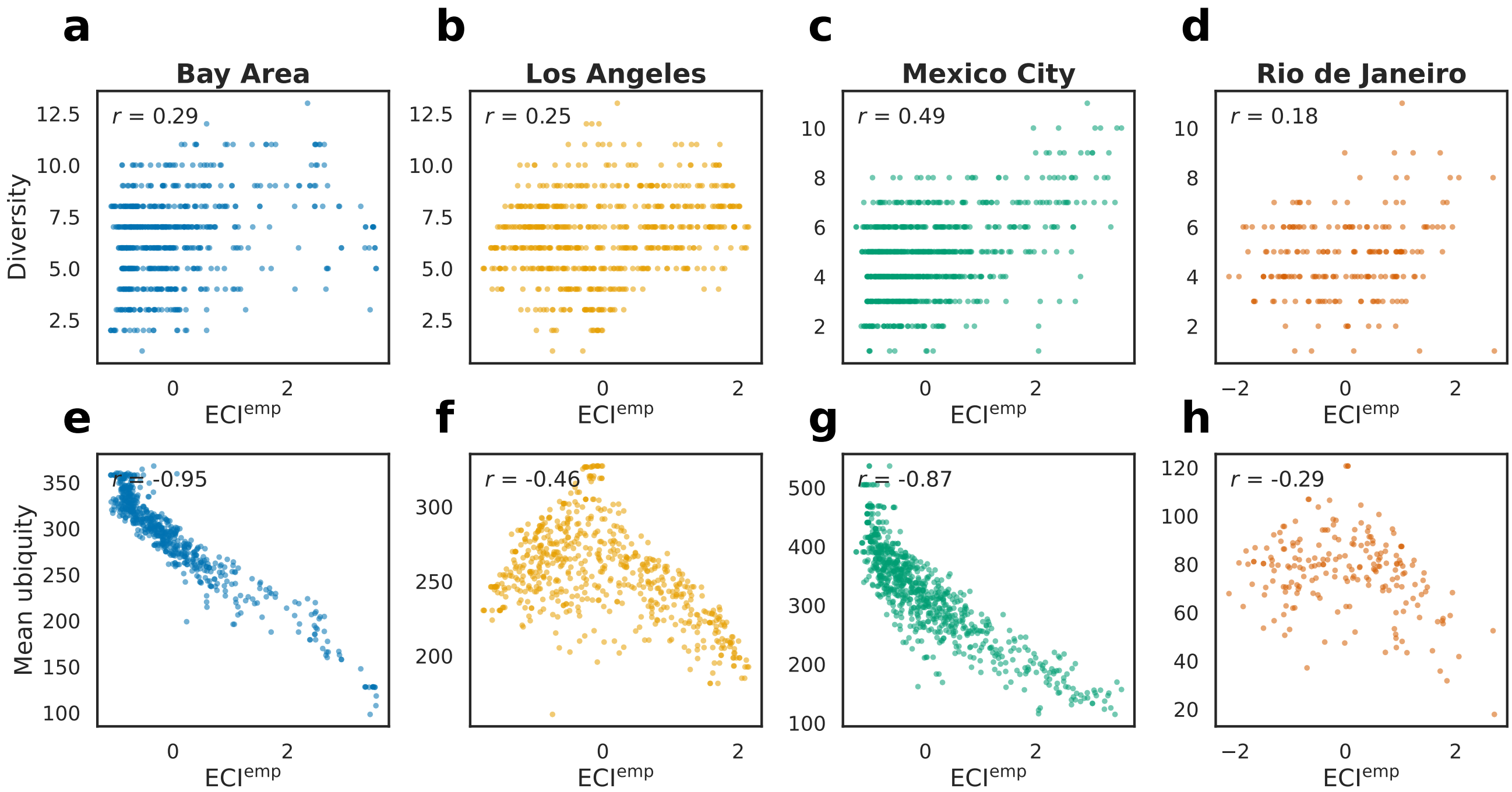

Supplementary Figure 29: Zone-level ECI^{emp} versus diversity (**a**–**d**) and mean ubiquity (**e**–**h**) for the four cities. Pearson correlation coefficients are shown for each city. Panels **a** and **e** correspond to the Bay Area, **b** and **f** to Los Angeles, **c** and **g** to Mexico City, and **d** and **h** to Rio de Janeiro. Correlations are computed over n = 677, 605, 793 and 199 zones for the Bay Area, Los Angeles, Mexico City and Rio de Janeiro respectively. All correlations are Pearson coefficients from two-sided tests with no correction for multiple comparisons; degrees of freedom are $n-2$ for the sample size given above.

Supplementary Table 10: WorkReach model performance using ECI^{emp}, diversity alone, mean ubiquity alone, or the orthogonal residual $ECI^{employment,\perp}$ as the destination attribute. Bootstrap 95% confidence intervals (1,000 replicates) are shown in brackets.

City	Model	CPC [95% CI]	r [95% CI]
Bay Area	WorkReach	0.389 [0.381, 0.397]	0.524 [0.487, 0.560]
	Diversity	0.363 [0.355, 0.370]	0.470 [0.454, 0.493]
	Ubiquity	0.392 [0.384, 0.400]	0.514 [0.493, 0.536]
	$ECI^{employment,\perp}$	0.367 [0.359, 0.375]	0.486 [0.459, 0.509]
Los Angeles	WorkReach	0.413 [0.405, 0.420]	0.580 [0.539, 0.612]
	Diversity	0.412 [0.404, 0.420]	0.574 [0.546, 0.593]
	Ubiquity	0.430 [0.422, 0.437]	0.601 [0.573, 0.619]
	$ECI^{employment,\perp}$	0.412 [0.404, 0.419]	0.569 [0.539, 0.590]
Mexico City	WorkReach	0.486 [0.480, 0.493]	0.658 [0.648, 0.667]
	Diversity	0.486 [0.480, 0.492]	0.661 [0.651, 0.670]
	Ubiquity	0.496 [0.489, 0.502]	0.682 [0.671, 0.693]
	$ECI^{employment,\perp}$	0.483 [0.477, 0.490]	0.661 [0.651, 0.670]
Rio de Janeiro	WorkReach	0.614 [0.606, 0.621]	0.677 [0.659, 0.693]
	Diversity	0.623 [0.616, 0.629]	0.690 [0.673, 0.706]
	Ubiquity	0.654 [0.646, 0.661]	0.735 [0.719, 0.749]
	$ECI^{employment,\perp}$	0.609 [0.601, 0.616]	0.670 [0.652, 0.687]

mean ubiquity, substantially outperforms the randomized-ECI^{emp} null from the permutation analysis (Supplementary Table 4): for example, CPC of 0.367 versus 0.330 in the Bay Area and 0.609 versus 0.572 in Rio de Janeiro. This confirms that ECI^{emp} encodes information about destination attractiveness beyond what its two constituent ingredients provide. The advantage of ECI^{emp} is not strictly predictive but structural: it is embedded in the broader economic complexity framework, which provides the feasibility and opportunity score needed to identify which new sectors a zone could realistically develop and to simulate the resulting change in commuting accessibility (Supplementary Note 19). Neither diversity nor ubiquity alone supports this type of counterfactual analysis.

Supplementary Note 15: ECI^{emp} versus Income and Wealth Proxies

ECI^{emp} captures the sophistication of a zone's employment structure, which may overlap with local income or wealth levels. To test whether it adds predictive value beyond a direct economic proxy, we estimated three model variants for each city: the original WorkReach specification (ECI^{emp} only), a wage-only specification replacing ECI^{emp} with a measure of destination economic status, and a combined model including both. Because direct wage data at the work location are unavailable for the Latin American cities, we construct city-specific proxies described below.

Income and Wealth Data Sources

Bay Area and Los Angeles. Wage data come from the Replica synthetic employment dataset. We aggregate individual incomes at the work location to zone-level means.

Rio de Janeiro. Individual-level wage records are not available. Instead, we use residential household income from the 2020 Brazilian Census at the zone level (in BRL per month). Because census income is measured at the *residential* location, direct assignment to destination zones would mix residential and employment characteristics. We therefore aggregate income to the employment location using the observed commuting flows as weights:

$$\bar{y}_j = \frac{\sum_i T_{ij}\, y_i}{\sum_i T_{ij}}\,, \tag{4}$$

where y_i is the residential-zone mean household income and T_{ij} is the commuting flow from zone i to zone j. This yields a flow-weighted income proxy for each destination zone, reflecting the income distribution of workers who actually commute there.

Mexico City. No equivalent income variable with broad spatial coverage is available for Mexico City. We construct a composite wealth index from the 2020 Mexican Population and Housing Census at the AGEB (census tract) level, following previous work [6]. The index is based on a principal component analysis (PCA) of sixteen household-level indicators: access to internet, motor vehicle, computer, microwave, washing machine, cable television, streaming services, videogame consoles, and mobile phone; share of dwellings with three or more rooms; access to a water storage tank; absence of dirt floors; absence of overcrowding; and an overall access-to-information-devices composite. The first principal component explains the largest share of variance across these dimensions and is converted to an index (0–1). The resulting residential wealth index is then aggregated to destination zones using the same flow-weighting formula as for Rio de Janeiro.

ECI^{emp} and Income/Wealth Correlation

Supplementary Fig. 30 shows the zone-level relationship between ECI^{emp} and the respective income or wealth proxy across all four cities. ECI^{emp} and destination economic status are positively correlated in every city (Pearson $r = 0.54$ for the Bay Area, $r = 0.60$ for Los Angeles, $r = 0.59$ for Mexico City, and $r = 0.59$ for Rio de Janeiro; all $p < 0.001$). The correlation is moderate rather than near-perfect, indicating that ECI^{emp} and wages or wealth capture overlapping but distinct dimensions of destination quality.

Model Comparison

Supplementary Table 11 reports predictive performance with block-bootstrap 95% confidence intervals for all four cities using mean income or wealth as the destination proxy. For Rio de Janeiro and Mexico City the proxy is the flow-weighted mean of the residential income or wealth variable defined above.

Likelihood-ratio (LR) tests confirm that each variable contributes independently when added to the other (Supplementary Table 12). In every city and both test directions, the improvement in log-likelihood is highly significant ($p < 0.001$), indicating that ECI^{emp} and the income or wealth proxy carry complementary information about destination attractiveness.

In the Bay Area, ECI^{emp} outperforms the wage proxy (ΔCPC = +0.012; Δr = +0.040), while in Los Angeles the two are essentially tied (ΔCPC = +0.001; Δr = +0.008). In the Latin American cities, the

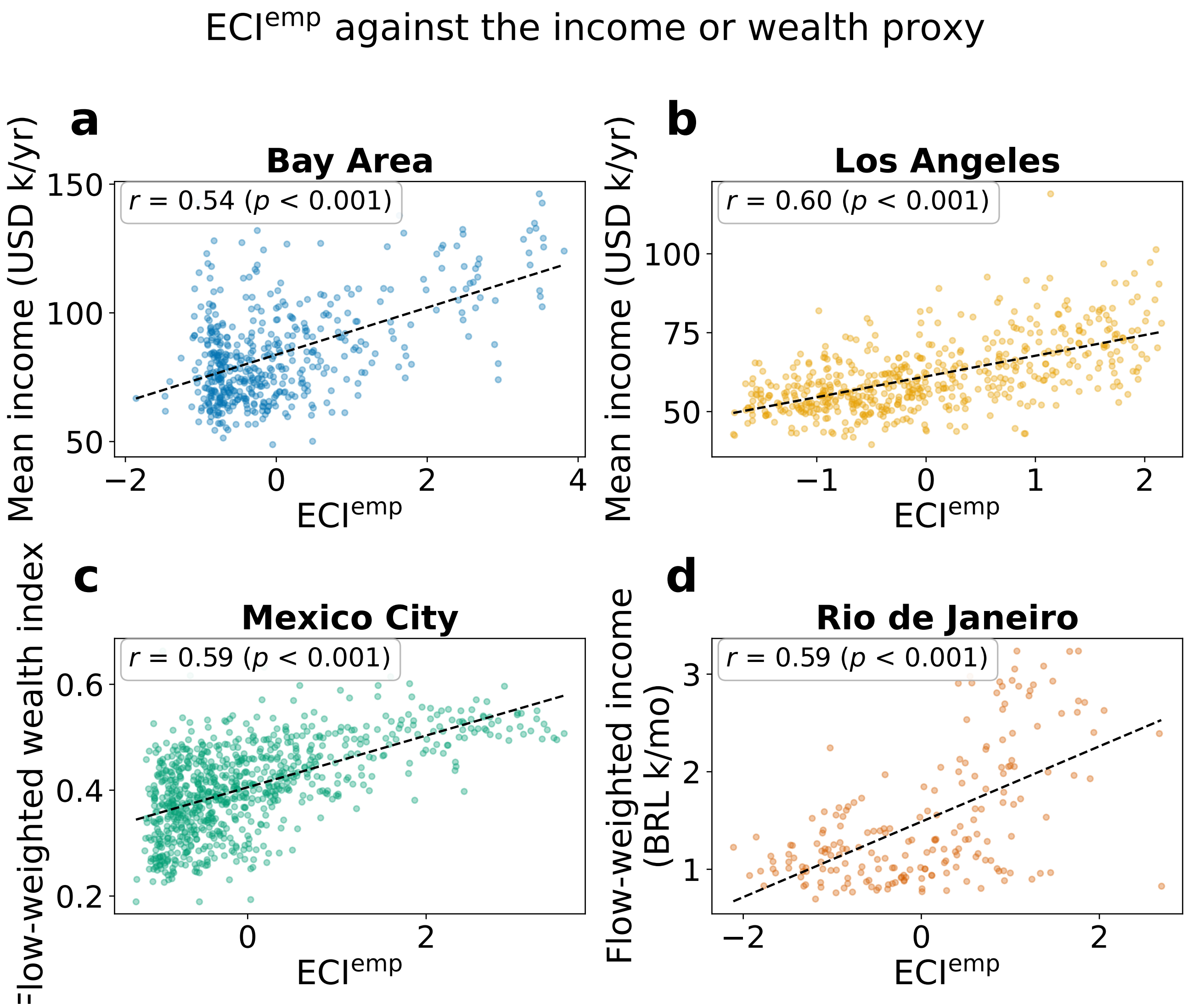

Supplementary Figure 30: Zone-level $\mathrm{ECI}^{\mathrm{emp}}$ versus employment-location income or wealth proxy across all four cities. U.S. cities use mean annual income from the Replica dataset (USD); Rio de Janeiro uses flow-weighted mean household income from the 2010 Census (BRL/month); Mexico City uses a flow-weighted mean of a PCA-based wealth percentile (0–1) derived from 16 census indicators. Dashed lines show OLS fits; correlation statistics are shown for each city. Panels **a**–**d** correspond to the Bay Area, Los Angeles, Mexico City and Rio de Janeiro, respectively. Correlations are computed over the zones with both attributes, n = 493, 546, 829 and 199 for the Bay Area, Los Angeles, Mexico City and Rio de Janeiro respectively. All correlations are Pearson coefficients from two-sided tests with no correction for multiple comparisons; degrees of freedom are $n-2$ for the sample size given above.

Supplementary Table 11: Predictive performance across all four cities for three model variants: WorkReach, Proxy-only, and combined (ECI^{emp} + Proxy). The proxy is mean annual income for U.S. cities (Replica), flow-weighted mean household income for Rio de Janeiro (2010 Census, BRL/month), and flow-weighted mean PCA wealth percentile for Mexico City (0–1).

City	Model	CPC [95% CI]	r [95% CI]
Bay Area	WorkReach	0.389 [0.381, 0.397]	0.524 [0.487, 0.560]
	Proxy-only	0.377 [0.368, 0.386]	0.484 [0.450, 0.520]
	WorkReach + Proxy	0.390 [0.382, 0.399]	0.537 [0.501, 0.573]
Los Angeles	WorkReach	0.413 [0.405, 0.420]	0.580 [0.539, 0.612]
	Proxy-only	0.412 [0.404, 0.419]	0.572 [0.544, 0.590]
	WorkReach + Proxy	0.413 [0.406, 0.421]	0.580 [0.553, 0.597]
Mexico City	WorkReach	0.486 [0.480, 0.493]	0.658 [0.648, 0.667]
	Proxy-only	0.497 [0.491, 0.503]	0.675 [0.661, 0.688]
	WorkReach + Proxy	0.499 [0.493, 0.506]	0.677 [0.663, 0.690]
Rio de Janeiro	WorkReach	0.614 [0.606, 0.621]	0.677 [0.659, 0.693]
	Proxy-only	0.636 [0.628, 0.644]	0.680 [0.660, 0.700]
	WorkReach + Proxy	0.635 [0.628, 0.643]	0.679 [0.660, 0.698]

Supplementary Table 12: Likelihood-ratio tests for the independent significance of ECI^{emp} and the income or wealth proxy across all four cities. $\Delta(2\ell)$ follows a χ^2 distribution with one degree of freedom; the test is one-sided, with no correction for multiple comparisons. All $p < 0.001$.

City	Test	$\Delta(2\ell)$	p-value
Bay Area	WorkReach → ECI^{emp} + Proxy (proxy adds to ECI^{emp})	263,136	< 0.001
	Proxy-only → ECI^{emp} + Proxy (ECI^{emp} adds to proxy)	831,813	< 0.001
Los Angeles	WorkReach → ECI^{emp} + Proxy (proxy adds to ECI^{emp})	2,596	< 0.001
	Proxy-only → ECI^{emp} + Proxy (ECI^{emp} adds to proxy)	118,590	< 0.001
Mexico City	WorkReach → ECI^{emp} + Proxy (proxy adds to ECI^{emp})	839,904	< 0.001
	Proxy-only → ECI^{emp} + Proxy (ECI^{emp} adds to proxy)	279,335	< 0.001
Rio de Janeiro	WorkReach → ECI^{emp} + Proxy (proxy adds to ECI^{emp})	604,977	< 0.001
	Proxy-only → ECI^{emp} + Proxy (ECI^{emp} adds to proxy)	194,580	< 0.001

flow-weighted income or wealth proxy outperforms ECI^{emp} alone (ΔCPC $= -0.011$ for Mexico City, -0.022 for Rio de Janeiro). An important caveat applies to these two cities: because no direct work-location wage data are available, we construct the proxy by aggregating residential income to destination zones using the observed commuting flows themselves. This flow-weighting procedure introduces a circularity, as the same flow matrix enters both the dependent variable and the construction of the covariate, which may inflate the apparent predictive advantage of the proxy in Mexico City and Rio de Janeiro. In all cases the absolute CPC differences remain small (≤ 0.022), and both covariates contribute independently to the combined model (all LR tests $p < 0.001$). Importantly, wages capture only one dimension of destination quality: the economic complexity framework additionally encodes inter-industry relatedness, productive knowledge agglomeration, and the feasibility of diversification into new activities, dimensions that we explore further in the sectoral relatedness analysis (Supplementary Note 16).

Bootstrap Significance of ECI^{emp} vs. Proxy Differences

We test whether these CPC and r differences are significant using a block bootstrap (2,000 replicates), resampling origin zones with replacement to preserve within-origin correlation. Supplementary Fig. 31 shows the resulting 95% confidence intervals. In the Bay Area, the CPC difference between ECI^{emp} and the wage proxy is not significant ($p = 0.38$), although the Pearson r difference is ($p = 0.001$). In Los Angeles, ECI^{emp} significantly outperforms the proxy on both CPC ($p < 0.001$) and r ($p < 0.001$). In Mexico City and Rio de Janeiro, the proxy outperforms ECI^{emp} on both metrics ($p \leq 0.004$), though the circularity noted above applies to this comparison. Taken together, the results confirm that ECI^{emp} and the income proxy are not interchangeable predictors across the four cities.

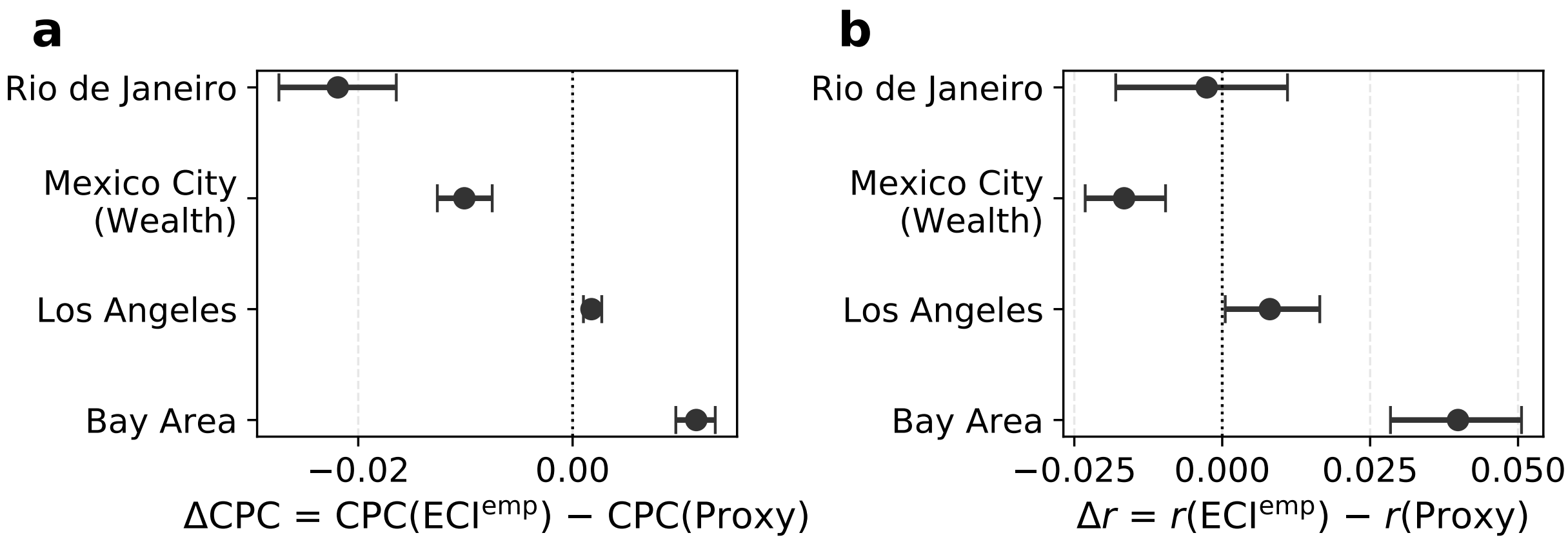

Supplementary Figure 31: Bootstrap 95% confidence intervals for the difference between the ECI^{emp}-only model and the income/wealth proxy-only model across all four cities, from n = 2,000 row-wise bootstrap replicates of the observed flow matrix. **a** Difference in CPC. **b** Difference in Pearson r. Values above zero indicate that ECI^{emp} outperforms the proxy; values below zero indicate the reverse.

Supplementary Note 16: Sectoral Relatedness Analysis

The economic complexity framework encodes not only the sophistication of individual zones (ECI^{emp}) but also the relatedness between economic sectors. Two sectors are considered related when they tend to co-locate in the same zones, which reflects shared labor requirements, technologies, or institutional conditions. We refer to this quantity as *sectoral relatedness*: it measures the similarity between two zones' sector portfolios, built from the product-space proximity between sectors [5]. This co-location structure is captured by the product-space proximity matrix $\mathbf{\Phi}$ [5], whose entries are

$$\phi_{ss'} = \frac{\sum_z M_{zs} M_{zs'}}{\max(K_s, K_{s'})} , \tag{5}$$

where M_{zs} is the Revealed Comparative Advantage indicator and $K_s = \sum_z M_{zs}$ is the ubiquity of sector s. The matrix $\mathbf{\Phi} \in \mathbb{R}^{S \times S}$ has entries $[\mathbf{\Phi}]_{ss'} = \phi_{ss'}$, so that each element measures how frequently sectors s and s' appear together across the urban economy, normalized by the more common of the two.

For each origin–destination pair (i, j), we aggregate these pairwise sector proximities into a single sectoral relatedness score

$$\text{SR}_{ij} = \mathbf{c}_i \cdot \mathbf{\Phi} \cdot \mathbf{c}_j , \tag{6}$$

where $\mathbf{c}_i = (c_{i1}, \ldots, c_{iS})$ is the employment-share vector of zone i across $S = 17$ harmonized NAICS sectors. Intuitively, SR_{ij} is high when both zones specialize in sectors that are closely related in the product space, and low when the destination offers employment that is structurally different from what the origin provides.

Following the same comparison strategy as for the income and wealth proxies (Supplementary Note 15), we estimated three model variants per city: the original WorkReach, a model replacing ECI^{emp} with SR, and a combined model including both. The SR and combined models are estimated on the subset of origin–destination pairs for which both zones have sector-level data; WorkReach is re-estimated on the same subset for a valid likelihood-ratio comparison.

Supplementary Table 14 reports parameter estimates and performance metrics for all variants across the four cities. The SR coefficient is negative in all four cities, indicating that workers systematically commute to destinations whose sector composition is less related to their home zone, consistent with a necessity or sectoral-mismatch hypothesis rather than a sector-matching one.

Supplementary Table 13: Predictive performance for WorkReach, SR-only, and combined (ECI^{emp} + SR) models, with block-bootstrap 95% confidence intervals (2,000 replicates).

City	Model	CPC [95% CI]	r [95% CI]
Bay Area	WorkReach	0.389 [0.381, 0.395]	0.524 [0.485, 0.560]
	SR-only	0.391 [0.382, 0.398]	0.522 [0.499, 0.543]
	WorkReach+SR	0.399 [0.390, 0.405]	0.553 [0.530, 0.575]
Los Angeles	WorkReach	0.413 [0.405, 0.421]	0.580 [0.542, 0.606]
	SR-only	0.425 [0.415, 0.434]	0.583 [0.567, 0.602]
	WorkReach+SR	0.428 [0.419, 0.436]	0.597 [0.570, 0.613]
Mexico City	WorkReach	0.486 [0.480, 0.492]	0.658 [0.648, 0.666]
	SR-only	0.495 [0.488, 0.501]	0.684 [0.671, 0.694]
	WorkReach+SR	0.497 [0.491, 0.503]	0.681 [0.668, 0.691]
Rio de Janeiro	WorkReach	0.614 [0.607, 0.621]	0.677 [0.661, 0.694]
	SR-only	0.652 [0.646, 0.659]	0.734 [0.719, 0.747]
	WorkReach+SR	0.655 [0.649, 0.662]	0.735 [0.720, 0.748]

Likelihood-ratio tests confirm that SR significantly improves model fit beyond ECI^{emp} alone in all four cities, and that ECI^{emp} likewise contributes independently beyond SR (Supplementary Table 15).

ECI^{emp} remains independently significant after controlling for SR in all four cities, as seen by the combined model retaining a positive β_{ECI} and a significant LR test statistic in every case. The marginal contribution of ECI^{emp} beyond SR is largest in the Bay Area ($\Delta(2\ell) = 517{,}799$) and smallest in Rio de Janeiro ($\Delta(2\ell) = 140{,}059$). Pairwise Pearson correlations between destination ECI^{emp} and SR across all off-diagonal origin–destination pairs are moderate in Mexico City ($r = -0.51$) and the Bay Area ($r = -0.48$), weak in Rio de Janeiro ($r = -0.24$), and negligible in Los Angeles ($r = -0.02$). The negative sign of β_{SR} across all cities supports the interpretation that long-distance commutes are associated with sector dissimilarity, meaning that workers travel to zones offering employment in sectors different from those available near home, consistent with an occupational-mismatch mechanism rather than sector matching.

Supplementary Table 14: Parameter estimates with multinomial bootstrap 95% confidence intervals for WorkReach, SR-only, and combined (ECI^{emp} + SR) models. Threshold τ is reported in km.

City	Model	β_{dist}	β_{ECI}	β_{SR}	β_{inf}	τ [km]	k
Bay Area	WorkReach	−21.292 [−21.305, −21.278]	2.462 [2.459, 2.465]	–	−14.735 [−14.762, −14.707]	0.006 [0.006, 0.006]	128.764 [128.760, 128.766]
	SR-only	−13.140 [−13.152, −13.127]	–	−8.542 [−8.550, −8.533]	−2.427 [−2.457, −2.408]	0.006 [0.006, 0.006]	28.165 [28.159, 28.173]
	ECI^{emp}+SR	−17.683 [−17.701, −17.667]	1.562 [1.557, 1.566]	−8.542 [−8.552, −8.532]	−4.362 [−4.389, −4.340]	0.006 [0.006, 0.006]	37.972 [37.964, 37.979]
Los Angeles	WorkReach	−9.601 [−9.609, −9.593]	0.482 [0.479, 0.484]	–	−8.702 [−8.716, −8.687]	0.006 [0.006, 0.006]	32.413 [32.398, 32.439]
	SR-only	−5.778 [−5.791, −5.766]	–	−6.756 [−6.773, −6.742]	−1.794 [−1.808, −1.775]	2.189 [2.158, 2.213]	19.589 [19.541, 19.646]
	ECI^{emp}+SR	−5.858 [−5.872, −5.847]	0.698 [0.695, 0.701]	−6.714 [−6.729, −6.701]	−1.836 [−1.850, −1.818]	2.599 [2.571, 2.619]	20.438 [20.391, 20.489]
Mexico City	WorkReach	−21.881 [−21.898, −21.861]	4.220 [4.208, 4.230]	–	10.250 [10.207, 10.280]	16.201 [16.169, 16.222]	10.824 [10.782, 10.880]
	SR-only	−7.390 [−7.402, −7.381]	–	−4.595 [−4.604, −4.587]	−0.665 [−0.682, −0.650]	2.252 [2.246, 2.258]	46.397 [46.274, 46.512]
	ECI^{emp}+SR	−7.384 [−7.396, −7.375]	1.050 [1.047, 1.056]	−3.554 [−3.563, −3.543]	−4.620 [−4.644, −4.602]	2.254 [2.249, 2.260]	47.955 [47.951, 47.960]
Rio	WorkReach	−16.447 [−16.459, −16.424]	6.603 [6.593, 6.615]	–	4.267 [4.244, 4.298]	24.955 [24.904, 25.026]	9.654 [9.580, 9.720]
	SR-only	−3.692 [−3.701, −3.681]	–	−6.756 [−6.768, −6.743]	−0.792 [−0.814, −0.768]	2.126 [2.113, 2.139]	27.612 [27.524, 27.688]
	ECI^{emp}+SR	−4.027 [−4.036, −4.014]	0.935 [0.931, 0.939]	−6.756 [−6.767, −6.745]	−1.133 [−1.156, −1.112]	2.128 [2.114, 2.139]	27.617 [27.530, 27.684]

Supplementary Table 15: Likelihood-ratio tests for the independent significance of $\mathrm{ECI^{emp}}$ and sectoral relatedness across all four cities. $\Delta(2\ell)$ follows a χ^2 distribution with one degree of freedom; the test is one-sided, with no correction for multiple comparisons. All $p < 0.001$.

City	Test	$\Delta(2\ell)$	p-value
Bay Area	WorkReach → $\mathrm{ECI^{emp}}$ + SR (SR adds to $\mathrm{ECI^{emp}}$)	552,839	< 0.001
	SR → $\mathrm{ECI^{emp}}$ + SR ($\mathrm{ECI^{emp}}$ adds to SR)	517,799	< 0.001
Los Angeles	WorkReach → $\mathrm{ECI^{emp}}$ + SR (SR adds to $\mathrm{ECI^{emp}}$)	1,109,813	< 0.001
	SR → $\mathrm{ECI^{emp}}$ + SR ($\mathrm{ECI^{emp}}$ adds to SR)	231,432	< 0.001
Mexico City	WorkReach → $\mathrm{ECI^{emp}}$ + SR (SR adds to $\mathrm{ECI^{emp}}$)	723,691	< 0.001
	SR → $\mathrm{ECI^{emp}}$ + SR ($\mathrm{ECI^{emp}}$ adds to SR)	220,929	< 0.001
Rio de Janeiro	WorkReach → $\mathrm{ECI^{emp}}$ + SR (SR adds to $\mathrm{ECI^{emp}}$)	1,445,363	< 0.001
	SR → $\mathrm{ECI^{emp}}$ + SR ($\mathrm{ECI^{emp}}$ adds to SR)	140,059	< 0.001

Marginal Substitution Rates

The marginal substitution rate (MSR) framework carries over directly to the specifications that include sectoral relatedness. Because SR_{ij} enters the utility through the same transition weight $w_{ij}(\tau, k)$ as $\mathrm{ECI^{emp}}$ and informality, its marginal utility, and hence its substitution rates, follow the expressions derived for $\mathrm{ECI^{emp}}$. In the sectoral-relatedness-only specification, where SR_{ij} replaces $\mathrm{ECI}_j^{\mathrm{emp}}$, the substitution rate of relatedness for distance is $\mathrm{MSR}_{\mathrm{SR/dist}} = \beta_{\mathrm{SR}}/\beta_{\mathrm{dist}}$, in direct analogy with $\mathrm{MSR}_{\mathrm{ECI^{emp}/dist}} = \beta_{\mathrm{ECI^{emp}}}/\beta_{\mathrm{dist}}$ in the WorkReach model. Its estimated value is 0.650 in the Bay Area, 1.169 in Los Angeles, 0.622 in Mexico City, and 1.830 in Rio de Janeiro. Supplementary Table 16 reports the complete set of pairwise substitution rates for this specification.

For the combined $\mathrm{ECI^{emp}}$+SR model, the systematic utility is

$$V_{ij} = \beta_{\mathrm{dist}} d_{ij} + w_{ij}(\tau, k) \left(\beta_{\mathrm{ECI^{emp}}}\, \mathrm{ECI}_j^{\mathrm{emp}} + \beta_{\mathrm{SR}}\, \mathrm{SR}_{ij} + \beta_{\mathrm{inf}}\, \mathrm{Inf}_i\right) , \tag{7}$$

Here distance plays two roles by entering directly through $\beta_{\mathrm{dist}} d_{ij}$ and indirectly through the transition weight $w_{ij}(\tau, k)$, which scales the three opportunity- and origin-side terms. Differentiating with respect to d_{ij} therefore produces a direct term together with one from the distance dependence of w_{ij}; using $\partial w_{ij}/\partial d_{ij} = k\, w_{ij}(1 - w_{ij})$,

$$\frac{\partial V_{ij}}{\partial d_{ij}} = \beta_{\mathrm{dist}} + k\, w_{ij}(1 - w_{ij}) \left(\beta_{\mathrm{ECI^{emp}}}\, \mathrm{ECI}_j^{\mathrm{emp}} + \beta_{\mathrm{SR}}\, \mathrm{SR}_{ij} + \beta_{\mathrm{inf}}\, \mathrm{Inf}_i\right) , \tag{8}$$

whereas each modulated attribute enters only through w_{ij}, giving $\partial V_{ij}/\partial \mathrm{ECI}_j^{\mathrm{emp}} = w_{ij}\beta_{\mathrm{ECI^{emp}}}$, $\partial V_{ij}/\partial \mathrm{SR}_{ij} = w_{ij}\beta_{\mathrm{SR}}$, and $\partial V_{ij}/\partial \mathrm{Inf}_i = w_{ij}\beta_{\mathrm{inf}}$. The marginal substitution rate between distance and any modulated attribute $X \in \{\mathrm{ECI^{emp}}, \mathrm{SR}, Inf\}$ follows from the ratio of the corresponding marginal utilities and holds at any value of the transition weight,

$$\begin{aligned} \mathrm{MSR}_{X/\mathrm{dist}} &= \frac{\partial V_{ij}/\partial X}{\partial V_{ij}/\partial d_{ij}} \\ &= \frac{w_{ij}\beta_X}{\beta_{\mathrm{dist}} + k\, w_{ij}(1 - w_{ij}) \left(\beta_{\mathrm{ECI^{emp}}}\, \mathrm{ECI}_j^{\mathrm{emp}} + \beta_{\mathrm{SR}}\, \mathrm{SR}_{ij} + \beta_{\mathrm{inf}}\, \mathrm{Inf}_i\right)} \\ &\xrightarrow[w_{ij} \to 1]{} \frac{\beta_X}{\beta_{\mathrm{dist}}} . \end{aligned} \tag{9}$$

This general form varies with location through w_{ij} and the attribute values. The coefficient ratio $\beta_X/\beta_{\mathrm{dist}}$ is the special case obtained in the opportunity-driven regime ($w_{ij} \to 1$, the limit of long trips for which the transition weight saturates), where the modulating term $k\, w_{ij}(1 - w_{ij})$ vanishes; this limiting value is the one reported in Supplementary Table 17, following the convention of Table 2. The substitution rate between two modulated attributes, by contrast, holds in every regime: the shared weight w_{ij} cancels exactly, so $\mathrm{MSR}_{X/Y} = \beta_X/\beta_Y$ at any distance.

This structure generalizes to any number of modulated attributes. For a utility of the form $V_{ij} = \beta_{\mathrm{dist}}\, d_{ij} + w_{ij}(\tau, k) \sum_{m=1}^{K} \beta_m X_{ij}^{(m)}$, in which K attributes share the transition weight, each modulated attribute has marginal utility $w_{ij}\beta_m$. The substitution rate between any two modulated attributes is therefore the ratio of their coefficients, $\mathrm{MSR}_{X^{(a)}/X^{(b)}} = \beta_a/\beta_b$, independent of w_{ij} and hence of location, while the

rate against distance reduces to $\beta_m/\beta_{\text{dist}}$ in the opportunity-driven regime. The functional form of each pairwise rate is thus invariant to how many attributes the model contains; the fitted coefficients are not, so the numerical rates shift as covariates are added, as reflected in the attenuation of $\beta_{\text{ECI}^{\text{emp}}}$ between the WorkReach model and the combined specification.

Supplementary Table 17 reports the full set of pairwise substitution rates for the combined model. A negative $\text{MSR}_{\text{ECI}^{\text{emp}}/\text{dist}}$ reflects a positive trade-off (workers tolerate longer commutes for higher ECI^{emp}), and its magnitude is smaller than in the WorkReach model, consistent with the attenuation of $\beta_{\text{ECI}^{\text{emp}}}$ once sectoral relatedness is included. The positive $\text{MSR}_{\text{SR}/\text{dist}}$ in every city indicates that higher sectoral relatedness amplifies the distance penalty, the behavioral counterpart of the negative β_{SR}: workers tolerate longer commutes toward destinations whose sector composition is less related to their own. Once sectoral relatedness is controlled for, $\text{MSR}_{Inf/\text{dist}}$ is positive in all four cities, including Mexico City and Rio de Janeiro, where β_{inf} changes sign relative to the WorkReach model. The relatedness-to-complexity rate $\text{MSR}_{\text{SR}/\text{ECI}^{\text{emp}}} = \beta_{\text{SR}}/\beta_{\text{ECI}^{\text{emp}}}$ exceeds one in magnitude in every city, so holding utility fixed, an added unit of sectoral relatedness must be offset by more than a unit of economic complexity: relatedness weighs more heavily on the choice than complexity.

Supplementary Table 16: Pairwise marginal substitution rates (MSR) for the sectoral-relatedness-only model, computed as ratios of coefficients β_X/β_Y in the opportunity-driven regime ($w_{ij} \to 1$).

	**Bay Area**	**Los Angeles**	**Mexico City**	**Rio de Janeiro**
$\text{MSR}_{\text{SR}/\text{dist}}$	0.650	1.169	0.622	1.830
95% C.I.	[0.649, 0.651]	[1.166, 1.173]	[0.620, 0.623]	[1.824, 1.836]
$\text{MSR}_{Inf/\text{dist}}$	0.185	0.310	0.090	0.215
95% C.I.	[0.183, 0.187]	[0.308, 0.313]	[0.088, 0.092]	[0.208, 0.221]
$\text{MSR}_{Inf/\text{SR}}$	0.284	0.266	0.145	0.117
95% C.I.	[0.281, 0.287]	[0.263, 0.268]	[0.141, 0.148]	[0.114, 0.121]

Supplementary Table 17: Pairwise marginal substitution rates (MSR) for the combined ECI^{emp}+SR model, computed as ratios of coefficients β_X/β_Y in the opportunity-driven regime ($w_{ij} \to 1$).

	**Bay Area**	**Los Angeles**	**Mexico City**	**Rio de Janeiro**
$\text{MSR}_{\text{ECI}^{\text{emp}}/\text{dist}}$	−0.088	−0.119	−0.142	−0.232
95% C.I.	[−0.089, −0.088]	[−0.120, −0.119]	[−0.143, −0.142]	[−0.233, −0.231]
$\text{MSR}_{\text{SR}/\text{dist}}$	0.483	1.146	0.481	1.678
95% C.I.	[0.482, 0.484]	[1.143, 1.150]	[0.480, 0.483]	[1.672, 1.683]
$\text{MSR}_{Inf/\text{dist}}$	0.247	0.313	0.626	0.281
95% C.I.	[0.245, 0.248]	[0.311, 0.316]	[0.623, 0.629]	[0.276, 0.287]
$\text{MSR}_{\text{SR}/\text{ECI}^{\text{emp}}}$	−5.469	−9.619	−3.385	−7.226
95% C.I.	[−5.486, −5.452]	[−9.665, −9.573]	[−3.402, −3.368]	[−7.259, −7.193]
$\text{MSR}_{Inf/\text{ECI}^{\text{emp}}}$	−2.793	−2.630	−4.400	−1.212
95% C.I.	[−2.810, −2.775]	[−2.656, −2.605]	[−4.428, −4.373]	[−1.236, −1.188]
$\text{MSR}_{Inf/\text{SR}}$	0.511	0.273	1.300	0.168
95% C.I.	[0.508, 0.514]	[0.271, 0.276]	[1.293, 1.307]	[0.164, 0.171]

ECI^{emp} versus Sectoral Relatedness: Predictive and Policy Trade-offs

The SR-only and ECI^{emp}+SR models outperform WorkReach in CPC and r across all four cities, raising a natural question about why we center the WorkReach specification on ECI^{emp} rather than SR. The answer lies in the distinct analytical roles of the two quantities and their suitability for policy applications.

ECI^{emp} is an intrinsic property of a destination zone: it summarizes the diversity and sophistication of the zone's productive structure without reference to any particular origin. Sectoral relatedness, by contrast, is a pairwise quantity that measures the overlap in sector composition between a specific origin and a specific destination. It cannot be attributed to either zone in isolation; the same destination may have high SR with respect to one origin and low SR with respect to another. This asymmetry has three consequences for policy translation.

First, because ECI^{emp} is a zone-level attribute, it can be paired with the opportunity score [5, 7] to design targeted economic development strategies. The opportunity score identifies which new sectors a zone could realistically develop given the relatedness of those sectors to the zone's existing economic activities. An increase in ECI^{emp} resulting from the adoption of new sectors can be fed directly into WorkReach to simulate the change in commuting accessibility, as we demonstrate in Supplementary Note 19. No comparable intervention exists for sectoral relatedness: because the metric depends jointly on the compositions of both origin and destination, there is no well-defined policy lever at a single location that would systematically shift SR across all origin–destination pairs.

Second, ECI^{emp} captures information that neither SR nor wages provide. SR measures sector overlap between two zones but says nothing about the sophistication of those sectors; wages summarize current compensation but not the productive structure that sustains it. ECI^{emp}, by contrast, reflects which sectors co-locate and how that pattern compares across zones, encoding the agglomeration externalities and tacit knowledge that underpin long-run economic development [8, 9]. Wages, tested as a covariate in Supplementary Note 15, summarize current compensation levels but provide no information about the structural conditions that sustain or could improve those levels. A policy objective framed as "increase wages at location j" lacks a mechanism; the complexity framework supplies that mechanism by identifying which productive activities are feasible and which would raise ECI^{emp}.

Third, adding SR as a sixth covariate to the existing WorkReach specification improves fit but complicates interpretation. The five-parameter model has a clear structure: each coefficient maps to a single behavioral quantity (distance penalty, attraction to complexity, effect of residential informality, regime threshold, transition steepness). An SR coefficient, by contrast, mixes labor-market mismatch with spatial sorting, making it harder to derive policy-relevant elasticities from the model. Sectoral relatedness is moreover a property of the home–work pair rather than of the home zone, and so does not align with residential informality. Two origins with the same informality can have very different relatedness to a given destination, and two with very different informality nearly the same, which blurs the link between worker demographics and opportunity that the parsimonious model keeps clear.

In summary, SR and ECI^{emp} are complementary quantities derived from the same product-space structure. We report the SR analysis in full to demonstrate that the economic complexity framework captures multiple drivers of commuting behavior, while retaining ECI^{emp} in the main specification because it is the dimension most amenable to policy intervention.

To characterize how sector composition relates to commuting patterns, Supplementary Fig. 32 computes the within-sector commuting fraction: the share of workers in a given sector whose origin zone's dominant sector matches the destination sector. Because these are absolute shares across the full population, they reflect both how frequently a sector appears as the dominant activity across zones and how spatially concentrated its employment is. The fractions vary substantially across sectors and reveal clear distinctions between regions. Retail stands out in Latin American cities ($\approx$63% in Mexico City, $\approx$38% in Rio de Janeiro), far above the U.S. cities where it remains below 10%, consistent with retail being the dominant sector in a large number of zones in those metropolitan areas. The pattern reverses for health care, professional services, and manufacturing, all of which show markedly higher fractions in the Bay Area and Los Angeles than in Mexico City or Rio de Janeiro. These contrasts reflect differences in which sectors dominate the spatial landscape of employment in each region.

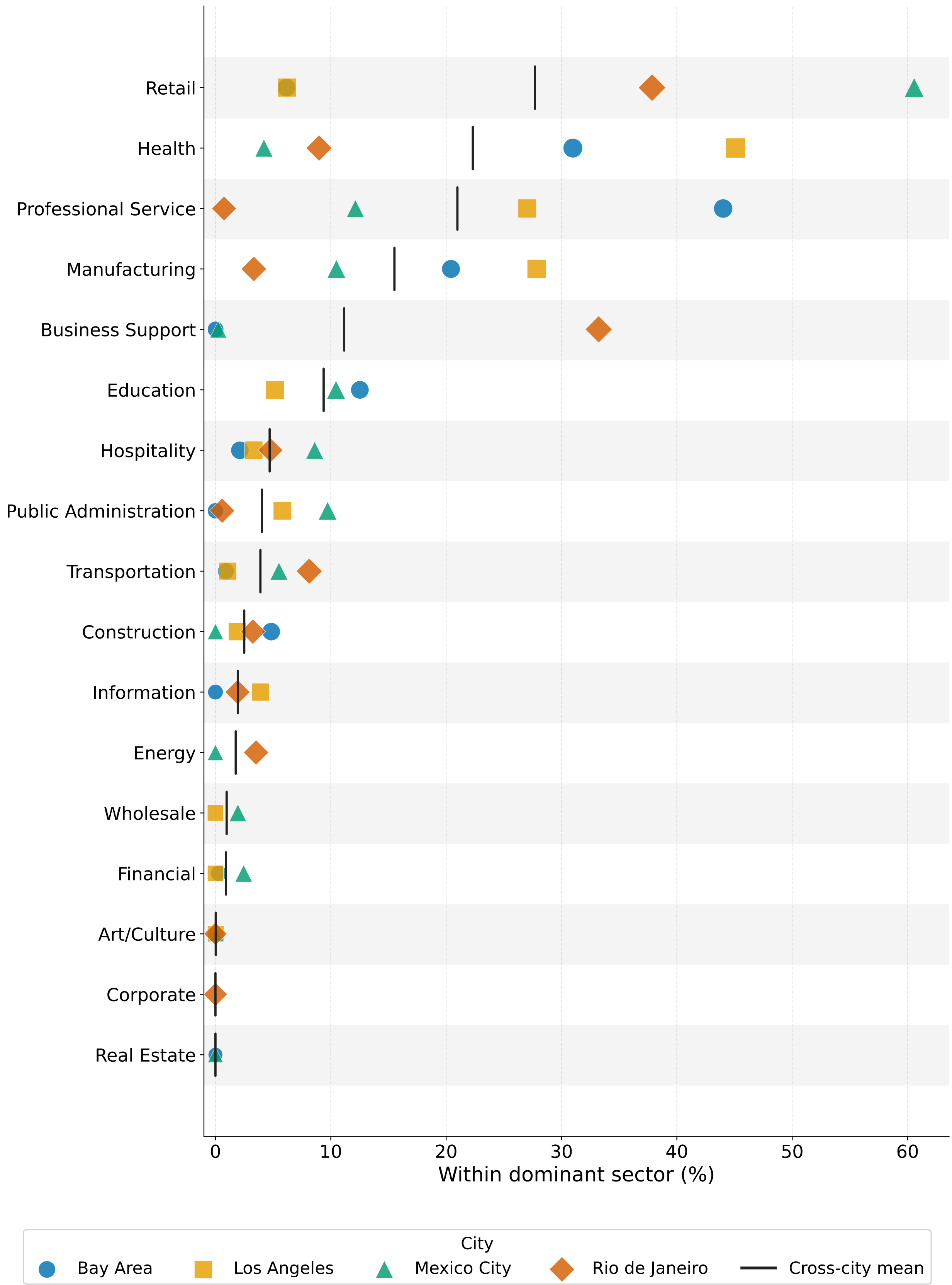

Supplementary Figure 32: Within-sector commuting fraction by sector and city. Each dot represents the fraction of commuters in a given sector whose origin zone's dominant sector matches the destination sector. Each point is one dominant sector in one city, weighted by the total outflow from the zones it dominates. Sectors shown are those dominant in at least one zone, n = 14, 13, 15 and 13 for the Bay Area, Los Angeles, Mexico City and Rio de Janeiro respectively.

Supplementary Fig. 33 stratifies the SR–distance relationship by origin informality level using two measures: OD-pair SR (computed for each origin–destination pair) and per-origin mean SR (averaged across all destinations for a given origin). In Mexico City and Rio de Janeiro, the high-informality group exhibits a negative correlation between SR and commuting distance under both measures, while the low-informality group shows no significant relationship. In Bay Area and Los Angeles, the correlation is negative for all informality groups under both measures, with one exception: OD-pair SR is not significant for the low-informality group in the Bay Area. These patterns indicate that workers from high-informality zones are more likely to cross sector boundaries to reach distant employment, a relationship that is consistent across cities and robust to the choice of SR aggregation.

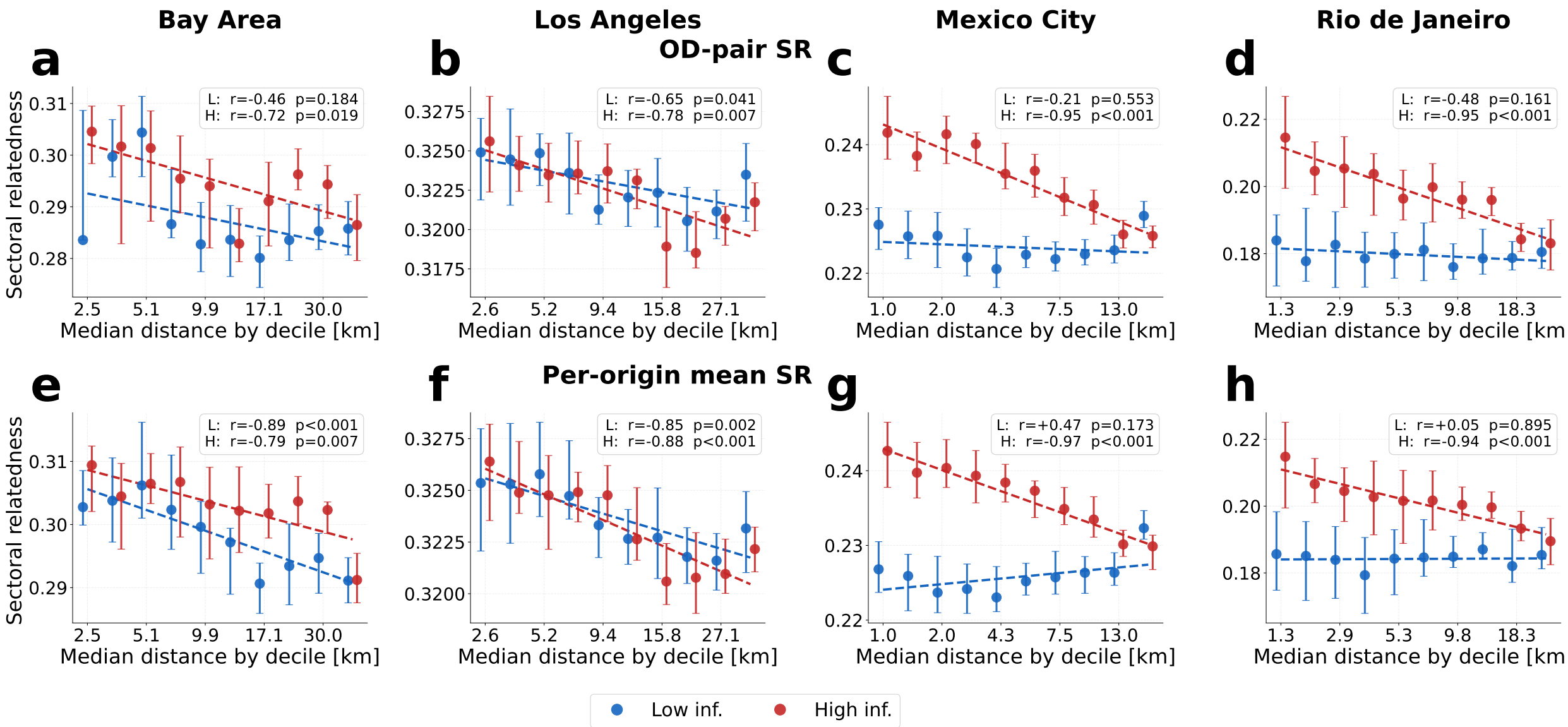

Supplementary Figure 33: Binned scatter plot of sectoral relatedness versus commuting distance decile, stratified by origin informality group. Points are flow-weighted medians across the origin zones in each cell and error bars are 95% confidence intervals from n = 500 bootstrap resamples; the correlation and its p-value are given in each panel. Panels **a**–**d** show OD-pair SR and panels **e**–**h** per-origin mean SR, for the Bay Area, Los Angeles, Mexico City and Rio de Janeiro in that order. In Latin American cities, only the high-informality group exhibits a significant negative relationship; in U.S. cities, the relationship is negative for all groups except the low-informality group in the Bay Area (OD-pair SR). Reported correlations are two-sided and weighted by the flow in each decile, with 8 degrees of freedom for the ten deciles and no correction for multiple comparisons.

Supplementary Note 17: Relationship between Accessibility and Informality

The WorkReach model allows to compute accessibility metrics based on utility (consumer surplus). Figure 34 shows the relationship between the informality rate of the home location and three different measures of accessibility (distance weighted, consumer surplus, and the mean of the z-scores of the previous two). All of the cities except for Mexico City exhibit medium to strong correlations for the three accessibility metrics.

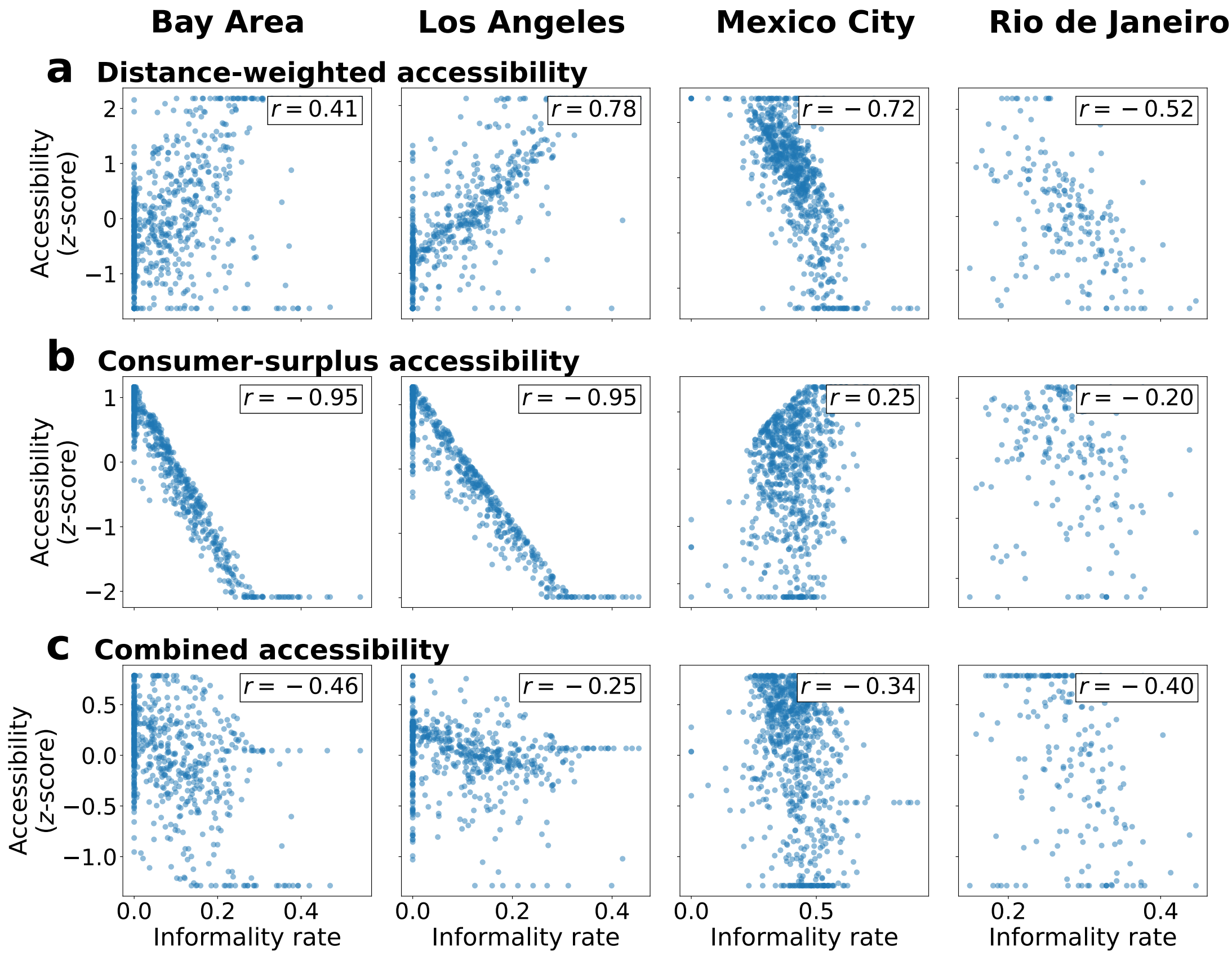

Supplementary Figure 34: Scatter plots of accessibility versus informality rate for each city. **a** Distance-weighted accessibility. **b** Consumer Surplus accessibility. **c** Combined accessibility (mean of z-scored DW and CS measures). The plots show the Pearson correlation (r) for each relationship, highlighting how the relationship between accessibility and informality differs depending on the metric used. Correlations are computed over n = 677, 605, 793 and 199 zones for the Bay Area, Los Angeles, Mexico City and Rio de Janeiro respectively. All correlations are Pearson coefficients from two-sided tests with no correction for multiple comparisons; degrees of freedom are $n - 2$ for the sample size given above.

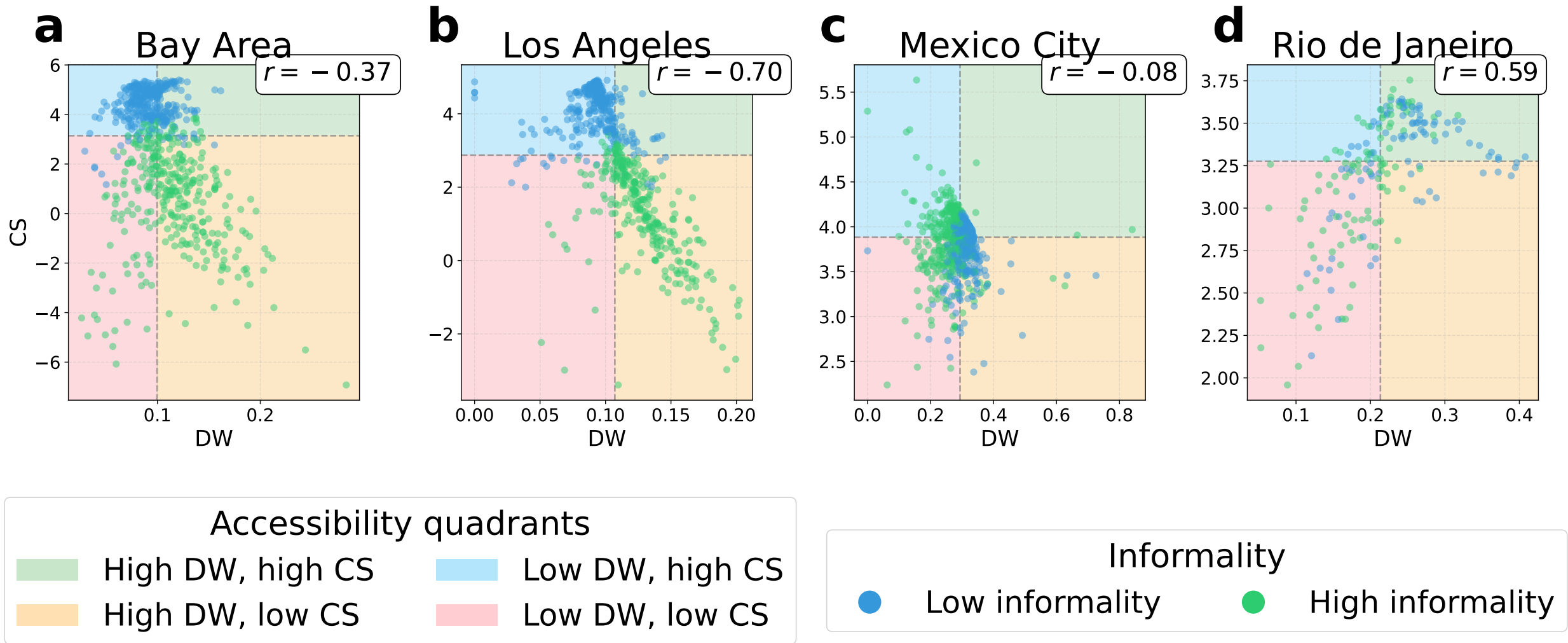

Supplementary Figure 35: Relationship between distance-weighted (DW) and consumer-surplus (CS) accessibility measures across cities. Scatter plots show the correlation between distance-weighted accessibility (x-axis) and consumer-surplus accessibility (y-axis) for each origin location, with points colored by informality level (blue = low informality, green = high informality). Background quadrants indicate accessibility combinations: high DW/high CS (green), high DW/low CS (orange), low DW/high CS (light blue), and low DW/low CS (pink). Pearson correlation coefficients (r) quantify the linear relationship between the two accessibility measures. Dashed lines indicate median values for each measure. Panels **a**–**d** correspond to the Bay Area, Los Angeles, Mexico City and Rio de Janeiro, respectively. Correlations are computed over n = 677, 605, 793 and 199 zones for the Bay Area, Los Angeles, Mexico City and Rio de Janeiro respectively. All correlations are Pearson coefficients from two-sided tests with no correction for multiple comparisons; degrees of freedom are $n - 2$ for the sample size given above.

Supplementary Note 18: Correlation to Census Variables

To contextualize the informality rate used in the WorkReach model, Supplementary Table 18 reports the census variables most strongly correlated with the informality rate in each city.

Supplementary Table 18: Top 5 Census Variables Correlated with informality rate

City	Variable Description	Correlation
**Bay Area**	Non-family households at/above poverty level (%)	0.479
	Family households at/above poverty level (%)	-0.427
	Female population 16+ didn't work. Household at/above poverty level (%)	-0.415
	Male population 16+ didn't work. Household at/above poverty level (%)	-0.413
	Female population 16+ not in labor force. Household at/above poverty level (%)	-0.410
**Los Angeles**	Foreign-born non-citizens at/above poverty line (%)	0.443
	Households in owner-occupied houses at/above poverty level (%)	-0.399
	Households with retirement income (%)	-0.395
	Foreign-born non-citizens below poverty line (%)	0.390
	Female population 16+ not in labor force. Household at/above poverty level (%)	-0.380
**Mexico City**	Occupants in overcrowded housing (%)	0.743
	Population 15+ without basic education (%)	0.742
	Dwellings without internet (%)	0.740
	Dwellings without refrigerator (%)	0.692
	Occupants in household without mobile phone (%)	0.558
**Rio de Janeiro**	Dwellings without computer at home (%)	0.783
	Housing inadequacy level	0.737
	Dwellings without washing machine (%)	0.728
	Household density (persons per room) (%)	0.699
	Household income (in minimum wages)	-0.681

Supplementary Note 19: Counterfactual $\mathrm{ECI}^{\mathrm{emp}}$ Uplift and Worker Inflow

The WorkReach model can be used to evaluate how targeted changes in a zone's economic structure would reshape commuting flows. We combine the product-space framework with the fitted model to identify zones where gaining a single feasible sector would attract the most additional workers, and we decompose the predicted new inflow by the informality level of the sending origins.

Opportunity Scoring

For each zone z that does not yet hold a comparative advantage in sector s, we compute an opportunity score based on its relatedness density and the sector's product complexity:

$$O_{zs} = \omega_{zs} \cdot \mathrm{PCI}_s^{\mathrm{emp}} \cdot (1 - M_{zs}) \,, \tag{10}$$

where ω_{zs} is the relatedness density of zone z to sector s, defined as

$$\omega_{zs} = \frac{\sum_{s'} \phi_{ss'} \, M_{zs'}}{\sum_{s'} \phi_{ss'}} \,, \tag{11}$$

and $\phi_{ss'}$ is the product-space proximity between sectors s and s' (defined in Supplementary Note 16). The opportunity score is high when a zone already hosts many sectors that are related to s (high ω_{zs}) and s itself has high product complexity (high $\mathrm{PCI}_s^{\mathrm{emp}}$). For each zone we select the sector $s_z^* = \arg\max_s O_{zs}$ as the recommended diversification target.

Counterfactual $\mathrm{ECI}^{\mathrm{emp}}$ and Inflow Decomposition

We simulate the effect of each zone z gaining its recommended sector s_z^* by setting $M_{z,s_z^*} = 1$, recomputing $\mathrm{ECI}^{\mathrm{emp}}$ from the modified RCA matrix, and obtaining a counterfactual $\mathrm{ECI}^{\mathrm{emp}}$ gain $\Delta\mathrm{ECI}_z^{\mathrm{emp}}$. Zones with $\Delta\mathrm{ECI}_z^{\mathrm{emp}} \leq 0$ are discarded. We further restrict candidates to zones with above-median baseline inflow, so that the simulated changes affect a meaningful number of workers.

For each surviving candidate z, we replace its $\mathrm{ECI}^{\mathrm{emp}}$ value, recompute the full WorkReach probability matrix, and measure the change in predicted inflow. We further decompose the inflow change by whether the sending origin belongs to the high- or low-informality group, with percentage changes computed relative to each group's baseline inflow. The top ten zones per city are selected by total percentage inflow increase.

To illustrate where these opportunities concentrate, Supplementary Fig. 36 maps the selected zones for each city. Panels **a**–**d** show the ten highest-impact zones colored by $\Delta\mathrm{ECI}^{\mathrm{emp}}$, overlaid on the baseline $\mathrm{ECI}^{\mathrm{emp}}$ distribution, and panels **e**–**h** color all candidate zones by their recommended sector. In all four cities the selected zones are spatially dispersed and span a range of baseline complexity levels, indicating that the gains are not confined to already-complex cores.

Supplementary Fig. 37 summarizes the inflow response across cities: panel **a** reports the median percentage change in worker inflow for all origins, high-informality origins, and low-informality origins, with interquartile ranges; panel **b** shows the share of new workers arriving from high-informality zones, where values above 50% indicate that the uplift disproportionately benefits informal-sector workers; and panel **c** reports the mean shift in each zone's high-informality workforce share (in percentage points), capturing whether the uplift changes the composition of the zone's labor force.

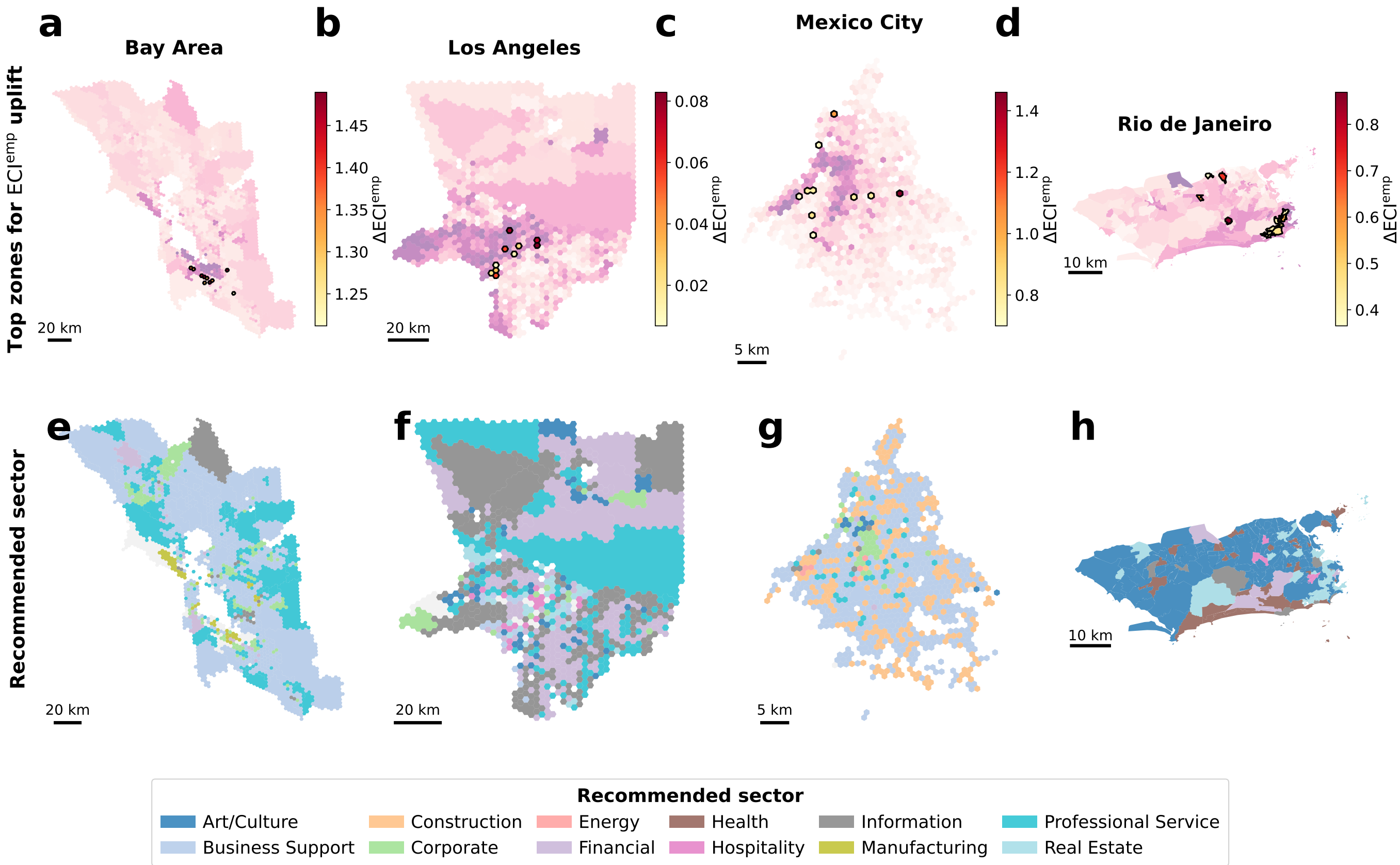

Supplementary Figure 36: Counterfactual $\mathrm{ECI}^{\mathrm{emp}}$ uplift analysis. **a**–**d** The ten zones per city with the largest predicted percentage increase in worker inflow after gaining their highest-opportunity sector, colored by $\Delta\mathrm{ECI}^{\mathrm{emp}}$ (background shading reflects baseline $\mathrm{ECI}^{\mathrm{emp}}$). **e**–**h** All candidate zones colored by their recommended sector. Cities appear in the order Bay Area, Los Angeles, Mexico City and Rio de Janeiro in both cases. The sector legend is shared across cities. Panels **a**–**d** mark the n = 10 highest-impact zones per city; panels **e**–**h** show every feasible zone.

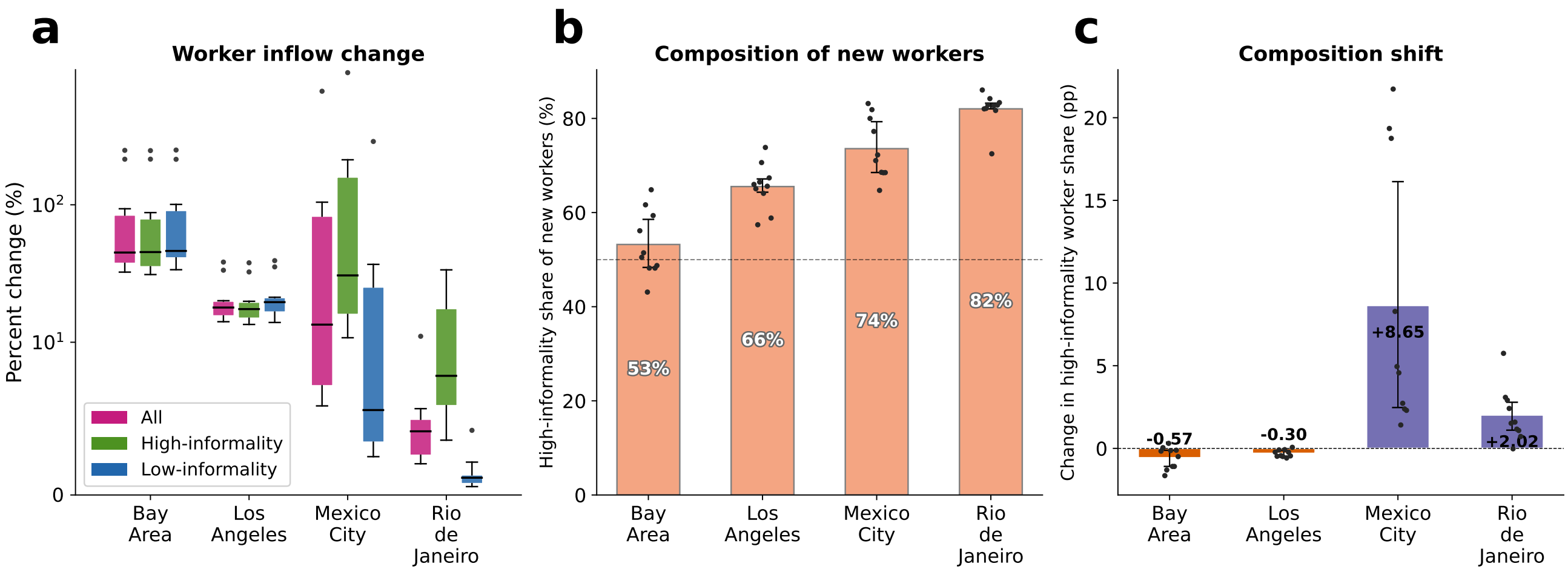

Supplementary Figure 37: Cross-city summary of counterfactual inflow changes for the n = 10 highest-impact zones per city. **a** Percentage change in worker inflow, decomposed by origin informality group, shown as boxes spanning the 25th to 75th percentiles with the median as the center line, whiskers extending to the most extreme value within 1.5 times the interquartile range and zones beyond that drawn individually, on a symmetric logarithmic vertical axis. **b** Share of new workers originating from high-informality zones, where bars are the mean across the ten zones, error bars span the 25th to 75th percentile and each of the n = 10 zones is overlaid as a point. **c** Mean change in the high-informality share of each zone's workforce (percentage points), with error bars spanning the 25th to 75th percentile and each of the n = 10 zones overlaid as a point; positive values indicate a compositional shift toward workers from high-informality origins.

Supplementary References

1. INEGI. *Encuesta Origen-Destino en Hogares de la Zona Metropolitana del Valle de México (EOD) 2017* tech. rep. (Instituto Nacional de Estadística y Geografía, Aguascalientes, Mexico, 2017).
2. Cabanas-Tirapu, O., Danús, L., Moro, E., Sales-Pardo, M. & Guimerà, R. Human mobility is well described by closed-form gravity-like models learned automatically from data. *Nature Communications* **16.** Publisher: Nature Publishing Group, 1336. ISSN: 2041-1723. https://www.nature.com/articles/s41467-025-56495-5 (Feb. 2025).
3. Giuliano, G., Kang, S. & Yuan, Q. Agglomeration economies and evolving urban form. *The Annals of Regional Science* **63,** 377–398 (2019).
4. Bustos, S., Gomez, C., Hausmann, R. & Hidalgo, C. A. The Dynamics of Nestedness Predicts the Evolution of Industrial Ecosystems. *PLOS ONE* **7,** e49393 (2012).
5. Hidalgo, C. A., Klinger, B., Barabási, A.-L. & Hausmann, R. The Product Space Conditions the Development of Nations. *Science* **317.** Publisher: American Association for the Advancement of Science, 482–487. https://www.science.org/doi/10.1126/science.1144581 (July 2007).
6. Lucchini, L. *et al.* Socioeconomic disparities in mobility behavior during the COVID-19 pandemic in developing countries. *EPJ Data Science* **14.** Number: 1 Publisher: Springer Berlin Heidelberg, 25. ISSN: 2193-1127. https://epjds.epj.org/articles/epjdata/abs/2025/01/13688_2025_Article_532/13688_2025_Article_532.html (Dec. 2025).
7. Hidalgo, C. A. Economic complexity theory and applications. *Nature Reviews Physics* **3,** 92–113. ISSN: 2522-5820. https://www.nature.com/articles/s42254-020-00275-1 (Jan. 2021).
8. Hidalgo, C. A. & Hausmann, R. The building blocks of economic complexity. *Proceedings of the National Academy of Sciences* **106,** 10570–10575. ISSN: 0027-8424, 1091-6490. https://pnas.org/doi/full/10.1073/pnas.0900943106 (June 2009).
9. Balland, P.-A. *et al.* The new paradigm of economic complexity. *Research Policy* **51,** 104450. ISSN: 0048-7333 (2022).
