# Supplementary material for: The WorkReach model for urban work location choices through economic complexity and informality
Source: Nat Commun. 2026 Oct 7;17:11422. doi: 10.1038/s41467-026-78293-3 (PMC13737282; doi:10.1038/s41467-026-78293-3)
Supplement: Supplementary file 2 [file 41467_2026_78293_MOESM2_ESM.pdf]

nature portfolio

Corresponding author(s): Marta C. González

Last updated by author(s): Sep 4, 2026

Reporting Summary

Nature Portfolio wishes to improve the reproducibility of the work that we publish. This form provides structure for consistency and transparency in reporting. For further information on Nature Portfolio policies, see our Editorial Policies and the Editorial Policy Checklist.

Statistics

For all statistical analyses, confirm that the following items are present in the figure legend, table legend, main text, or Methods section.

n/a	Confirmed	
☐	☒	The exact sample size (*n*) for each experimental group/condition, given as a discrete number and unit of measurement
☐	☒	A statement on whether measurements were taken from distinct samples or whether the same sample was measured repeatedly
☐	☒	The statistical test(s) used AND whether they are one- or two-sided<br>*Only common tests should be described solely by name; describe more complex techniques in the Methods section.*
☐	☒	A description of all covariates tested
☐	☒	A description of any assumptions or corrections, such as tests of normality and adjustment for multiple comparisons
☐	☒	A full description of the statistical parameters including central tendency (e.g. means) or other basic estimates (e.g. regression coefficient) AND variation (e.g. standard deviation) or associated estimates of uncertainty (e.g. confidence intervals)
☐	☒	For null hypothesis testing, the test statistic (e.g. *F*, *t*, *r*) with confidence intervals, effect sizes, degrees of freedom and *P* value noted<br>*Give P values as exact values whenever suitable.*
☐	☒	For Bayesian analysis, information on the choice of priors and Markov chain Monte Carlo settings
☐	☒	For hierarchical and complex designs, identification of the appropriate level for tests and full reporting of outcomes
☐	☒	Estimates of effect sizes (e.g. Cohen's *d*, Pearson's *r*), indicating how they were calculated

Our web collection on statistics for biologists contains articles on many of the points above.

Software and code

Policy information about availability of computer code

Data collection	No custom code was used by the authors to collect raw data; all inputs are pre-existing (secondary) data obtained from external providers and official registries: anonymized location-based-services (LBS) mobility traces from Quadrant (a commercial LBS data provider); synthetic mobility data from the Replica platform; census data from INEGI (Mexico, 2020), IBGE (Brazil, 2010) and the U.S. Census Bureau (ACS 5-year and LEHD); and employment data from DENUE (Mexico), RAIS (Brazil) and Replica (U.S.).
Data analysis	Home and work locations were inferred from the raw LBS pings using custom BigQuery SQL scripts implementing a circadian-rhythm heuristic, available at https://github.com/ollin18/gc-lbs. All subsequent analyses were performed in Python 3.12.3 with custom code implementing the WorkReach model, available at https://github.com/humnetlab/workreach. Libraries: NumPy 1.26.4, SciPy 1.16.0 (optimize.minimize, L-BFGS-B), pandas 2.2.3, GeoPandas 1.0.1, Shapely 2.0.6, h3-py 4.2.2 (Uber H3 hexagonal grid), NetworkX 3.4.2 (product-space networks), econci (economic complexity calculations; the package does not publish a version number), Matplotlib 3.9.2 and seaborn 0.13.2.

For manuscripts utilizing custom algorithms or software that are central to the research but not yet described in published literature, software must be made available to editors and reviewers. We strongly encourage code deposition in a community repository (e.g. GitHub). See the Nature Portfolio guidelines for submitting code & software for further information.

Data

Policy information about availability of data

All manuscripts must include a data availability statement. This statement should provide the following information, where applicable:

- Accession codes, unique identifiers, or web links for publicly available datasets
- A description of any restrictions on data availability
- For clinical datasets or third party data, please ensure that the statement adheres to our policy

The aggregated commuting flows and the derived $\ECI$ and informality indices at the H3 zone level generated in this study are available in the project repository at https://github.com/humnetlab/workreach and archived at Zenodo (https://doi.org/10.5281/zenodo.21924441). The raw location-based services traces for Mexico City and Rio de Janeiro were obtained under a commercial license from Quadrant that does not permit redistribution and can be licensed from that provider. The Replica synthetic mobility and employment data for the Bay Area and Los Angeles were accessed under the agreement Replica holds with the University of California, Berkeley, which grants access to researchers affiliated with the university; other researchers can request access from Replica. The census and employment data used in this study are publicly available from the national statistical agencies, namely DENUE and the 2020 population census from INEGI for Mexico City, RAIS and the 2010 demographic census from IBGE for Rio de Janeiro, and the American Community Survey and the Longitudinal Employer-Household Dynamics data from the U.S. Census Bureau for the Bay Area and Los Angeles.

Research involving human participants, their data, or biological material

Policy information about studies with human participants or human data. See also policy information about sex, gender (identity/presentation), and sexual orientation and race, ethnicity and racism.

Reporting on sex and gender	Sex and gender were not collected or used. The mobility data are anonymized and aggregated to sub-city zones; no individual-level sex or gender information was obtained or analysed, and results are not disaggregated by sex or gender. This information was not collected.
Reporting on race, ethnicity, or other socially relevant groupings	The socially relevant categorization variable used is LABOR INFORMALITY, represented as a residential informality rate per zone. It is derived from official census/administrative data using context-specific definitions: Mexico (INEGI 2020) — absence of affiliation to social-security/health services; Brazil (IBGE 2010, AEP scale) — employees without a formal work permit ("sem carteira assinada") or self-employed not contributing to social security; United States — workers present in ACS but absent from LEHD payroll records (Graham & Ong method). Informality is used as a socioeconomic labor- market attribute and is NOT a proxy for race or ethnicity. Potential confounding is addressed by entering covariates jointly in the utility function and by robustness analyses (income/wealth proxies; sectoral relatedness) reported in the Supplementary Information.
Population characteristics	Analyses are at the aggregate sub-city-zone level (population, ECI^emp, residential informality rate); no individual-level covariates were used. See answers above.
Recruitment	No participants were recruited. The study uses pre-existing, anonymized, passively collected mobility traces (smartphone app users who consented to location sharing via Quadrant's terms of service), a synthetic mobility dataset (Replica), and official census/employment registries. Self-selection bias: LBS samples over-represent smartphone users and may under-represent some demographic groups; we mitigate this by applying population expansion factors, by requiring inferred home/work consistency across two years, and by cross-validating Mexico City flows against the official Origin Destination Survey (SI). The synthetic Replica data are calibrated to official statistics.
Ethics oversight	This research used only de-identified, aggregated secondary data (anonymized mobility traces and publicly available census and employment statistics) and did not involve interaction with human participants or access to identifiable personal information. It was therefore determined to be exempt from review by the University of California, Berkeley Institutional Review Board (Office for the Protection of Human Subjects).

Note that full information on the approval of the study protocol must also be provided in the manuscript.

Field-specific reporting

Please select the one below that is the best fit for your research. If you are not sure, read the appropriate sections before making your selection.

☐ Life sciences ☒ Behavioural & social sciences ☐ Ecological, evolutionary & environmental sciences

For a reference copy of the document with all sections, see nature.com/documents/nr-reporting-summary-flat.pdf

Behavioural & social sciences study design

All studies must disclose on these points even when the disclosure is negative.

Study description	Quantitative, observational, cross-sectional study based on existing datasets. We build a computational discrete-choice (random-utility) model of workplace location choice from aggregate origin–destination commuting flows. No experimental manipulation; coefficients reflect systematic associations in cross-sectional flows.

Research sample	Existing datasets for four metropolitan areas comparable in population scale: Mexico City and the municipality of Rio de Janeiro (Latin America) and Los Angeles and the San Francisco Bay Area (United States). Units of analysis are sub-city zones (Uber H3 hexagons: resolution 8 for Mexico City, resolution 7 for the U.S. cities; native AEP polygons for Rio de Janeiro) and the commuting flows between them. The sample represents the commuting populations of these metros; the four were chosen for comparable population size and contrasting labor-informality contexts (high-informality Latin American vs. lower-informality U.S. cities).
Sampling strategy	No survey sampling and no sample-size calculation. We use the complete observed commuting network in each city (all zones and all origin–destination links passing the inclusion thresholds below); the analysis is therefore a census of observed flows rather than a drawn sample. Sample sizes (Table 1) are large (tens of thousands to hundreds of thousands of OD links per city) and sufficient for stable maximum-likelihood estimation, as confirmed by bootstrap confidence intervals.
Data collection	Data were not collected by interaction with participants. For Mexico City and Rio de Janeiro, anonymized LBS ping data were obtained from Quadrant (a single commercial LBS data provider); home and work locations were inferred from these raw pings using custom BigQuery SQL scripts implementing a circadian-rhythm heuristic (https://github.com/ollin18/gc-lbs). For Los Angeles and the Bay Area, Replica's synthetic average-weekday commuting data were used. Census and employment attributes were merged from official registries. No researcher was present during data generation, and data processing was blind to the study hypothesis.
Timing	Mobility: March–May 2023 and March–May 2024 (six months total) for Mexico City and Rio de Janeiro; Spring 2023 for the Replica U.S. data. Census: 2020 (Mexico, INEGI), 2010 (Brazil, IBGE), and ACS 2019-2023 5-year estimates (United States). Spatial scale: metropolitan/city extent, analysed at sub-city H3-zone resolution.
Data exclusions	Exclusion criteria were pre-established. We discarded origin–destination pairs with fewer than five observed flows and origins with fewer than ten total outgoing flows; zones with zero population or missing ECI^emp / informality values were dropped; self-loops (home = work zone) were removed. Expansion factors were applied to flows before model fitting.
Non-participation	Exclusion criteria were pre-established. We discarded origin–destination pairs with fewer than five observed flows and origins with fewer than ten total outgoing flows; zones with zero population or missing ECI^emp / informality values were dropped; self-loops (home = work zone) were removed. Expansion factors were applied to flows before model fitting.
Randomization	Not applicable. This is an observational study with no allocation of units into experimental groups. Covariates (distance, economic complexity, informality) are entered directly into the utility function rather than controlled by randomization. Robustness to additional covariates is assessed in the Supplementary Information: we re-estimate the model adding (i) income and wealth proxies as an alternative to economic complexity, (ii) sectoral relatedness between home and work zones, and (iii) diversity and ubiquity as decompositions of economic complexity; we also verify that economic complexity and residential informality are empirically distinct (near-zero correlation in the U.S. cities, moderate in the Latin American cities) and that the zone–sector matrices underlying the complexity index are nested in all four cities. These checks confirm that the main associations are not artefacts of the specific covariates chosen.

Reporting for specific materials, systems and methods

We require information from authors about some types of materials, experimental systems and methods used in many studies. Here, indicate whether each material, system or method listed is relevant to your study. If you are not sure if a list item applies to your research, read the appropriate section before selecting a response.

Materials & experimental systems

n/a	Involved in the study
☒	☐ Antibodies
☒	☐ Eukaryotic cell lines
☒	☐ Palaeontology and archaeology
☒	☐ Animals and other organisms
☒	☐ Clinical data
☒	☐ Dual use research of concern
☒	☐ Plants

Methods

n/a	Involved in the study
☒	☐ ChIP-seq
☒	☐ Flow cytometry
☒	☐ MRI-based neuroimaging

Plants

Seed stocks	*Report on the source of all seed stocks or other plant material used. If applicable, state the seed stock centre and catalogue number. If plant specimens were collected from the field, describe the collection location, date and sampling procedures.*
Novel plant genotypes	*Describe the methods by which all novel plant genotypes were produced. This includes those generated by transgenic approaches, gene editing, chemical/radiation-based mutagenesis and hybridization. For transgenic lines, describe the transformation method, the number of independent lines analyzed and the generation upon which experiments were performed. For gene-edited lines, describe the editor used, the endogenous sequence targeted for editing, the targeting guide RNA sequence (if applicable) and how the editor was applied.*
Authentication	*Describe any authentication procedures for each seed stock used or novel genotype generated. Describe any experiments used to assess the effect of a mutation and, where applicable, how potential secondary effects (e.g. second site T-DNA insertions, mosiacism, off-target gene editing) were examined.*
