# Supplementary material for: The WorkReach model for urban work location choices through economic complexity and informality
Source: Nat Commun. 2026 Oct 7;17:11422. doi: 10.1038/s41467-026-78293-3 (PMC13737282; doi:10.1038/s41467-026-78293-3)
Supplement: Supplementary file 3 [file 41467_2026_78293_MOESM3_ESM.pdf]

nature portfolio

Peer Review File

The WorkReach model for urban work location choices through economic complexity and informality

Corresponding Author: Professor Marta González

This manuscript has been previously reviewed at another journal that is not operating a transparent peer review scheme. The manuscript was considered suitable for publication without further review at Nature Communications.

This file contains all reviewer reports in order by version, followed by all author rebuttals in order by version.

Version 0:

Reviewer comments:

Reviewer #1

(Remarks to the Author)
Review of "WorkReach: Modeling Urban Work Location Choices Through Economic Complexity, Informality, Mobility Data"
Summary
This paper makes important contributions to understanding urban commuting patterns. The study of informality its relationship to commuting behavior is both timely significant, particularly given limited research on informal labor in high-income countries. Including cities from the Global South (Mexico City Rio Janeiro) alongside U.S. cities provides valuable comparative insights. The key empirical finding—that vulnerable populations in Latin American cities endure longer commutes to reach high-quality opportunities, while in U.S. cities the longest commutes are undertaken by affluent suburban workers—is genuinely interesting policy-relevant. Methodologically, the transition weight function $w\_ij(\tau,k)$ is a worthwhile innovation. The authors also benchmark their model against established alternatives, showing it is not necessarily worse. However, several concerns regarding theoretical foundations, model specification, interpretation of results should be addressed before this work can meet the standards of Nature Communications.

Major Comments
Comment 1: Behavioral Plausibility the Choice of ECI over Wage
The paper's central premise—that workers make commuting decisions based on the Economic Complexity Index (ECI) of destinations—lacks behavioral plausibility. Workers do not observe "ECI" when evaluating job opportunities; they observe wages, benefits, job security, working conditions. Wage is the most direct salient measure of job quality, yet it is absent from the utility function.
There exists substantial literature on wage-distance trade-offs in commuting decisions that is largely ignored here. Manning (2003) demonstrates how monopsony power in labor markets affects workers' willingness to commute for higher wages, while Le Barbanchon et al. (2021) show significant gender differences in how workers trade commuting time for wage premiums. The principle of parsimony suggests using wage data would be both simpler more behaviourally grounded than relying on ECI that workers cannot directly observe.
Suggestions: First, run the model using average wage at the destination level as the primary measure of job quality rather than ECI. If fine-grained regional wage data are unavailable, industry-level wages should at minimum be accessible could be spatially aggregated. Second, run the model with both ECI wage as simultaneous controls to assess whether ECI captures variance in commuting patterns beyond what wage explains. Third, justify theoretically why ECI should matter independent of or more than wage, articulating what behavioral mechanism makes workers responsive to an aggregate complexity index.

Comment 2: Agglomeration Benefits as Mechanism
One plausible justification for including ECI—which the paper does not adequately develop—is that it may proxy for agglomeration externalities not captured by current wages alone. These could include learning opportunities from working alongside skilled professionals, knowledge spillovers across firms industries, network benefits that enhance future career prospects, signals of labor market thickness job security. If workers are forward-looking, they might accept lower current wages in high-ECI areas because these locations offer greater human capital accumulation or future wage growth. However, the paper does not articulate this argument.

Suggestions: Explicitly discuss agglomeration theory as a potential mechanism linking ECI to worker utility, engaging with the urban economics literature on agglomeration economies thick labor markets (Moretti 2011; Glaeser & Gottlieb 2009). Acknowledge the limitation that ECI's indirect observability makes it a weaker behavioral primitive than wage, even if it proxies for important latent factors.

Comment 3: Skill Relatedness the Necessity of Long Commutes
The paper interprets long-distance commuting as workers willingly trading distance for higher-quality opportunities (high ECI). However, an alternative interpretation is that workers commute long distances because they cannot find suitable jobs locally—their skills don't match the available opportunities near their residence.
This perspective connects to the economic complexity literature's own emphasis on skill relatedness. The product space network the authors construct (Fig. 2b) encodes which industries share capabilities, yet this information is never used to test whether skill-relatedness between origin destination industries affects commuting patterns. If a worker lives in an area specialized in manufacturing but possesses skills suited for information services, they may have no choice but to commute to distant job centers.
The expected relationship with commuting flows is theoretically ambiguous. On one hand, higher skill relatedness between origin destination industries might increase flows because workers trained in one location possess transferable skills valued in related industries elsewhere. On the other hand, lower skill relatedness might force longer commutes precisely because workers cannot find matching opportunities locally. Regardless of direction, testing this relationship would clarify whether commuting patterns reflect willing pursuit of opportunity versus compensation for local skill mismatch.
Suggestion: Test whether skill relatedness between origin destination industries predicts commuting flows. Calculate a skill-relatedness measure for each origin-destination pair based on their industry compositions sector proximities (using the product space proximity matrix from Fig. 2b), then test whether including this variable improves model fit or changes the interpretation of the ECI coefficient.

Comment 4: Clarifying the Correlational Nature of the Analysis
The paper frequently employs causal language when describing correlational relationships. For example, stating that "a reduction of economic complexity of 1% in a work location in the Bay Area would translate in a 0.13% reduction in the probability of a commuter to select that specific location" (p. 13) implies causality. Similarly, describing "substitution rates elasticities, which quantify how individuals value different work attributes" (p. 12) suggests the model reveals causal preferences. However, the analysis is descriptive correlational.
Suggestions: State clearly in the introduction that the analysis is correlational that the model describes observed patterns rather than identifying causal mechanisms. Revise causal language throughout. Add a limitations section acknowledging that results should be interpreted as suggestive of behavioral patterns rather than definitive evidence of preferences.

Comment 5: Clarifying the "Rational Agents" Assumption
The paper invokes "rational agents" claims to be "grounded in discrete choice theory" (p. 2), but the relationship between Random Utility Models (RUM) rationality requires clarification. In neoclassical economics, rationality typically means complete transitive preferences with maximization of a stable utility function. Moreover, behavioral economics has moved substantially beyond pure rationality, incorporating bounded rationality satisficing (Simon 1955), reference dependence loss aversion (Kahneman & Tversky 1979), social preferences. Simply adopting utility maximization does not make the model "behaviorally founded."
Suggestions: Clarify what "rational" means in this context. If you mean "choices follow a stochastic utility function," say so explicitly. Acknowledge that real workers may satisfice rather than optimize, may be constrained by imperfect information, or may face psychological or social costs of long commutes not captured in the utility function. Rather than claiming behavioral foundations, acknowledge that the model provides a useful first-order approximation of aggregate commuting patterns under the assumption that workers behave "as if" maximizing utility.

Comment 6: Clarifying the Nature Interpretation of ECI
The paper states that "Economic complexity, a measure of the diversity sophistication of a region's economy, serves as a useful proxy for the economic quality of work opportunities" (p. 3). This claim requires more careful justification. Technically, ECI is derived from spectral decomposition of a matrix encoding product co-occurrences—essentially the second eigenvector of the normalized Laplacian. At its core, it is a clustering algorithm that identifies structure in the bipartite network of locations industries (Mealy et al. 2019).
The labeling of "high" versus "low" complexity is based on empirical correlations with GDP per capita at the country level, how was the labelling done here?
Suggestions: Be explicit that ECI is a network-derived clustering measure, not a directly observed economic fundamental. Clarify the labeling convention: how did you define "high" versus "low" ECI within cities? Make this transparent. Soften the claim that ECI proxies for job quality, acknowledging that ECI may capture dimensions of job quality related to industry structure though its precise relationship to worker outcomes requires further investigation.

Comment 7: Statistical Significance Model Validation
The paper does not report standard errors or confidence intervals for the estimated beta coefficients (Table 2), making it difficult to assess whether differences between cities are statistically significant. While confidence intervals are provided for marginal rates of substitution elasticities, the underlying parameter estimates lack measures of uncertainty.
The model benchmarking (Section 2.3.3, Figure 4) evaluates goodness of fit on the same data used for parameter estimation without train-test splitting. This does not provide a fair assessment of out-of-sample predictive performance risks overfitting, particularly when comparing models with different numbers of parameters. The paper should specify how many parameters each benchmark model has—WorkReach seems to have five (β_distance, β_ECI, β_informality, τ, k), but the parameter counts for comparison models are not stated.

Examining prediction error correlations across models would provide insight into what WorkReach captures beyond traditional spatial interaction models. If errors are uncorrelated with benchmark models, this suggests the utility-based approach explains distinct aspects of commuting behavior. High correlation would indicate all models struggle with the same flows.
Suggestions: Report standard errors or bootstrap confidence intervals for all coefficients in Table 2. Implement train-test splitting by estimating parameters on a subset of data evaluating predictive performance on held-out observations. Specify the number of parameters for each benchmark model. Analyze error correlations by computing residuals for each model examining their correlation structure to understand what additional information WorkReach provides.

Minor Comments
Table 2 should show standard errors for the beta coefficients, their statistical significance discussed.
The paper uses "six months of data covering March-May 2023 March-May 2024." Why only these months? Are there seasonality effects one should be concerned about? Please justify the time window or discuss potential seasonal biases.
Section S1.2 exponentiates ECI Informality Rate (which are normally distributed) to "induce log-normality," then applies min-max scaling. The motivation for this transformation is unclear. Why is matching the distributional shape of distance necessary? How does this affect interpretation of coefficients? Please provide clearer justification.
The abstract mentions "the U.S. Latin America." It would be clearer to specify "cities in the U.S., Mexico, Brazil.
Typo: "workersexperience" should be "workers experience."
References
Manning, A. (2003). Monopsony in Motion: Imperfect Competition in Labor Markets. Princeton University Press.
Le Barbanchon, T., Rathelot, R., & Roulet, A. (2021). Gender differences in job search: Trading off commute against wage. Quarterly Journal of Economics, 136(1), 381–426.
Moretti, E. (2011). Local labor markets. In O. Ashenfelter & D. Card (Eds.), Handbook of Labor Economics (Vol. 4B, pp. 1237–1313). Elsevier.
Glaeser, E. L., & Gottlieb, J. D. (2009). The wealth of cities: Agglomeration economies spatial equilibrium in the United States. Journal of Economic Literature, 47(4), 983–1028.
Pissarides, C. A. (2000). Equilibrium Unemployment Theory (2nd ed.). MIT Press.
Neffke, F., & Henning, M. (2013). Skill relatedness firm diversification. Strategic Management Journal, 34(3), 297–316.
Simon, H. A. (1955). A behavioral model of rational choice. Quarterly Journal of Economics, 69(1), 99–118.
Kahneman, D., & Tversky, A. (1979). Prospect theory: An analysis of decision under risk. Econometrica, 47(2), 263–291.
Mealy, P., Farmer, J. D., & Teytelboym, A. (2019). Interpreting economic complexity. Science Advances, 5(1), eaau1705.

(Remarks on code availability)

Reviewer #2

(Remarks to the Author)
WorkReach: Modeling Urban Work Location Choices Through Economic Complexity, Informality, Mobility Data

The manuscript presents a novel model for linking urban mobility to residential location work indicators. They apply the model to two cities in the USA two cities in Latin America to calibrate the model. Instead of adopting a "black box" approach (for example, using ML techniques), they develop an equation-based model, which incurs the cost of estimation but yields tractable results in return. The contribution is that those equations, already calibrated with data, can be used to analyse distinct scenarios sensitivities.

Although the model offers interesting insights (and the figures are great), I think there are three core weaknesses in their approach to understanding urban mobility.

First, the authors use a person's residence as a fixed element take their work as the variable. But a person can move to a different house. So why look at work as the dynamic part ignore residential mobility? Would a worker from a sector move to another job because it is closer, or would they move to a different house because it is closer to their work? In the US, for example, housing relocation within the same city is above 5% per year, in Mexico City, over 1% of the population moves to a different municipality each year (and a much higher rate if movements inside the same municipality are considered).
See: Sobrino, Jaime. "Housing prices submarkets in Mexico City: A hedonic assessment." Estudios económicos (2014): 57-84.

Second, comparing cities is, of course, challenging, particularly when they are from different countries. I am happy to see that the authors considered cities outside the US for their study, which is quite challenging considering the data discrepancies. However, there are some issues in the way they compare cities their mobility. The Bay Area LA are metropolitan areas, but when they are compared to Mexico City, this was considered by its administrative boundaries not the whole metropolitan area. In the metropolitan area of Mexico City, there are more than 22 million inhabitants, but the study only considers the core part, with 40% of them. the peripheral part of Mexico City is the one with the longest commutes, lower accessibility more informality. I don't think that their four cities are comparable. In the US, most commutes are made by car, but in Mexico City, nearly half of the journeys use some form of public transport. The prominent role of public transport shaping mobility patterns is critical outside the US, but I think it was not considered. The median distance of 4.12 km for

travel in Mexico City is extremely small, particularly since it is estimated that the average commute in the city is close to one hour. The authors claim that "Across the four cities, peripheral locations, especially the western zones of Rio Janeiro, emerge as the most underserved", but the peripheral Mexico City is not part of their study.

Also, when four cities are observed the results are not particularly consistent across all of them, why claim "a universal preference for economically sophisticated areas" as it is written in the abstract discussion? How can four observations, all of them across huge cities, even suggest an idea of universality? Could the authors claim something regarding the mobility of a small city in Indonesia or a village in the mountains in Austria using these results?

Third, I am hesitant regarding the data the process for capturing some aspects of the study. In Mexico City, for example, the diagram in Figure 1 shows that some locations with remarkably low informality are located in remote areas, but have an informality rate of about 30% in Benito Juárez Polanco. People with no insurance could work in some informal sector, but could also be students or retired, so I wonder how the age structure in the city changes their estimates. Regarding the employment data, DENUE only gives formal locations with size ranges (including one that says "above 250 employees"). How is the location of informal jobs determined? The claim that "Workers from high-informality neighborhoods in Mexico City Rio Janeiro are willing to travel farther to reach economically complex job centers, overcoming geographic socioeconomic barriers."

Minor comments.
There are some phrases that sound odd to me:
-Rio Janeiro has the smallest number of links nodes, while Mexico City has the largest, it is worth noting that the number of links corresponds to a complete graph with no self-loops, meaning that there is at least one commute connecting each pair of locations.

-Meaning that the higher the ECI of the work locations the more attractive to workers it becomes, this regardless of the region.

The model seems to persistently under-estimate the most common journeys (Figure 4). With a log scale, this is partly hidden, but without the log scale, this would be far more evident. This is more evident in the Fig. S14-17. I wonder how can this be fixed.

Equations need punctuation marks.

(Remarks on code availability)
The GitHub repository is well-documented commented.

Reviewer #3

(Remarks to the Author)
I co-reviewed this manuscript with one of the reviewers who provided the listed reports. This is part of the Nature Communications initiative to facilitate training in peer review to provide appropriate recognition for Early Career Researchers who co-review manuscripts.

(Remarks on code availability)

Reviewer #4

(Remarks to the Author)
This paper introduces a new method to predict commuting flows by incorporating two relatively neglected dimensions: informal employment economic complexity of urban locations. It tests the performance of the method using mobility data. From both a data methodological perspective, the paper is clearly novel, the four example cities studied allow the authors to showcase cross-city differences in a rich informative way.
Despite these novel aspects, the valuable data collection/processing effort, the solid empirical execution, in my view the paper suffers from oversimplification a lack of careful interpretation, which leads to overly weak conclusions. This places the paper far from universality, despite the rich empirical setting behind it. Below, I collect several points that may help improve the manuscript address these issues.

1) I believe the paper suffers from a misinterpretation of economic complexity in the context of employment across industries urban neighborhoods. In this setting, complexity captures the sophistication of the local employment structure, or perhaps the sophistication of the local job market. However, high ECI values in this context clearly do notimply higher “job quality” or more “complex job opportunities.”
This lack of a clear interpretation makes the reader suspect that the empirical exercise is motivated primarily by data availability rather than by a well-articulated conceptual argument supporting variable selection.

2) From an empirical standpoint, this interpretation could be supported by robustness checks examining how income, pure

job concentration, the share of higher-educated workers, or land-use patterns relate to higher ECI values. This would help validate the use of ECI (i.e., show that it captures something otherwise unobserved) support a more accurate interpretation of the results.

3) The relationship between ECI commuting, as well as between ECI informal employment, is not clearly articulated. My intuition is that it is not ECI per se that drives informal workers, but rather the demand for relatively less-qualified labor or for workers willing to accept lower pay or harsher working conditions (among several possible mechanisms).
One possible explanation is that places with higher ECI offer a more diverse set of opportunities, creating a safer environment in which informal workers may have access to a larger pool of jobs, or in which switching jobs is easier due to localized information on job opportunities. Clarifying this relationship is, in my view, key to the paper. I can accept the argument that informality ECI represent independent dimensions, but in that case this interpretation should be made explicit supported as clearly as possible.
4) Why does the combination of high ECI high informality generate the highest utility for commuters? An illustrative example or stylized scenario would be very helpful to make this mechanism more intuitive.

5) The paper does a very poor job of engaging with the existing literature that applies economic complexity measures at the intra-city scale. Some of these studies even use mobility data to explain the role of ECI (e.g., Juhász et al., 2023). Below are a few relevant references that should be discussed:
Juhász et al. (2023) Amenity complexity urban locations of socio-economic mixing. EPJ Data Science
Kim et al. (2024) Redefining urban centrality: Integrating economic complexity indices to central place theory. arXiv:2407.19762
Rossi Mori et al. (2025) Time-space dynamics of income segregation in the city of Milan. PNAS Nexus
Chen et al. (2026) Neighborhood clusters citywide technological diversification. Research Policy

6) Figure 2c suggests that intra-city economic complexity measures are more strongly correlated in U.S. cities than in Latin American megacities. I suspect this reflects differences in development, wealth, urban form, regulation, institutions, other contextual factors.
It would be useful to explicitly compare the contexts of these cities provide basic statistics on socio-economic characteristics such as income, wealth, dominant commuting modes, etc., rather than leaving this as speculation.

7) Economic complexity measures are known to perform reliably when the underlying binary matrix exhibits a nested structure (see Bustos et al. 2012 PLOS One). Showing the four underlying matrices would substantially strengthen the credibility of the analysis.

8) The network of Figure 2b appears almost fully connected from a visual perspective, yet it is interpreted as showing that "highly complex sectors tend to cluster closely in the network, while less complex sectors do the same." Applying an MST-based approach or a disparity filter to retain only statistically significant ties would help the visualization convey this message more clearly.

9) In Figure 2b, the colorbar label is very close to panel (c), making it easy to miss that panel (c) is not related to PCI. This should be corrected.

10) From the text, we learn that informality ECI are weakly correlated. This is an important result for the paper, as it suggests that the two variables capture largely independent properties of urban locations. Please highlight this explicitly using a heatmap or a standard correlation table.

11) It is problematic that variable names such as ECI PCI are used in contexts that differ substantially from their original definitions. I strongly suggest using alternative terminology that better reflects this novel use case, such as "neighborhood complexity" "industry complexity," analogous to ECI PCI. This approach is consistent with prior work, including Juhász et al. (2023, EPJ Data Science).

12) ECI reflects not only industrial diversity at urban locations but also ubiquity. Ubiquity likely plays a major role in commuting: retail jobs, for example, require less specialized skills are spatially widespread to ensure proximity to customers, which likely reduces commuting distances. At the same time, ubiquity may be closely linked to informality. Please provide robustness checks demonstrating that the ECI measure captures something distinct from pure diversity ubiquity.

13) On page 12, the paper states: "The coefficient (β_informality) is negative in the Bay Area Los Angeles, which indicates that high-informality areas in these cities are less prone to travel long distances for high-ECI work alternatives." This interpretation is difficult to reconcile with Figure 2e, where the high-informality–high-complexity group in these cities exhibits the highest median commuting distance. Please clarify how these results should be interpreted jointly.

14) Based on Figure 5 alone, it is unclear what the "low" "high" groups refer to. Please revise the figure to make it self-contained interpretable without relying on the main text.

(Remarks on code availability)
I reviewed the code I found it to be well structured, thoughtfully organized, clearly documented.

Version 1:

Reviewer comments:

Reviewer #1

(Remarks to the Author)
We thank the authors for the thorough constructive response. Comments other than 1 3 have been fully addressed. With respect to comments 1 3, we consider the new analyses on wages (S4.5), skill relatedness (S5), the informality–distance interaction to add substantially to the paper. They are interesting in their own right show that wages, skill relatedness, ECI each carry independent predictive information about commuting flows, with their relative contributions varying systematically across cities.
The study is interesting speaks to several distinct phenomena at once. Our remaining concern is that the current framing pushes a single ECI/pull narrative, when there is no evidence in the paper that this is the dominant mechanism. In fact, we think there is something more interesting in the results: the joint evidence for multiple co-existing forces shaping commuting behaviour.
Specifically, the new analyses show:

A wage / income-wealth proxy carries comparable predictive information to ECI, outperforming it in two of four cities (Table S11). We recognise the wage–distance trade-off is well established in labour economics may not feel novel to report; in this paper's context, however, it is informative as a benchmark for how much of the ECI effect overlaps with simple compensation, even if a direct apples-to-apples comparison of behavioural magnitudes is not straightforward given the different scales of the variables.
Skill relatedness is negative highly significant in all four cities, the ECI+SR specification outperforms WorkReach everywhere (Tables S13–S15). This is less commonly reported in the commuting literature we think it is a new finding worth foregrounding.
In the joint ECI+SR specification (Table S14), β_{ECI} attenuates substantially in the Latin American cities (Mexico City: 4.22 to 1.05; Rio: 6.60 to 0.94), β_{inf} flips sign in both (Mexico City: +10.25 to −4.62; Rio: +4.27 to −1.13). The empirical anchor of the discussion section's policy framing, namely high-informality workers commuting to high-ECI zones, does not survive controlling for skill relatedness.

Together, these results are consistent with at least three distinct mechanisms that the cross-sectional data cannot fully disentangle: pull from agglomeration complexity, pull from compensation, push from local skill mismatch. The β_{inf} sign flip in particular is consistent with workers in Latin American peripheries commuting out because local options are limited, as much as being drawn in by central-zone complexity.
We note that the authors' own MSR framework (Section 2.3.2) is well suited to expressing these trade-offs in comparable behavioural units, such as kilometres per unit of ECI, per unit of SR, or per unit of wage. This would let readers see directly how the forces compare on a common footing, which is otherwise difficult given that the raw coefficients sit on different scales.
We therefore believe the current narrative, focused on ECI alone, does not report the full story the analyses support. We suggest the following:
(1) Provide evidence that the ECI/pull interpretation is the dominant mechanism. This would justify retaining ECI as the central narrative. Approaches that would help include: a local-job-availability control at the origin (push effects should attenuate ECI SR coefficients if mismatch is the driver); stratification by local labour-market tightness (pull predicts uniform ECI effects, push predicts stronger effects where local options are scarce); sector-specific submodels (pull predicts stronger ECI effects in tradable, high-skill sectors); or decomposing ECI into specific agglomeration channels (own-sector density, sector diversity). We acknowledge this may be out of scope, so as an alternative:
(2) Discuss all three mechanisms with equal standing in the abstract, introduction, discussion. Report SR alongside ECI as a co-equal finding, present MSR-based comparisons across ECI, SR, wages so readers can see how the forces compare in behavioural terms, revise language such as workers "valuing" complexity, which presumes a pull interpretation. The β_{inf} sign change in the joint specification deserves explicit discussion given how central the positive β_{inf} finding is to the current policy framing.
We think the paper is more credible, not less, for acknowledging the richer picture the analyses reveal.

(Remarks on code availability)

Reviewer #2

(Remarks to the Author)
WorkReach: Modeling Urban Work Location Choices Through Economic Complexity, Informality, Mobility Data

The authors have carefully analysed the comments I provided in an earlier revision. There are still a few weaknesses in the study, but, given the authors' explanation, I think they are part of the data challenges cannot be fixed. For example, the data for Mexico (DENUE) only provides a wide interval for the number of employees in large companies.

I think the figures are a great part of the manuscript convey information very succinctly.

Given the topic, I would like to see a comparison of the informality rate between Mexico City its metropolitan area. I am still unconvinced that considering only a political division is enough for such an analysis, unfortunately, I think that taking two cities based on their political division does not guarantee comparability. For example, taking the central part of Mexico City

(named CDMX) is not the same as taking Manhattan, or the City of London, or Paris proper. If the informality is similar both within beyond the political definition of Mexico City, given its metropolitan area, this would not be problematic. Maybe a histogram (as figure 1, bottom) is sufficient.

Please make sure that equations are expressions that can be read. They need punctuation marks (missing in equations 4, 5, 12, which need a comma after).

(Remarks on code availability)

Reviewer #3

(Remarks to the Author)
I co-reviewed this manuscript with one of the reviewers who provided the listed reports. This is part of the Nature Communications initiative to facilitate training in peer review to provide appropriate recognition for Early Career Researchers who co-review manuscripts.

(Remarks on code availability)

Reviewer #5

(Remarks to the Author)
I read with interest the revised version of the manuscript appreciate the substantial effort made by the authors to address the reviewers' comments. The paper has improved considerably, the analyses are more convincing, the contribution is now clearly articulated more robust. I only have one minor point remaining.
A new skill-relatedness approach is added, which I found to be a very nice valuable robustness check. However, I would suggest renaming it. The original skill-relatedness literature (see Neffke Henning, 2013) connects industries through labor mobility provides an alternative relatedness metric to co-occurrence-based measures such as the product space. More labor flow than expected signals the reusability of skills between industries , thereby making them “skill-related”. Please consider choosing a different name to avoid confusion with this established literature. I think the current solution is, in essence, closer to the “complementarity” measure suggested by Balland Boschma (2021).

Subject to this small revision, I believe the manuscript is suitable for publication.

References
Neffke, F., & Henning, M. (2013). Skill relatedness firm diversification. Strategic Management Journal, 34(3), 297–316.
Balland, P.-A., & Boschma, R. (2021). Complementary interregional linkages Smart Specialisation: An empirical study on European regions. Regional Studies, 55(6), 1059–1070.

(Remarks on code availability)

Version 2:

Reviewer comments:

Reviewer #1

(Remarks to the Author)
The authors have not addressed my comment but circumvented it. I asked that the co-existing mechanisms be reported with equal standing in the abstract, introduction, discussion; the response instead defends the model specification, which I never asked them to change. My concern is that the paper pursues a narrative rather than reporting all of its evidence: sectoral relatedness outperforms WorkReach in all four cities, the informality coefficient flips sign once it is included, yet neither result reaches the abstract or introduction. These belong in the main text, no change to the model is needed to put them there.

(Remarks on code availability)

Reviewer #2

(Remarks to the Author)
The authors have made a great contribution with this manuscript have followed all comments suggestions I wrote during the revision.

I truly appreciate the analysis of informality rates between CDMX the ZMVM, as well as the efforts to make this more

transparent. Additionally, I appreciate that the authors included an additional visualisation (Supplementary Figure 14) for this specific topic.

(Remarks on code availability)

Reviewer #3

(Remarks to the Author)
I co-reviewed this manuscript with one of the reviewers who provided the listed reports. This is part of the Nature Communications initiative to facilitate training in peer review to provide appropriate recognition for Early Career Researchers who co-review manuscripts.

(Remarks on code availability)

Reviewer #5

(Remarks to the Author)
The authors have addressed all of my previous comments carefully thoroughly. The manuscript has improved substantially since the initial submission, I congratulate the authors on their work.

(Remarks on code availability)

Open Access This Peer Review File is licensed under a Creative Commons Attribution 4.0 International License, which permits use, sharing, adaptation, distribution and reproduction in any medium or format, as long as you give appropriate credit to the original author(s) and the source, provide a link to the Creative Commons license, and indicate if changes were made.
In cases where reviewers are anonymous, credit should be given to 'Anonymous Referee' and the source.
The images or other third party material in this Peer Review File are included in the article's Creative Commons license, unless indicated otherwise in a credit line to the material. If material is not included in the article's Creative Commons license and your intended use is not permitted by statutory regulation or exceeds the permitted use, you will need to obtain permission directly from the copyright holder.
To view a copy of this license, visit https://creativecommons.org/licenses/by/4.0/

Point-by-Point Response to Reviewer Comments

WorkReach: Modeling Urban Work Location Choices Through Economic Complexity, Informality, and Mobility Data

Ollin D. Langle-Chimal, Steffen Knoblauch, Marta C. González

May 1, 2026

Point-by-Point Responses

We thank the reviewers for their thorough and constructive feedback on our manuscript. Each comment is reproduced verbatim in a quoted block, followed by an italicized summary of how we read it and our response. All new or modified text in the manuscript is highlighted in blue. Before proceeding, we highlight a cross-cutting methodological change that affects several responses.

Updated ECI^{emp} **computation.** We recomputed ECI^{emp} by assigning individual job records directly to H3 hexagonal zones based on establishment coordinates, rather than aggregating from census-tract-level data. Informality rates remain measured at the census-tract level (Census Block Groups, AGEBs, or AEPs) and are area-weighted to H3 zones; Rio de Janeiro retains its original AEP boundaries for both variables. We also excluded the "Other Services" NAICS category to improve interpretability in the product-space framework, particularly for the counterfactual uplift analysis. This consistent spatial resolution enables the new product-space analyses reported in the SI, including skill relatedness, diversity and ubiquity decompositions, and counterfactual ECI^{emp} uplift scenarios. All core WorkReach results hold under this recomputation; where specific numerical values have changed (e.g., elasticities, correlations), we note this in the relevant response. One notable change is that the cross-city correlation of sector co-location patterns (Figure 2c) now shows equally strong within-region similarity for both U.S. cities ($r = 0.85$) and Latin American cities ($r = 0.86$), whereas the previous aggregation yielded a moderate correlation for the Latin American pair ($r = 0.51$).

Covariate preprocessing. In the original submission, ECI^{emp} and informality were exponentiated before min-max scaling to $[0, 1]$, following the distributional shape of commuting distance. In the revised manuscript, all three covariates enter the utility function linearly after min-max scaling, without any nonlinear transformation. This change simplifies the model specification: each coefficient directly represents the utility change per unit of the scaled covariate, with no intermediate transformation complicating the mapping between raw variable values and their contribution to utility. All results reported in the revised manuscript use this linear preprocessing.

Accessibility analysis. We now standardize both accessibility metrics (distance-weighted and consumer-surplus) using within-city z-scores and present a combined index as the mean of the two z-scores (Figure 5, panel c). In the original submission we used PCA to combine the metrics, but because distance-weighted and consumer-surplus accessibility are negatively correlated in U.S. cities, the first principal component captured the contrast between the two measures rather than their overall level, producing counterintuitive results for zones with low scores on both metrics. The mean of z-scores avoids this issue and provides a directly interpretable combined accessibility measure.

Reviewer 1

Comment 1: ECI vs. Wages as Behavioral Primitive

> *The paper's central premise—that workers make commuting decisions based on the Economic Complexity Index (ECI) of destinations—lacks behavioral plausibility. Workers do not observe "ECI" when evaluating job opportunities; they observe wages, benefits, job security, and working conditions. Wage is the most direct and salient measure of job quality, yet it is absent from the utility function.*
>
> *There exists substantial literature on wage-distance trade-offs in commuting decisions that is largely ignored here. Manning (2003) demonstrates how monopsony power in labor markets affects workers' willingness to commute for higher wages, while Le Barbanchon et al. (2021) show significant gender differences in how workers trade commuting time for wage premiums. The principle of parsimony suggests using wage data would be both simpler and more behaviourally grounded than relying on ECI that workers cannot directly observe.*
>
> *Suggestions: First, run the model using average wage at the destination level as the primary measure of job quality rather than ECI. If fine-grained regional wage data are unavailable, industry-level wages should at minimum be accessible and could be spatially aggregated. Second, run the model with both ECI and wage as simultaneous controls to assess whether ECI captures variance in commuting patterns beyond what wage explains. Third, justify theoretically why ECI should matter independent of or more than wage, articulating what behavioral mechanism makes workers responsive to an aggregate complexity index.*

The reviewer questions whether economic complexity (ECI) is the right destination attribute and suggests that prevailing wages may be the more natural primitive in a discrete-choice framework, since wages directly enter budget constraints and are the standard destination attribute in commuting models.

We agree that wages are the canonical destination attribute in labor economics, and we have addressed this concern through both theoretical justification and empirical robustness analysis.

Theoretical justification (Introduction). We added text explaining why we prefer ECI over wages as the primary destination attribute. We first note that "wages capture only current compensation," whereas "ECI^{emp}, by contrast, is derived from the productive structure of each zone and therefore reflects location-level advantages that current pay does not internalize." Drawing on agglomeration theory, we explain that zones where diverse industries co-locate "generate productivity externalities from sectoral complementarities, lower job-search costs by concentrating a wider range of employment opportunities, and facilitate human capital accumulation through exposure to a broader set of economic activities" (citing Moretti 2011; Glaeser & Gottlieb 2009). We also cite Manning (2003) and Le Barbanchon et al. (2021) to situate the distance–wage trade-off in the labor economics literature.

Empirical robustness (Results + SI Section S4.5). We estimated three model variants for each city: WorkReach with ECI only, an income/wealth-proxy-only specification, and a combined model including both. For the Bay Area and Los Angeles we use zone-level mean wages from the Replica synthetic employment dataset; for Rio de Janeiro, flow-weighted mean household income from the 2020 Brazilian Census; and for Mexico City, a flow-weighted composite wealth index derived from 16 census indicators. Key findings:

- Likelihood-ratio tests show that each variable is independently significant when added to the other ($p < 0.001$ in all four cities and both directions; Table S12).
- Zone-level correlations between ECI and the income or wealth proxy are moderate ($r = 0.53$ Bay Area, $r = 0.61$ Los Angeles, $r = 0.59$ Mexico City, $r = 0.54$ Rio de Janeiro; Figure S29), confirming that the two measures capture overlapping but distinct dimensions.
- Performance metrics (CPC, r) with bootstrap confidence intervals are reported in Table S11 and Figure S30. In Los Angeles, ECI significantly outperforms the wage proxy; in the Bay Area, the difference is not significant; in the Latin American cities, the income/wealth proxy outperforms ECI, though a circularity caveat applies because the proxy is constructed using the same commuting flows that serve as the dependent variable.

These results demonstrate that ECI and wages are complementary rather than redundant, and that the original WorkReach specification captures behavioral dimensions not reducible to prevailing wages.

Comment 2: Agglomeration Benefits as Mechanism

> *One plausible justification for including ECI—which the paper does not adequately develop—is that it may proxy for agglomeration externalities not captured by current wages alone. These could include learning opportunities from working alongside skilled professionals, knowledge spillovers across firms and industries, network benefits that enhance future career prospects, and signals of labor market thickness and job security. If workers are forward-looking, they might accept lower current wages in high-ECI areas because these locations offer greater human capital accumulation or future wage growth. However, the paper does not articulate this argument.*
>
> *Suggestions: Explicitly discuss agglomeration theory as a potential mechanism linking ECI to worker utility, engaging with the urban economics literature on agglomeration economies and thick labor markets (Moretti 2011; Glaeser & Gottlieb 2009). Acknowledge the limitation that ECI's indirect observability makes it a weaker behavioral primitive than wage, even if it proxies for important latent factors.*

The reviewer asks how workers perceive ECI, noting that it is not directly observable, and suggests that agglomeration economies may be the underlying mechanism through which ECI influences commuting decisions.

We agree that ECI is not directly observed by individual workers and have added text addressing this point in two places:

- **Introduction:** We added sentences grounding ECI in agglomeration theory. The new text explains that high-ECI zones "generate productivity externalities from sectoral complementarities, lower job-search costs [. . .], and facilitate human capital accumulation through exposure to a broader set of economic activities" (citing Moretti 2011; Glaeser & Gottlieb 2009).
- **Discussion (Limitations):** We acknowledge that ECI is an aggregate index proxying for a latent bundle of agglomeration benefits whose individual components remain unidentified in our specification. Decomposing the "ECI effect" into its constituent agglomeration mechanisms is an important direction for future work.

Comment 3: Skill Relatedness and Necessity of Long Commutes

> *The paper interprets long-distance commuting as workers willingly trading distance for higher-quality opportunities (high ECI). However, an alternative interpretation is that*

workers commute long distances because they cannot find suitable jobs locally—their skills don't match the available opportunities near their residence.

This perspective connects to the economic complexity literature's own emphasis on skill relatedness. The product space network the authors construct (Fig. 2b) encodes which industries share capabilities, yet this information is never used to test whether skill-relatedness between origin and destination industries affects commuting patterns. If a worker lives in an area specialized in manufacturing but possesses skills suited for information services, they may have no choice but to commute to distant job centers.

The expected relationship with commuting flows is theoretically ambiguous. On one hand, higher skill relatedness between origin and destination industries might increase flows because workers trained in one location possess transferable skills valued in related industries elsewhere. On the other hand, lower skill relatedness might force longer commutes precisely because workers cannot find matching opportunities locally. Regardless of direction, testing this relationship would clarify whether commuting patterns reflect willing pursuit of opportunity versus compensation for local skill mismatch.

Suggestion: Test whether skill relatedness between origin and destination industries predicts commuting flows. Calculate a skill-relatedness measure for each origin-destination pair based on their industry compositions and sector proximities (using the product space proximity matrix from Fig. 2b), then test whether including this variable improves model fit or changes the interpretation of the ECI coefficient.

The reviewer asks whether the product-space framework can be used to test whether skill overlap between origin and destination predicts commuting patterns, and whether long commutes reflect necessity rather than preference.

We developed a skill-relatedness (SR) extension of WorkReach to address this comment (SI Section S5). Using the product-space proximity matrix, we computed a bilinear skill-relatedness score $\mathrm{SR}_{ij} = \mathbf{c}_i \cdot \mathbf{\Phi} \cdot \mathbf{c}_j$ for each origin–destination pair and added it to the utility function alongside ECI and informality. The analysis covers all four cities.

Key finding: The SR coefficient is negative in all four cities ($\beta_{\mathrm{SR}} = -8.550$ Bay Area; -6.744 Los Angeles; -4.583 Mexico City; -6.756 Rio de Janeiro; Table S14), indicating that workers systematically commute to destinations whose sector composition is *less* related to their home zone's profile. This supports a necessity or skill-mismatch hypothesis: workers travel to zones offering employment in sectors different from those available locally, rather than seeking destinations with similar profiles.

Additional findings:

- Likelihood-ratio tests confirm SR significantly improves fit beyond ECI alone ($p < 0.001$, all cities; Table S15).
- ECI remains independently significant after controlling for SR in three of four cities, supporting the complementary roles of aggregate destination quality and sector-specific skill overlap.
- Within-sector commuting fractions reveal clear regional distinctions (Figure S31): retail dominates in Latin American cities but not in the US, while health care, professional services, and manufacturing show the opposite pattern, reflecting differences in the spatial distribution of jobs across regions. When stratified by origin informality (Figure S32), the negative SR–distance relationship is significant only for high-informality origins in Latin American cities, whereas in US cities it holds across both informality groups.

Although the SR extension improves predictive fit, we retain ECI as the primary covariate for three reasons, discussed in detail in SI Section S5 ("$\mathrm{ECI}^{\mathrm{emp}}$ versus Skill Relatedness: Predictive

and Policy Trade-offs"):

1. **ECI is intrinsic to a destination.** ECI summarizes the diversity and sophistication of a zone's productive structure without reference to any particular origin. SR, by contrast, is a pairwise origin–destination quantity that cannot be attributed to either zone alone. This makes ECI directly amenable to policy simulation: a zone's ECI can be updated after a feasibility-guided intervention and fed back into WorkReach to predict changes in commuting flows (SI Section S8). No comparable single-location lever exists for SR.
2. **ECI captures structural information beyond wages or sector overlap.** SR measures how similar two zones' sector profiles are, while wages reflect current compensation. ECI encodes which sectors co-locate and how that pattern compares across zones, capturing agglomeration externalities and tacit knowledge relevant to long-run development.
3. **Interpretability.** The five-parameter WorkReach model maps each coefficient to a single behavioral quantity. Adding SR as a sixth parameter mixes labor-market mismatch with spatial sorting, complicating the extraction of policy-relevant elasticities.

We therefore report the SR analysis as a robustness check that demonstrates the richness of the economic complexity framework while retaining ECI in the main specification. The full analysis appears in a new Results subsection, a new Methods paragraph, and SI Section S5.

Comment 4: Causal Language

> *The paper frequently employs causal language when describing correlational relationships. For example, stating that "a reduction of economic complexity of 1% in a work location in the Bay Area would translate in a 0.13% reduction in the probability of a commuter to select that specific location" (p. 13) implies causality. Similarly, describing "substitution rates and elasticities, which quantify how individuals value different work attributes" (p. 12) suggests the model reveals causal preferences. However, the analysis is descriptive and correlational.*
>
> *Suggestions: State clearly in the introduction that the analysis is correlational and that the model describes observed patterns rather than identifying causal mechanisms. Revise causal language throughout. Add a limitations section acknowledging that results should be interpreted as suggestive of behavioral patterns rather than definitive evidence of preferences.*

The reviewer notes that the manuscript's language sometimes implies causal relationships where the analysis is cross-sectional and correlational.

We have systematically revised the manuscript to replace causal phrasing with correlational language. Specific changes include:

- Introduction: "a reduction of economic complexity of 1% ... would translate in a 0.13% reduction" was changed to "is associated with a 0.804% reduction." (The numerical change reflects the updated ECI^{emp} computation and preprocessing described at the start of this document, under *Updated* ECI^{emp} *computation* and *Covariate preprocessing*.)
- Results: "how individuals value different work attributes" was changed to "how commuting flows vary with different work attributes."
- Results: "suggesting that workers in areas of high-informality are more willing to travel" was changed to "consistent with workers in areas of high-informality traveling."
- Discussion: "are willing to travel farther" was changed to "travel farther."
- Discussion: "can greatly improve" was changed to "may help expand."

- Discussion (Limitations): New paragraph explicitly stating that the analysis is cross-sectional and correlational, that coefficients reflect systematic associations rather than experimentally identified preferences, and that quasi-experimental or longitudinal designs are needed for causal identification.

Comment 5: Rational Agents Assumption

> *The paper invokes "rational agents" and claims to be "grounded in discrete choice theory" (p. 2), but the relationship between Random Utility Models (RUM) and rationality requires clarification. In neoclassical economics, rationality typically means complete and transitive preferences with maximization of a stable utility function. Moreover, behavioral economics has moved substantially beyond pure rationality, incorporating bounded rationality and satisficing (Simon 1955), reference dependence and loss aversion (Kahneman & Tversky 1979), and social preferences. Simply adopting utility maximization does not make the model "behaviorally founded."*
>
> *Suggestions: Clarify what "rational" means in this context. If you mean "choices follow a stochastic utility function," say so explicitly. Acknowledge that real workers may satisfice rather than optimize, may be constrained by imperfect information, or may face psychological or social costs of long commutes not captured in the utility function. Rather than claiming behavioral foundations, acknowledge that the model provides a useful first-order approximation of aggregate commuting patterns under the assumption that workers behave "as if" maximizing utility.*

The reviewer asks us to clarify the relationship between Random Utility Models (RUM) and rationality, noting that utility maximization alone does not constitute a behavioral foundation, and suggests adopting an "as if" framing.

We agree that our original language overstated the behavioral implications of the RUM framework. We have revised the Abstract and Introduction to clarify that the model captures aggregate commuting patterns consistent with workers behaving *as if* they maximize utility, without assuming full rationality in the neoclassical sense. Specifically:

- Abstract: "reveals a behavioral principle that characterizes how rational agents select work locations" was changed to "reveals how aggregate commuting patterns emerge when workers behave as if maximizing a location-choice utility."
- Introduction: "a utility function that commuters, as rational economic agents, are assumed to maximize" was changed to "a utility function that commuters are assumed to maximize, so that the resulting parameters approximate the aggregate trade-offs reflected in observed commuting patterns."

Comment 6: Nature and Interpretation of ECI

> *The paper states that "Economic complexity, a measure of the diversity and sophistication of a region's economy, serves as a useful proxy for the economic quality of work opportunities" (p. 3). This claim requires more careful justification. Technically, ECI is derived from spectral decomposition of a matrix encoding product co-occurrences—essentially the second eigenvector of the normalized Laplacian. At its core, it is a clustering algorithm that identifies structure in the bipartite network of locations and industries (Mealy et al. 2019).*

The labeling of "high" versus "low" complexity is based on empirical correlations with GDP per capita at the country level, how was the labelling done here?

Suggestions: Be explicit that ECI is a network-derived clustering measure, not a directly observed economic fundamental. Clarify the labeling convention: how did you define "high" versus "low" ECI within cities? Make this transparent. Soften the claim that ECI proxies for job quality, acknowledging that ECI may capture dimensions of job quality related to industry structure though its precise relationship to worker outcomes requires further investigation.

The reviewer asks us to be explicit about the spectral nature of ECI, clarify the labeling convention for "high" versus "low" complexity within cities, and soften the claim that ECI proxies for job quality.

We have expanded the Methods (ECI section) to clarify that ECI corresponds to the eigenvector associated with the largest non-trivial eigenvalue of the zone-space projection of the bipartite network. We explain that its entries partition zones according to their connectivity patterns (analogous to a spectral bisection), where zones with similar entries share denser connections to zones with similar sector structure. We also state that the sign of the eigenvector is fixed by requiring a positive correlation with diversity, following the convention of Hidalgo and Hausmann (2009).

Comment 7: Confidence Intervals, Benchmarking, and Error Analysis

The paper does not report standard errors or confidence intervals for the estimated beta coefficients (Table 2), making it difficult to assess whether differences between cities are statistically significant. While confidence intervals are provided for marginal rates of substitution and elasticities, the underlying parameter estimates lack measures of uncertainty.

The model benchmarking (Section 2.3.3, Figure 4) evaluates goodness of fit on the same data used for parameter estimation without train-test splitting. This does not provide a fair assessment of out-of-sample predictive performance and risks overfitting, particularly when comparing models with different numbers of parameters. The paper should specify how many parameters each benchmark model has—WorkReach seems to have five (β_{dist}, β_{ECI}, β_{inf}, τ, k), but the parameter counts for comparison models are not stated.

Examining prediction error correlations across models would provide insight into what WorkReach captures beyond traditional spatial interaction models. If errors are uncorrelated with benchmark models, this suggests the utility-based approach explains distinct aspects of commuting behavior. High correlation would indicate all models struggle with the same flows.

Suggestions: Report standard errors or bootstrap confidence intervals for all coefficients in Table 2. Implement train-test splitting by estimating parameters on a subset of data and evaluating predictive performance on held-out observations. Specify the number of parameters for each benchmark model. Analyze error correlations by computing residuals for each model and examining their correlation structure to understand what additional information WorkReach provides.

The reviewer notes that Table 2 did not report standard errors or confidence intervals for the estimated coefficients, that the model benchmarking did not use train-test splitting, and suggests analyzing prediction error correlations across models.

We have added 95% confidence intervals for all coefficients, marginal rates of substitution, and elasticities in Table 2. These are computed using a row-wise multinomial bootstrap in which origin-level destination shares are resampled conditional on observed home populations and the full model is re-estimated for each replicate, yielding an empirical distribution of parameters from which percentile intervals are computed. This approach does not require distributional assumptions on the error term and accounts for the discrete, count-based nature of the flow data.

Parameter counts. As requested, we now state the number of free parameters explicitly in the Benchmark Models section: Gravity (4), Extended Radiation (1), BMS Plausible (6), and WorkReach (5).

Train-test splitting. We conducted a stratified 5-fold cross-validation on origin zones. In each fold, 80% of origin zones are used for training and 20% are held out for evaluation, stratified by quartile bins of population, ECI^{emp}, and informality rate. All destination zones remain in the choice set for both train and test origins; only the observed outflow rows of test origins are withheld during parameter estimation, preserving the multinomial logit structure. Stitching the test predictions across all five folds yields a complete out-of-sample prediction for every origin–destination pair.

The train–test performance gap is negligible for all models and cities ($\Delta\text{CPC} \leq 0.001$, $\Delta r \leq 0.02$; SI Section S3.5, Table S5, Figure S23), confirming that the parsimonious parameterizations (1–6 free parameters on 40,000–630,000 OD pairs) are not susceptible to overfitting. We retain the full-data estimates for all reported coefficients, substitution rates, elasticities, and derived analyses (accessibility, counterfactual uplift), since the cross-validation demonstrates that out-of-sample performance is indistinguishable from in-sample performance. We have added a sentence in the Results noting this finding.

Regarding error correlation analysis: we have computed pairwise Pearson correlations between the prediction residuals (observed minus predicted flows) of all four models for each city (SI Section S3.4, Figure S21). Gravity and BMS Plausible share near-identical residual structure ($r = 0.96$–0.99), consistent with the structural similarity of their formulations. WorkReach's residual correlations with the benchmarks vary across cities: highest in Los Angeles ($r = 0.92$–0.95) and lowest in Bay Area ($r = 0.86$–0.92) and Mexico City ($r = 0.66$–0.86), indicating that the inclusion of ECI and informality captures distinct predictive information in these cities. The Extended Radiation model diverges most from all other models, particularly in Mexico City ($r = 0.66$ with WorkReach) and Los Angeles ($r = 0.73$), likely because its intervening-opportunities mechanism produces a qualitatively different error structure from parametric distance decay.

Minor Comments

Table 2 should show standard errors for the beta coefficients, and their statistical significance discussed.

The paper uses "six months of data covering March–May 2023 and March–May 2024." Why only these months? Are there seasonality effects one should be concerned about? Please justify the time window or discuss potential seasonal biases.

Section S1.2 exponentiates ECI and Informality Rate (which are normally distributed) to "induce log-normality," then applies min-max scaling. The motivation for this transformation is unclear. Why is matching the distributional shape of distance necessary? How does this affect interpretation of coefficients? Please provide clearer justification.

The abstract mentions "the U.S. and Latin America." It would be clearer to specify "cities in the U.S., Mexico, and Brazil."

Typo: "workersexperience" should be "workers experience."

The reviewer raises several minor points: (i) justification for the March–May time window, (ii) the exponential transformation of covariates, (iii) specifying "U.S., Mexico, and Brazil" in the abstract, (iv) the "workersexperience" typo, and (v) equation punctuation.

Seasonality and time window. The March–May 2023 and March–May 2024 window reflects the period for which LBS data are available for Mexico City and Rio de Janeiro (U.S. cities use Replica's Spring 2023 synthetic dataset). Our inference pipeline mitigates seasonal bias in two ways. First, we do not model the full set of trips or stops; instead, we extract only the home and work locations for each user based on circadian patterns, requiring a minimum of 14 distinct days for home detection and 8 distinct days for work detection. This filtering retains only users with stable, repeated commuting behavior and discards transient or sporadic visits that would be most affected by seasonal variation. Second, we retain only users whose home and work locations are consistent across both years, further ensuring that the inferred commuting patterns reflect habitual behavior rather than short-term fluctuations. To validate this approach, we compared our LBS-derived OD matrix for Mexico City against the official 2017 Origin-Destination Survey, finding strong agreement (Pearson $r = 0.76$, cosine similarity $= 0.887$; SI Section S2). We have added a clarification of these points in the Data subsection of Variable Construction.

Covariate preprocessing. We agree that the motivation for the exponential transformation was unclear. In the revised manuscript, we have removed it: all three covariates (distance, $\mathrm{ECI}^{\mathrm{emp}}$, and informality) are now min-max scaled to $[0, 1]$ directly, entering the utility function linearly. This addresses the reviewer's concern by eliminating the need to justify the distributional shape matching. The linear specification also simplifies coefficient interpretation, as each β directly represents the utility change per unit of the min-max scaled covariate without an intervening nonlinear transformation. We compared both specifications and found that the linear version yields comparable or slightly improved predictive performance across all cities (see the *Covariate preprocessing* summary at the start of this document). The updated preprocessing is described in the Estimated Coefficients subsection and SI Section S1.2.

Abstract, typo, and equations. We have specified "cities in the U.S., Mexico, and Brazil" in the Abstract, corrected "workersexperience" to "workers experience," and added punctuation marks (commas or periods) to all display equations throughout the manuscript.

Reviewer 2

Comment 1: Residential Mobility

> *First, the authors use a person's residence as a fixed element and take their work as the variable. But a person can move to a different house. So why look at work as the dynamic part and ignore residential mobility? Would a worker from a sector move to another job because it is closer, or would they move to a different house because it is closer to their work? In the US, for example, housing relocation within the same city is above 5% per year, and in Mexico City, over 1% of the population moves to a different municipality each year (and a much higher rate if movements inside the same municipality are considered).*
>
> *See: Sobrino, Jaime. "Housing prices and submarkets in Mexico City: A hedonic assessment." Estudios económicos (2014): 57–84.*

The reviewer raises the question of why we treat the workplace as the variable element and residence as fixed, noting that residential relocations within a city are not negligible (above 5% per year in the U.S. and over 1% across municipalities in Mexico City).

We thank the reviewer for this insightful comment and fully acknowledge that residential mobility is an important dimension of urban dynamics that our framework does not capture. Our study employs a static, cross-sectional approach and does not track longitudinal changes in either residence or workplace, which is a limitation we now state explicitly in the Discussion.

That said, available labor-market statistics support our framing of workplace choice as the more dynamic variable. In the United States, the monthly job-switching rate is approximately 2.2%, which compounds to roughly 23% annually [1], substantially higher than the 5% annual rate of within-city residential moves. In Latin America, first-year job turnover rates are 16.6% in Mexico and 11.3% in Brazil [2], also exceeding residential mobility rates. These figures indicate that, within the time horizons captured by our mobility data, workers are considerably more likely to change jobs than to relocate their homes, providing a strong empirical rationale for treating residence as the anchor and workplace as the choice variable.

We have added a paragraph in the Discussion acknowledging this limitation, citing the supporting evidence, and noting that longitudinal data tracking both residential and workplace transitions would allow a more complete characterization of the joint home-work decision.

Comment 2

> *Second, comparing cities is, of course, challenging, particularly when they are from different countries. And I am happy to see that the authors considered cities outside the US for their study, which is quite challenging considering the data discrepancies. However, there are some issues in the way they compare cities and their mobility. The Bay Area and LA are metropolitan areas, but when they are compared to Mexico City, this was considered by its administrative boundaries and not the whole metropolitan area. In the metropolitan area of Mexico City, there are more than 22 million inhabitants, but the study only considers the core part, with 40% of them. And the peripheral part of Mexico City is the one with the longest commutes, lower accessibility and more informality. I don't think that their four cities are comparable. In the US, most commutes are made by car, but in Mexico City, nearly half of the journeys use some form of public transport. The prominent role of public transport shaping mobility patterns is critical outside the US, but I think it was not considered. The median distance of 4.12 km for travel in*

Mexico City is extremely small, particularly since it is estimated that the average commute in the city is close to one hour. The authors claim that "Across the four cities, peripheral locations, especially the western zones of Rio de Janeiro, emerge as the most underserved", but the peripheral Mexico City is not part of their study.

Part A: City Comparability and Spatial Boundaries

The reviewer questions the comparability of the four cities, noting that the Bay Area is a metropolitan region while Mexico City is defined by its administrative boundaries, which exclude the peripheral municipalities with the longest commutes, lowest accessibility, and highest informality.

We appreciate this important observation. We want to clarify that the use of administrative rather than metropolitan boundaries was not limited to Mexico City; the same criterion was applied to Los Angeles and Rio de Janeiro. The San Francisco Bay Area is the exception, defined as a multi-county metropolitan region because the economic dynamics of that labor market span multiple jurisdictions without a single dominant administrative unit.

The rationale behind these boundary choices was to maintain comparability in population scale (all four areas range from approximately 6 to 10 million inhabitants) and in the number of spatial units used for the analysis (∼200–800 zones). We acknowledge that this approach excludes the wider metropolitan periphery of Mexico City (the Zona Metropolitana del Valle de México). However, the administrative boundaries of Mexico City proper still encompass large, populous areas with pronounced peripheral characteristics, including Iztapalapa, Tlalpan, Xochimilco, and Tláhuac, which collectively represent a substantial share of the city's population and exhibit high informality rates, long commutes, and low accessibility. The internal heterogeneity captured within these boundaries is therefore considerable. We have added a note to the Study Areas section clarifying the boundary rationale, and we thank the reviewer for bringing the reference on housing submarkets in Mexico City to our attention [3].

Part B: Transport Modes and Commute Distance

The reviewer notes that transport modes differ markedly between U.S. and Mexican cities (car-dominated versus public-transport-dependent), and questions the plausibility of the 4.12 km median commuting distance reported for Mexico City, given that the average commute in the city is estimated to be close to one hour.

Regarding transport modes, we agree that modal differences are an important factor and we acknowledge this in the Results when discussing the distance threshold parameter τ, where we note that better highway infrastructure and widespread car access in U.S. cities make longer commutes more feasible. A detailed, mode-specific decomposition of commuting behavior is beyond the current scope of WorkReach but represents a valuable direction for future work.

Regarding the commuting distance, we have added a comprehensive validation analysis in the Supplementary Information (Section S2) that compares our Location-Based Services (LBS) data with Mexico City's official 2017 Origin-Destination Survey (EOD) for CDMX proper. The EOD also provides aggregate statistics for the full Zona Metropolitana del Valle de México (ZMVM). The key findings are:

- The EOD survey does not report trip distances directly; we computed centroid-to-centroid Haversine distances between districts. The resulting median distance is 6.23 km for CDMX proper and 7.39 km for the ZMVM, alongside median travel times of 45 and 50 minutes, respectively. This confirms that short geographic distances coexisting with long commute durations is a structural feature of the city's mobility, not an artifact of our data.

- Our distance estimates are sensitive to spatial resolution. We computed LBS distances at the AGEB (census tract) level and four H3 hexagonal grid resolutions (6 through 9). At H3 resolution 6, where the 35 hexagonal cells covering CDMX are most comparable in number and size to the survey's 16 districts, the mean LBS distance is 7.56 km versus the survey's 7.74 km (a difference of only 0.18 km). Finer resolutions yield shorter distances as they resolve short-range trips that coarser grids merge into single zones.
- The origin-destination matrices derived from our LBS data and from the EOD survey are highly concordant for CDMX proper: Pearson $r = 0.76$ and cosine similarity $= 0.887$.
- All distances reported in our study are Haversine (great-circle) distances, which provide a geometric lower bound on actual road travel distances and naturally underestimate the path lengths experienced by commuters.

Part C: Claim of Universality

> *Also, when four cities are observed and the results are not particularly consistent across all of them, why claim "a universal preference for economically sophisticated areas" as it is written in the abstract and discussion? How can four observations, all of them across huge cities, even suggest an idea of universality? Could the authors claim something regarding the mobility of a small city in Indonesia or a village in the mountains in Austria using these results?*

The reviewer questions whether four cities can support a claim of "universality."

We fully agree with the reviewer. The term "universal" overstates the generalizability of our findings given the scope of the study. We have revised the manuscript throughout, replacing "universal preference" with "consistent preference across the four cities studied" in both the Abstract and the Discussion, and we have removed any language that implies generalization beyond our sample.

Comment 3: Informality Data and Estimation

> *Third, I am hesitant regarding the data and the process for capturing some aspects of the study. In Mexico City, for example, the diagram in Figure 1 shows that some locations with remarkably low informality are located in remote areas, but have an informality rate of about 30% in Benito Juárez and Polanco. People with no insurance could work in some informal sector, but could also be students or retired, so I wonder how the age structure in the city changes their estimates. Regarding the employment data, DENUE only gives formal locations with size ranges (including one that says "above 250 employees"). How is the location of informal jobs determined? The claim that "Workers from high-informality neighborhoods in Mexico City and Rio de Janeiro are willing to travel farther to reach economically complex job centers, overcoming geographic and socioeconomic barriers."*

The reviewer questions the plausibility of some informality estimates, noting that certain remote locations appear with low informality and that neighborhoods such as Benito Juárez and the area near Polanco show informality rates around 30%, and asks how the location of informal jobs is determined.

We thank the reviewer for the careful inspection of our data. We address each observation in turn.

Remote low-informality zones. The zones that appear in remote areas with low informality correspond to census tracts with very low population density (e.g., mountainous or conservation areas in the south and west of Mexico City). Because very few economically active residents are recorded in these tracts, the informality estimate becomes unreliable. These zones were filtered from the main analysis: we retain only locations with incoming and outgoing commuting flows above a minimum threshold, which effectively removes these sparsely populated areas.

Informality estimation methodology. For Mexico City, our informality rate is computed from INEGI's 2020 Population and Housing Census microdata at the urban AGEB level. We use affiliation to social security services (access to medical insurance) as a proxy for formal employment, following the legal requirement for formal employers in Mexico to register workers in the social security system (IMSS). We compute the informality rate among the economically active population, adjusting for insured dependents (minors covered through a family member who are not themselves employed). This methodology is validated against Mexico's National Survey of Occupations and Employment (ENOE, 2022), which confirms that 99.95% of formally employed individuals have medical insurance coverage, compared to only 11.18% of informal workers. We have expanded this description in the Variable Construction section of the revised manuscript.

Employment data. DENUE reports the number of employees per establishment as categorical size ranges rather than exact counts. We draw a uniform random integer within each range to obtain a point estimate; for the open-ended upper category (251 employees or more) we set a ceiling of 500. The same procedure is applied to RAIS data for Rio de Janeiro. We have added this description to the Variable Construction section and acknowledge the resulting imprecision as a limitation.

Benito Juárez and Polanco. The informality rates in the range of 25–35% observed in these areas, while initially surprising, are plausible when the spatial composition of these neighborhoods is considered. Benito Juárez contains several historically lower-income colonias (Álamos, Portales, Narvarte Oriente) alongside its more affluent sections, and these areas host concentrations of street vendors, domestic workers, and small-scale service providers who operate informally. Similarly, the zones near Polanco that show elevated informality adjoin the colonias of Anáhuac and Pensil in the Miguel Hidalgo borough, which have long-standing patterns of informal economic activity. The spatial resolution of our analysis (AGEB level, $\sim$0.85 km^2) naturally aggregates these adjacent social realities within the same zone. The spatial autocorrelation analysis presented in the Supplementary Information (Moran's I for informality in Mexico City = 0.407, $p < 0.001$) confirms that these patterns are spatially structured rather than random, supporting the robustness of our informality estimates.

Minor Comments

There are some phrases that sound odd to me:

—Rio de Janeiro has the smallest number of links and nodes, while Mexico City has the largest, it is worth noting that the number of links corresponds to a complete graph with no self-loops, meaning that there is at least one commute connecting each pair of locations.

—Meaning that the higher the ECI of the work locations the more attractive to workers it becomes, this regardless of the region.

The model seems to persistently under-estimate the most common journeys (Figure 4). With a log scale, this is partly hidden, but without the log scale, this would be far more evident. This is more evident in the Fig. S14–17. I wonder how can this be fixed.

Equations need punctuation marks.

The reviewer flags two sentences that read oddly, notes that the model persistently underestimates the most common journeys (Figure 4), and requests punctuation for display equations.

Phrasing. We revised the two flagged sentences for clarity: the sentence on link/node counts now explicitly states that the OD matrix is a complete graph (no self-loops), and the sentence on the ECI coefficient has been restructured to improve readability.

Underestimation of common journeys. We agree that WorkReach (and gravity/radiation baselines alike) systematically underestimates the highest-flow OD pairs, a pattern visible on the log-scale scatter plots (Figures S15–S18) and in Figure 4. This is a known limitation of multinomial logit models applied to spatially concentrated flow distributions: a handful of dominant short-distance OD pairs carry disproportionate flow mass, and the softmax prediction distributes probability more evenly across destinations than the observed data. Because these high-flow pairs are typically short-distance intra-zone or adjacent-zone trips, the underestimation has limited impact on the model's ability to capture the distance–complexity–informality trade-offs that are the focus of this study. Developing a zero-inflated or mixture extension that better captures the heavy right tail of the flow distribution is an interesting direction for future work.

Equation punctuation. We have added commas or periods to all display equations throughout the manuscript so that they integrate grammatically with the surrounding text.

Reviewer 4

Comment 1: ECI Interpretation and Validation

> *I believe the paper suffers from a misinterpretation of economic complexity in the context of employment across industries and urban neighborhoods. In this setting, complexity captures the sophistication of the local employment structure, or perhaps the sophistication of the local job market. However, high ECI values in this context clearly do not imply higher "job quality" or more "complex job opportunities."*
>
> *This lack of a clear interpretation makes the reader suspect that the empirical exercise is motivated primarily by data availability rather than by a well-articulated conceptual argument supporting variable selection.*

The reviewer notes that economic complexity in the context of urban employment captures the sophistication of the local job market, but that high ECI does not necessarily imply higher "job quality" or more "complex job opportunities," and that the empirical exercise appears motivated by data availability rather than by a clear conceptual argument.

We thank the reviewer for this important point. We have revised the Introduction and Discussion to provide a clearer conceptual argument for why ECI^{emp} is a useful destination attribute. In the Introduction, we now ground the choice of ECI^{emp} over wages in agglomeration theory: zones where diverse and complementary industries co-locate generate productivity externalities, lower job-search costs by concentrating a wider range of employment opportunities, and facilitate human capital accumulation [4, 5]. ECI^{emp} captures these location-level advantages that current compensation does not internalize. In the Discussion (Limitations), we acknowledge that ECI^{emp} remains an aggregate index and that decomposing which agglomeration channels most strongly attract commuters is an important direction for future work. We have also adopted the notation $\text{ECI}^{\text{emp}}/\text{PCI}^{\text{emp}}$ throughout the manuscript to distinguish our sub-city measures from the country-level originals [6], and we confirm that the zone–sector binary matrices underlying the ECI^{emp} computation are nested in all four cities (SI Section S4.2), supporting the reliability of the complexity rankings.

Comment 2: Empirical Validation of ECI

> *From an empirical standpoint, this interpretation could be supported by robustness checks examining how income, pure job concentration, the share of higher-educated workers, or land-use patterns relate to higher ECI values. This would help validate the use of ECI (i.e., show that it captures something otherwise unobserved) and support a more accurate interpretation of the results.*

The reviewer suggests that the interpretation of ECI could be supported by robustness checks examining how income, job concentration, education, and land-use patterns relate to ECI values across zones.

We have conducted this analysis for the most direct economic proxy available in each city: zone-level mean wages for the Bay Area and Los Angeles (from the Replica synthetic employment dataset), flow-weighted mean household income for Rio de Janeiro (2020 Brazilian Census), and a flow-weighted composite wealth index for Mexico City (16 census indicators). The results appear in SI Section S4.5.

Zone-level correlations between ECI and these income or wealth proxies are positive and moderate ($r = 0.53$ Bay Area, $r = 0.61$ Los Angeles, $r = 0.59$ Mexico City, $r = 0.54$ Rio de Janeiro;

Figure S29), confirming that ECI captures information related to but not reducible to current economic status. Likelihood-ratio tests show that each variable contributes independently when added to the other ($p < 0.001$ in all four cities and both directions; Table S12).

A systematic analysis of education levels and land-use patterns across zones would require spatially harmonized data that are not uniformly available across our four study cities. We note this as a valuable direction for future work and thank the reviewer for the suggestion.

Comment 3: ECI–Informality Relationship

> *The relationship between ECI and commuting, as well as between ECI and informal employment, is not clearly articulated. My intuition is that it is not ECI per se that drives informal workers, but rather the demand for relatively less-qualified labor or for workers willing to accept lower pay or harsher working conditions (among several possible mechanisms).*
>
> *One possible explanation is that places with higher ECI offer a more diverse set of opportunities, creating a safer environment in which informal workers may have access to a larger pool of jobs, or in which switching jobs is easier due to localized information on job opportunities. Clarifying this relationship is, in my view, key to the paper. I can accept the argument that informality and ECI represent independent dimensions, but in that case this interpretation should be made explicit and supported as clearly as possible.*

The reviewer notes that the relationship between ECI and commuting, and between ECI and informal employment, is not clearly articulated. The reviewer suggests it may not be ECI per se that attracts informal workers, but rather demand for less-qualified labor, willingness to accept harsher conditions, or the job-market thickness of high-ECI areas. The reviewer can accept the argument that informality and ECI represent independent dimensions but asks for this interpretation to be made explicit and supported.

We thank the reviewer for this important observation. We have addressed it through both empirical evidence and mechanistic discussion.

ECI is a *destination* attribute that summarizes the productive sophistication of a zone's economy; residential informality is an *origin* attribute that characterizes labor market formalization at the worker's home location. We computed the cross-zone correlation between the two for all four cities: near-zero in the U.S. cities ($r = 0.030$ Bay Area, $r = 0.016$ Los Angeles; both $p > 0.4$) and moderate in the Latin American cities ($r = -0.401$ Mexico City, $r = -0.394$ Rio de Janeiro; both $p < 0.001$, $R^2 \leq 0.16$). The full analysis appears in a new SI section (Section S4.1, with zone-level scatter plots and correlation table).

Regarding the mechanisms behind the positive β_{inf} in Latin American cities: the three hypotheses the reviewer raises (demand for less-qualified labor, willingness to accept harsher conditions, job-market thickness) are not mutually exclusive and are all consistent with our specification. In the model, β_{ECI} captures aggregate destination attractiveness and β_{inf} captures origin-side differences in distance tolerance. Disentangling which mechanism dominates would require individual-level data on skills and job search histories; we note this as a direction for future work.

Specific changes:

- Results (Estimated Coefficients): added a sentence on the regional contrast in β_{inf} with reference to SI Section S4.1.
- Discussion: added a paragraph on the ECI–informality correlation and the spatial-mismatch mechanism in Latin American cities.

- SI Section S4.1 (new): "ECI and Informality as Complementary Dimensions" with scatter plots and correlation table.

Comment 4: Intuition for the High-ECI / High-Informality Interaction

> *Why does the combination of high ECI and high informality generate the highest utility for commuters? An illustrative example or stylized scenario would be very helpful to make this mechanism more intuitive.*

The reviewer asks why the combination of high ECI at the destination and high residential informality at the origin generates the highest utility for commuters, and requests an illustrative example or stylized scenario to make this mechanism more intuitive.

The utility function (Eq. 1) in the far-away regime ($w_{ij} \approx 1$) takes the form:

$$V_{ij} \approx \beta_{\text{dist}}\, d_{ij} + \beta_{\text{ECI}}\, ECI_j + \beta_{\text{inf}}\, Inf_i\,.$$

In this regime both β_{ECI} and β_{inf} are positive in the Latin American cities (Mexico City: 4.220 and 10.250; Rio de Janeiro: 6.603 and 4.267). High ECI at the destination therefore adds a positive term, and high informality at the origin adds a second, independent positive term. The two effects compound: a worker from a high-informality neighborhood gains utility both from the richness of the destination and from the structural feature of their origin that makes such long-distance trips worthwhile.

A concrete scenario makes this transparent. Consider a worker living in a peripheral, high-informality borough of Mexico City (e.g., eastern Iztapalapa), where the local labor market consists largely of street vendors, domestic workers, and small family workshops, all of which are informal, low-complexity, and without social security coverage. When this worker evaluates commuting to a distant high-ECI district (e.g., a professional or financial corridor), two forces act simultaneously. First, the destination's high ECI contributes a direct utility gain ($\beta_{\text{ECI}} \cdot ECI_j > 0$): high-ECI zones concentrate a broader and more diverse set of formally registered employment opportunities, reducing job-search friction for all workers. Second, the worker's high residential informality contributes an additional utility gain ($\beta_{\text{inf}} \cdot Inf_i > 0$): because local formal employment is scarce, the marginal benefit of accessing a distant high-ECI zone is larger for this worker than for one who already has convenient access to formal, complex jobs. These two terms add rather than cancel, making the high-ECI / high-informality pairing the utility-maximizing combination in the far-away regime.

The opposite pattern holds in the U.S. cities, where $\beta_{\text{inf}} < 0$ (Bay Area: -14.735; Los Angeles: -8.702). Here the term $\beta_{\text{inf}} \cdot Inf_i$ is negative, so high residential informality *reduces* the utility of distant high-ECI destinations. The highest utility for long commutes in these cities is therefore associated with *low*-informality origins commuting to high-ECI destinations, the pattern of formally employed suburban workers willing to travel to dense professional centers, which is consistent with the longest-commute group identified in Figure 2e. This regional contrast follows directly from the sign reversal of β_{inf} and requires no additional assumptions beyond the estimated model.

Comment 5: Engagement with the Intra-City Economic Complexity Literature

> *The paper does a very poor job of engaging with the existing literature that applies economic complexity measures at the intra-city scale. Some of these studies even use mobility data to explain the role of ECI (e.g., Juhász et al., 2023). Below are a few relevant references that should be discussed:*

Juhász et al. (2023) Amenity complexity and urban locations of socio-economic mixing. EPJ Data Science

Kim et al. (2024) Redefining urban centrality: Integrating economic complexity indices to central place theory. arXiv:2407.19762

Rossi Mori et al. (2025) Time-space dynamics of income segregation in the city of Milan. PNAS Nexus

Chen et al. (2026) Neighborhood clusters and citywide technological diversification. Research Policy

The reviewer notes that the paper does not sufficiently engage with the growing literature applying economic complexity measures at the intra-city scale, and provides four specific references: Juhász et al. (2023), Kim et al. (2024), Rossi Mori et al. (2025), and Chen et al. (2026).

We are genuinely grateful for these references, which we had not fully incorporated into our discussion. We have now revised the Introduction to engage more explicitly with this literature, adding citations alongside our existing reference to Magalhães et al. (2023) at the point where we introduce sub-city economic complexity. We note, with appreciation for the reviewer's thoroughness, that two of the four suggested references, Rossi Mori et al. (2025) and Chen et al. (2026), appeared after our manuscript was originally submitted, and were therefore not available to us at the time of writing. We are delighted to include them in the revised version.

We briefly note how each work relates to ours:

- Juhász et al. (2023) extend the complexity framework to amenity portfolios in Budapest, and crucially, use individual-level mobility traces to show that locations with high amenity complexity attract more socioeconomically diverse visitors. This is methodologically close to our own approach: we similarly use mobility data and a complexity index to study destination selection, though we focus on workplace rather than amenity choice and on the modulating role of residential informality.
- Kim et al. (2024) demonstrate, for Seoul, that ECI and PCI at the sub-city level capture urban centrality in the sense of Central Place Theory, showing that high-complexity zones act as magnets within the urban system. Their results are consistent with our finding of a consistent positive β_{ECI} across all four cities.
- Rossi Mori et al. (2025) combine mobile phone data with income-based zone classification to study the time-space dynamics of socioeconomic mixing in Milan. Their focus on how mobility redistributes social contact across the urban fabric complements our framework, which examines how complexity and informality shape the directionality of commuting flows.
- Chen et al. (2026) analyze neighborhood-level technology clusters across Chinese cities and find that local clusters promote citywide diversification toward related and more complex technologies. While their unit of analysis is innovation rather than labor mobility, the finding that neighborhood-scale complexity has city-scale consequences is broadly consistent with the sub-city ECI gradients we document.

Comment 6: Cross-City Differences and Socioeconomic Comparison

Figure 2c suggests that intra-city economic complexity measures are more strongly correlated in U.S. cities than in Latin American megacities. I suspect this reflects differences in development, wealth, urban form, regulation, institutions, and other contextual factors.

It would be useful to explicitly compare the contexts of these cities and provide basic statistics on socio-economic characteristics such as income, wealth, dominant commuting modes, etc., rather than leaving this as speculation.

The reviewer observes that Figure 2c suggests intra-city economic complexity measures are more strongly correlated in U.S. cities than in Latin American megacities, and asks for an explicit comparison of socioeconomic contexts across the four cities.

We agree that the regional pattern in Figure 2c reflects structural differences in development, urban form, and institutional context. The Bay Area and Los Angeles share a common regulatory environment, similar sector definitions (NAICS), and comparable levels of economic development, which produces highly correlated co-location patterns ($r = 0.85$). Under the updated ECI^{emp} computation (see the *Updated* ECI^{emp} *computation* summary at the start of this document), Mexico City and Rio de Janeiro now show equally strong within-region similarity ($r = 0.86$), despite differences in industrial composition, labor regulation, and data sources (DENUE vs. RAIS). The cross-regional correlations are lower ($r = 0.68$–0.71), consistent with the structural differences the reviewer identifies.

We have added contextual discussion in the manuscript noting these differences. A full cross-city comparison of income distributions, dominant commuting modes, housing markets, and regulatory frameworks would be a valuable complement to our analysis but falls outside the scope of the current study, which focuses on whether the ECI–informality–distance trade-off structure is consistent across cities with very different socioeconomic profiles. The fact that WorkReach's qualitative findings hold across these diverse contexts, despite quantitative differences in parameter magnitudes, is itself informative about the generality of the underlying mechanism.

Comment 7: Nested Structure of the Binary RCA Matrices

Economic complexity measures are known to perform reliably when the underlying binary matrix exhibits a nested structure (see Bustos et al. 2012 PLOS One). Showing the four underlying matrices would substantially strengthen the credibility of the analysis.

The reviewer notes that economic complexity measures perform reliably when the underlying binary matrix is nested (i.e., the sector portfolio of less-diversified zones is a subset of the portfolio of more-diversified zones) and requests that we show this property holds for our four zone–sector matrices.

We have conducted this analysis and report the results in SI Section S4.2. For each city we plot the binary RCA matrix sorted by zone diversity and sector ubiquity (Figure S26) and compute NODF scores against a random null distribution (Table S7). All four matrices exhibit staircase patterns and NODF scores well above their null expectations ($p < 0.001$ in all cities), confirming significant nestedness and, with it, the reliability of the ECI rankings [7].

- Added SI Section S4.2 ("Nested Structure of Zone–Sector Binary Matrices") with sorted zone–sector binary heatmaps (Figure S26) and a NODF results table (Table S7).

Comment 8: Product-Space Network Visualization

The network of Figure 2b appears almost fully connected from a visual perspective, yet it is interpreted as showing that "highly complex sectors tend to cluster closely in the network, while less complex sectors do the same." Applying an MST-based approach or a disparity filter to retain only statistically significant ties would help the visualization convey this message more clearly.

The reviewer observes that the product-space network in Figure 2b appears almost fully connected, and suggests applying an MST-based approach or a disparity filter to retain only statistically significant ties.

We agree that the original visualization was difficult to read because many weak edges obscured the clustering structure. We considered applying a disparity filter [8] to extract the network backbone, but found it unsuitable for our setting: with only 17 NAICS sectors the network has at most $\binom{17}{2} = 136$ possible edges, leaving the filter's null model underpowered. At $\alpha = 0.05$ the filter retains 0–1 edges per city, and even at $\alpha = 0.40$ it retains fewer edges than a fixed proximity threshold.

We therefore adopted the approach of Hidalgo et al. [9], who constructed the original product-space visualization as a maximum spanning tree (MST) supplemented by all edges above a proximity threshold. The MST guarantees a single connected component, while the threshold controls network density. We use $\phi^* = 0.30$ for the U.S. cities and $\phi^* = 0.20$ for the Latin American cities. The lower cutoff for Mexico City and Rio de Janeiro reflects an empirical asymmetry in co-location density: the U.S. cities exhibit denser proximity matrices where many sectors co-occur at comparable rates (62–73 edges, mean degree 7.3–8.6), while the Latin American cities display sparser networks with fewer strong ties (57–60 edges, mean degree 6.7–7.1), indicating sharper sectoral specialization. This structural difference is consistent with the higher nestedness scores observed for the U.S. cities (Table S7). Product-space network plots for all four cities are provided in SI Section S4.3.

- Updated Figure 2b to use the MST-based filtering approach.
- Added SI Section S4.3 with a four-city product-space network comparison (Figure S27) and a summary table of network statistics across filtering strategies (Table S8).

Comment 9: Colorbar Placement in Figure 2

> *In Figure 2b, the colorbar label is very close to panel (c), making it easy to miss that panel (c) is not related to PCI. This should be corrected.*

The reviewer notes that the PCI colorbar label in Figure 2b is positioned too close to panel (c), making it easy to misread panel (c) as related to PCI.

We have adjusted the layout of Figure 2 so that the PCI colorbar is clearly separated from panel (c). We thank the reviewer for flagging this issue.

Comment 10: Highlighting the ECI–Informality Correlation Explicitly

> *From the text, we learn that informality and ECI are weakly correlated. This is an important result for the paper, as it suggests that the two variables capture largely independent properties of urban locations. Please highlight this explicitly using a heatmap or a standard correlation table.*

The reviewer asks for the weak correlation between ECI and informality to be highlighted explicitly using a heatmap or correlation table, noting that this is an important result supporting the independence of the two variables.

We are glad to report that this result is now present in the manuscript in two places. In the Introduction, we state explicitly that "zone-level correlations between ECI and informality rates are near-zero in the U.S. cities ($r \leq 0.03$, $p > 0.4$) and moderate in the Latin American cities ($r \approx -0.40$), confirming that they capture distinct aspects of the urban labor market and are not interchangeable proxies," with a reference to SI Section S4.1. The same finding is reported in the Results. SI Section S4.1 contains zone-level ECI–informality scatter plots and a correlation table

for all four cities (Table S6), which directly fulfill the reviewer's request for a visual and tabular summary.

Comment 11: ECI/PCI Naming Convention

> *It is problematic that variable names such as ECI and PCI are used in contexts that differ substantially from their original definitions. I strongly suggest using alternative terminology that better reflects this novel use case, such as "neighborhood complexity" and "industry complexity," analogous to ECI and PCI. This approach is consistent with prior work, including Juhász et al. (2023, EPJ Data Science).*

The reviewer suggests replacing the terms ECI and PCI with "neighborhood complexity" and "industry complexity" to better reflect the sub-city context, following the precedent of Juhász et al. (2023).

We appreciate this suggestion and considered it carefully. We opted instead to adopt the notation ECI^{emp} and PCI^{emp} throughout the manuscript, using calligraphic script to distinguish our sub-city measures from the country-level originals (ECI and PCI). We added a sentence in the Economic Complexity Framework section explaining this convention: "Because our ECI and PCI are derived from employment data at the sub-city level rather than from trade data at the country level, we denote them ECI^{emp} and PCI^{emp} throughout to distinguish them from their original formulations." We chose this approach over "neighborhood complexity" and "industry complexity" for two reasons: (i) the underlying mathematical construction (method of reflections applied to a bipartite zone–sector network) is identical to the original ECI/PCI, so a new name might obscure rather than clarify the methodological lineage; and (ii) the calligraphic notation immediately signals to readers familiar with the complexity literature that the same index is being applied at a different spatial scale, while remaining self-explanatory to readers encountering the concept for the first time.

Comment 12: ECI versus Diversity and Ubiquity

> *ECI reflects not only industrial diversity at urban locations but also ubiquity. Ubiquity likely plays a major role in commuting: retail jobs, for example, require less specialized skills and are spatially widespread to ensure proximity to customers, which likely reduces commuting distances. At the same time, ubiquity may be closely linked to informality. Please provide robustness checks demonstrating that the ECI measure captures something distinct from pure diversity and ubiquity.*

The reviewer notes that ECI combines industrial diversity and sector ubiquity, and raises the concern that high ubiquity may be linked to informality (e.g., retail jobs are widespread and disproportionately informal). The reviewer asks whether ECI captures something distinct from its constituent components and requests robustness checks.

This is an insightful observation. ECI is constructed from the same binary matrix that underlies diversity and ubiquity: the method of reflections iterates between the two, assigning high complexity to zones that are diversified specifically into rare sectors. The reviewer is correct that neither component alone carries the joint signal, and that high-ubiquity sectors such as retail tend to be less complex and are over-represented in informal employment. We have conducted the requested robustness analysis; results appear in SI Section S4.4.

We first examine how ECI relates to its two raw ingredients (Figure S28). ECI is weakly to moderately correlated with diversity ($r = 0.18$–0.49) and more strongly, but heterogeneously, correlated with mean ubiquity ($r = -0.29$ in Rio de Janeiro to -0.95 in the Bay Area). In cities where the ubiquity correlation is high, ECI is largely recoverable from that single component; in others, the iterative reflections add substantial information. We then compare four WorkReach specifications, using ECI, diversity only, mean ubiquity only, and an orthogonal residual $\mathrm{ECI}^{\mathrm{employment},\perp}$ that removes all variation explained by diversity and ubiquity (Table S10).

Ubiquity alone yields higher CPC and r than ECI in all four cities, and diversity outperforms ECI in Mexico City and Rio de Janeiro. This is not surprising: mean ubiquity has a direct mechanical link to commuting, since zones dominated by ubiquitous sectors (e.g., retail) offer locally available employment that reduces the need for long-distance travel, making ubiquity a strong standalone predictor of flow concentration. Critically, even the orthogonal residual $\mathrm{ECI}^{\mathrm{employment},\perp}$, which by construction contains no information from diversity or mean ubiquity, substantially outperforms the randomized-ECI null from the permutation analysis, confirming that ECI encodes information about destination attractiveness beyond what its two raw ingredients provide. The advantage of ECI over its individual components is therefore not strictly predictive but structural: ECI is embedded in the broader economic complexity framework, which provides the feasibility score needed to identify which new sectors a zone could realistically develop and to simulate the resulting change in commuting accessibility (SI Section S8). Neither diversity nor ubiquity alone supports this type of counterfactual analysis.

On the retail–informality concern specifically: retail carries low product complexity index in all four cities and yields low zone ECI when it is the dominant sector. In the WorkReach utility, retail-dominated destinations therefore receive low utility in the long-distance regime, precisely because the transition weight w_{ij} makes the ECI term influential at larger distances. A diversity-only measure would incorrectly assign high utility to zones that host many sectors if those sectors happen to be ubiquitous. ECI's discount of ubiquitous sectors is the property that makes it especially well suited as a covariate in our model.

- Added SI Section S4.4 ("ECI versus Diversity and Ubiquity") with an orthogonal decomposition, scatter plots of ECI against its constituent components (Figure S28), and a model comparison table across four specifications (Table S10).

Comment 13: β_{inf} Sign versus Figure 2e

> *On page 12, the paper states: "The coefficient (β_{inf}) is negative in the Bay Area and Los Angeles, which indicates that high-informality areas in these cities are less prone to travel long distances for high-ECI work alternatives." This interpretation is difficult to reconcile with Figure 2e, where the high-informality–high-complexity group in these cities exhibits the highest median commuting distance. Please clarify how these results should be interpreted jointly.*

The reviewer notes that $\beta_{\text{inf}} < 0$ in the Bay Area and Los Angeles implies that high-informality areas are less prone to long-distance high-ECI commutes, yet Figure 2e shows the high-informality/high-ECI group exhibiting the highest median commuting distance in these cities. The reviewer asks how these results should be interpreted jointly.

We thank the reviewer for identifying this apparent tension. The reconciliation lies in the distinction between the model's conditional prediction and the unconditional descriptive statistic shown in Figure 2e.

The model coefficient β_{inf} enters the utility through the interaction $w_{ij}(\tau, k) \cdot \beta_{\text{inf}} \cdot Inf_i$, where

$w_{ij} \approx 1$ only for destinations beyond the distance threshold τ. When $\beta_{\text{inf}} < 0$, the model predicts that, *all else equal*, a worker from a high-informality origin assigns lower utility to distant high-ECI destinations than an otherwise identical worker from a low-informality origin. This is a conditional statement: it holds when controlling for the spatial distribution of origins and destinations.

Figure 2e, by contrast, reports the unconditional median commuting distance within each informality–ECI group separately. It describes each group's own distribution of commutes; it does not benchmark one group against another at a given (distance, ECI) combination. The negative β_{inf} in the Bay Area and Los Angeles is precisely such a between-group statement: holding distance and destination ECI fixed, workers from high-informality origins assign lower utility to distant high-ECI destinations than workers from low-informality origins do.

We further note that Figure 2e shows the longest median commutes in the Bay Area and Los Angeles for the *low*-informality / high-ECI group, not the high-informality / high-ECI group. This descriptive pattern is itself consistent with $\beta_{\text{inf}} < 0$ in these cities and aligns with the between-group interpretation above.

Comment 14: Figure 5 Group Definitions

> *Based on Figure 5 alone, it is unclear what the "low" and "high" groups refer to. Please revise the figure to make it self-contained and interpretable without relying on the main text.*

The reviewer notes that Figure 5 does not define the "low" and "high" groups, making the figure difficult to interpret without the main text.

We have revised the caption of Figure 5 to state explicitly that origins are classified as "low" or "high" informality based on whether their informality rate falls below or above the city-specific median. This makes the figure self-contained and interpretable without reference to the text.

References

1. U.S. Bureau of Labor Statistics. *Job Openings and Labor Turnover Survey (JOLTS) Highlights* Federal Reserve Bank of St. Louis (FRED). Monthly quits rate approximately 2.2%. 2024. https://fred.stlouisfed.org/series/JTSQUR.
2. Alaimo, V., Bosch, M., Kaplan, D. S., Pagés, C. & Ripani, L. *Jobs for Growth* (Inter-American Development Bank, Washington, DC, 2017).
3. Sobrino, J. Housing Prices and Submarkets in Mexico City: A Hedonic Assessment. *Estudios Económicos* **29,** 57–84 (2014).
4. Moretti, E. in *Handbook of Labor Economics* (eds Ashenfelter, O. & Card, D.) 1237–1313 (Elsevier, 2011).
5. Glaeser, E. L. & Gottlieb, J. D. The Wealth of Cities: Agglomeration Economies and Spatial Equilibrium in the United States. *Journal of Economic Literature* **47,** 983–1028 (2009).
6. Hidalgo, C. A. & Hausmann, R. The building blocks of economic complexity. *Proceedings of the National Academy of Sciences* **106,** 10570–10575. ISSN: 0027-8424, 1091-6490. https://pnas.org/doi/full/10.1073/pnas.0900943106 (June 2009).
7. Bustos, S., Gomez, C., Hausmann, R. & Hidalgo, C. A. The Dynamics of Nestedness Predicts the Evolution of Industrial Ecosystems. *PLOS ONE* **7,** e49393 (2012).

8. Serrano, M. Á., Boguñá, M. & Vespignani, A. Extracting the Multiscale Backbone of Complex Weighted Networks. *Proceedings of the National Academy of Sciences* **106,** 6483–6488 (2009).

9. Hidalgo, C. A., Klinger, B., Barabási, A.-L. & Hausmann, R. The Product Space Conditions the Development of Nations. *Science* **317.** Publisher: American Association for the Advancement of Science, 482–487. `https://www.science.org/doi/10.1126/science.1144581` (July 2007).

Point-by-Point Response to Reviewer Comments

Second Round of Review

The WorkReach model for urban work location choices through economic complexity and informality

Ollin D. Langle-Chimal, Steffen Knoblauch, Marta C. González

June 30, 2026

Point-by-Point Responses

We thank the reviewers for their careful re-evaluation of the revised manuscript and welcome their positive assessment. Each comment is reproduced verbatim in a quoted block, followed by an italicized summary of how we read it and our response. All new or modified text introduced in this revision is highlighted in blue; text added in the previous round appears in black. We have additionally reformatted the manuscript to the Nature Communications style, including an unpunctuated title, a 150-word abstract, unnumbered single-level headings, separate Data Availability, Code Availability, Author Contributions, and Competing Interests sections, the consolidation of data and variable-construction details into the Methods which were previously part of the Introduction, and display items cited in order of appearance.

Reviewer 1

Comment 1: Framing of Multiple Mechanisms

We thank the authors for the thorough and constructive response. Comments other than 1 and 3 have been fully addressed. With respect to comments 1 and 3, we consider the new analyses on wages (S4.5), skill relatedness (S5), and the informality–distance interaction to add substantially to the paper. They are interesting in their own right and show that wages, skill relatedness, and ECI each carry independent predictive information about commuting flows, with their relative contributions varying systematically across cities.

The study is interesting and speaks to several distinct phenomena at once. Our remaining concern is that the current framing pushes a single ECI/pull narrative, when there is no evidence in the paper that this is the dominant mechanism. In fact, we think there is something more interesting in the results: the joint evidence for multiple co-existing forces shaping commuting behaviour.

Specifically, the new analyses show:

A wage / income-wealth proxy carries comparable predictive information to ECI, outperforming it in two of four cities (Table S11). We recognise the wage–distance trade-off is well established in labor economics and may not feel novel to report; in this paper's context, however, it is informative as a benchmark for how much of the ECI effect overlaps with simple compensation, even if a direct apples-to-apples comparison of behavioural magnitudes is not straightforward given the different scales of the variables.

Skill relatedness is negative and highly significant in all four cities, and the ECI+SR specification outperforms WorkReach everywhere (Tables S13–S15). This is less commonly reported in the commuting literature and we think it is a new finding worth foregrounding.

In the joint ECI+SR specification (Table S14), β_{ECI} attenuates substantially in the Latin American cities (Mexico City: 4.22 to 1.05; Rio: 6.60 to 0.94), and β_{inf} flips sign in both (Mexico City: $+10.25$ to -4.62; Rio: $+4.27$ to -1.13). The empirical anchor of the discussion section's policy framing, namely high-informality workers commuting to high-ECI zones, does not survive controlling for skill relatedness.

Together, these results are consistent with at least three distinct mechanisms that the cross-sectional data cannot fully disentangle: pull from agglomeration and complexity, pull from compensation, and push from local skill mismatch. The β_{inf} sign flip in particular is consistent with workers in Latin American peripheries commuting out because local options are limited, as much as being drawn in by central-zone complexity.

We note that the authors' own MSR framework (Section 2.3.2) is well suited to expressing these trade-offs in comparable behavioural units, such as kilometres per unit of ECI, per unit of SR, or per unit of wage. This would let readers see directly how the forces compare on a common footing, which is otherwise difficult given that the raw coefficients sit on different scales.

We therefore believe the current narrative, focused on ECI alone, does not report the full story the analyses support. We suggest the following:

(1) Provide evidence that the ECI/pull interpretation is the dominant mechanism. This would justify retaining ECI as the central narrative. Approaches that would help include:

a local-job-availability control at the origin (push effects should attenuate ECI and SR coefficients if mismatch is the driver); stratification by local labour-market tightness (pull predicts uniform ECI effects, push predicts stronger effects where local options are scarce); sector-specific submodels (pull predicts stronger ECI effects in tradable, high-skill sectors); or decomposing ECI into specific agglomeration channels (own-sector density, sector diversity). We acknowledge this may be out of scope, and so as an alternative:

(2) Discuss all three mechanisms with equal standing in the abstract, introduction, and discussion. Report SR alongside ECI as a co-equal finding, present MSR-based comparisons across ECI, SR, and wages so readers can see how the forces compare in behavioural terms, and revise language such as workers "valuing" complexity, which presumes a pull interpretation. The β_{inf} *sign change in the joint specification deserves explicit discussion given how central the positive* β_{inf} *finding is to the current policy framing.*

We think the paper is more credible, not less, for acknowledging the richer picture the analyses reveal.

We thank the reviewer for this careful and constructive reading, and we agree with its central observation that the analyses point to several co-existing forces that the cross-sectional data cannot fully separate, and that no single force can be claimed to dominate. We did not set out to present economic complexity as the dominant driver of commuting, and we have revised the framing accordingly, making the broader set of co-existing forces explicit and pointing readers to the supplementary analyses of the alternative variables. This largely follows the reviewer's second suggestion. We depart from it in one respect, explained below: rather than giving several covariates equal standing in the main text, we keep a single interpretable opportunity measure there and develop the alternatives fully in the Supplementary Information.

Contribution. The contribution is not only economic complexity as a mechanism, but the modeling framework itself, which yields two interpretable products that existing flow models do not. The first is a parsimonious model grounded in discrete choice in which a transition weight separates convenience-driven from opportunity-driven travel and each of its five parameters maps to a single interpretable quantity, so that the trade-offs reflected in observed commuting can be read as marginal substitution rates and elasticities. The second is a measure of accessibility that captures the perceived benefit of the opportunities a worker can reach rather than their mere proximity. We chose economic complexity as the opportunity measure not because it dominates, but because, among the destination attributes we examined, it is the one whose value does not depend on the worker's origin and that is actionable as a policy lever.

On compensation. We do not have a consistent wage measure across the four cities. For the U.S. cities we use a synthetic income estimate, and for Mexico City and Rio de Janeiro, where work-location wages are unavailable, we build income and wealth proxies from census data and assign them to workplaces through the commuting flows, which introduces a circularity, since the same flows then enter both the proxy and the dependent variable. We therefore cannot treat compensation as a clean, co-equal covariate, and we report the comparison transparently in the Supplementary Information while noting its limits in the main text.

On sectoral relatedness. This analysis takes up the reviewer's first-round suggestion to test whether the relatedness between origin and destination industries, computed from the product-space proximity matrix, predicts commuting flows. We originally named this measure "skill relatedness." Reviewer 5 subsequently noted that this term already denotes an established, labor-mobility-based metric (Neffke and Henning [1]) distinct from our co-occurrence, product-space construction.

After careful consideration we agreed, and to avoid confusion we renamed the measure "sectoral relatedness" throughout the manuscript and Supplementary Information; the measure and the way it is computed are unchanged, and only the name has changed. This is the analysis we find most interesting, and we present it in full. As the reviewer notes, the sectoral-relatedness-only and combined specifications outperform WorkReach on prediction in all four cities, and we say so plainly (Supplementary Note 5). We keep it out of the main specification for a reason of interpretability rather than of performance. Sectoral relatedness is not a property of a location but of the home–work pair, so the same residential informality level can correspond to very different relatedness depending on the destination, and very different informality levels can yield similar relatedness to a given destination. Folding it into the main model would blur how a worker's residential informality relates to the opportunity at a destination, the very relationship the model is built to make legible, and would cost the parsimony that lets each parameter carry a single interpretable meaning. We want to stress that this analysis is not a concession but a demonstration of the framework's value. It shows that the WorkReach form readily accommodates new variables and surfaces the interactions between them, here a relational, spatial-mismatch signal that an intrinsic measure such as economic complexity cannot express. This is precisely the kind of probing the model is designed to enable, which is why we develop the sectoral-relatedness analysis as a full companion study in the Supplementary Information.

On the informality coefficient. As the reviewer notes, the informality coefficient changes sign in Mexico City and Rio de Janeiro once sectoral relatedness is added. We now address this explicitly in the Discussion, where we test how robust the positive-coefficient reading is and show that, once sectoral relatedness is included, the informality coefficient turns negative in every city, so the reading of high-informality workers commuting toward complex central zones holds only in the parsimonious model. We present the informality signal not as a single push or pull but as entangled with sectoral structure, consistent with the interacting-forces picture. We have likewise revised language that presumed a pull reading, replacing formulations such as workers "valuing" complexity with descriptions phrased in terms of the estimated trade-offs.

Comparing the forces in common units. The reviewer suggests using our marginal-substitution-rate framework to compare these forces in common units. We have generalized the analytical derivation of the marginal substitution rate to specifications with any number of transition-weight-modulated attributes, and report the full set of pairwise rates, with 95% confidence intervals, for the sectoral-relatedness-only and combined specifications (Supplementary Tables 16 and 17). The income and wealth proxies sit on city-specific scales and are flow-weighted in the Latin American cities, which prevents a direct comparison with the substitution rates; we therefore keep their comparison with economic complexity in Supplementary Note 4.5 rather than alongside these rates.

In summary, this comment led us to the following changes to the manuscript and Supplementary Information:

- Made the broader set of co-existing forces explicit in the Results and Discussion and pointed to the supplementary analyses of the alternative variables, framing the contribution around the interpretable framework and its two products, a parsimonious model and a perceived-benefit accessibility metric, with economic complexity as the chosen, policy-actionable opportunity measure.
- Stated explicitly that a consistent wage measure is unavailable across the four cities, and reported the income/wealth proxy comparison, with its circularity caveat, in Supplementary Note 4.5.
- Explained in Supplementary Note 5 why sectoral relatedness, despite outperforming WorkReach on prediction, is retained as a companion analysis rather than added to the main model: as a property of the home–work pair it complicates the demographic–opportunity interpretation

and erodes the model's parsimony.

- Added a passage to the Discussion showing that the informality coefficient changes sign once sectoral relatedness is included, so the positive-coefficient reading holds only in the parsimonious model.
- Revised pull-presuming language (for example, workers "valuing" complexity) into trade-off-based descriptions in the main text.
- Generalized the analytical MSR derivation to any number of attributes and added Supplementary Tables 16 and 17 (95% confidence intervals) to Supplementary Note 5, enabling any pair of forces to be compared in common units.

Reviewer 2

> *The authors have carefully analysed the comments I provided in an earlier revision. There are still a few weaknesses in the study, but, given the authors' explanation, I think they are part of the data challenges and cannot be fixed. For example, the data for Mexico (DENUE) only provides a wide interval for the number of employees in large companies.*
>
> *I think the figures are a great part of the manuscript and convey information very succinctly.*

We thank the reviewer for their positive assessment of the revised manuscript and of the figures, and for recognizing that certain remaining constraints (such as DENUE reporting employee counts as size ranges for all establishments, most coarsely for the largest ones) are inherent to the available data sources rather than shortcomings of the analysis. We address the two specific remaining points below.

Comment 1: Informality in Mexico City versus its Metropolitan Area

> *Given the topic, I would like to see a comparison of the informality rate between Mexico City and its metropolitan area. I am still unconvinced that considering only a political division is enough for such an analysis, and unfortunately, I think that taking two cities based on their political division does not guarantee comparability. For example, taking the central part of Mexico City (named CDMX) is not the same as taking Manhattan, or the City of London, or Paris proper. If the informality is similar both within and beyond the political definition of Mexico City, given its metropolitan area, this would not be problematic. Maybe a histogram (as figure 1, bottom) is sufficient.*

We have added this comparison to the Supplementary Information (Supplementary Note 2). Using the same census-based informality proxy used for the model, we computed the informality rate for every populated H3 zone across the full Zona Metropolitana del Valle de México (ZMVM, $n = 2{,}610$ zones) and compared its distribution against that within Mexico City proper (CDMX, $n = 908$ zones), which the metropolitan area extends by adding the conurbated municipalities of the State of México and Hidalgo.

The two distributions cover essentially the same range, each running from near-zero informality to highly informal zones (from near 0 to 0.90 in CDMX proper, and from near 0 to 0.96 across the ZMVM), and they overlap substantially. They differ mainly in where their mass falls, with the metropolitan distribution sitting modestly higher (medians 0.414 for CDMX versus 0.486 for the ZMVM; two-sample Kolmogorov–Smirnov statistic $D = 0.282$), reflecting the somewhat more informal conurbated periphery. The political boundary of Mexico City proper therefore captures almost the same internal range of informality as the wider metropolitan area, from near-formal central zones to high-informality peripheral ones, which is the within-city variation the model draws on.

Concretely, we made the following addition:

- Added a new subsection and figure to Supplementary Note 2 comparing the informality distribution of CDMX proper against the full ZMVM (Supplementary Fig. 14), with a metropolitan-wide choropleth of the informality rate alongside the overlaid distributions of the two areas and their summary statistics.

Comment 2: Equation Punctuation

> *Please make sure that equations are expressions that can be read. They need punctuation marks (missing in equations 4, 5, 12, which need a comma after).*

We have added the missing terminal punctuation to the three equations the reviewer identified, the two accessibility measures and the extended radiation model. As part of reformatting the manuscript to the Nature Communications guidelines, the display equations were renumbered, so these now appear as Equations (4), (5), and (14); the radiation equation previously numbered 12 is now Equation (14), while the two accessibility equations remain (4) and (5). We further verified that all remaining display equations end with the appropriate comma or period so that each integrates grammatically with the surrounding text.

Reviewer 3

> *I co-reviewed this manuscript with one of the reviewers who provided the listed reports. This is part of the Nature Communications initiative to facilitate training in peer review and to provide appropriate recognition for Early Career Researchers who co-review manuscripts.*

Reviewer 3 co-reviewed the manuscript as part of the Nature Communications initiative to provide training and recognition for early-career researchers, and did not raise additional comments. We thank them for their contribution to the review process.

Reviewer 5

Comment 1: Naming of the Sectoral Relatedness Measure

> *A new skill-relatedness approach is added, which I found to be a very nice and valuable robustness check. However, I would suggest renaming it. The original skill-relatedness literature (see Neffke and Henning, 2013) connects industries through labor mobility and provides an alternative relatedness metric to co-occurrence-based measures such as the product space. More labor flow than expected signals the reusability of skills between industries, thereby making them "skill-related". Please consider choosing a different name to avoid confusion with this established literature. I think the current solution is, in essence, closer to the "complementarity" measure suggested by Balland and Boschma (2021).*
>
> *Subject to this small revision, I believe the manuscript is suitable for publication.*

We thank the reviewer and have renamed the measure. As the reviewer notes, our score is a co-occurrence (product-space) measure rather than the labor-mobility-based skill relatedness of Neffke and Henning [1]; we have accordingly renamed it "sectoral relatedness" throughout the manuscript and the Supplementary Information, grounding it explicitly in the product-space proximity of Hidalgo et al. [2] and distinguishing it from the skill-relatedness literature.

The reviewer also notes that our construction is close to the complementarity measure of Balland and Boschma [3]. Since our score quantifies the relatedness, or similarity, between two zones' sector portfolios, we keep "sectoral relatedness" as its most precise label, while recognizing the connection the reviewer draws.

References

1. Neffke, F. & Henning, M. Skill relatedness and firm diversification. *Strategic Management Journal* **34,** 297–316 (2013).
2. Hidalgo, C. A., Klinger, B., Barabási, A.-L. & Hausmann, R. The Product Space Conditions the Development of Nations. *Science* **317.** Publisher: American Association for the Advancement of Science, 482–487. `https://www.science.org/doi/10.1126/science.1144581` (July 2007).
3. Balland, P.-A. & Boschma, R. Complementary interregional linkages and Smart Specialisation: an empirical study on European regions. *Regional Studies* **55,** 1059–1070 (2021).

Point-by-Point Response to Reviewer Comments

Third Round of Revision

The WorkReach model for urban work location choices through economic complexity and informality

Ollin D. Langle-Chimal, Steffen Knoblauch, Marta C. González

September 3, 2026

Point-by-Point Responses

We thank the editor and the reviewers for their continued attention to this manuscript. Each comment is reproduced verbatim in a quoted block, followed by our response. All text added or modified in this revision is highlighted in blue in the manuscript; text added in previous rounds now appears in black.

Editorial Request

> *Please make amendments to the Introduction and Discussion section following Reviewer #1 request. It is not mandatory to report the co-existing mechanisms with equal standing in the abstract, introduction, and discussion, but this should not be only mentioned in the SI file.*

The co-existing mechanisms are now reported in the Introduction, the Results, and the Discussion rather than only in the Supplementary Information. Following the editor's guidance that equal standing is not mandatory, we report them plainly without restructuring the manuscript around them, and we retain economic complexity as the opportunity measure of the main specification. Our response to Reviewer 1 describes the changes in detail.

Reviewer 1

Comment 1

> *The authors have not addressed my comment but circumvented it. I asked that the co-existing mechanisms be reported with equal standing in the abstract, introduction, and discussion; the response instead defends the model specification, which I never asked them to change. My concern is that the paper pursues a narrative rather than reporting all of its evidence: sectoral relatedness outperforms WorkReach in all four cities, and the informality coefficient flips sign once it is included, yet neither result reaches the abstract or introduction. These belong in the main text, and no change to the model is needed to put them there.*

We thank the reviewer for this comment, which has made the paper more complete. These results are now in the main text, with their values.

Results. We now report that substituting relatedness for $\mathrm{ECI}^{\mathrm{emp}}$ in the utility yields a Common Part of Commuters of 0.391 (Bay Area), 0.425 (Los Angeles), 0.495 (Mexico City), and 0.652 (Rio de Janeiro), against 0.389, 0.413, 0.486, and 0.614 for WorkReach, while a model carrying both reaches 0.399, 0.428, 0.497, and 0.655 (Supplementary Table 13). We also report that adding either covariate to a model that already contains the other still improves fit significantly (Supplementary Table 15), so the two co-exist rather than substitute for one another, and that the informality coefficient becomes negative in all four cities, changing sign in Mexico City (10.250 to -4.620) and Rio de Janeiro (4.267 to -1.133) (Supplementary Table 14).

Introduction. The closing paragraph now states that work location choices are shaped by co-existing, complementary influences, names both economic complexity and sectoral relatedness, and reports that relatedness reproduces the flows slightly more closely than economic complexity.

Discussion. The paragraph on the informality coefficient now reports that relatedness can replace or complement $\mathrm{ECI}^{\mathrm{emp}}$ in our formulation and concludes that complexity and relatedness describe co-existing influences on work location choices.

Reviewer 2

> *The authors have made a great contribution with this manuscript and have followed all comments and suggestions I wrote during the revision.*
>
> *I truly appreciate the analysis of informality rates between CDMX and the ZMVM, as well as the efforts to make this more transparent. Additionally, I appreciate that the authors included an additional visualisation (Supplementary Figure 14) for this specific topic.*

We thank the reviewer for this assessment and for the earlier comments that led to the metropolitan-area comparison for Mexico City. No further changes were required.

Reviewer 3

> *I co-reviewed this manuscript with one of the reviewers who provided the listed reports. This is part of the Nature Communications initiative to facilitate training in peer review and to provide appropriate recognition for Early Career Researchers who co-review manuscripts.*

We thank the co-reviewer for their contribution to the evaluation of this manuscript. No changes were required.

Reviewer 5

> *The authors have addressed all of my previous comments carefully and thoroughly. The manuscript has improved substantially since the initial submission, and I congratulate the authors on their work.*

We thank the reviewer for this assessment and for the earlier comments that improved the manuscript, including the renaming of the relatedness measure. No further changes were required.
